# Plastics and Sustainable Development – Identifying and Quantifying Ecodesign Strategies for Plastics

**DOI:** 10.1002/gch2.202500033

**Published:** 2026-03-23

**Authors:** Venkateshwaran Venkatachalam, Sebastian Spierling, Mikołaj Owsianiak, Frederik R. Wurm, Leonie Barner, Hans‐Josef Endres

**Affiliations:** ^1^ Institute of Plastics and Circular Economy Leibniz Universität Hannover Garbsen Germany; ^2^ Section for Quantitative Sustainability Assessment Department of Environmental and Resource Engineering Technical University of Denmark Lyngby Denmark; ^3^ Centre for Absolute Sustainability Technical University of Denmark Lyngby Denmark; ^4^ Sustainable Polymer Chemistry Department of Molecules and Materials Faculty of Science and Technology MESA + Institute for Nanotechnology University of Twente Enschede The Netherlands; ^5^ School of Chemistry and Physics Faculty of Science Centre for Environment and Society Queensland University of Technology Brisbane Queensland Australia

**Keywords:** ecodesign, plastic, recycling, resource efficiency, sustainable development

## Abstract

In 2015, the United Nations introduced the 17 Sustainable Development Goals (SDGs) to address major global challenges in sustainable development. Different industrial sectors are held accountable by their stakeholders for how their actions can contribute to achieving these goals. Among others, the plastic sector has come under scrutiny due to its linearity, characterized by low recovery rates after use and adverse effects on the environment resulting from uncontrolled disposal. Therefore, focus should be given not only to improving production processes and developing regulatory frameworks to manage plastic wastes but also to product design, as composition and construction determine whether products can be effectively recovered and recycled after use. Ecodesign is an approach that seeks to integrate different environmental considerations as early as during the design phase by focusing on the entire lifecycle of a product. To understand how plastics influence various dimensions of sustainability, a systematic literature review was conducted to examine the impact of plastics across different indicators of SDGs and their relationship to different ecodesign strategies. Based on the findings of the review, this study aims to identify plastic‐specific ecodesign strategies that can enhance resource efficiency and material recoverability. Furthermore, to compare and quantify these ecodesign strategies, a new methodological framework is proposed.

## Graphical abstract

A comprehensive literature review was conducted to understand how the scientific literature addresses plastics in relation to different aspects of sustainable development. From the qualitative assessment of the literature, the impacts of plastics across their lifecycle on sustainable development were identified, along with the relationship of the literature to different ecodesign strategies. Based on this assessment, plastic‐specific ecodesign strategies were identified, and a methodological framework was proposed to quantify them.

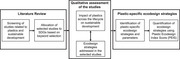

## 1 Introduction

The magnitude at which plastic is produced and consumed across the world has resulted in an exponential increase in the generation of plastic waste [1]. The plastic sector, unlike other materials such as glass and metals, has so far been mostly linear, using fossil resources to produce plastic products and disposing of them after use with limited focus on recovery and recycling [2]. In countries that lack sufficient waste management facilities, this linear use has contributed to widespread mismanagement and uncontrolled disposal of plastic waste into the environment. This has led to an unprecedented accumulation of macro‐, micro‐ and nanoplastics [3, 4, 5]. Therefore, plastic wastes can end up in ecosystems such as oceans [6], lakes [7], rivers [8], wetlands [9], soils [10], and even the atmosphere [11], causing adverse impacts on both biotic and abiotic resources [12, 13, 14].

Global recycling rates of plastics remain low, highlighting structural challenges in plastic waste management. The Organization for Economic Co‐operation and Development (OECD) in its 2022 ‘Global Plastics Outlook’ report estimates that only 9% of global plastic waste was ultimately recycled. While 19% of it was incinerated, 50% went to sanitary landfills and the remaining 22% of global plastic waste was disposed of in uncontrolled dumpsites, burnt in open pits or leaked into the environment [1, 15]. The drivers of plastic leakage extend beyond the plastic product itself and include, for example, pellet spills during production and transportation, inconsistent regulatory frameworks for waste handling and consumer practices. Therefore, although plastics are often described as recyclable, recyclability in itself does not guarantee that plastic waste is actually recovered after use. Effective recovery of plastic waste depends on systemic factors such as establishing adequate waste recovery infrastructure [15, 16]; replacing and recovering harmful additives used in plastics; prioritizing reuse and refurbishment over recycling in accordance with the waste hierarchy [17, 18] (Prevention → Reuse → Recycling → Recovery → Disposal); and fostering markets for plastic recyclates [18]. Addressing these interconnected factors is essential to reduce plastic leakage and improve resource efficiency across the value chain.

To quantify resource consumption and environmental impacts of plastics across their lifecycle, various assessment approaches exist. Some of the frequently used approaches include: (1) Material Flow Analysis (MFA) [19] which analyses the throughput (inputs and outputs) of process chains comprising extraction, transformation, manufacturing, consumption, recycling and disposal of materials [20]; (2) Life Cycle Assessment (LCA) [21] which evaluates potential environmental impacts throughout a product's lifecycle [22]; (3) Material Input Per Unit of Service (MIPS) [23], which refers to the material consumption from cradle‐to‐cradle per unit of service or function [24]; (4) Eco‐efficiency [25], which examines the efficiency of economic activity with regards to nature's goods and services [26].

These approaches are typically applied after a product has been developed to quantify impacts across its lifecycle. However, they offer limited guidance for integrating resource efficiency optimization during the design stage. To enhance the sustainability performance of plastics across their lifecycle, an approach is needed that not only quantifies the resource consumption and environmental impacts, but also evaluates how design choices influence product lifespan, material composition, repairability and recyclability. These aspects must be identified and evaluated by plastic manufacturers as early as the product design phase.

### 1.1 Ecodesign

Ecodesign is an approach that aims to minimize environmental impacts and maximize resource efficiency across a product's value chain during the product development stage [27]. The value chain refers to lifecycle stages of the product like raw material acquisition, production, processing, distribution, use, transport, disposal, and recovery, involving multiple stakeholders. During product development, product properties and the value chain are analyzed to identify shortcomings that limit resource efficiency and recoverability. Ecodesign strategies can then be applied to design new products by re‐evaluating existing product systems and value chains [27]. Moreover, ecodesign strategies can be implemented at different levels (process, product, sector, regional, national) to increase resource efficiency and the recoverability of products after use.

Because ecodesign explicitly considers the entire value chain of products in use, it provides a suitable basis for designing future products with improved resource efficiency across their lifecycle. For this reason, the ecodesign approach was adopted in this study in preference to other approaches such as LCA, MIPS or eco‐efficiency. While other approaches are effective in quantifying impacts after product development, ecodesign enables the identification of challenges and design‐related measures that influence the sustainable production, consumption and recovery of plastics at an early stage.

Common ecodesign strategies include, but are not limited to, implementing new material alternatives, optimizing production processes, waste recovery in production, creating new business models for recyclates, and eliminating hazardous chemicals in products. Examples of some of the ecodesign strategies implemented by organizations in the plastic sector are shown in Table S1 (SI1).

Beyond identifying strategies, their progress across sectors and regions must be assessed and quantified over time. Quantifying ecodesign strategies helps sectors set goals and benchmarks to improve resource efficiency across regions. Although recent studies have examined ecodesign strategies for plastics [28, 29, 30] and their role as decision‐support tools for sustainable production and use of products [31, 32], very few studies have focused on quantifying them [33, 34, 35]. Moreover, while plastic pollution and its adverse impacts on human health and ecosystems are well documented [36, 37], limited research has explored the relationship between plastics, sustainable development and the Sustainable Development Goals (SDGs) [38, 39, 40]. These studies examine the contributions and impacts of plastics across the SDGs but do not focus on ecodesign strategies. To date, no study has proposed a framework that identifies and quantifies plastic‐specific ecodesign strategies based on the impact of plastics across different aspects of sustainable development.

### 1.2 Objectives

The main objective of this study is to understand the effects of plastics across the value chain on different aspects of sustainable development and to identify plastic‐specific ecodesign strategies. To achieve this objective, a comprehensive literature review was conducted using Scopus. Studies were identified in Scopus by combining the terms ‘plastics’ and ‘polymers’ with keywords derived from the indicators and targets of the SDG framework [41], which were chosen to capture the environmental, economic and social impacts of plastics discussed in the scientific literature.

Using a set of screening criteria, the selected studies were allocated to the 17 SDGs and subsequently assessed for their interactions with other SDGs, based on the Scopus mapping framework and the authors’ own evaluation.

The beneficial and adverse impacts of plastics on the environment, economy and society, as mentioned in the selected studies, were then qualitatively analyzed. This was followed by a qualitative assessment of the relationship between the selected studies and the ecodesign strategies proposed in the Life Cycle Design Strategy (LiDS) Wheel [42]. Based on the observations from this qualitative assessment, plastic‐specific ecodesign strategies and corresponding parameters for those strategies were identified.

Building on this analysis, a preliminary methodological framework was proposed to calculate the Plastic Ecodesign Index Score (PEIS), based on the SDG Index score methodology [43]. PEIS enables comparison and quantification of the progress of ecodesign strategies at the product, sector, or regional level for a defined time. A practical example demonstrating the application of PEIS to a plastic product is provided, followed by a discussion of the study's limitations and the challenges associated with identifying and implementing ecodesign strategies.

Understanding these ecodesign strategies and implementing a methodological framework such as PEIS can help the plastic sector develop a roadmap to address future challenges related to product recovery. Quantifying the ecodesign strategies can subsequently support their progress in designing products that can be recovered and recycled after use.

The paper is structured as follows. Section 2 discusses the screening criteria for the literature review and the allocation of selected studies to the 17 SDGs, followed by the qualitative assessment of the studies and their relationship to different ecodesign strategies. Section 3 presents the results and observations of the qualitative assessment, followed by the preliminary identification of plastic‐specific ecodesign strategies. Section 4 introduces the PEIS framework and provides an illustrative example of its application. Section 5 outlines the limitations of the study and the challenges associated with identifying and implementing ecodesign strategies for plastics. Finally, Section 6 concludes the paper by summarizing the main findings of the study and discussing how the plastic sector can identify and implement ecodesign strategies in the future.

## 2 Materials and Methods

**FIGURE 1:**
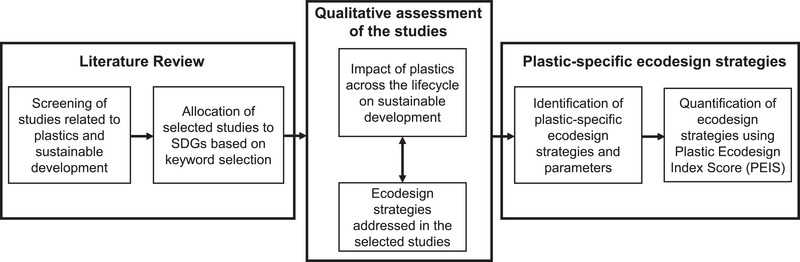
Conceptual framework of the study illustrating how the literature on plastics and sustainable development was assessed to identify and quantify plastic‐specific ecodesign strategies.

This section describes the screening of studies for the literature review, as well as the methods used to qualitatively assess the impact of plastics on different aspects of sustainable development and the relationship of the selected studies to different ecodesign strategies. The conceptual framework adopted in this study to identify and quantify plastic‐specific ecodesign strategies is shown in Figure 1.

### 2.1 Literature Review

The literature review for this study was carried out using Scopus. Although there exist different search engines like Web of Science and Google Scholar, Scopus was selected because it has developed a framework based on extensive keyword queries and machine learning that maps publications to different SDGs [44]. At the time of conducting this review, this SDG‐mapping framework was not available in other search engines.

After assigning the publications to different SDGs based on keyword searches, a qualitative assessment was carried out by the author team to evaluate the impact of plastics on sustainable development and to examine the relationship between the selected studies and different ecodesign strategies.

#### 2.1.1 Review Strategy

The authors of this study collectively defined the screening criteria for publications to be included in the literature review and allocated to different SDGs. Keywords were derived from the indicators and targets of all 17 SDGs [41]. For each SDG, the selected keywords were combined with the terms ‘Plastic’ and ‘Polymer’ using Boolean operators (AND, OR, AND NOT).

Initially, keyword searches were conducted within article titles only and were subsequently extended to include title, keywords, and abstracts in Scopus. The search timeframe was set from 2015 (as the 17 SDGs were introduced by the UN in 2015 [45]) to 30^th^ May 2024. The keywords used for each SDG, along with the studies considered in this review, are presented in Table 1.

**TABLE 1:** Studies considered for the literature review along with the keywords used in each SDG.

Nr.	SDG	Keywords used for the review in each SDG (in addition to Plastic/Polymers)	Studies considered for this review
1	No poverty	Poverty, people, population	[48, 49, 50, 51, 52, 53, 54, 55, 56, 57]
2	Zero hunger	Agriculture, food, food‐security, hunger	[58, 59, 60, 61, 62, 63, 64, 65, 66, 67, 68, 69, 70, 71, 72, 73, 74, 75, 76, 77]
3	Good health and well‐being	Health, death, illness, disease, mortality	[78, 79, 80, 81, 82, 83, 84, 85, 86, 87, 88, 89, 90, 91, 92, 93, 94, 95, 96, 97]
4	Quality education	Education, skill, awareness, qualification	[98, 99, 100, 101, 102, 103, 104, 105, 106, 107, 108, 109, 110, 111, 112, 113, 114, 115, 116, 117]
5	Gender equality	Gender, women, equality, policy	[118, 119, 120, 121, 122, 123, 124, 125, 126, 127, 128, 129, 130, 131, 132, 133, 134, 135, 136, 137]
6	Clean water and sanitation	Water, sanitation, drinking, scarcity	[138, 139, 140, 141, 142, 143, 144, 145, 146, 147, 148, 149, 150, 151, 152, 153, 154, 155, 156, 157]
7	Affordable and clean energy	Energy, renewable, sustainable, clean	[158, 159, 160, 161, 162, 163, 164, 165, 166, 167, 168, 169, 170, 171, 172, 173, 174, 175, 176, 177]
8	Decent work and economic growth	Economy, job, growth, employment	[178, 179, 180, 181, 182, 183, 184, 185, 186, 187, 188, 189, 190, 191, 192, 193, 194, 195, 196, 197]
9	Industry, innovation, and infrastructure	Innovation, research, infrastructure, industry	[198, 199, 200, 201, 202, 203, 204, 205, 206, 207, 208, 209, 210, 211, 212, 213, 214, 215, 216, 217, 218, 219, 220, 221, 222, 223, 224, 225, 226, 227]
10	Reduced inequalities	Income, injustice, inequality, social	[228, 229, 230, 231, 232, 233, 234, 235, 236, 237, 238, 239, 240, 241, 242, 243, 244, 245, 246, 247]
11	Sustainable cities and communities	Cities, settlements, communities, municipal, waste	[248, 249, 250, 251, 252, 253, 254, 255, 256, 257, 258, 259, 260, 261, 262, 263, 264, 265, 266, 267]
12	Responsible consumption and production	Sustainable, production, consumption, efficiency, footprint, waste	[268, 269, 270, 271, 272, 273, 274, 275, 276, 277, 278, 279, 280, 281, 282, 283, 284, 285, 286, 287, 288, 289, 290, 291, 292, 293, 294, 295, 296, 297]
13	Climate action	Climate change, mitigation, global warming	[298, 299, 300, 301, 302, 303, 304, 305, 306, 307, 308, 309, 310, 311, 312, 313, 314, 315, 316, 317, 318, 319, 320, 321, 322, 323, 324, 325, 326, 327]
14	Life below water	Marine, pollution, coast, fishing	[328, 329, 330, 331, 332, 333, 334, 335, 336, 337, 338, 339, 340, 341, 342, 343, 344, 345, 346, 347, 348, 349, 350, 351, 352, 353, 354, 355, 356, 357]
15	Life on land	Ecosystem, soil, land, terrestrial, wildlife, wetlands	[358, 359, 360, 361, 362, 363, 364, 365, 366, 367, 368, 369, 370, 371, 372, 373, 374, 375, 376, 377, 378, 379, 380, 381, 382, 383, 384, 385, 386, 387]
16	Peace, justice, and strong institutions	Justice, peace, regulation, legislation, governance, policy	[388, 389, 390, 391, 392, 393, 394, 395, 396, 397, 398, 399, 400, 401, 402, 403, 404, 405, 406, 407]
17	Partnerships for the goals	Partnership, cooperation, global, trade, market	[408, 409, 410, 411, 412, 413, 414, 415, 416, 417, 418, 419, 420, 421, 422, 423, 424, 425, 426, 427]

The co‐occurrence of keywords within each SDG was analyzed and visualized using VOSViewer [46]. VOSViewer is a tool used to visualize bibliometric networks of keywords between different studies. It can also assess the strength and frequency of keywords used across studies. Analyzing keyword co‐occurrence helps us understand the significance and frequency of keywords when searching for the literature based on the defined screening criteria. The keyword co‐occurrence diagrams for all 17 SDGs are provided in Figures S1–S18 (SI1). A list of frequently used keywords and their occurrences for individual SDGs is also included in the Supporting Information (SI2 Page 27).

#### 2.1.2 Screening of Literature Related to Plastics and Sustainable Development

In total, 1994 studies were identified across the 17 SDGs based on the keyword search. These studies were subjected to an extensive screening process that involved evaluating titles, abstracts, accessibility of the publications (availability to view and download the publications), relevance to the objectives of the study, and potential duplicates.

The screening criteria for inclusion in the review were as follows: (1) Only studies focusing explicitly on plastics were considered; studies addressing composites (primarily focused on natural, carbon or glass fibers and less on plastic use and recovery) or other materials such as glass were excluded; (2) Combining the keyword ‘plastic’ with keywords like ‘women’, ‘health’ and ‘skills’ resulted in studies related to plastic surgery, plasticity, implants (despite adding the ‘AND NOT’ operator during the search), which after inspection were not considered; (3) Although the literature search was primarily conducted in English, studies published in other languages were excluded; (4) Studies that were inaccessible (due to subscription restrictions or lack of an electronic version) were excluded, even if they addressed SDG‐related keywords; (5) Only original research articles and review studies were included; reviews of reviews were excluded to avoid redundancy with the observations; (6) Studies addressing municipal wastes or waste management without a specific focus on plastics were excluded, as such wastes follow different end‐of‐life pathways.

**FIGURE 2:**
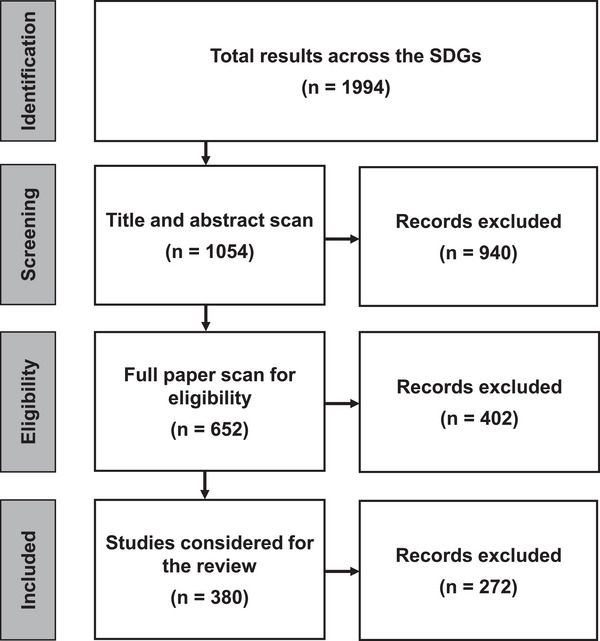
Screening analysis of studies considered for the literature review (Reproduced under terms of the CC‐BY license [47]).

After applying these criteria, 380 studies were selected for the comprehensive literature review. The screening analysis of the studies considered for the literature review is shown in Figure 2. The number of studies identified and selected for each SDG is provided in the Supporting Information (SI2 Page 2).

These 380 studies allocated to 17 SDGs were then thoroughly assessed with respect to two main aspects:

1. The beneficial and adverse impacts of plastics across different lifecycle stages on sustainable development
2. The ecodesign strategies addressed in the selected studies

The assessment methodology for these aspects is described in the following sections. Based on this analysis, plastic‐specific ecodesign strategies were identified, and a framework to quantify these strategies was proposed. The studies allocated to each SDG, along with the keywords used in the review, are presented in Table 1.

### 2.2 Qualitative Assessment of the Studies

This section describes the qualitative assessment of the impacts of plastics on different aspects of sustainable development and the relationship between the selected studies and ecodesign strategies. The list of studies included in the review, along with the results of the qualitative assessment, is provided in the Supporting Information (SI2 Pages 3–9).

#### 2.2.1 Impact of Plastics Across the Lifecycle on Sustainable Development

To assess the impact of plastics across the entire value chain in relation to sustainable development, both beneficial and critical impacts were identified using a qualitative scoring system. The lifecycle stages considered in this assessment are: (a) Raw material stage—Cracking of crude oil, production of polymers; (b) Production stage—Conversion of polymers into plastic products, and the addition of additives and chemicals during processing; (c) Use stage—Product use, maintenance, and repair, as well as associated resource consumption; (d) End‐of‐life—Recovery and disposal pathways documented in the literature; (e) Transportation/Logistics—Transportation and distribution of polymers and plastic products, exports of plastic products and waste, and the environmental transport of macro‐, meso‐, micro‐, and nanoplastics.

Environmental, economic and social impacts were considered across all lifecycle stages. Each of the 380 studies was qualitatively assessed and scored by the author team based on the criteria presented in Table 2. The scoring system was developed by the author team for this study based on domain expertise.

**TABLE 2:** Scoring system used to assess the impacts of plastics on sustainable development in the reviewed literature.

Aspects assessed	Impact	Points
Beneficial impacts of polymer/plastic in each life cycle stage on environment, economy and society being the primary focus of the study	Highly Beneficial	+3
Focus on the beneficial impacts of polymer/plastic in each life cycle stage on environment, economy and society, but is not the primary focus of the study	Beneficial	+2
Focus on both the beneficial impacts and the critical impacts of polymers/plastics in each life cycle stage on environment, economy and society	Neutral	+1
No mention of the benefits/critical impacts of polymers/plastics in each life cycle stage on environment, economy and society	Not Applicable	0
Focus on the critical impacts of polymers/plastics in each life cycle stage on environment, economy and society, but is not the primary focus of the study	Critical	−2
Critical impacts of polymers/plastics in each life cycle stage on environment, economy and society being the primary focus of the study	Highly critical	−3

An illustrative example of the qualitative assessment is provided using the study by Zhang et al. [76] which was allocated to SDG 2 in this review. This study examined the use of plastic mulch to increase crop yields and the adverse effects of residual plastic film accumulated in soil [76]. Increased crop yields of 25–42% due to improved soil temperature and moisture [76] were classified as ‘Beneficial’ impacts (+2 Points) during the use stage. However, the use of additives like phthalates in mulch films was assessed as having ‘Critical’ impacts (−2 Points) in the raw‐material and production stages due to associated health risks [76]. Furthermore, the accumulation of plastic film residues in agricultural soils, combined with limited recovery infrastructure, was assessed as having highly critical impacts (−3 Points) at the end‐of‐life stage [76]. As transportation‐related impacts were not discussed in the study, the impacts for this stage were assessed to be ‘Not Applicable’ (0 Point).

Following this approach and based on the scoring criteria shown in Table 3, all 380 studies were qualitatively assessed and scored across all lifecycle stages. For each SDG, the sum of the total points secured by every study for each lifecycle phase was then compared with the maximum beneficial and critical points that could be scored by a set of literature for that SDG. For example, if 30 publications are assigned to an SDG for review, then the total points that could be scored by those 30 publications in that SDG could range from +90 to −90 (30 studies with highly beneficial or highly critical impacts) and based on this range, the total points to be beneficial, neutral and critical were assigned. The aggregated results were visualized in the form of a heat map, which are discussed in Section 3.1. Detailed scoring results are provided in the Supporting Information (SI2 Pages 10–19).

**TABLE 3:** Ecodesign strategies and the associated parameters for the product development, based on LiDS Wheel [42].

Strategy	Parameters
Low‐impact materials	Cleaner materials Renewable materials Lower energy content materials Recycled materials Recyclable materials
Reduction of materials usage	Reduction in weight Reduction in (transport) volume
Optimization of production techniques	Alternative production techniques Fewer production steps Lower/cleaner energy consumption Fewer/cleaner production and consumables
Optimization of distribution system	Less/cleaner/reusable packaging Energy‐efficient logistics
Reduction of impact during use	Lower energy consumption Cleaner energy source Fewer consumables needed Cleaner consumables No waste of energy/consumables
Optimization of initial life	Reliability and durability Easy maintenance and repair Modular product structure Classic design Human centered design
Optimization of end‐of‐life system	Re‐use of product Remanufacturing/Refurbishing Recycling of materials
New concept development	Dematerialization Shared use product Integration of functions Functional optimization of product (components) Efficient function fulfillment Frameworks and regulatory approaches

#### 2.2.2 Ecodesign Strategies Addressed in the Selected Studies

In addition to assessing plastic impacts, this study qualitatively examined the relationship between the selected literature and ecodesign strategies. The ecodesign strategies considered were based on the Life Cycle Design Strategy (LiDS) Wheel [42], which is a widely used qualitative framework for identifying ecodesign strategies [27]. The selected strategies and its associated parameters are shown in Table 3.

In addition to the strategies defined in the LiDS Wheel, the parameter ‘Frameworks and regulatory approaches’ (Table 3) was included under the ecodesign strategy ‘New concept development’. The inclusion of this parameter reflects the strong emphasis in the reviewed literature on national and international regulatory frameworks supporting material substitution and plastic alternatives.

All 380 studies were qualitatively analyzed to understand whether and how they addressed the selected ecodesign strategies and its parameters (Table 3). Relationships between these studies and strategies were evaluated and scored by the author team using criteria similar to those applied in the impact assessment (Section 2.2.1), as shown in Table 4.

**TABLE 4:** Assessment of the relationship between the studies and ecodesign strategies.

Description of relationship	Relationship	Points
Primary focus of the study on design related aspects or problems related to those aspects	Strong	3
Primary focus of the study not on design related aspects or problems related to those aspects, but were nevertheless addressed within the study	Moderate	2
Design aspects/problems mentioned in the study unclear when taken in the larger context	Unclear	1
No relationship between the design aspects/problems and the focus of the study	No	0

For example, if a study focuses on the impacts and recovery of additives and chemicals in plastic products, it was qualitatively assessed by the author team as having a ‘Strong’ relationship with the ecodesign strategies ‘Low‐impact materials’ and ‘Optimization of end‐of‐life system’ respectively. The qualitative assessment of the relationship between the selected studies and the ecodesign strategies is illustrated below using a specific example.

Egun et al. [143] was identified as one of the studies allocated to SDG 6 in the literature review. This study analyzed the Polyethylene Terephthalate (PET) bottle system in Nigeria and proposed an integrated PET bottle system for sustainable waste management [143]. The study emphasizes the use of recyclates in bottle manufacturing and the application of extended producer responsibility to reduce natural resource consumption and improve oversight of the plastic bottle supply chain [143]. In addition, it advocates the establishment of improved recovery and recycling infrastructure, as well as national policies for the sustainable management of used PET bottles in Nigeria [143].

Based on the observations and recommendations presented in this study, its relationship to different ecodesign strategies (Table 3) was qualitatively assessed (Table 4). The study was found to have a ‘Strong’ relationship with the ecodesign strategies ‘Low impact materials’, ‘Reduction of materials usage’, ‘Optimization of end‐of‐life system’ and ‘New concept development’. This assessment reflects the study's primary focus on the feasibility of establishing collection and recycling infrastructure for sustainable production and consumption of PET bottles in Nigeria, as well as challenges related to regulatory frameworks and consumer behavior.

However, the study also addressed additional aspects such as the production of recyclates, collection systems for PET bottles, extended producer responsibility, and extending the lifetime of PET bottles during the use stage. As these aspects were discussed as recommendations rather than the main focus of the study, the relationships with the ecodesign strategies ‘Optimization of production techniques’, ‘Optimization of distribution system’, ‘Reduction of impact during use’, and ‘Optimization of initial life’ were assessed as ‘Moderate’.

The contribution of studies from each SDG to the eight ecodesign strategies was subsequently aggregated and visualized using a Sankey diagram, which is discussed in Section 3.2.

## 3 Results and Discussion

The significant aspects and impacts of plastics on sustainable development were identified through the qualitative assessment of the literature. Based on this assessment, the most frequently discussed aspects within the studies allocated to each SDG are summarized in Table 5.

**TABLE 5:** Beneficial and critical aspects of plastics addressed across 380 studies toward sustainable development.

Goals	Aspects (Beneficial and Critical)
SDG 1 – No poverty	Difficulties in collection of plastic wastes (Critical) Ocean plastics and poverty of the people around (Critical) Implication of plastic bans on poor people (Neutral—Beneficial impacts for people during use stage (sales, durability) and critical impacts due to the use of single use plastic and disposal of wastes in the environment) Policy interventions to support informal recycling (Beneficial) Reuse of plastic wastes into useful products (Beneficial)
SDG 2 – Zero hunger	Use and impacts of plastic mulch films (Neutral—Beneficial for the increased crop yield but critical due to the improper disposal of films after use) Controlling plastic wastes in agriculture (Critical) Transfer of micro and microplastic from soils to food chains (Critical) Impacts of plasticizers in soils (Critical)
SDG 3 – Good health and well‐being	Impacts of plastic pollution on health (Critical) Occurrence of microplastic in food and humans (Critical) Use of face masks (Beneficial) Environmental risks of face masks disposal in the environment (Critical) Polymer contamination in water bottles (Critical) Plastic pollution in air and road dust (Critical)
SDG 4 – Quality education	Awareness in handling plastic wastes (Beneficial) Plastic use and pollution in education (Beneficial) Irresponsible handling and disposal of plastic wastes (Critical) Consumer behavior in handling plastic wastes (Critical)
SDG 5 – Gender equality	Gender based determinants for handling and disposing household plastic wastes (Critical) Phthalate exposure on pregnant women and female hygiene products (Critical) Women's activism against plastic pollution (Critical) Women empowerment through plastic recycling (Beneficial)
SDG 6 – Clean water and sanitation	Use of sachet water—Clean water (Beneficial) and plastic pollution (Critical) Microplastic contamination in water, wastewater plants (Critical) Blockage of sewage and rivers due to plastic wastes (Critical) Use of plastic in women's sanitation practices in informal settlements (Neutral—Beneficial due to the prevention of sanitation related diseases and critical due to the increase in the generation of plastic wastes that are not recovered and disposed properly)
SDG 7 – Affordable and clean energy	Conversion of plastic into fuels (Beneficial) Use of plastics in renewable energy technologies (wind turbines, supercapacitors) (Beneficial) Chemical recycling—Pyrolysis of plastics (Beneficial)
SDG 8 – Decent work and economic growth	Policy interventions for Circular Economy in plastic industries (Critical due to the adverse effects of plastic pollution) Job creation in the plastic sector (Beneficial) Cost structure for recycling plastic wastes (Neutral—Beneficial in some countries and critical in countries in the Global South) Economic growth (Beneficial) and (ocean) plastic pollution (Critical)
SDG 9 – Industry, innovation, and infrastructure	Recycling infrastructure for plastic wastes (Critical) Innovation in biodegradable plastics (Neutral) Businesses tackling ocean plastic pollution (Neutral) Digitalization and technological innovation in formal and informal plastic recycling (Beneficial)
SDG 10 – Reduced inequalities	Inequalities among the Global North‐South plastic waste trade (Critical) Plastic pollution as a threat for indigenous population (Critical) Economic, social and environmental implications in the informal recycling of plastic wastes (Critical) Inequal benefits and burdens of plastic products on human health (Neutral—Beneficial for the use of plastic products in medical applications but critical in the case of additives and single use bio wastes on human health)
SDG 11 – Sustainable cities and communities	Barriers to reduce single use plastics (Critical) Packaged water consumption in cities (Beneficial) and the accompanying pollution (Critical) Plastic recycling practices in different cities across the world (Critical) Ban of plastic waste imports and its effects (Beneficial)
SDG 12 – Responsible consumption and production	Upcycling of plastic wastes (Beneficial) Circular economy routes for plastic wastes (Beneficial) Challenges and tradeoffs of using bioplastics (Neutral) Efforts and challenges to increase the recyclability of post‐consumer plastic wastes (Neutral—Beneficial or producing new business models but critical due to the lack of infrastructure in different countries)
SDG 13 – Climate action	Climate change and marine plastic pollution (Critical) Fossil fuels and climate change (Critical) Plastic mulching and greenhouse gas emissions (Neutral—Beneficial for the crops but critical when it comes to the production of materials and generation of wastes from sustainability point of view) Plastic recycling and climate change mitigation strategies (Neutral—Beneficial from the environmental point of view but critical due to the poor recycling rate of plastics)
SDG 14 – Life below water	International policies, norms and responses to curb marine plastic pollution (Critical) Impacts of plastic pollution on marine wildlife (Critical) Biodegradable plastics to combat ocean pollution (Neutral—Beneficial if they really degrade else can cause critical impacts on the environment)
SDG 15 – Life on land	Plastic pollution in wetlands, freshwater ecosystems, lakes (Critical) Microplastics in flora and fauna (Critical) Soil under stress due to microplastic pollution (Critical)
SDG 16 – Peace, justice and strong institutions	Plastic pollution as a form of injustice (Critical) Marine plastic pollution in Asia (Critical) Role of corporations in global governance of plastic pollution (Critical) Singe use plastic policies in vulnerable communities (Critical)
SDG 17 – Partnership for the goals	(Global) governance of ocean plastic pollution (Critical) Entrepreneurial partnerships for handling plastic wastes Regional co‐operation in plastic waste cleanup (Critical) Citizen science and corporate social responsibility for combating the plastic pollution (Critical) Partnerships between academia and industry on handling plastic wastes and circular economy (Beneficial)

Table 5 shows that most studies allocated to the 17 SDGs focused on the impacts associated with the use of plastic products, as well as the impacts of recovering and disposing of plastic waste on the environment. However, with the exception of studies allocated to SDGs 8 and 9, which focused on the economic impacts of plastics, most studies in the review emphasized the environmental and social impacts of plastics.

Studies allocated to SDGs 3, 5, 6, and 10 primarily examined the impacts of plastics on human health. These included issues such as the presence of phthalates in plastic products and their effects on women's reproductive health, microplastic contamination in drinking water and wastewater treatment plants, plastic pollution affecting indigenous populations and challenges associated with the disposal of plastic products in medical applications. Studies assigned to SDGs 4 and 12 focused on increasing awareness among stakeholders across the plastic value chain regarding the recovery and recycling of plastic products after use.

Although many studies under SDG 12 emphasized the beneficial aspects of plastics being recyclable and use of recyclates in production, they also highlighted challenges related to waste recovery in certain regions and low recycling rates, even in some developed countries. Studies allocated to SDGs 6, 11, and 13 addressed the challenges and impacts of plastic pollution, including discussions on banning single‐use plastic products. Studies associated with SDGs 14–17 primarily focused on policy and legal frameworks aimed at curbing plastic pollution and preventing the illegal export of plastic waste to countries in the Global South (low‐ and middle‐income regions with comparatively limited waste management infrastructure).

Only a limited number of studies (allocated to SDGs 1, 2, 7, and 12) highlighted the versatility (beneficial aspect) of plastics in sectors such as agriculture (e.g., mulch films), medical applications (e.g., face masks and surgical instruments), food systems (e.g., packaged drinking water, take‐away food, and vegetables) and energy (e.g., wind turbines and incineration of plastic wastes with energy recovery). However, even those studies also discussed the critical challenges (critical aspect) related to the disposal and recovery of plastics after use.

In addition to identifying beneficial and critical aspects across the SDGs, the co‐occurrence of significant keywords was visualized in the form of bibliometric analysis using VOSViewer [46]. These visualizations, provided for all SDGs in Figures S1–S18 (SI1), illustrate the themes emphasized in the scientific literature when examining plastics and sustainable development. Furthermore, chord diagrams showing interactions between studies with other SDGs were generated using Flourish and are presented in Figures S20–S37 (SI1). Together, these analyses support the identification of plastic‐specific ecodesign strategies.

### 3.1 Impact of Plastics Across the Life Cycle on Sustainable Development

**FIGURE 3:**
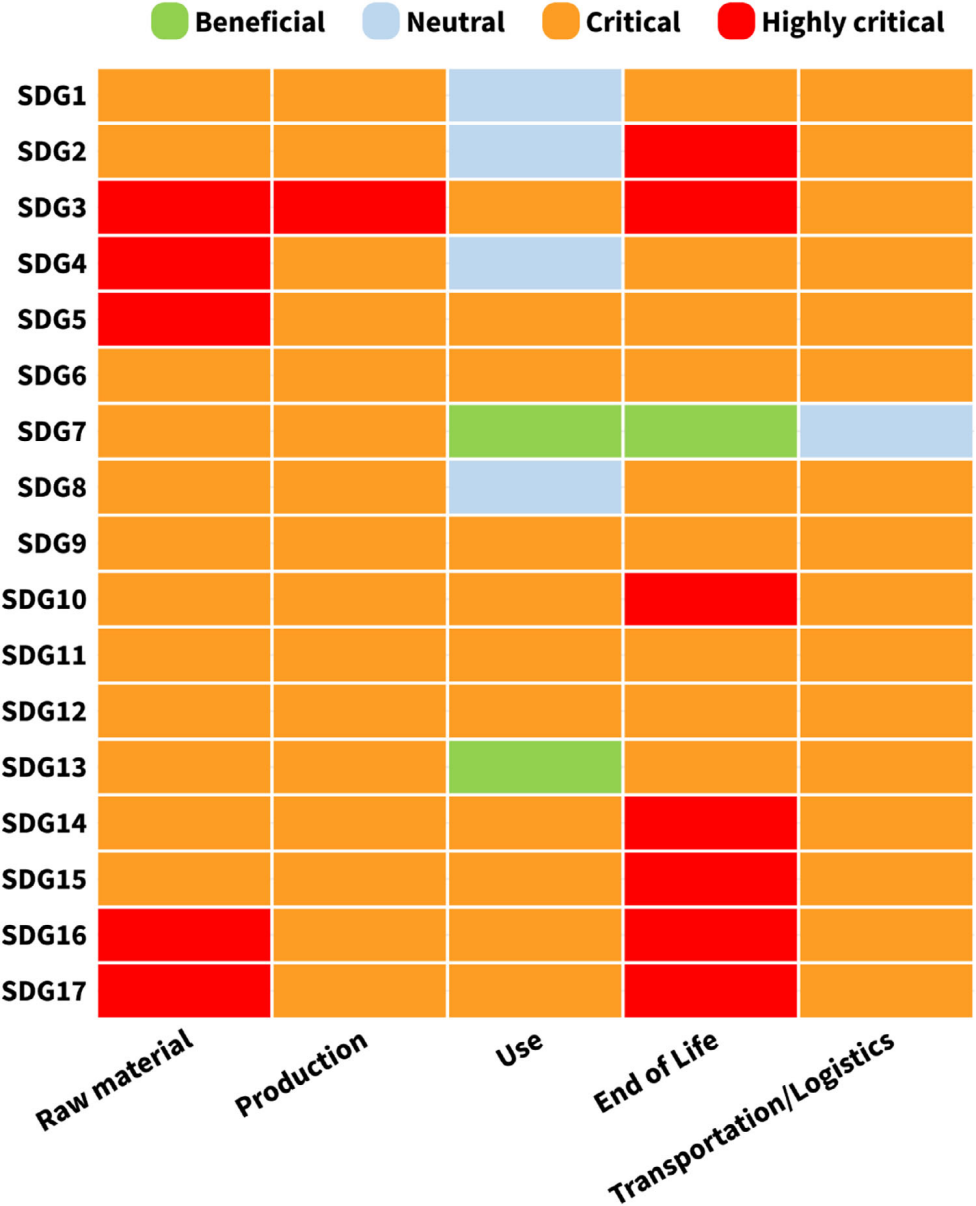
Impacts of plastics discussed in the literature on sustainable development across all the lifecycle stages for 17 SDGs in the form of a heatmap. Different lifecycle stages of plastics in X‐axis and the studies allocated to 17 different SDGs in Y‐axis (Created with Flourish [428]).

The impact of plastics across the lifecycle in relation to different SDGs was assessed using the aspects of the plastic value chain outlined in Table 5 and the scoring methodology described in Section 2.2.1. The resulting impacts across different lifecycle stages for the studies allocated to the 17 SDGs are visualized in the form of a heatmap in Figure 3.

The heatmap shows that studies allocated to nearly all SDGs exhibit critical to highly critical impacts across different lifecycle stages. In particular, studies associated with SDGs 3–6, 9–12 and 14–17 were assessed as having critical to highly critical impacts across all lifecycle stages of the plastic value chain. This outcome is partly influenced by the choice of keyword combinations related to plastics and polymers, together with indicators of sustainable development from the SDGs such as ‘poverty’, ‘health’, ‘inequality’, ‘awareness’, ‘education’, ‘injustice’, ‘infrastructure’, ‘sanitation’ and ‘pollution’. The results (publications) for these keywords highlighted recurring critical issues associated with plastic use in society and its consequences for sustainable development.

Using the scoring system (Section 2.2.1), aggregated scores across lifecycle stages resulted predominantly in critical impacts for most SDGs. The heatmap also reveals beneficial, neutral and highly critical impacts for certain lifecycle stages, which are discussed below. Detailed scoring results and aggregated SDG scores are provided in the Supporting Information (SI2 Pages 10–19).

- Studies allocated to SDG 7 (Affordable and clean energy) and SDG 13 (Climate action) showed beneficial impacts during the use stage of the plastic value chain. For SDG 7, beneficial impacts were also observed at the end‐of‐life stage due to the potential of plastic waste as a fuel source. Incineration and chemical recycling were discussed as a contribution to global decarbonization strategies. For example, the World Economic Forum reported that scaling up of chemical recycling of hard‐to‐recycle plastics could produce nearly 7.5 Mt of secondary feedstock by 2050, when combined with measures like reuse, reduce, design for recycling and mechanical recycling [429].
- Studies allocated to SDG 7 were assessed to have neutral impacts during the transportation stage, reflecting mixed findings regarding the benefits of importing and exporting plastic wastes for incineration versus the critical environmental impacts of long‐distance transport of plastic wastes. Similarly, studies allocated to SDG 8 exhibited neutral impacts during the use stage due to discussion on job creation and economic growth associated with plastic industries and recycling activities. Supporting this observation, the Organisation for Economic Co‐operation and Development (OECD) estimates that the circular economy (recovery of products after use, unlike the linear economy) sector could create around 2.5 million new jobs in the European Union by 2030, particularly in recycling, repair and reuse activities [430].
- Studies allocated to SDGs 1, 2, 4, and 8 were assessed to have neutral impacts during the use stage. Some studies addressed the improvements in sanitation and business opportunities among marginalized communities through the use of single‐use plastic bags. However, other studies under these SDGs highlighted critical impacts, including health risks from plastics entering the food chain (use and end‐of‐life stage), blockage of drainage systems and drinking water sources due to plastic litter (end‐of‐life stage), and loss of tourism in coastal regions affected by plastic pollution (end‐of‐life stage). It has been estimated that more than 200 million people are at high risk of plastic‐aggravated flooding globally [431] and projections suggest that plastic in the oceans could outweigh fish by mass within the next 15 years [432].
- Studies allocated to SDG 3, 4, 5, 16, and 17 were assessed to have highly critical impacts at the raw material stage. These studies emphasized the adverse health effects associated with oil cracking and petrochemical processes during polymer production, particularly for women and marginalized communities. The production and use of additives that contained phthalates were linked to hormonal imbalance and pregnancy complications, which are reflected in the highly critical impacts observed for the production stages in SDG 3 (Good health and well‐being). Globally, more than 350 000 cardiovascular deaths have been attributed to exposure to phthalates [433].
- Studies allocated to SDGs 2, 3, 10, 14, 15, 16, and 17 showed highly critical impacts at the end‐of‐life stages. These impacts were largely attributed to inadequate waste management infrastructure and the effects of microplastics and plastic pollution on human health and biodiversity. Uncollected plastic waste has been identified as the largest contributor to plastic pollution in the Global South, accounting for approximately 68% of plastic waste emissions and 85% of debris emissions in the Global South [434].

From Figure 3 and Table 5, it can be seen that even though polymers and plastic products have found applications and provided multiple benefits across different sectors, the chemicals needed to produce them already contribute to critical impacts on the environment. In addition, the presence of plastic particles in the environment, resulting from pellet losses during production and transportation as well as wastes generated during production and use, together with micro‐ and nanoplastics across the entire value chain, cause critical social, economic and environmental impacts globally.

### 3.2 Ecodesign Strategies Addressed in the Selected Studies

Besides evaluating the impacts of plastics on different aspects of sustainable development, this study examined the extent to which the selected studies addressed different ecodesign strategies. Based on the scoring criteria described in Section 2.2.2, the total contribution of studies allocated to each SDG toward each ecodesign strategy was evaluated. Individual and aggregated scores are presented in the Supporting Information (SI2 Pages 20–25).

**FIGURE 4:**
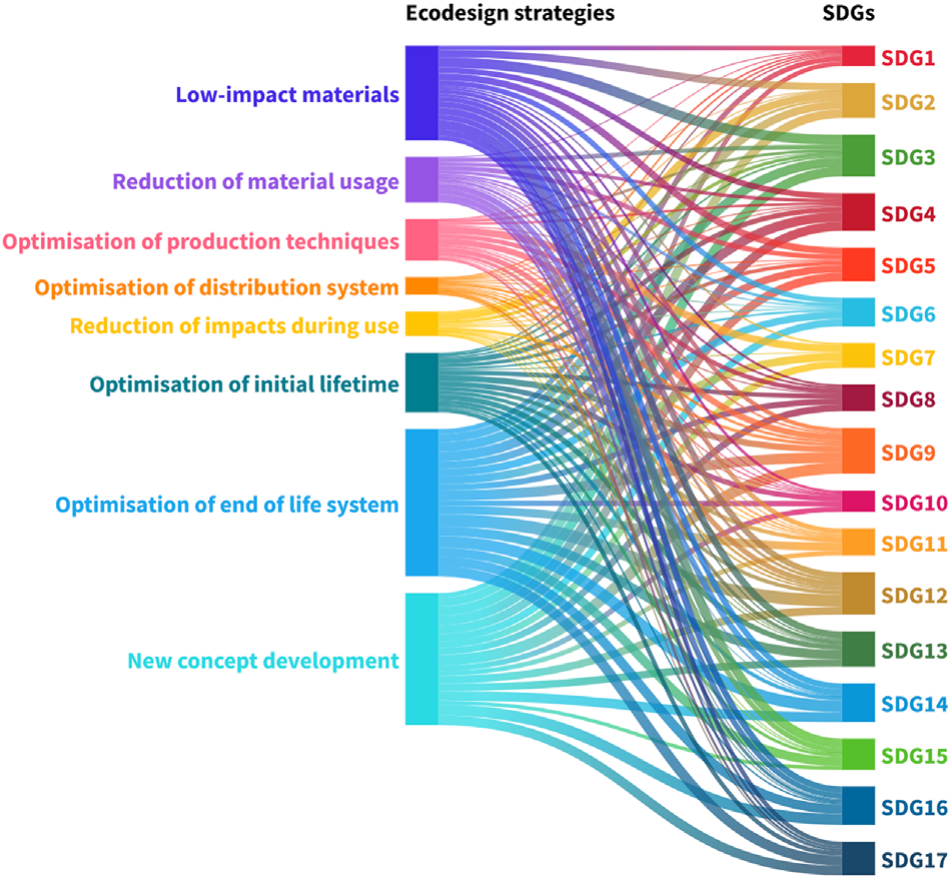
Sankey diagram showing the relationship between the assessed studies allocated to each SDG with different ecodesign strategies (Created with Flourish [428]).

The strength of relationships between the 17 SDGs and the ecodesign strategies listed in Table 3 is visualized using a Sankey diagram, as shown in Figure 4. The Sankey diagram helps to better understand the strength of the relationships between studies allocated to a given SDG and the different ecodesign strategies. The thickness of the flow lines in the Sankey diagram is proportional to the total score obtained by each SDG in relation to a specific ecodesign strategy. Where studies allocated to a particular SDG did not address a given ecodesign strategy, no flow is shown between them.

Additional Sankey diagrams illustrating the contributions of individual SDGs to different ecodesign strategies are provided in Figures S38–S54 (SI1). Key observations derived from these relationships are summarized in Table 6.

**TABLE 6:** Observations based on the relationship between the studies and ecodesign strategies and their contributions toward sustainable development.

Ecodesign strategy	Observations and contributions toward sustainable development
Low‐impact materials	Additives and microplastics that impact human health (SDG 3, 14) Plastic wastes that affect biodiversity (SDG 15) Biodegradable plastic mulches for agricultural yield (SDG 2)
Reduction of material usage	Reduction in weight and transport volume thereby preventing leakage and wastes (SDG 13, 14, 15, 16)
Optimisation of production techniques	Energy efficiency in processing of plastics and plastic wastes (SDG 7) Production processes and infrastructure involved in the recovery of plastic wastes (SDG 9, 12)
Optimisation of distribution system	Transport of products in single use plastic packaging and unchecked distribution of plastic wastes (SDG 12, 16)
Reduction of impacts during use	Less consumption and recovery of plastic mulch films from the soils (SDG 2) Infrastructure for reuse and repair (SDG 9, 12)
Optimisation of initial lifetime	Increase recovery facilities for plastic wastes in cities so that the materials stay within the system for further applications (SDG 8, 9, 10)
Optimisation of end‐of‐life system	Reuse and recycling of plastic wastes (SDG 4, 7, 9, 12, 13) Mitigate the impacts from the effects of microplastics (SDG 2, 3, 5, 6, 14, 15, 16)
New concept development	Regulatory frameworks, biodegradable plastics (SDG 2, 14, 15), business models for circular economy (SDG 7, 8) and investment in recycling infrastructure (SDG 9) along with sustainability assessment (SDG 11, 12, 13)

From Table 6 and Figure 4, some of the findings regarding the relationship between the studies and different ecodesign strategies are discussed below.

- Low‐impact materials: Studies allocated to SDG 7 showed a weak relationship with this strategy, as the studies focused primarily on the potential of plastic and plastic wastes as fuel sources when processed through pyrolysis or incineration. Furthermore, studies allocated to SDGs 8 and 10 also showed a weak relationship with this strategy, as most of the studies discussed job creation through plastic recycling and the challenges of plastic waste management in countries with inadequate recycling infrastructure. Studies that addressed the use of additives and their critical impacts on health, as well as studies focusing on alternative materials for plastics, demonstrated a stronger relationship with this strategy.
- Reduction of material usage: This strategy focusses on reducing material weight and volume during transportation thereby increasing resource and fuel efficiency. Studies allocated to SDGs 1 and 2 showed a weak relationship with this strategy, as most of these studies discussed the use and ban of plastics, along with the effects of plastics on agricultural yield and food systems. Studies allocated to SDGs 7 and 10 focused on different aspects of plastic waste management and therefore also showed a weak relationship with this strategy. In contrast, studies that addressed the transportation of plastic and plastic wastes across countries, measures to prevent leakage of plastic waste and the effects of plastic waste on terrestrial and marine environments demonstrated a stronger relationship with this strategy.
- Optimization of production techniques: This strategy addresses the need for fewer production steps and reduced consumption of resources during production. Studies allocated to SDGs 1, 2, 5, and 6 showed weak to no relationship with this strategy, as they primarily addressed the use of different plastic products and their effects on society, the economy and the environment. Studies allocated to SDGs 10, 14, and 16 focused more on plastic waste and its impacts and therefore also showed a weak relationship with this strategy. In contrast, studies that addressed aspects such as innovation in plastic production, the use of plastic wastes for energy and the recyclability of plastic waste were assessed as having a stronger relationship with this strategy.
- Optimization of distribution system: This strategy addresses the packaging of products after production and the logistics involved in transporting and recovering products. Accordingly, studies that examined regulatory frameworks for transporting plastic waste and the efficiency of recycling infrastructure showed a stronger relationship with this strategy. Studies allocated to SDGs 1–8, 10, 11, 13–15, and 17 showed a weak relationship with this strategy, as they focused less on the distribution and logistics of the plastic products before and after use. Among all ecodesign strategies, the studies considered in this review exhibited the weakest relationship with this particular strategy, despite the crucial role logistical aspects of transporting plastics and plastic waste play in product recovery.
- Reduction of impacts during use: As this strategy focuses on reducing resource consumption during the use stage, most of the studies assessed in this review showed a weak to no relationship with this strategy. Studies allocated to SDGs 7, 8, and 17 were assessed as having no relationship with this strategy, as they focused on aspects such as employment and energy generation from plastic waste rather than on resource efficiency during the use stage.
- Optimization of initial lifetime: This ecodesign strategy focuses on the design and lifetime of products, thereby increasing their repairability during use and recoverability after use. Studies allocated to SDGs 1–3, 6, 7, and 16 showed a weak relationship with this strategy, as these studies focused primarily on the use of plastic products or on the effects of plastic waste on society, the economy and the environment. They did not address how the design of plastic products could be optimized to extend product lifetime and enable recovery after use.
- Optimization of end‐of‐life system: Almost all of the 380 studies considered in this review showed a moderate to strong relationship with this strategy. Although studies allocated to SDGs 1, 2, 7, and 8 focused on the benefits of using plastic products and the potential of plastic wastes as fuel sources, they also acknowledged the need for improved end‐of‐life systems for plastic products currently in use worldwide. Studies allocated to SDGs 11–17 emphasized the need for adequate recovery and recycling infrastructure for plastics globally, which not only increases the resource efficiency and circularity of plastics in use but also helps prevent the leakage of plastic waste in the form of micro‐ and nanoplastics into the environment.
- New concept development: Studies focusing on innovations, policies, regulatory frameworks and investments related to the recovery, reuse and recycling of plastics showed a stronger relationship with this strategy. Studies allocated to SDG 9 demonstrated a strong relationship with this strategy, as most of these studies emphasized the need for improved recovery and recycling infrastructure for plastic waste. In addition, studies allocated to SDGs 14, 16, and 17 also showed a strong relationship with this strategy, as they primarily focused on the effects of plastic pollution in the environment alongside the legislative measures required to curb the uncontrolled disposal of plastic waste.

From Figure 4, it can be seen that studies allocated to SDG 9 exhibited a moderate to strong relationship with almost all ecodesign strategies. This can be attributed to the fact that these studies extensively discussed the need for improved design, innovation and infrastructure to recover and recycle plastics during and after use. Moreover, most studies in this review, when addressing plastics, focused more on recyclability and less on strategies related to reuse, repair, refurbishment and reduction.

The results further indicate that the majority of studies allocated to different SDGs predominantly focused on the ecodesign strategies ‘Optimization of end‐of‐life system’ and ‘New‐concept development’. In contrast, most studies were found to have weak to no relationship with ecodesign strategies such as ‘Optimization of production techniques’, ‘Optimization of distribution system’, ‘Reduction of material usage’, ‘Reduction of impacts during use’ and ‘Optimization of initial lifetime’. These strategies address aspects such as logistics, reuse, repairability and recoverability that must be considered during product design. This represents one of the major findings of this study, and implies that both industry and academia should place greater emphasis on identifying and developing sector‐specific ecodesign strategies at early stages of product development. Doing so is essential to retain resources within the system and to increase the lifetime and recoverability of plastics.

Identifying these strategies only after products have been produced can create a vicious cycle in addressing plastic pollution, as the products are already in use and the failure to recover them results in the loss of valuable resources to the environment. Therefore, based on the LiDS Wheel (Table 3) and the results of the qualitative assessment (Sections 3.1 and 3.2), a preliminary set of plastic‐specific ecodesign strategies, together with their associated parameters, is proposed in Table 7.

**TABLE 7:** Preliminary set of ecodesign strategies and its parameters for the plastics sector.

Nr.	Strategy	Parameter
1	Low‐impact materials	Less use of additives
2		Use of renewable materials
3		Use of recyclates in production
4		Recyclable materials after production
5	Reduction of materials use	Reduction in weight of products
6		Reduction in transport volume
7	Optimization of production	Renewable energy in production
8		Recovery of wastes during production
9		Reduced energy consumption
10		Recovery of auxiliary materials (waters, chemicals)
11	Optimization of distribution	Electric vehicle fleet
12		Reduced air transport
13		Recyclable packaging material
14		Reuse of packaging material
15	Reduction of impact during use	Labelling of products for recyclability
16		Increase in lifetime of products with multiple use (unlike single‐use)
17		Reduction in resource consumption during use
18	Optimization of lifetime	Availability of instructions to repair
19		Availability of spare parts
20		Ease of disassembly for repair
21	Optimization of end‐of‐life system	Products recovered for reuse, refurbish and recycle
22		Monomaterials used in the product
23		Price of recyclates from the product
24		Products that are incinerated after use
25		Products that are landfilled and safely disposed after use
26	New concept development	Product as service (leased, shared)
27		Data management for Sustainability Assessment
28		Take‐back facilities for products
29		Regional recycling infrastructure
30		Reduction of product use in regions with uncontrolled disposal

This preliminary set of strategies can be extended in the future to include additional parameters such as the quality and availability of recyclates, biodegradability and market mechanisms, depending on sector‐specific requirements. After the identification of plastic‐specific ecodesign strategies and associated parameters, it is essential to measure and quantify these strategies for their effective implementation during the product development phase.

## 4 Plastic Ecodesign Index Score (PEIS)

In order to measure and compare the progress of implementing plastic‐specific ecodesign strategies at an organizational or regional level, this study proposes a methodological framework based on the SDG Index score [43] to calculate the Plastic Ecodesign Index Score (PEIS). Previous studies [435, 436] have adapted the SDG Index score methodology to quantify the progress of localized targets related to the SDGs. In this study, however, the SDG Index score methodology is not applied to quantify SDG targets directly, but is instead used as a basis to develop a methodological framework to quantify selected ecodesign strategies in the plastic sector over a defined time period.

This framework can be extended to quantify and compare additional ecodesign strategies and their associated parameters for a product at specific organizational, sectoral, regional, national or international levels, either for a defined time period or across multiple years. The steps involved in calculating the PEIS of a product are presented below.

To illustrate this methodological framework, a representative packaging product made of High‐Density Polyethylene (HDPE) was assumed to be manufactured in Region 1. The individual ecodesign score for the strategy ‘Low‐impact materials’ was calculated for this packaging product in Region 1 and compared with corresponding scores for other regions (Region 2, 3, and 4) for the year 2024. As this is a fictitious example, both the ecodesign scores and the threshold limits for the selected indicators across different regions are illustrative and do not reflect actual performance.

Step 1. Definition of the time period, application, and application level

Before analyzing and quantifying ecodesign strategies, it is essential to define the time period and the level (company, region, country or globally) at which the strategies are to be quantified. This approach is comparable to the SDG Index Score methodology, which measures the progress of different countries at the national level toward the SDGs by comparing performance over time.

Another important aspect is to determine whether the selected ecodesign strategies and their associated parameters apply to a specific product, for example, a food packaging product made of HDPE, or to a group of products within a sector, such as packaging products used in transportation.

Step 2. Identification of ecodesign strategies and their parameters

In this study, 30 different parameters across eight ecodesign strategies were identified, as shown in Table 8. However, the number of strategies and the parameters is not fixed and may vary depending on the sector and product type. A critical step in defining these strategies and parameters is the assessment of data availability for each parameter.

**TABLE 8:** Parameters and corresponding indicators for the ecodesign strategy ‘Low‐impact materials’.

Parameter	Parameter (Full form)	Indicator	Unit
ed1_cleanmat	Less use of additives	Amount of harmful additives in the material/product composition that are not recoverable after use	%
ed1_renewmat	Bio‐based and renewable materials	Amount of bio‐based and other renewable alternatives in the material composition	%
ed1_recymat	Use of recyclates	Amount of recyclates in the material composition to fulfill same function	%
ed1_recyblmat	Use of recyclable materials	Total substitution potential of the recyclates of the material/product after use (Dimensionless)	No unit

Step 3. Definition of indicators for each parameter

After defining the parameters across the ecodesign strategies, these parameters are quantified using indicators. All indicators associated with the parameters considered in this study are presented in Table S3 (SI1). For the fictitious example used to illustrate the methodological framework, only the first ecodesign strategy ‘Low‐impact materials’, together with its corresponding parameters and indicators is considered and is shown in Table 8.

Step 4. Quantification of indicator scores for each indicator

Once indicators are defined for each parameter, the corresponding indicator scores are determined for each indicator. Similar to the SDG Index score methodology, for a given product type, threshold values for each indicator score are defined based on available data and the associated targets for each parameter within an ecodesign strategy (e.g., increasing the use of recyclates in a product by 25%). These threshold values range from an upper limit, representing best performance, to a lower limit, representing worst performance. Between these two limits, green and red threshold band scores are defined to indicate whether a region, country or an organization is progressing toward achieving a given parameter within an ecodesign strategy.

For example, if the ecodesign strategy ‘Low‐impact materials’ is to be assessed and quantified for HDPE plastic packaging across different regions, the associated indicator scores are quantified accordingly (Table 9). For the parameter ‘ed1_Cleanmat’, the percentage of additives in the packaging is defined to range from 0% to 20%, where 0% represents the upper limit (no addition of additives in the packaging) and 20% represents the lower limit (20% of additives in the product composition). In this example, the green threshold is set at 5%, which regions, countries or sectors aim to achieve, while the red threshold is set at 10%, which regions, countries or sectors seek to avoid exceeding.

**TABLE 9:** Range, threshold and normalized scores for different parameters associated with the ecodesign strategy ‘Low‐impact materials’.

Parameter	Upper limit max (X)	Green	Red	Lower limit min (X)	Reference product (HDPE Packaging) X	Normalized score X_i_^’^
ed1_cleanmat	0	5	10	20	2	90
ed1_renewmat	100	70	20	0	15	15
ed1_recymat	25	10	5	0	10	40
ed1_recyblmat	1	0.8	0.5	0.1	0.6	56

This process of defining limits and corresponding scores should be applied to each indicator and can be informed by industry reports, expert estimation, scientific literature, and relevant databases.

Step 5. Normalization of indicator scores

After establishing the limits and thresholds for each indicator score, the values are normalized to make them comparable across different levels using the following formula:

$$X_{i}^{\prime }=\frac{X-\min\left(X\right)}{\max\left(X\right)-\min\left(X\right)}\;\times 100$$

where 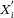 is the normalized (dimensionless) value for indicator i; *X* represents the original value of the indicator for the reference product in a given time period for a country, region or organization; max (*X*) and min (*X*) refer to the upper and lower threshold values defined for that indicator. In the case of ‘ed1_cleanmat’, these thresholds correspond to 0% (upper limit) and 20% (lower limit) as shown in Table 9.

In this illustrative example, the reference product (*X*) is assumed to have an indicator score of 2%, meaning that the reference product (HDPE Packaging in Region 1) contains 2% additives in its material composition. The normalized score for the parameter ‘ed1_cleanmat’, based on the reference and threshold indicator scores from Table 10, is therefore calculated as:

$$X_{i}^{\prime }\left(ed1_{cleanmat}\right)=\frac{2-20}{0-\;20}\times 100=90$$

**TABLE 10:** Ecodesign Index Score of the ecodesign strategy ‘Low‐impact materials’ for a reference product (packaging made of HDPE) produced across different regions and their progress for the year 2024.

Nation	ed1_cleanmat (normalized)	ed1_renewmat (normalized)	ed1_recymat (normalized)	ed1_recyblmat (normalized)	Individual Ecodesign score, ED_i_ (normalized)
Region 1	90	15	40	56	50.2 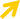
Region 2	80	20	65	30	48.7 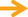
Region 3	92	50	78	70	72.5 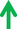
Region 4	60	10	35	45	37.5 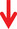

As the reference product is assumed to contain a relatively low proportion of additives, the resulting normalized score for this strategy is 90. This indicates that Region 1 is progressing toward achieving the ecodesign strategy of minimizing additive content in material composition, which can positively influence the recovery and recycling of products after use.

A representative example showing the threshold limits and corresponding normalized scores for different parameters of the ecodesign strategy ‘Low‐impact materials’ for the reference product (HDPE packaging in Region 1) is provided in Table 9. Descriptions of all parameters are available in Table S3 (SI1).

The upper and lower limits, as well as the threshold values for indicators associated with different parameters, should be derived from industrial associations, governmental agencies, scientific literature, or expert estimation. The normalized indicator scores can then be calculated for comparable products produced across different regions over a defined time period.

Step 6. Calculation of the individual ecodesign score

Once the normalized scores of all indicators within a given ecodesign strategy have been calculated, the individual ecodesign score (Table 10) is determined as the weighted average of the normalized indicator scores for that strategy using the following formula,


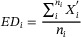

where, *ED_i_* represents the individual ecodesign score for an ecodesign strategy and *n_i_* denotes the total number of indicators associated with that ecodesign strategy.

For example, the individual ecodesign score for the strategy ‘Low‐impact materials’ for HDPE packaging manufactured in Region 1 in the year 2024, based on the normalized scores presented in Table 9, is calculated as:

$$ED_{i}\;\left(\mathrm{Low}-\mathrm{impact}\;\mathrm{materials}\right)=\frac{90+15+40+56}{4}=50.2$$

Step 7. Plastic Ecodesign Index Score

**FIGURE 5:**
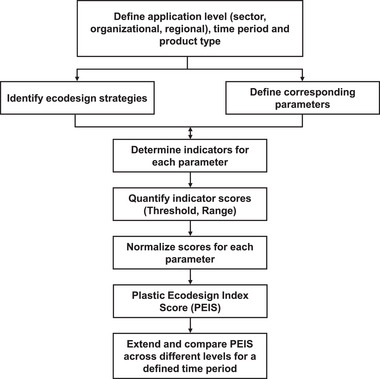
Steps to calculate Product Ecodesign Index Score (PEIS) of a product, based on the SDG Index Score Methodology.

The overall Plastic Ecodesign Index Score (PEIS) for a specific product in a given region over a defined time period is calculated as the weighted average of the individual ecodesign scores. In this study, the PEIS represents the weighted average of the ecodesign scores across the eight identified ecodesign strategies and is calculated as:


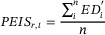

where *PEIS*_*r*,*t*_ denotes the Plastic Ecodesign Index Score for a particular product in region ‘r’ over time period ‘t’, and *n* is the total number of ecodesign strategies identified and assessed for that product. The methodological steps involved in calculating the PEIS for plastic products are illustrated in Figure 5.

Finally, the normalized individual ecodesign scores for a given product in a particular region can be compared with scores from previous years, to evaluate whether performance is decreasing 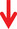, stagnating 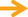, moderately improving 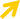, or on track 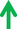 toward achieving the upper limit of the respective ecodesign strategy, as shown in Table 10.

Based on the individual ecodesign scores presented in Table 10, it is possible to assess how a region or sector needs to progress or has progressed in achieving a given ecodesign strategy for a product or sector. In the illustrative example, the representative ecodesign scores for the strategy ‘Low‐impact materials’ indicate that Region 3 has progressed and is on track to achieve the maximum score (100 points) for this strategy in the production of HDPE packaging. This outcome is attributed to the assumption that products manufactured in Region 3 contain lower amounts of harmful additives, a higher share of recyclates in production and a better substitution potential, reflecting the use of higher quality of recyclates capable of replacing virgin HDPE.

In contrast, for Region 4, the scores and observed trends suggest the need to increase the share of renewable content and recyclates in the production of HDPE packaging in the coming years. For Regions 1 and 2, although reductions in additive content have been achieved (as indicated by the normalized scores for ‘ed1_cleanmat’ in Table 10), further improvements are required in increasing the share of renewable materials (‘ed1_renewemat’ in Table 10) and enhancing product recyclability (‘ed1_recycblmat’ in Table 10).

Based on these normalized and individual ecodesign scores, goals and targets can be established within the sector to support the design of products that are recoverable after use and to improve the resource efficiency of the plastic value chain over time. This methodological framework can also be applied at the organizational level and to specific sets of plastic products.

## 5 Limitations

To assess the impact of plastics across the value chain on different aspects of sustainable development and to examine how the scientific literature addresses plastic‐related ecodesign strategies, a comprehensive literature review was conducted. Based on a defined set of screening criteria, approximately 380 studies were considered in this review. These studies were subsequently subjected to a qualitative assessment, through which plastic‐specific ecodesign strategies were identified, along with the development of the PEIS framework to quantify them.

However, the literature review, qualitative assessment and implementation of the PEIS framework to quantify ecodesign strategies are subject to certain limitations, which are discussed in this section.

### 5.1 Literature Review

Publications that were inaccessible to the author team were excluded from the review. Examples of inaccessible publications include unavailable book chapters, conference proceedings available only as abstracts, and journal articles not accessible due to subscription limitations of the authors’ institutions. The exclusion of such publications may influence the results of the qualitative assessment, as these studies could address relevant aspects of the role of plastics in sustainable development or discuss specific design strategies aimed at improving the resource efficiency of plastic across the value chain. Consequently, this limitation may affect both the scoring of impact of plastics on sustainable development and the total contribution of studies allocated to different SDGs toward specific ecodesign strategies.

Moreover, Scopus was selected as the sole search engine for the literature review due to its built‐in framework for mapping publications to the SDGs. As a result, publications not indexed in the Scopus database were not considered in this study. This may have led to the exclusion of relevant studies on plastics and sustainable development that are indexed in other databases or published in regional or non‐indexed journals.

### 5.2 Qualitative Assessment

Both the screening and scoring criteria developed by the authors of this study were applied to a limited set of search results that combined the terms ‘plastics’ and ‘polymers’ with keywords derived from the targets and indicators of the 17 SDGs. As a result, the selected literature may not provide a comprehensive representation of all benefits and adverse impacts of plastic products across their entire lifecycle.

The selection of studies was guided by different aspects of sustainable development and therefore primarily addressed pressing global societal challenges such as inequality, sanitation, pollution and health. Consequently, the literature considered in this review tends to emphasize the adverse impacts associated with the use and disposal of plastics.

Moreover, with the exception of some studies allocated to SDGs 7, 8, 9 and 10 that examined economic impacts, most studies focused predominantly on the environmental and social dimensions of plastic use. Nevertheless, there is no ‘one‐size‐fits‐all’ approach to evaluating the benefits and adverse impacts of plastics from a sustainability perspective. In the context of ecodesign strategies, however, assessing critical impacts rather than beneficial impacts of products currently in use is particularly important, as these aspects can be taken into consideration when designing plastic products in the future.

The scoring criteria developed to evaluate the impacts of plastics and their relationship with different ecodesign strategies were inherently subjective, as they were defined and applied by the author team based on their expertise and experience. Incorporating quantitative approaches alongside the qualitative assessment could enable a more robust understanding of the interactions between plastics and different dimensions of sustainable development.

For example, if a study focused exclusively on the impacts of additives and harmful chemicals used in the production of plastic products, it was assessed as having a ‘Strong’ relationship with the ecodesign strategy ‘Low‐impact materials’ during the qualitative assessment. In contrast, the same study was assumed to have a weaker relationship with other ecodesign strategies like ‘Optimization of distribution’ or ‘Optimization of lifetime’, if these aspects were not addressed. This limitation also reflects a broader trend in the literature and, to some extent, in industry practice, where less attention is given to the distribution and use stages of products due to limited data availability on product applications and consumer behavior.

### 5.3 Implementation of PEIS

The definition and comparability of upper and lower threshold limits for different indicators of ecodesign strategies within the proposed PEIS framework require co‐operation and harmonization across the plastic sector at regional and international level. As the identification of ecodesign strategies is not yet standardized, achieving acceptance of the defined parameters and the use of indicator scores across different ecodesign strategies among stakeholders may be time‐consuming.

Regular verification and the development of new ecodesign strategies, together with their associated parameters and indicator scores, require scientific and societal consensus. The ecodesign strategies identified for the plastic sector in this study are based on a qualitative assessment of the scientific literature addressing plastics and different aspects of sustainable development. However, future identification of ecodesign strategies should be informed by both qualitative and quantitative assessment, which would support a more holistic understanding of the plastic value chain among the stakeholders involved.

The implementation of ecodesign strategies, once identified, should be consistent across the value chain. This includes planning logistics for the collection and recovery of plastic waste, developing new business models to recover products and extend the lifetime of plastic products within the system, and, most importantly, aligning stakeholders with objectives related to resource recovery.

Based on the identified ecodesign strategies, changes in product design may require adjustments to production settings, including processing methods and resource consumption, as well as the identification of new material alternatives. This may involve establishing new supplier relationships, developing new value chains, and addressing considerations related to materials availability and cost while ensuring that product functionality is maintained.

In addition to implementing these strategies, appropriate regulatory frameworks must be established globally to ensure adequate regional recycling infrastructure for the recovery of plastic waste after use. It is also essential to consider the region‐specific economic viability and social equity when identifying and implementing ecodesign strategies. This is because a given set of ecodesign strategies may not be equally suitable across regions due to differences in cultural practices, access to controlled waste disposal, and levels of awareness among organizations and consumers regarding waste handling.

Ultimately, even when appropriate ecodesign strategies are in place, the successful recovery of plastic products depends on the availability of waste streams and supporting infrastructure, as well as the price and quality of recyclates.

## 6 Conclusion and Outlook

A qualitative assessment of 380 studies mapped to the 17 Sustainable Development Goals (SDGs) was conducted to evaluate the lifecycle impacts of plastics across environmental, economic and social dimensions. Furthermore, the relationship between the selected studies and ecodesign strategies was examined, using the Life Cycle Design Strategy (LiDS) Wheel as the conceptual basis. The scoring criteria used to assess impacts of plastics and relationships to ecodesign strategies were developed by the authors of this study.

The qualitative assessment revealed that studies associated with SDGs 1–6 predominantly highlighted adverse impacts on human health and the environment, particularly those related to additives and chemicals used in polymer production and use. Literature mapped to SDGs 8–15 emphasized the consequences of inadequate recovery and disposal of plastic waste, including the leakage of macro‐, micro‐ and nanoplastics across terrestrial and aquatic ecosystems. Studies allocated to SDGs 16 and 17 focused on governance‐related challenges, notably the intra‐ and inter‐continental transportation of plastic waste to regions with insufficient recycling infrastructure and the lack of harmonized regulatory frameworks. In contrast, studies allocated to SDG 7 reported beneficial impacts of using plastic waste as an energy source; however, incineration or pyrolysis of plastic waste results only in energy recovery rather than material recovery and therefore remains misaligned with circular economy principles [437].

The assessment of relationships between the studies and ecodesign strategies revealed a strong focus on end‐of‐life solutions, low‐impact materials and new concept development, reflecting the dominant emphasis on waste management and regulatory responses in the literature. Conversely, weak to no relationships were observed for strategies related to material reduction, optimization of production and distribution, reduction of impacts during use, and extension of product lifetime. This indicates that the literature considered in this study has largely underrepresented design‐oriented strategies such as product lifetime, utility, impacts during production, repair, and (re‐)use, despite their potential to significantly influence the recoverability of plastic products after use.

Building on these findings, the study identified a preliminary set of plastic‐specific ecodesign strategies and associated parameters. While this study relied on qualitative assessment, combining qualitative and quantitative approaches would enable a more comprehensive understanding of interactions across the plastic value chain. The scoring criteria for assessing the impacts of plastics and identifying plastic‐specific ecodesign strategies could be further refined by incorporating parameters such as economic viability [438], social equity [439], availability of regional recycling infrastructure [440], policy frameworks [213, 441] and policy recommendations [442].

To enable structured comparison and quantification of ecodesign strategies across products, sector, regions and time periods, the Plastic Ecodesign Index Score (PEIS) was proposed as a methodological framework based on the SDG Index score methodology. The PEIS framework translates ecodesign strategies into measurable indicators and supports the assessment of product performance at organizational, sectoral, regional, national, and international levels. For successful implementation of PEIS across these levels, harmonization of product‐specific parameters, indicators, and threshold values across stakeholders is essential.

Overall, the findings underscore the importance of complementing waste management solutions with design‐oriented strategies that influence resource efficiency and recoverability throughout the plastic value chain. Identifying ecodesign strategies early in the product development phase, and quantifying their implementation using a framework such as PEIS can support stakeholders in designing plastic products that remain within the system after use, thereby enhancing resource efficiency and contributing to sustainable development.

## Conflicts of Interest

The authors declare no conflict of interest.

## Supporting information




**Supporting File 1**: gch270099‐sup‐0001‐SuppMat.docx.

**Supporting File 2**: gch270099‐sup‐0002‐SuppMat.pdf.

## Data Availability

The authors have nothing to report.
