# Supplementary material for: Plastics and Sustainable Development – Identifying and Quantifying Ecodesign Strategies for Plastics
Source: Glob Chall. 2026 Mar 23;10(3):e00033. doi: 10.1002/gch2.202500033 (PMC13093836; doi:10.1002/gch2.202500033)
Supplement: Supplementary file 1 — Supporting File 1: gch270099‐sup‐0001‐SuppMat.docx. [file GCH2-10-e00033-s001.docx]

Supporting Information 1 (SI1)

Plastics and Sustainable Development - A Framework to Identify and Quantify Ecodesign Strategies for Plastics - A Literature-based Analysis

Venkateshwaran Venkatachalam ^*^, Sebastian Spierling, Mikołaj Owsianiak, Frederik R. Wurm, Leonie Barner and Hans-Josef Endres

^*^ Correspondence: venkatachalam@ikk.uni-hannover.de, Tel.: +49-511-762-13328

**A0. Ecodesign strategies in the plastic sector**

Table S 1 Practical examples of ecodesign strategies implemented by organizations in the plastic sector

| Ecodesign strategy | Industry | Solution |
| --- | --- | --- |
| New material alternatives | Kuori | Development of bio-based, biodegradable and recyclable Thermoplastic Elastomer (TPE) made of olive pits and nuts, used in outdoor sport products^[1]^ |
| Process optimization | Engel | Two-stage plastic processing technology to produce plastic parts directly from shredded plastic waste thereby reducing energy consumption upto 30%^[2]^ |
| Waste recovery in production | HolyPoly | Aquaplus re paint box made from 99.4% recycled plastic^[3]^ |
| New business models for recyclates | cirplus | Digital procurement platform for recycled plastics introducing additional quality specifications and certifications to suit the requirements of different sectors^[4]^ |
| Eliminating hazardous chemicals in the products | Pact Group | Plasma coating technology for containers that removes the need for fluorination, which causes PFAS (Per- and Polyfluoroalkyl substances)^[5]^ |

**A1. Keyword Occurrence Diagrams for Plastics and SDGs**

**
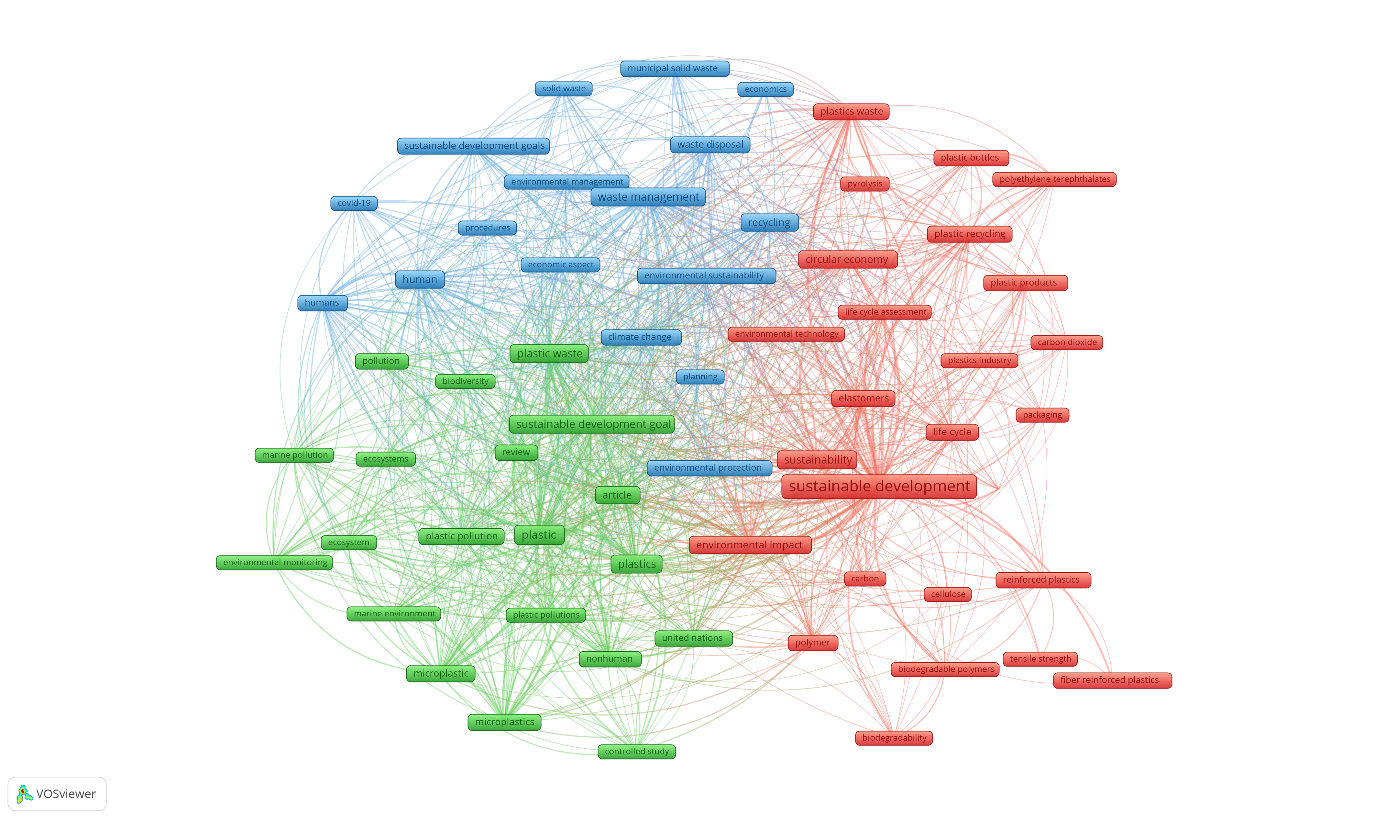
**

**Figure S 1** Keyword occurrences for studies belonging to 'Plastic' and 'Sustainable Development' (Created with VOSViewer^[6]^)

**
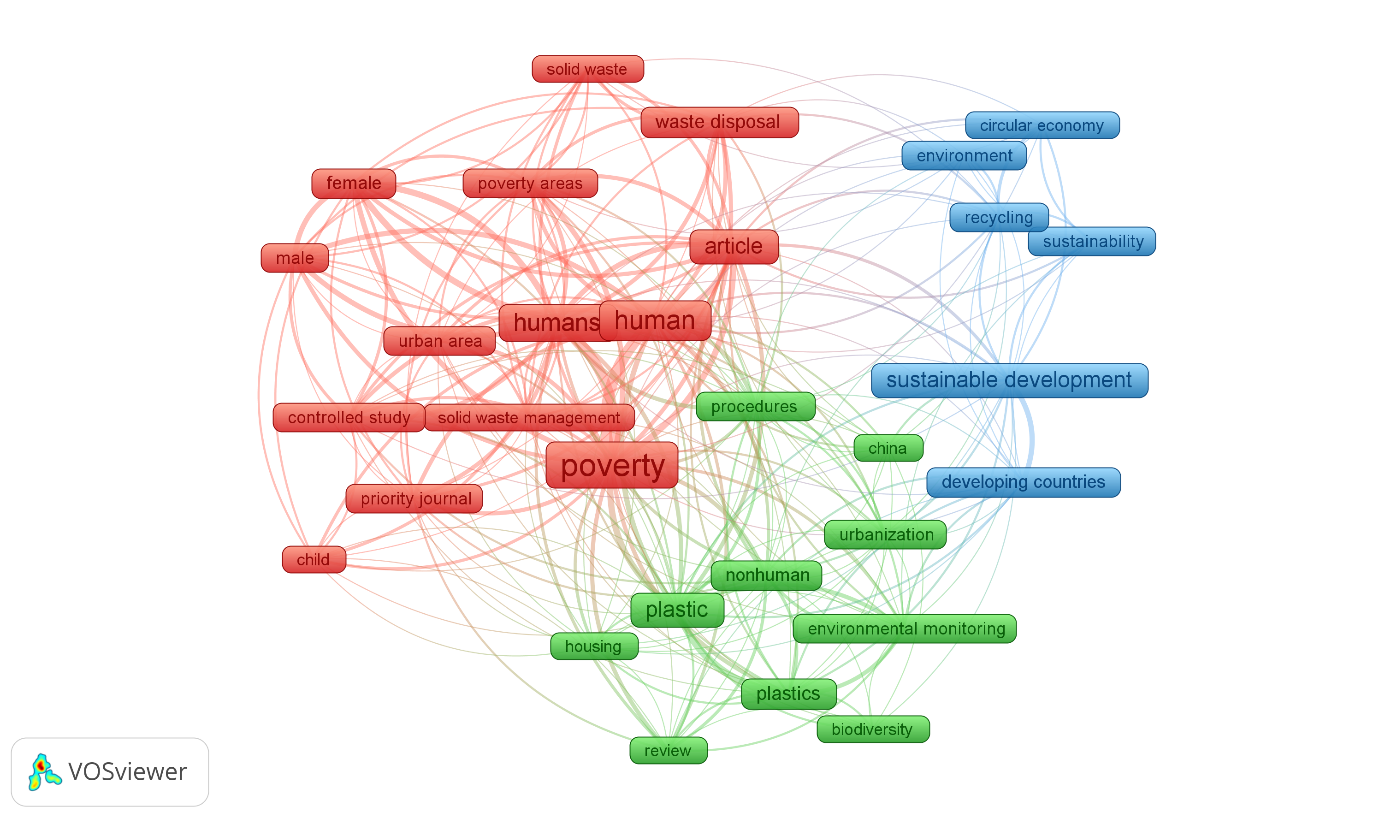
**

**Figure S 2** Keyword occurrences for studies belonging to SDG1 (Created with VOSViewer^[6]^)


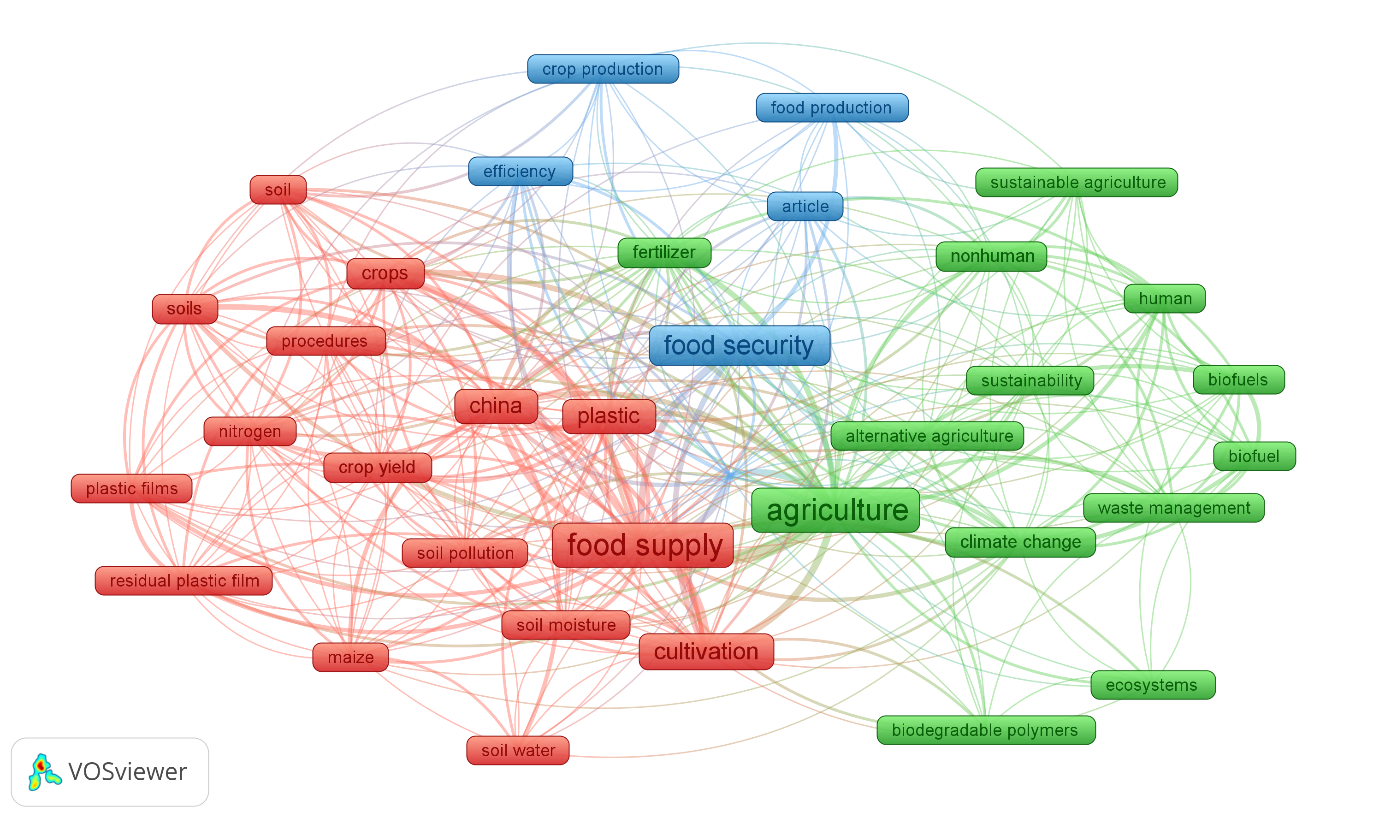


**Figure S 3** Keyword occurrences for studies belonging to SDG2 (Created with VOSViewer^[6]^)


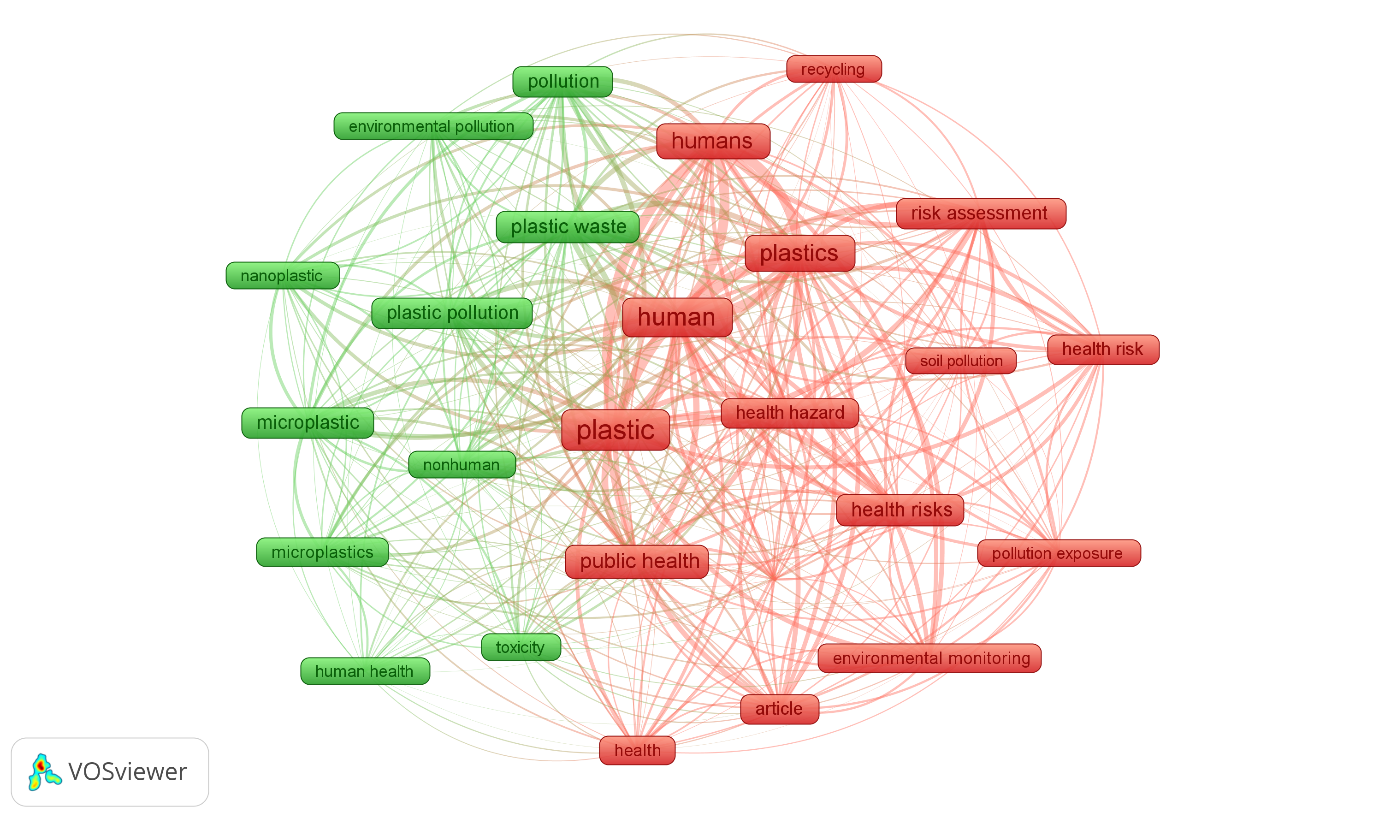


**Figure S 4** Keyword occurrences for studies belonging to SDG3 (Created with VOSViewer^[6]^)


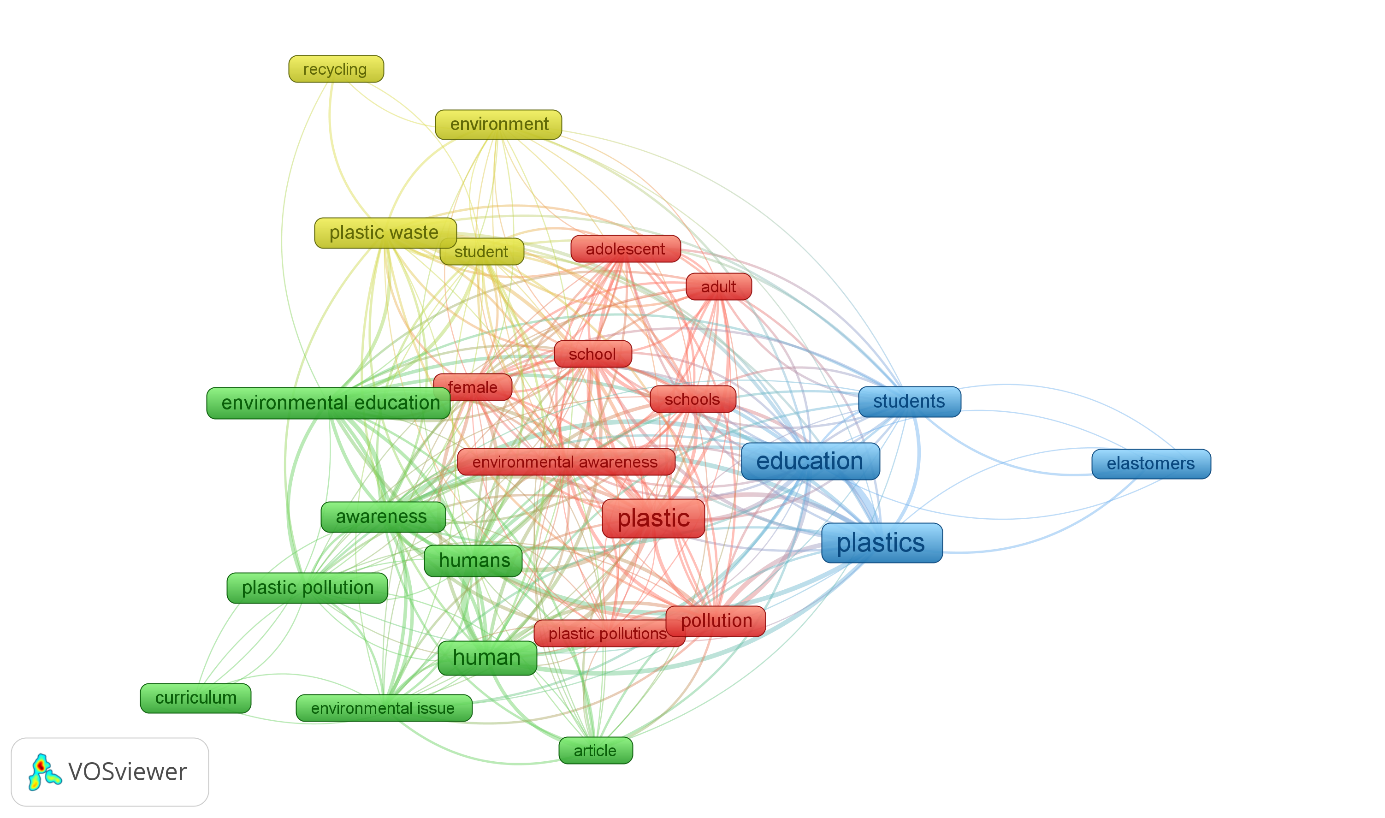


**Figure S 5** Keyword occurrences for studies belonging to SDG4 (Created with VOSViewer^[6]^)


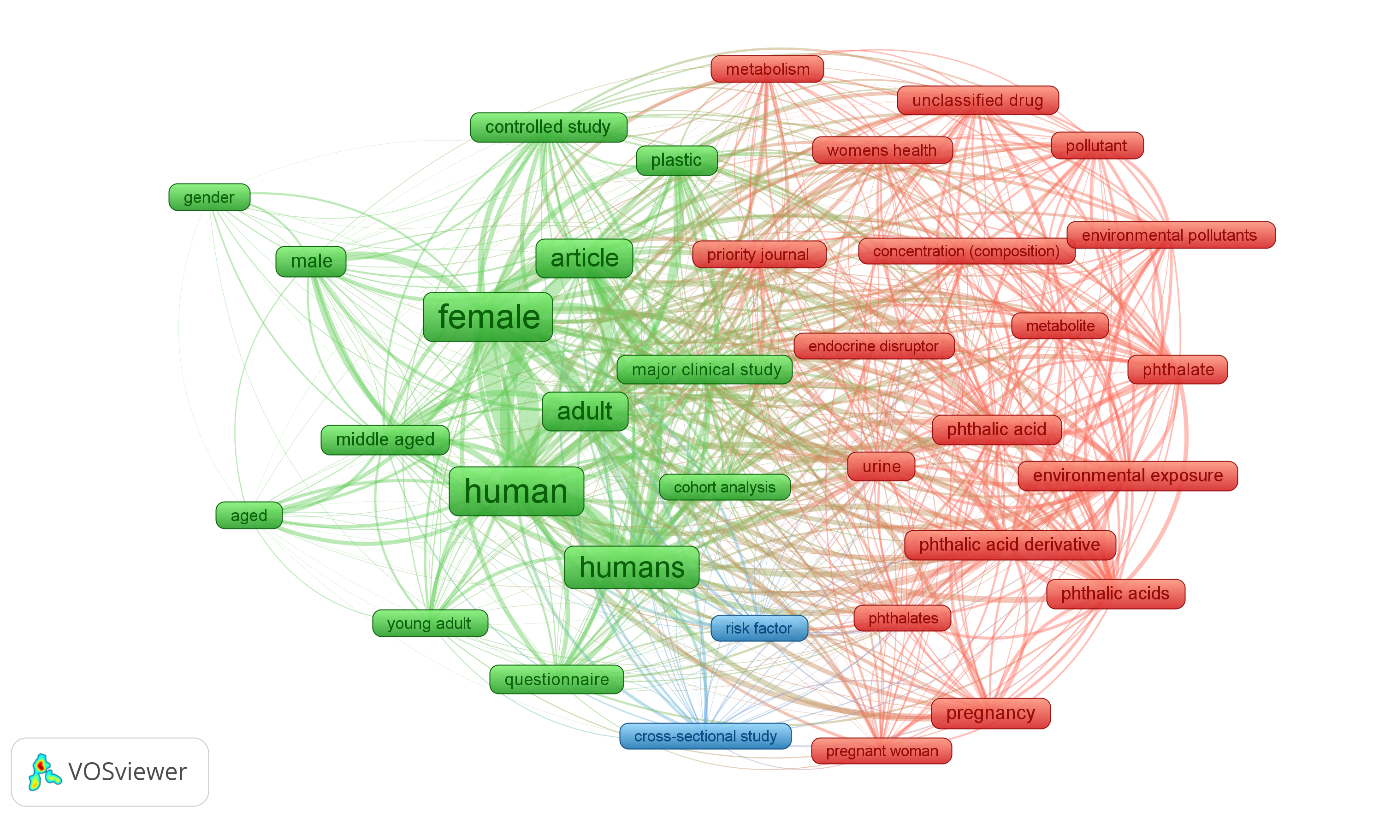


**Figure S 6** Keyword occurrences for studies belonging to SDG5 (Created with VOSViewer^[6]^)


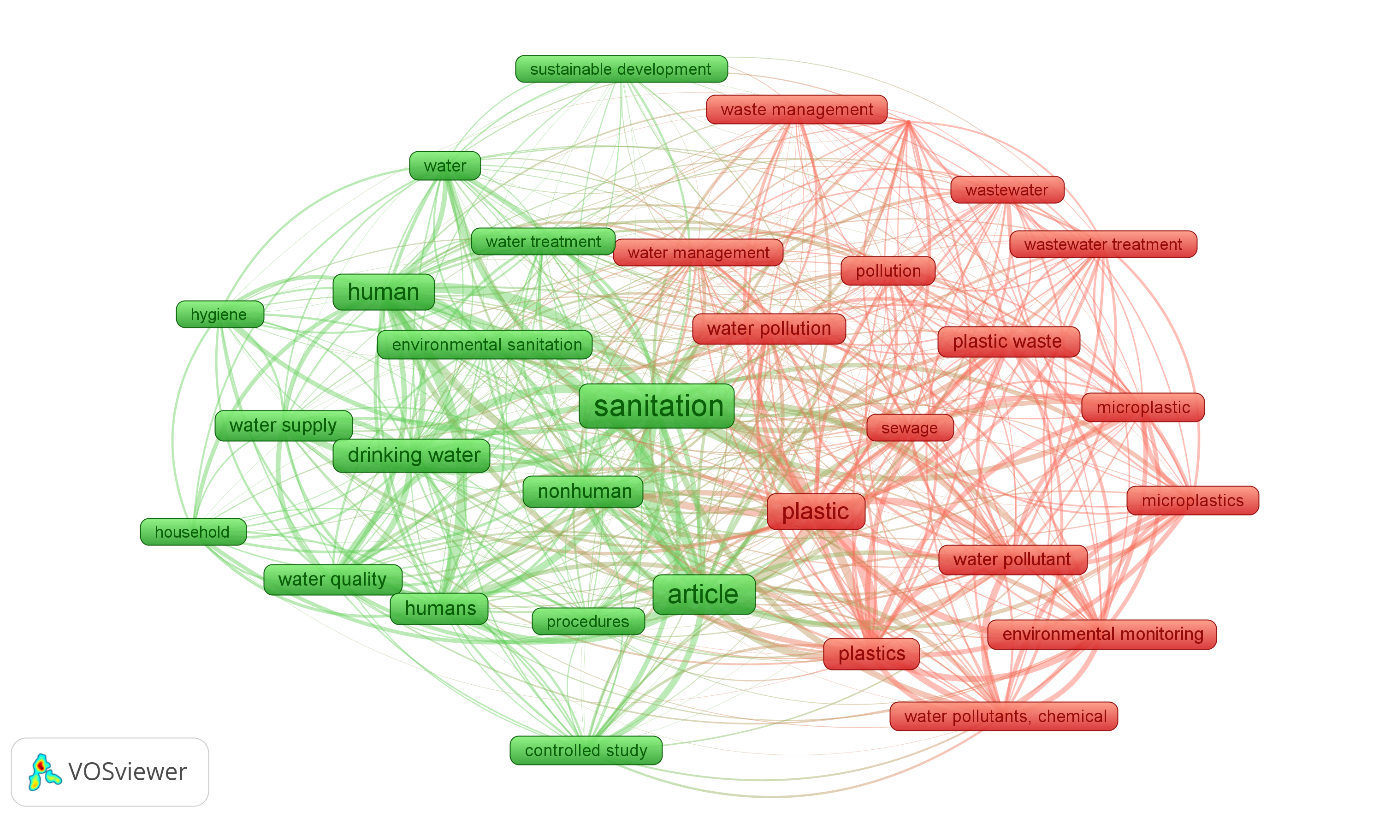


**Figure S 7** Keyword occurrences for studies belonging to SDG6 (Created with VOSViewer^[6]^)


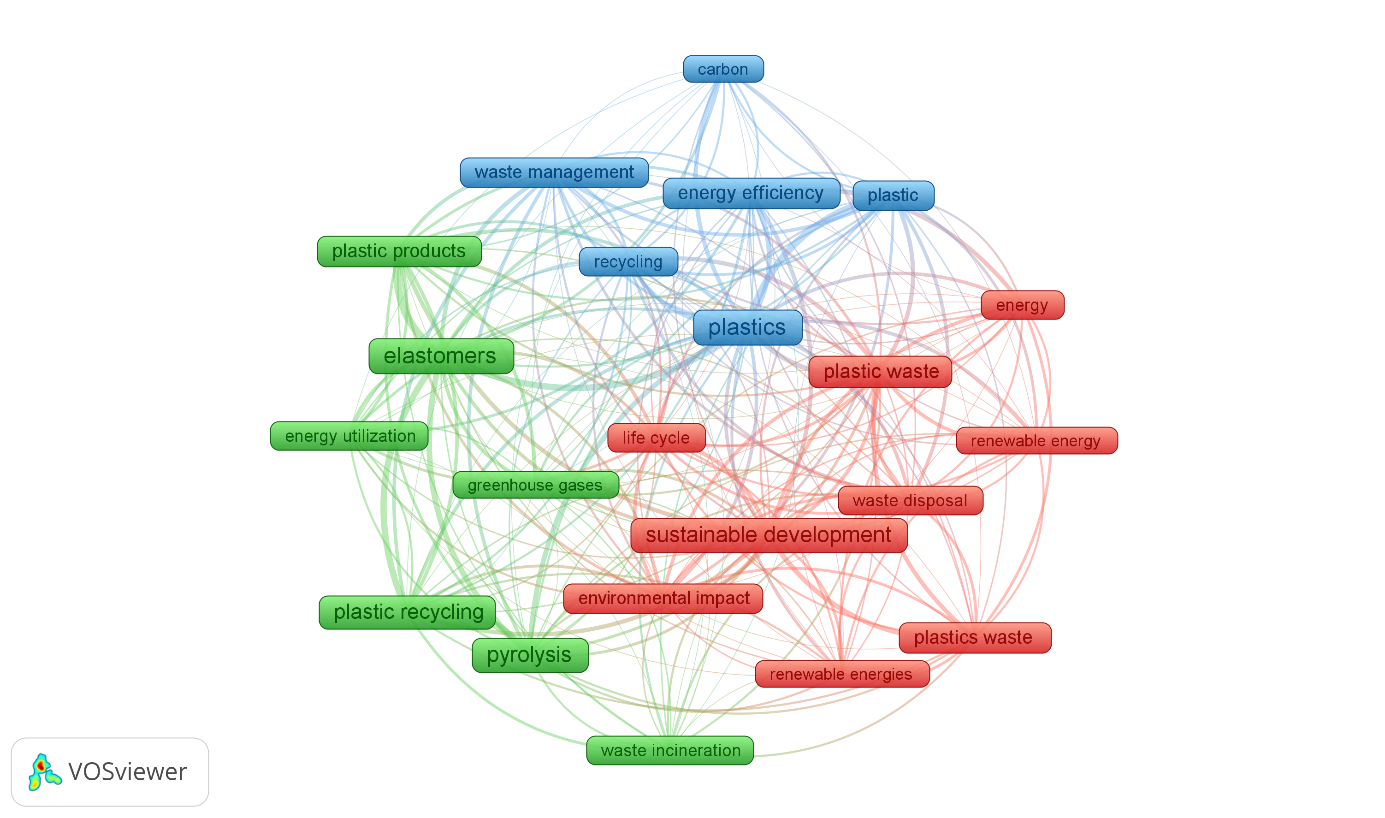


**Figure S 8** Keyword occurrences for studies belonging to SDG7 (Created with VOSViewer^[6]^)


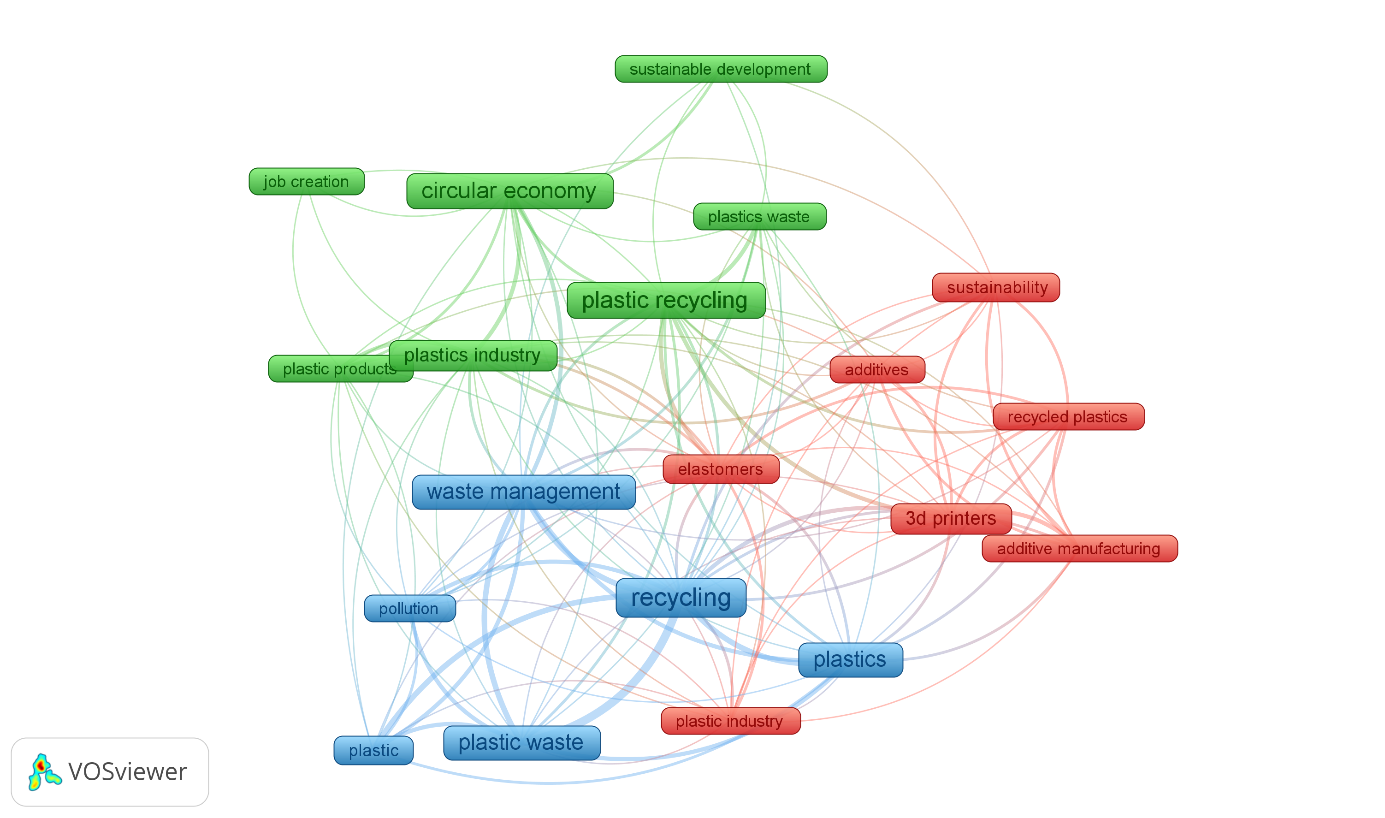


**Figure S 9** Keyword occurrences for studies belonging to SDG8 (Created with VOSViewer^[6]^)


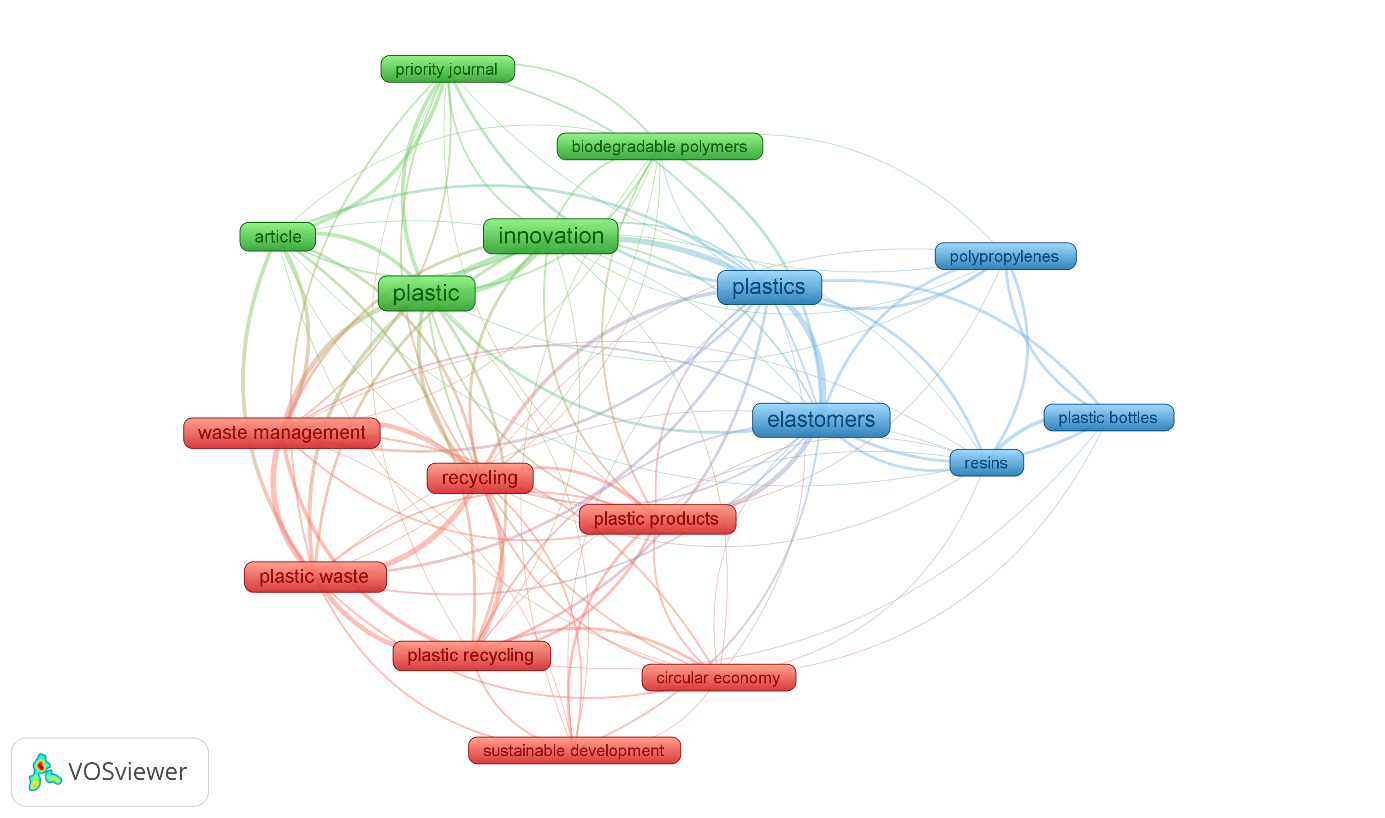


**Figure S 10** Keyword occurrences for studies belonging to SDG9 (Created with VOSViewer^[6]^)


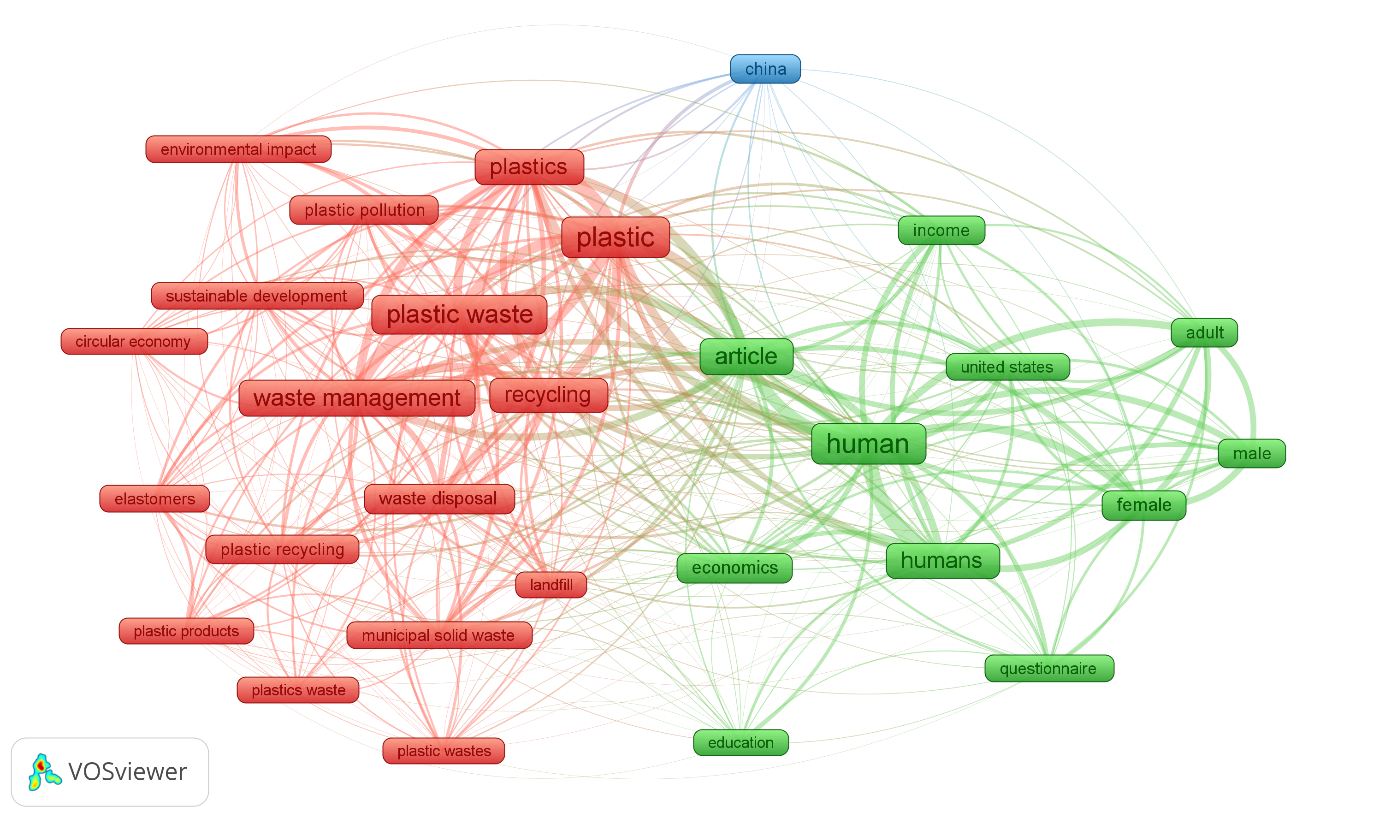


**Figure S 11** Keyword occurrences for studies belonging to SDG10 (Created with VOSViewer^[6]^)


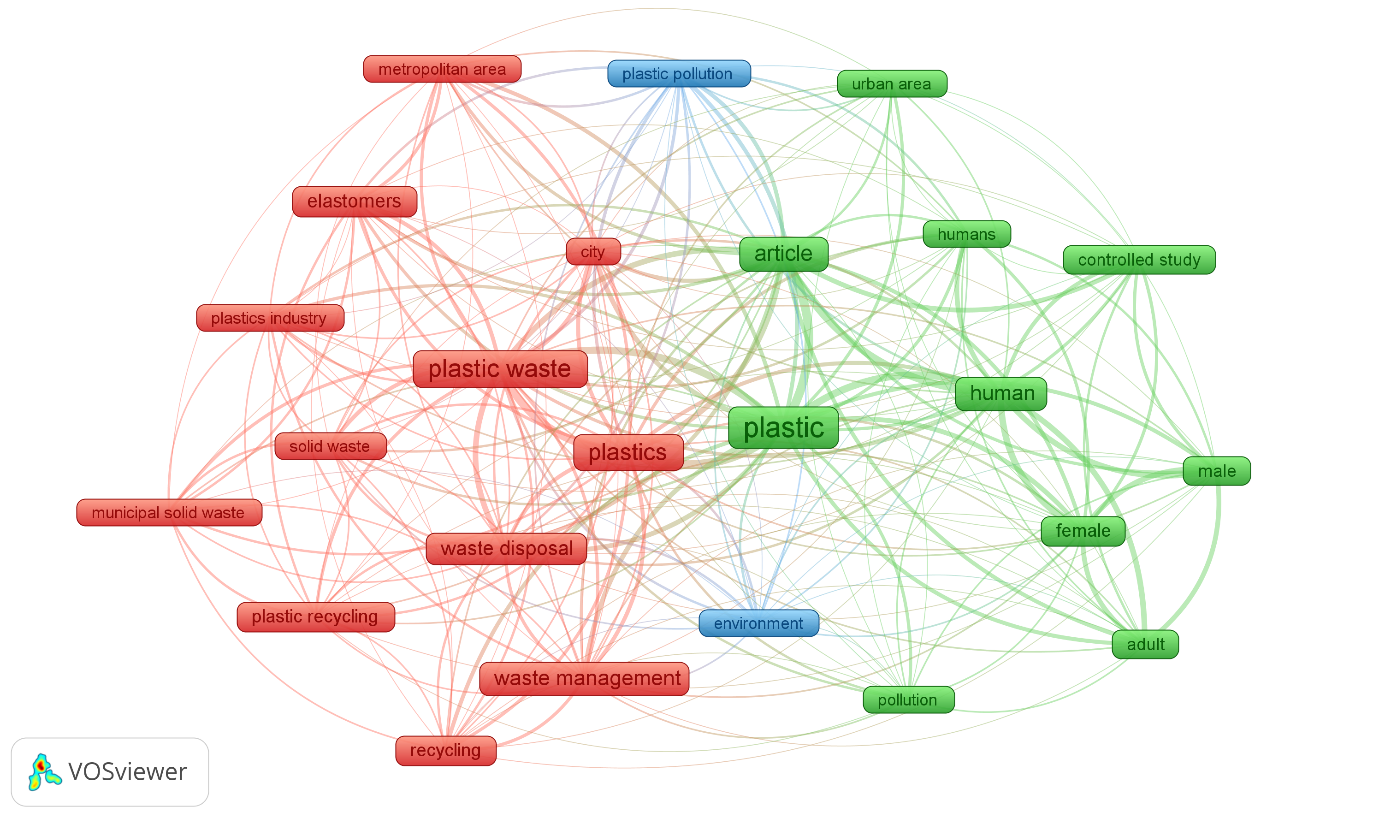


**Figure S 12** Keyword occurrences for studies belonging to SDG11 (Created with VOSViewer^[6]^)


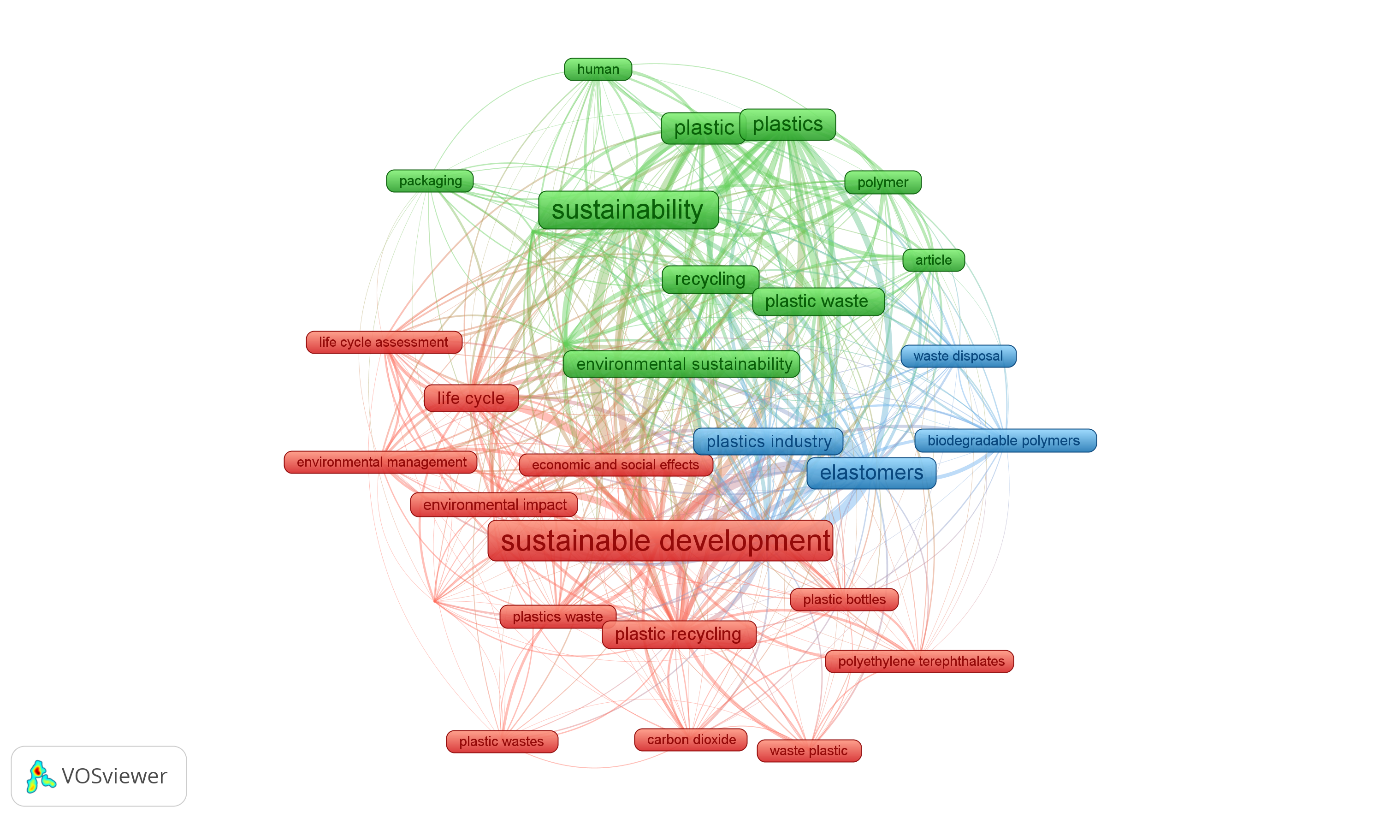


**Figure S 13** Keyword occurrences for studies belonging to SDG12 (Created with VOSViewer^[6]^)


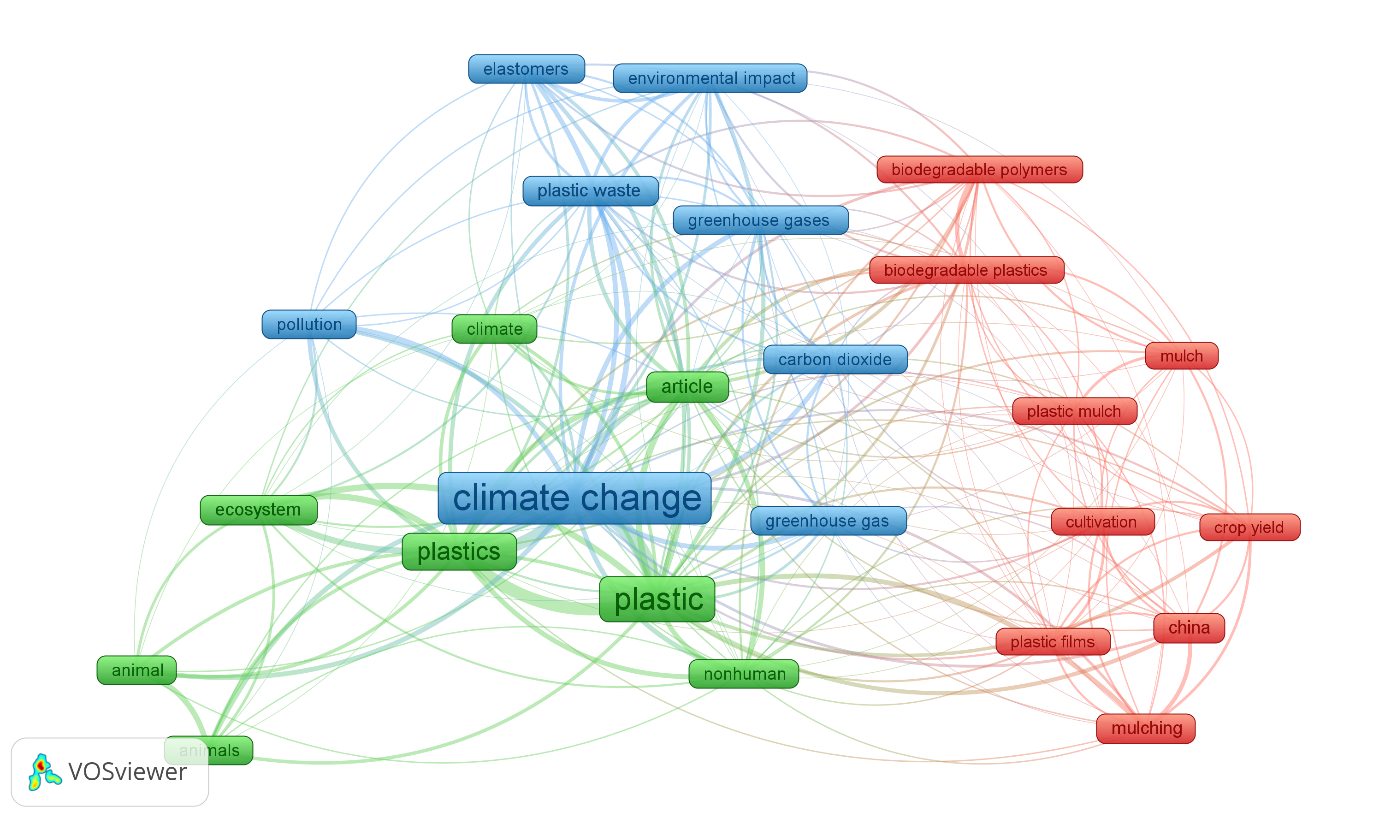


**Figure S 14** Keyword occurrences for studies belonging to SDG13 (Created with VOSViewer^[6]^)


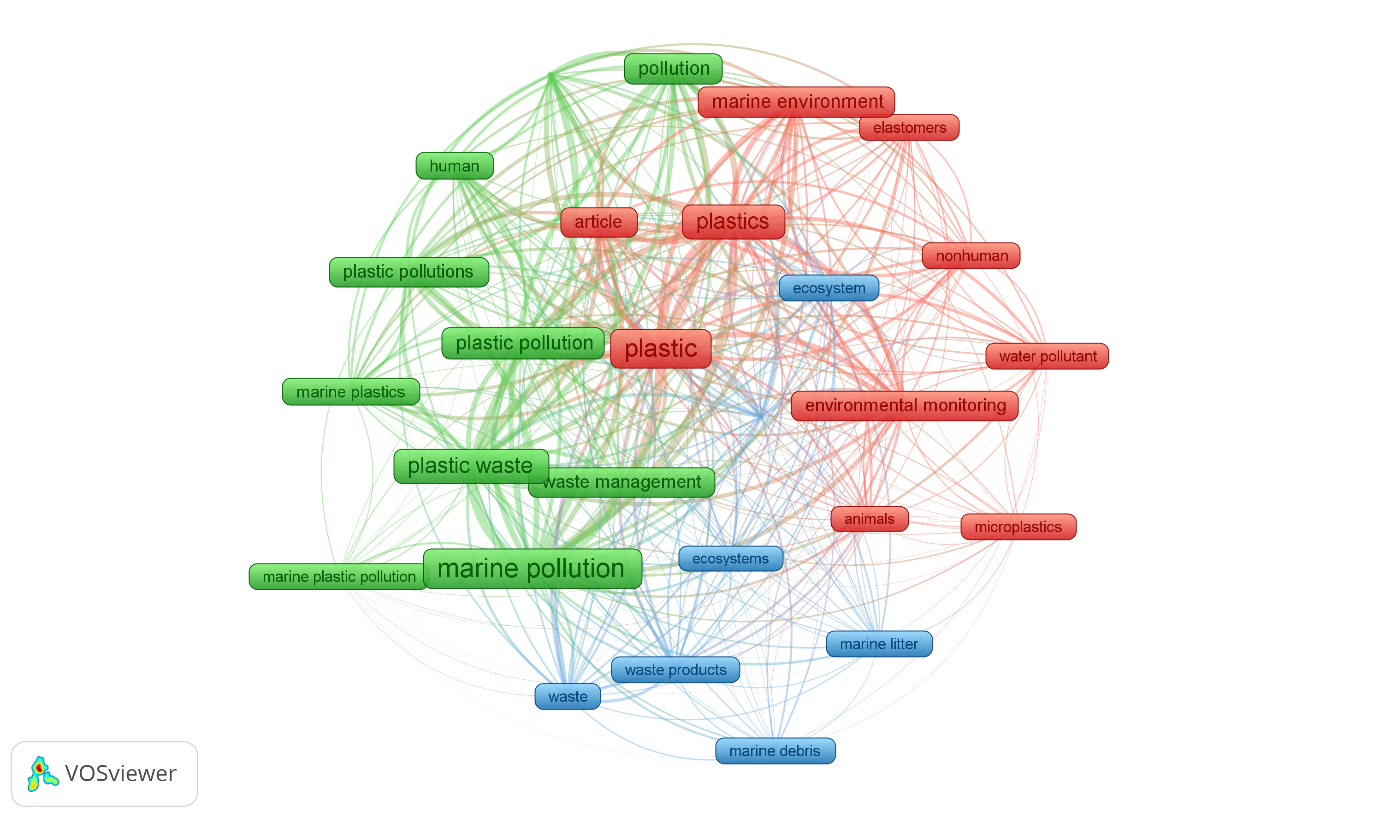


**Figure S 15** Keyword occurrences for studies belonging to SDG14 (Created with VOSViewer^[6]^)


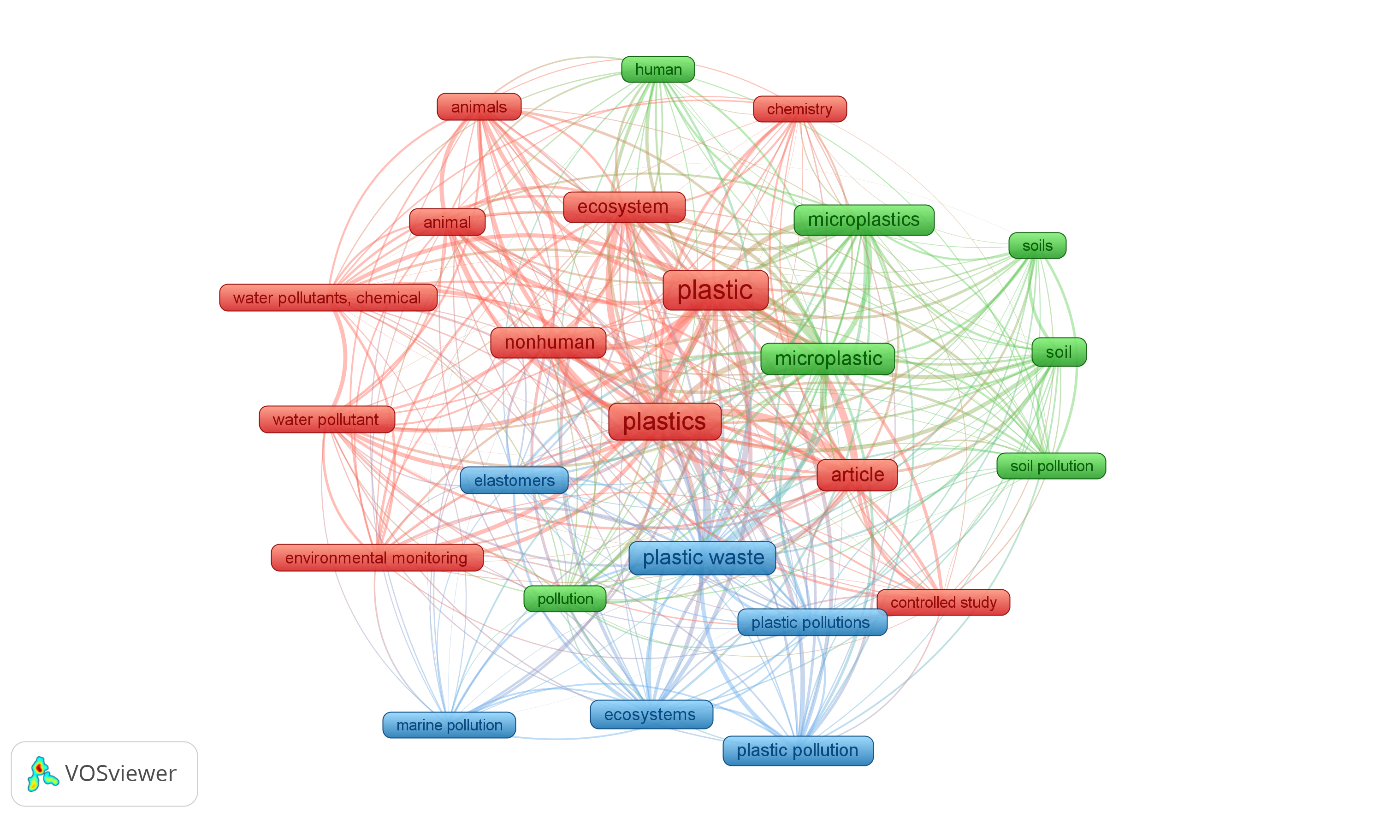


**Figure S 16** Keyword occurrences for studies belonging to SDG15 (Created with VOSViewer^[6]^)


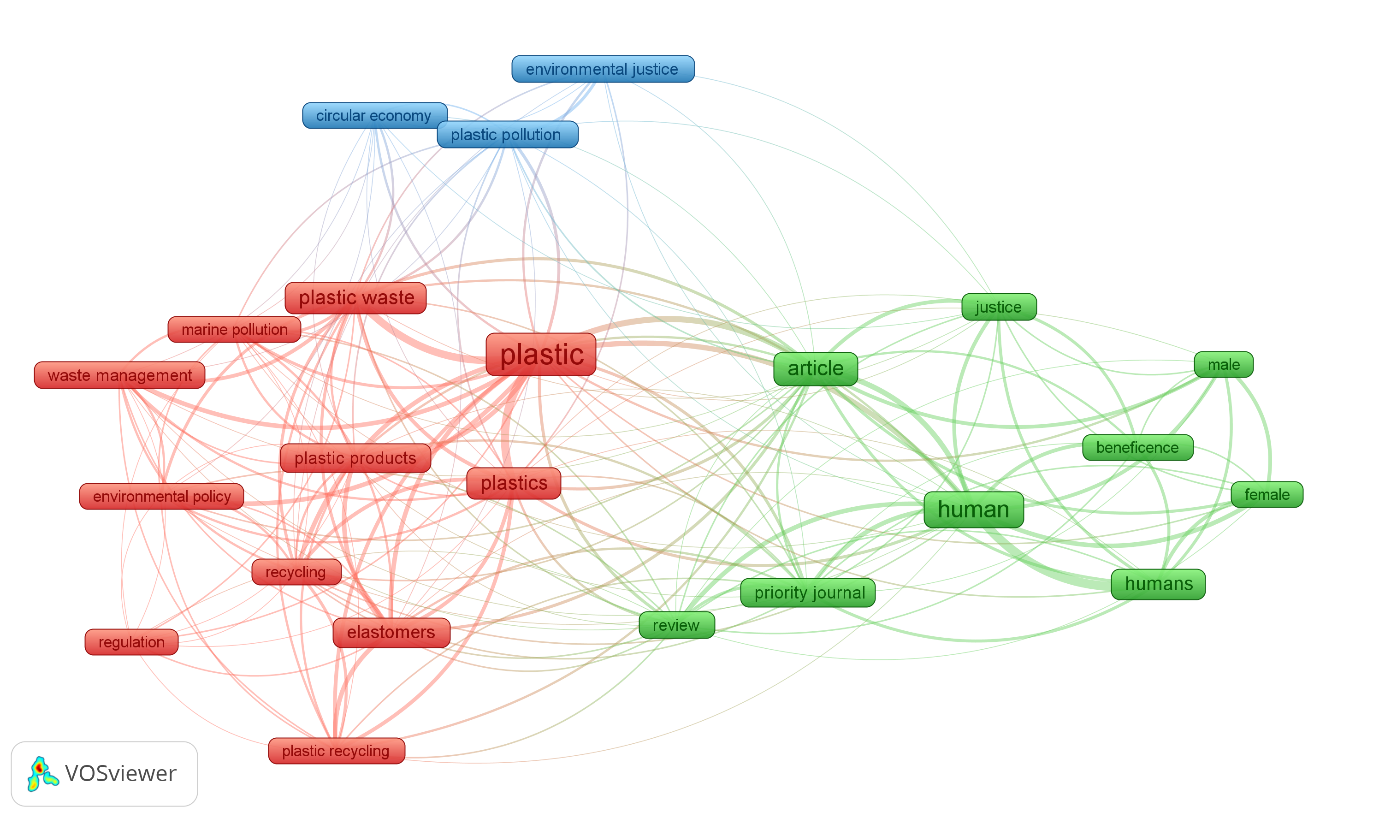


**Figure S 17** Keyword occurrences for studies belonging to SDG16 (Created with VOSViewer^[6]^)


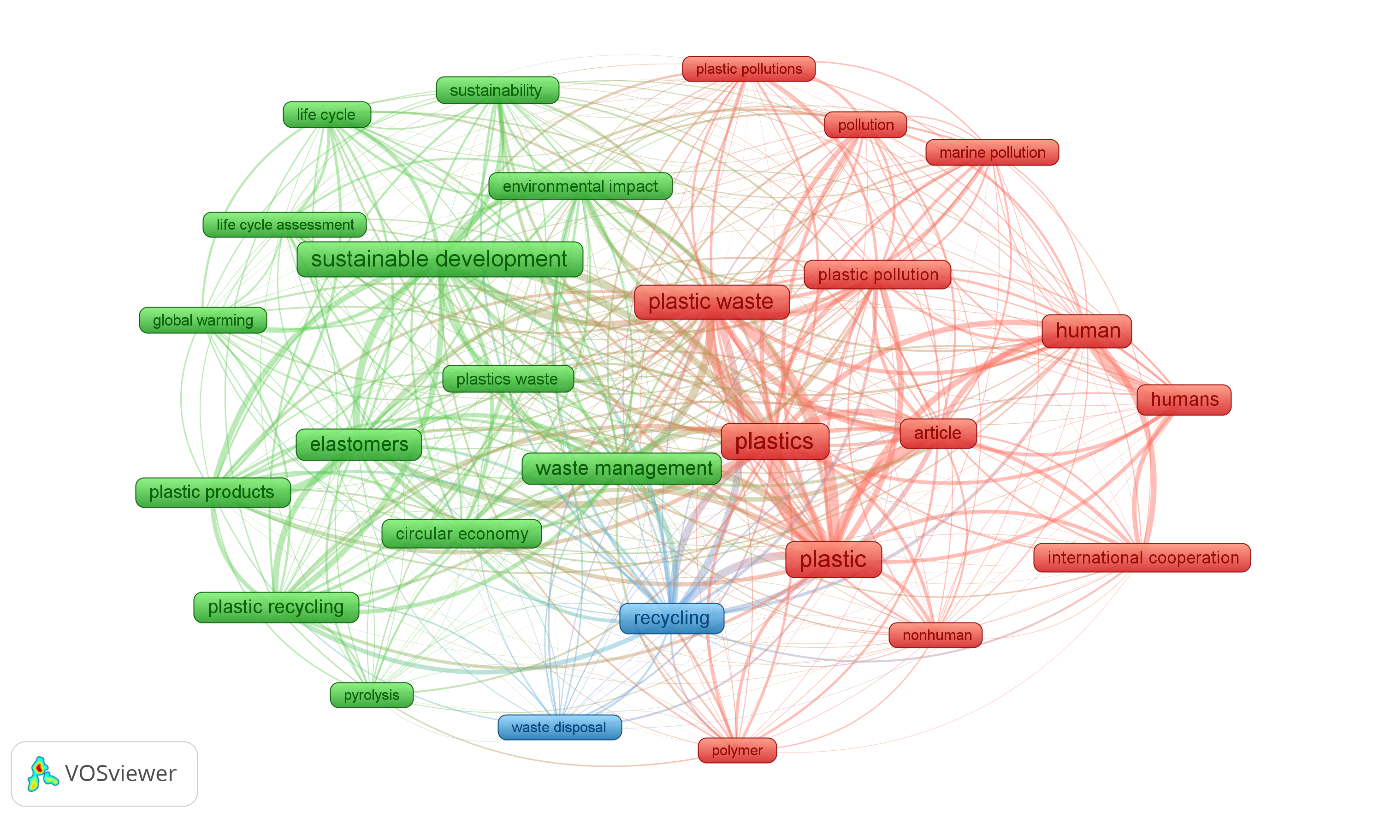


**Figure S 18** Keyword occurrences for studies belonging to SDG17 (Created with VOSViewer^[6]^)

**A2. Interactions of the studies with other SDGs**

After the screening analysis, these studies, were allocated to the respective SDG. For example, search results for the combination of keywords ‘plastics’ and ‘polymers’ with terms such as ‘poverty’, ‘people’ and ‘population’ were screened based on the criteria and are then allocated to SDG1. Then, for each study, their contribution towards other SDGs, based on the Scopus mapping framework^[7]^, was determined. The main objective of determining these interactions is to understand how the scientific literature about plastics discusses different aspects of SD. An example of the Scopus SDG mapping framework for the scientific literature is shown in Figure S19.


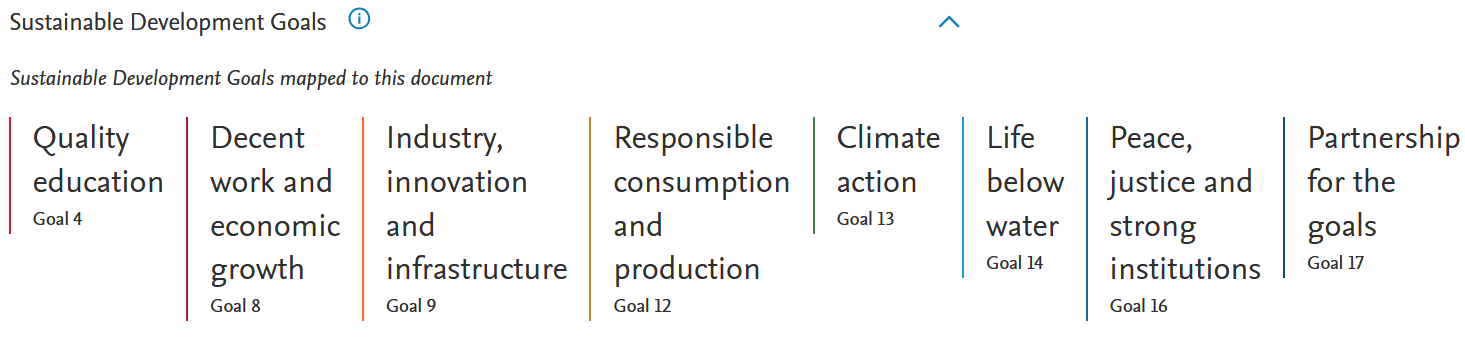


**Figure S 19** Scopus mapping of publication towards SDGs for Kumar et al.^[8]^

From the above figure, it can be seen that for a publication like Kumar et al.^[8]^, which discusses the relationship of plastics towards all the 17 SDGs, the publication’s SDG contribution was mapped only towards SDGs 4, 8, 9, 12, 13, 14, 16, and 17 by Scopus. Although Scopus has constantly improved the mapping approach of the studies towards SDGs over the last two years, a qualitative assessment was done for all the studies considered in this review to map them to the other possible SDGs, apart from the ones Scopus has already mapped.

Some of the assumptions in assessing the interactions between different SDGs for the studies are as follows:

- Studies from a particular SDG are not mapped to the same SDG. i.e., if a study from SDG1 is qualitatively assessed and even if the study is found to focus on the different aspects of SDG1 (through Scopus and own assessment), they were not mapped to SDG1 (as the aim of this methodology is to assess how the studies allocated to a particular SDG interacts with other SDGs)
- Qualitative mapping of the studies to other SDGs involves analyzing the keywords from the SDG indicator and target framework^[9]^ and comparing these aspects with the focus and discussion of each study.
- Apart from the mapping done by Scopus and through qualitative assessment, the total interactions of each study allocated to a SDG, to other SDGs were calculated by adding the interactions from Scopus and own assessment together

For example, Navarre et al.^[10]^ was allocated to the SDG 10 during the literature review, as it was one of the many results for the keywords ‘plastics’, ‘polymers’, along with the keywords, based on the targets/indicators for SDG 10 like ‘income’, ‘injustice’, ‘inequality’ and ‘social’. Along with the title, abstract and keywords of the study, the SDGs mapped to this study based on Scopus framework was also analyzed. And the SDGs mapped to this study, according to Scopus (as of 30th May 2024) were SDG 12 (Responsible Consumption and Production) and SDG 14 (Life below Water) as the study talks about how Dutch plastic food packaging waste gets leaked to the marine environment due to the export of plastic waste to nations in Asia, despite their high recycling rate for post-consumer plastic packaging^[10]^.

When analyzing the whole publication, it was found that this study also discusses about aspects that can contribute to SDGs, other than 12 and 14. These are: 1) how plastic packaging can prevent food loss and the associated anthropogenic greenhouse gas emissions (SDG 13 - Climate Action), along with the increase in food security (SDG 2 - Zero Hunger); 2) limited capacity of recycling facilities in different nations to handle large volumes of waste and waste ending up in landfills (SDG 9 - Industry, Innovation and Infrastructure and SDG 11 - Sustainable Cities and Communities); 3) leakage and effects of plastic wastes from land to the marine environment (SDG 15 - Life above Land); 4) propose for stringent regulatory policies and frameworks in the export of plastic wastes to the nations in Asia (SDG 16 - Peace, Justice and Strong Institutions)

The different interactions of the considered studies to other SDGs were then calculated and visualized in the form of chord diagrams, which are shown in Figures S 20 - S 37. Based on the SDG mapping framework of Scopus and our own qualitative assessment of every literature, considered for this study, the interactions of the studies towards other SDGs were determined. The contribution of each study towards other SDGs was then summed up to get the total interaction between a study allocated to a particular SDG towards other SDGs. They were then visualized in the form of chord diagrams to understand the relationship between the literature allocated to a particular SDG and other SDGs. The total interactions between the studies from each SDG towards other SDGs are shown in Figure S20 below.

**Figure S 20** Chord diagrams showing interactions between SDGs based on the mapping framework of Scopus (Top left), qualitative assessment of literature in this study (Top right) and Total interactions (Bottom) (Created with Flourish^[11]^)

In Figure S20, the top left chord diagram represents the mapping of the literature allocated to each SDG towards other SDGs, based on the Scopus framework and the top right refers to the interactions that were assessed by the author team (mapping of literature towards SDGs, apart from the ones already mapped using Scopus). The bottom chord diagram represents the total interactions. Individual chord diagrams of each SDG towards other SDGs are shown below from Figures S21-S37.

**Figure S 21** Interaction of the chosen studies in SDG 1 with other SDGs (Scopus + Own assessment = Total interactions) (Created with Flourish^[11]^)

**Figure S 22** Interaction of the chosen studies in SDG 2 with other SDGs (Scopus + Own assessment = Total interactions) (Created with Flourish^[11]^)

**Figure S 23** Interaction of the chosen studies in SDG 3 with other SDGs (Scopus + Own assessment = Total interactions) (Created with Flourish^[11]^)

**Figure S 24** Interaction of the chosen studies in SDG 4 with other SDGs (Scopus + Own assessment = Total interactions) (Created with Flourish^[11]^)

**Figure S 25** Interaction of the chosen studies in SDG 5 with other SDGs (Scopus + Own assessment = Total interactions) (Created with Flourish^[11]^)

**Figure S 26** Interaction of the chosen studies in SDG 6 with other SDGs (Scopus + Own assessment = Total interactions) (Created with Flourish^[11]^)

**Figure S 27** Interaction of the chosen studies in SDG 7 with other SDGs (Scopus + Own assessment = Total interactions) (Created with Flourish^[11]^)

**Figure S 28** Interaction of the chosen studies in SDG 8 with other SDGs (Scopus + Own assessment = Total interactions) (Created with Flourish^[11]^)

**Figure S 29** Interaction of the chosen studies in SDG 9 with other SDGs (Scopus + Own assessment = Total interactions) (Created with Flourish^[11]^)

**Figure S 30** Interaction of the chosen studies in SDG 10 with other SDGs (Scopus + Own assessment = Total interactions) (Created with Flourish^[11]^)

**Figure S 31** Interaction of the chosen studies in SDG 11 with other SDGs (Scopus + Own assessment = Total interactions) (Created with Flourish^[11]^)

**Figure S 32** Interaction of the chosen studies in SDG 12 with other SDGs (Scopus + Own assessment = Total interactions) (Created with Flourish^[11]^)

**Figure S 33** Interaction of the chosen studies in SDG 13 with other SDGs (Scopus + Own assessment = Total interactions) (Created with Flourish^[11]^)

**Figure S 34** Interaction of the chosen studies in SDG 14 with other SDGs (Scopus + Own assessment = Total interactions) (Created with Flourish^[11]^)

**Figure S 35** Interaction of the chosen studies in SDG 15 with other SDGs (Scopus + Own assessment = Total interactions) (Created with Flourish^[11]^)

**Figure S 36** Interaction of the chosen studies in SDG 16 with other SDGs (Scopus + Own assessment = Total interactions) (Created with Flourish^[11]^)

**Figure S 37** Interaction of the chosen studies in SDG 17 with other SDGs (Scopus + Own assessment = Total interactions) (Created with Flourish^[11]^)

Some of the significant observations based on these chord diagrams for each and every SDG are discussed in Table S1 below.

**Table S 2** Observations from the interactions between SDGs based on Scopus and Authors’ Assessment

| Goals | Interactions based on Scopus | Interactions based on own assessment |
| --- | --- | --- |
| SDG 1 | - Major contribution towards SDG 17 (Partnership for the goals) as SDG 17 have queries like “poverty”, “poor countr”, “low-income nation” included in them, which are closely related to SDG1 - As some of the studies focus on the state, infrastructure and economic implications of plastic waste management and recycling in different cities, there were significant interactions with SDG 8, SDG 9, SDG 11 and SDG 12 - Interactions can also be seen towards SDG 10 and SDG 14 as some of the studies focus on the plight of marginalized communities living along the coasts combating ocean plastic pollution | - As some of the studies discussed about awareness of waste management and educating people on the use of plastic bags, they were mapped to SDG 4 (Education) - Few studies also discussed the inequalities among people in the use and disposal of plastic products and they were mapped to SDG 9 (Infrastructure) and SDG 10 (Reduced inequalities) |
| SDG 2 | - As most of the studies report the use and economics of plastic mulching films and their greenhouse gas emissions in the low-income and middle-income countries, most of the documents were mapped to SDGs 8, 12, 13, 17 - Few of the studies focus on the agricultural yield using plastic mulch films and were mapped to SDG 2 (No hunger) - Some studies also reported the transport of mulches in the ground and surface water after use and were mapped to SDG 6 (Clean water and sanitation) | - Effects of plastic mulch on the soils and also the impacts of using mulch films on the land were discussed in several studies and therefore were mapped to SDG 15 (Life on land) - The presence of microplastics after the use of mulch films and their effects on oceans and marine ecosystems were discussed in some studies and therefore mapped to SDG 14 (Life below water) |
| SDG 3 | - As most of the studies had ‘pollution’, ‘packaging’, ‘environment’, ‘plastic waste’, ‘cities’ in their titles, keywords and abstracts, they were mapped primarily to SDG 11, 12, 14 - There were some studies mapped to SDG 17, as some of the studies focused on the international frameworks and partnerships with other countries to reduce plastic wastes and to improve the recycling infrastructure | - However, there were additional studies that could be mapped to SDG 12 as they discussed the sustainable production and consumption of plastics products - Moreover, some studies focus on the climate change effects (SDG 13) due to plastic pollution (open dump burning) and the lack of infrastructure (SDG 9) along with the inequalities (SDG 10) and pollution in the water bodies (SDG 14) and terrestrial ecosystems (SDG 15) they bring within the cities and communities (SDG 11) - Few studies were also mapped to SDG 1 (Poverty being one of the reasons for the open burning and inadequate collection infrastructure) and entry of plastics into the food chain (SDG 2) |
| SDG 4 | - As most of the studies dealt with the disposal and recycling of plastic wastes in educational institutions, they were mapped to SDG 12 - Some of the studies also focused on ocean plastics and economic growth in plastic industry and they were mapped to SDG 14 and SDG 8 | - After assessment, some studies were found to focus on the cooperation, plastic pollution on land, infrastructure and plastic waste management in cities and climate impacts. Thus, they were mapped to SDG 17, 15, 9,11 and 13 respectively |
| SDG 5 | - Most of the studies allocated to this SDG focus on the consumer behavior (based on gender) of (re)using plastic bags in different cities and empowerment of marginalized communities against plastic pollution and were therefore mapped to SDG 9, 10, 11, 12, 17 | - Some of the studies, apart from focusing on the recycling and infrastructure for handling plastic wastes, also focused on the abject poverty (SDG 1) and economic growth (SDG 8) in those areas, where plastic bags were implemented along with the lack of awareness (SDG 4), poor sanitation (SDG 6) and pollution in the fresh water ecosystems and environment (SDG 15) |
| SDG 6 | - Most of the studies were mapped to SDG 17 as they focus on the institutions that provide drinking water and bring policy frameworks to combat plastic pollution in the cities of developing countries (SDG 9, 11, 12) | - On further assessment, the studies also discussed the health impacts (SDG 3), living conditions (SDG 1) along with the working conditions (SDG 8) in mitigating the plastic pollution in coastal communities (SDG 10 and 14) |
| SDG 7 | - Almost all of the studies under SDG 7 focused on the potential of using plastic wastes as fuel and on the chemical recycling of plastic wastes. Therefore, they were mapped frequently to sustainable consumption and production of plastics (SDG 12), decarbonization and climate change (SDG 13) and their infrastructure (SDG 9) | - As some of the studies focused on the economic impacts and factors in designing waste to energy plants for plastics, they were mapped to SDG 8 and additionally to SDG 9 - Some of the studies explained the state of waste treatment in different cities and were therefore mapped to SDG 10 and 11 |
| SDG 8 | - Major contribution towards SDG 12 and SDG 17 as both the studies and accompanying keywords deal with circular economy policies and new models for job creation in plastics globally - As some of them also focused on the infrastructure and innovation to support circular economy, they were mapped to SDG 9 | - The circular economy framework for different countries also focused on the lack of awareness (SDG 4) with waste separation and collection along with the inequalities (SDG 10) with regards to the waste handling facilities (SDG 9) in the rural and urban regions across different countries (SDG 11) |
| SDG 9 | - Most of the studies were mapped primarily to SDG 14 and SDG 17 as the studies focused more on community-based recycling facilities across cities to counteract plastic pollution in the oceans and land - No mapping was found to SDG 8 and SDG 11, 12, 13, 15 although the studies reported recovery and pollution of plastic wastes along with their uncontrolled disposal | - On further assessment, some of the studies discussed the lack of economic infrastructure and incentives (SDG 8) to have circular economy in their frameworks that were not mapped - As these studies also reported the importance of recovering plastic wastes (SDG 12) across cities (SDG 11) thereby preventing them from leaking into the environment (SDG 15) and mitigating climate change (SDG 13) |
| SDG 10 | - Mapped mostly to SDG 11, 12, and 17 as the article titles and the associated keywords considered the export of plastic wastes and their recovery, along with the global development in combating plastic recovery | - However, within the studies, there were instances of studies highlighting the inequitable distribution and lack of awareness of plastic wastes (SDG 1 and 4) along with the emphasis on the lack of infrastructure (SDG 9) and marine pollution (SDG 14 and 15) and their climate effects (SDG 13) |
| SDG 11 | - As some of the studies assessed the recovery infrastructure and disposal of plastic bags in cities, they were mapped to SDG 9, 12 apart from their impacts on marine life globally (SDG 14 and 17) | - However, they also described the health impacts, lack of education (SDG 3, 4) along with the lack of economic incentives to handle these wastes (SDG 8) across communities (SDG 10) which could exacerbate the climate change effects (SDG 13) |
| SDG 12 | - Most of the studies were mapped towards the recovery and handling of plastics wastes (SDG 9, 11) along with circular economy frameworks (SDG 8, 17) that can have a large impact on climate change and marine ecosystems (SDG 13, 14) | - The studies allocated to this SDG also discussed the effects of plastic pollution on the living conditions (SDG 1, 2, 15) and health of people (SDG 3) across marginalized communities (SDG 10) - Some of the studies were also mapped to energy consumption in the plastic processing (SDG 7) and the revenue from recycling (SDG 8) |
| SDG 13 | - Infrastructure for circular economy and decarbonation through plastic wastes and preventing plastic pollution were discussed in some of the studies, thereby were mapped to SDG 9, 12, 14, and 17 | - However, they also focused on resources like energy (SDG7) and investments (SDG 8) that are needed for a working recycling infrastructure in cities (SDG 11 and 15) - Also focused on the governance and frameworks to curb the use of plastic bags (SDG 16 and 17) |
| SDG 14 | - The effects of marine plastic pollution were mapped towards the presence of microplastics in humans (SDG 3) along with the technical and regulatory measures to recover them (SDG 11, 12, 17) - Interestingly, not many direct interactions to SDG 15 were found, despite the fact that that macro-, micro- and nanoplastics travel across different mediums including soils and other terrestrial ecosystems | - On further assessment, the studies discussed a wide array of issues with the presence of micro and nanoplastics in the environment (SDG 15), starting from toxicity (SDG 3), sanitation (SDG 6), climate impacts (SDG 13) to the governance (SDG 16) and inequitable distribution and impacts of wastes (SDG 10) |
| SDG 15 | - Majority of the studies were mapped to SDG 14 due to the major focus on micro and nanoplastics in the environment. - No studies were mapped towards the societal impacts of plastic pollution in the terrestrial ecosystems despite them being the focus in many studies | - The societal impacts of microplastic pollution were identified through assessment and the corresponding studies were mapped to SDG 2, 3, 6, 8, 9, - Lack of recovery facilities in different cities meant those studies were mapped to SDG 11 and 12 |
| SDG 16 | - Studies that mainly analyze the regulatory and legal frameworks for circular economy (SDG 12 and 17) and preventing plastics polluting the marine environment (SDG 14) - The lack of infrastructure and the inequality with regards to the waste handling capacities and their impacts on lands were not mapped | - The economic implication of investing resources for complying with regulatory frameworks were identified and were then mapped to SDG 8, 9, 10, 11 - The impacts of uncontrolled disposal discussed in those studies along with their impacts on soils and fragile ecosystem were then identified to contribute to SDG 15 |
| SDG 17 | - Studies that focus on the academy-industry partnerships that can help increase the recovery of plastic wastes and prevent them from polluting the oceans (SDG 12 and 14) | - As these studies also handled the various economic and technological impacts by increasing the partnership between different stakeholders, those studies were mapped to SDG 8, 9, 10, and 11 - Furthermore, the recovery of plastics and avoidance of plastic pollution as a mitigating strategy for climate change meant that some of these studies were also mapped to SDG13 and 15 |

**A3. Relationship of the chosen studies from each SDG towards ecodesign strategies**


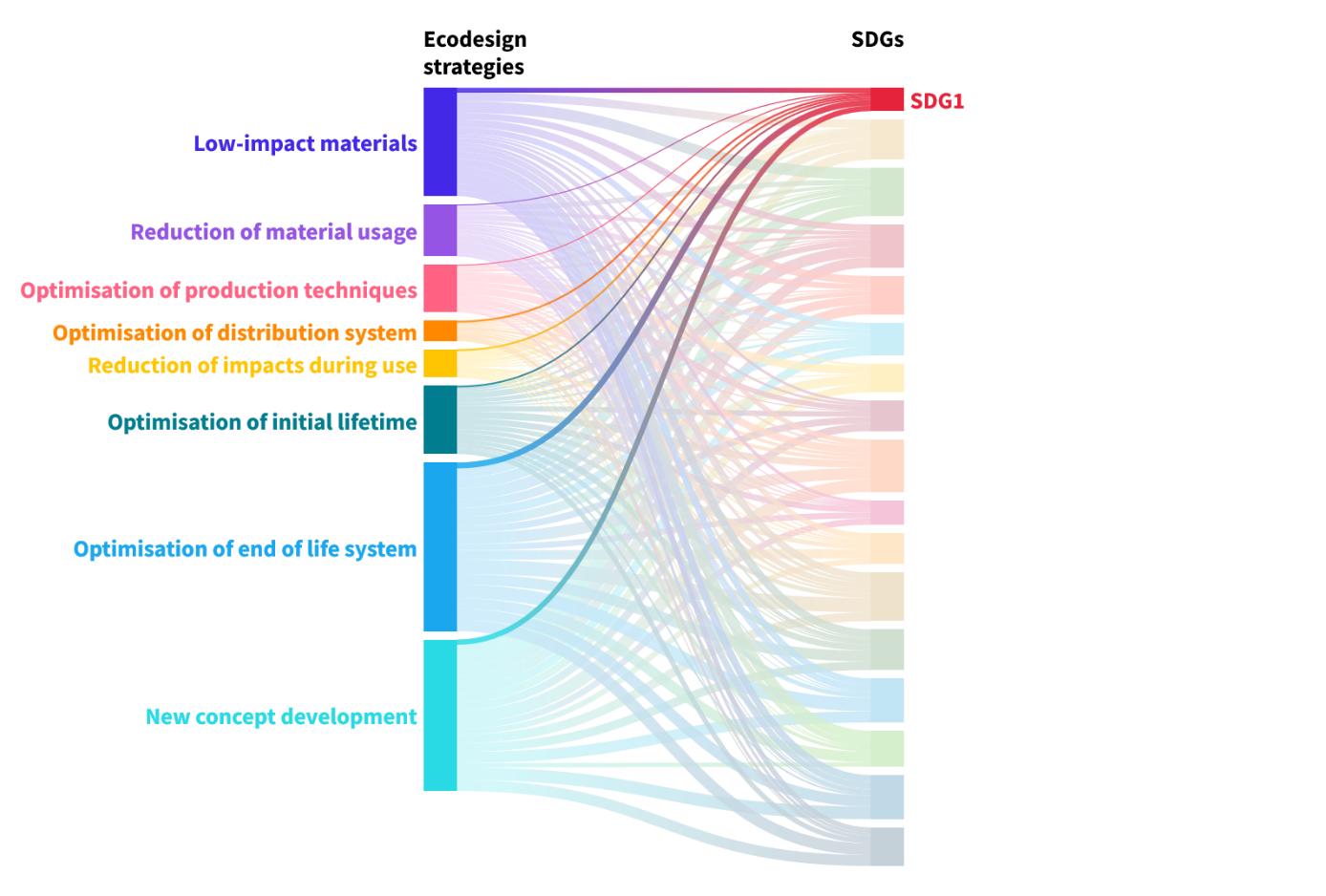


**Figure S 38** Relationship of the chosen studies in SDG 1 with the ecodesign strategies (Created with Flourish^[11]^)


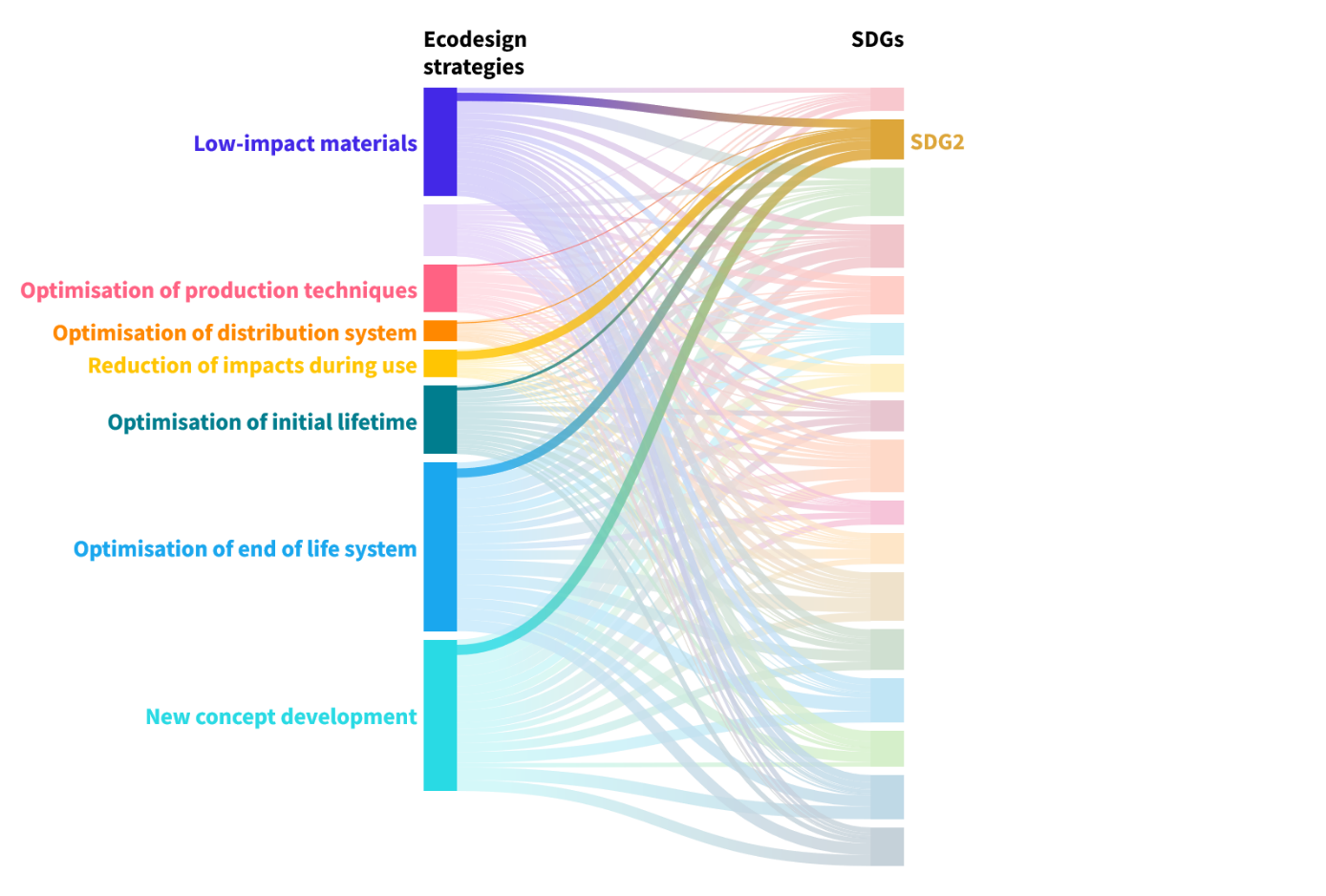


**Figure S 39** Relationship of the chosen studies in SDG 2 with the ecodesign strategies (Created with Flourish^[11]^)


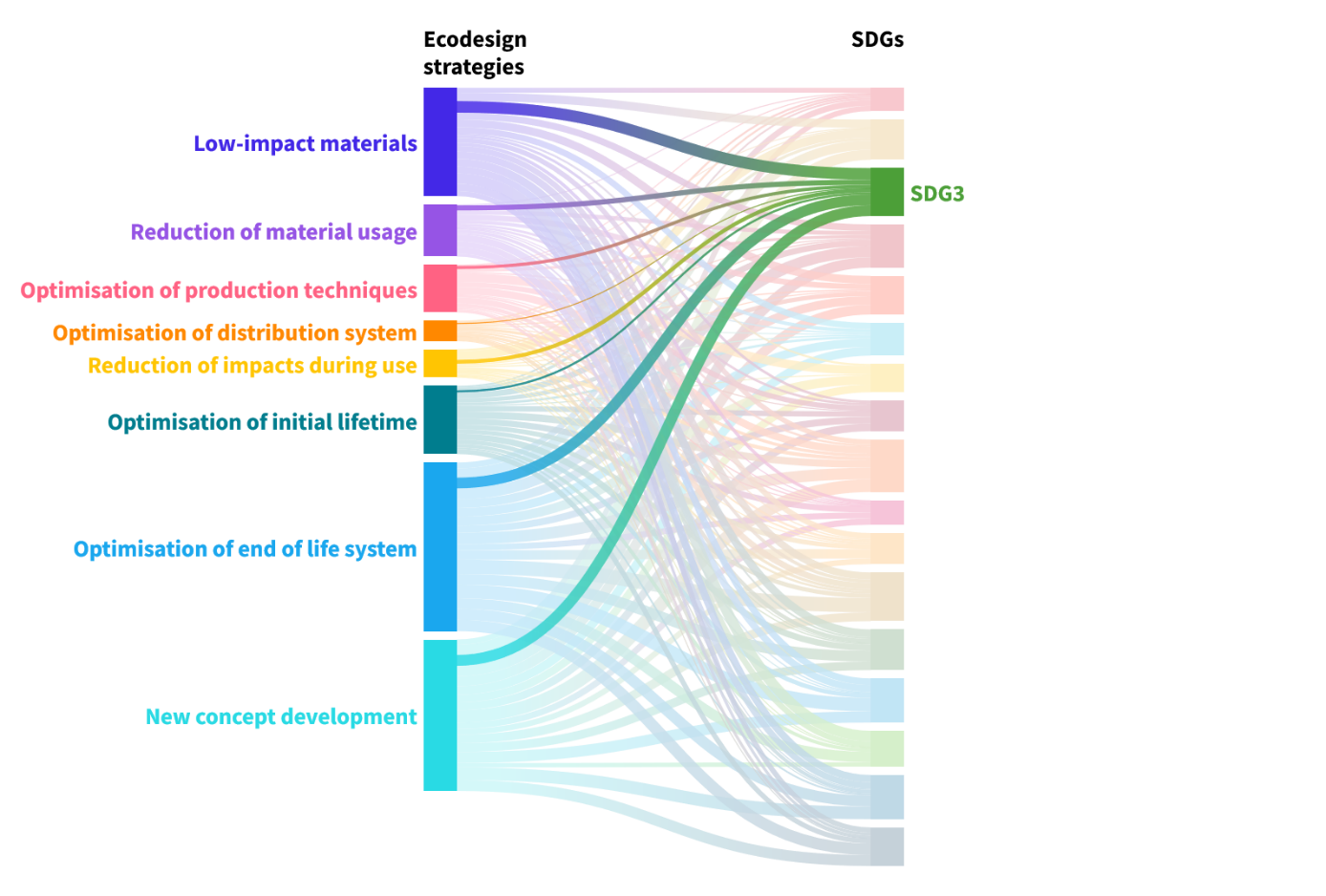


**Figure S 40** Relationship of the chosen studies in SDG 3 with the ecodesign strategies (Created with Flourish^[11]^)


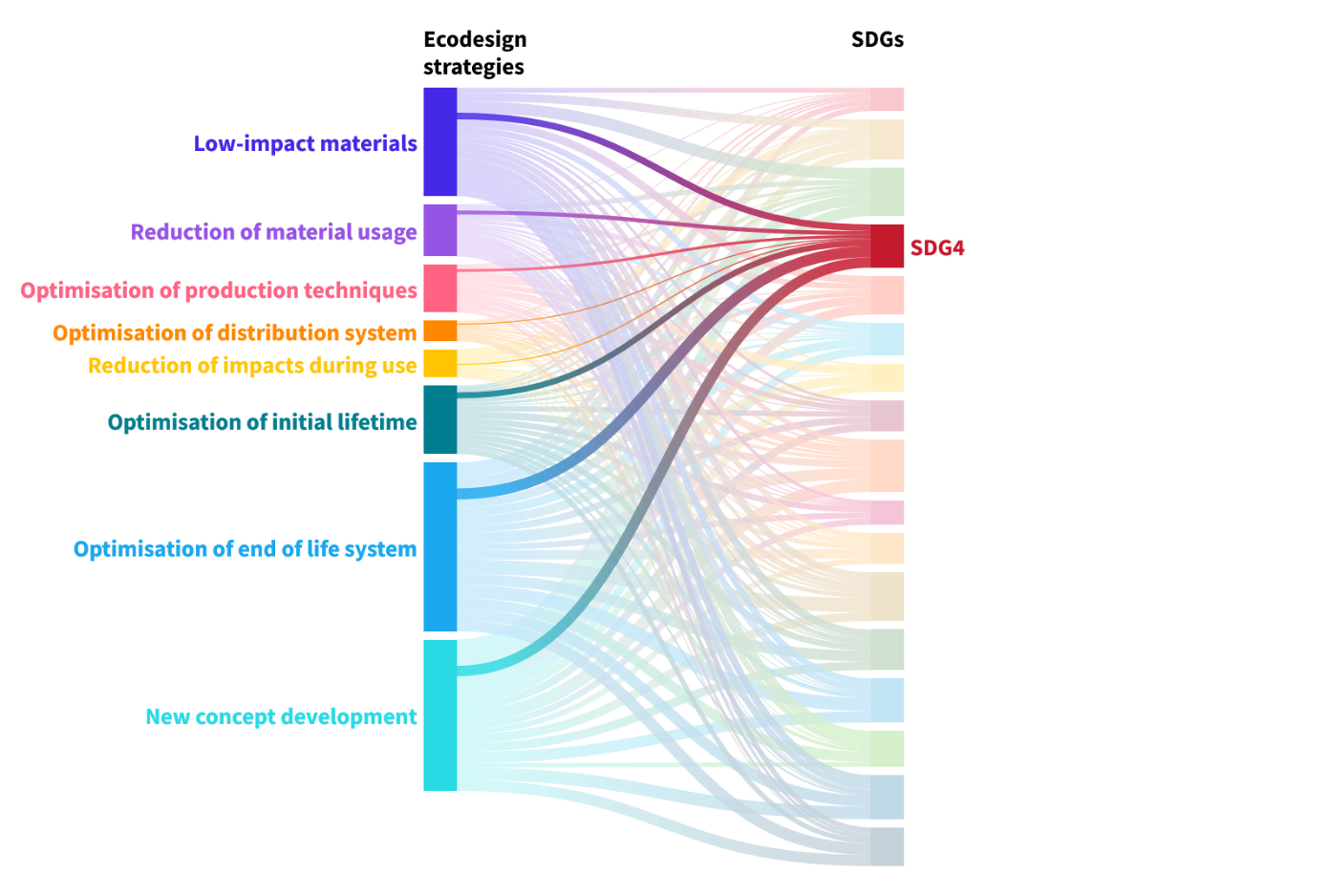


**Figure S 41** Relationship of the chosen studies in SDG 4 with the ecodesign strategies (Created with Flourish^[11]^)


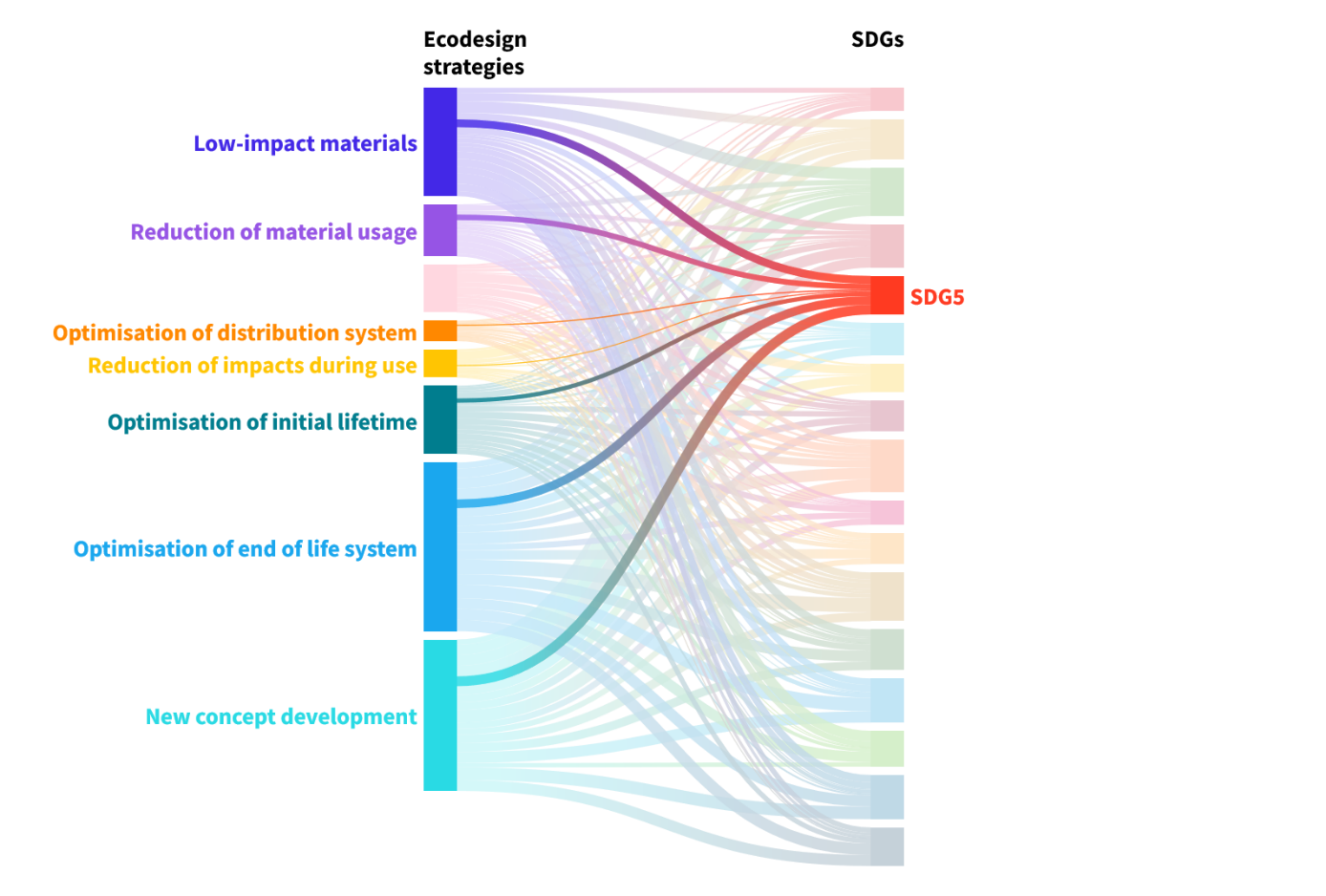


**Figure S 42** Relationship of the chosen studies in SDG 5 with the ecodesign strategies (Created with Flourish^[11]^)


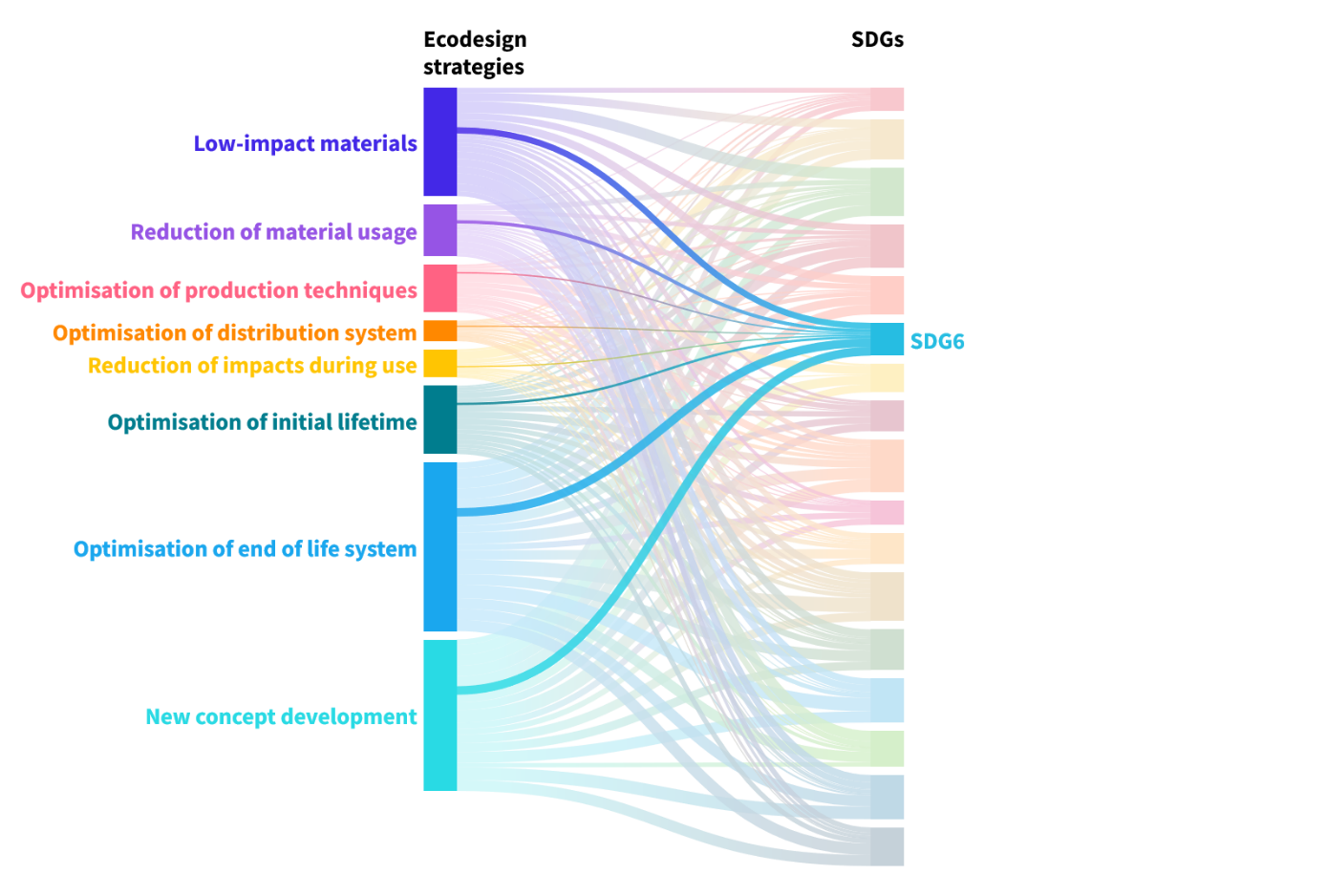


**Figure S 43** Relationship of the chosen studies in SDG 6 with the ecodesign strategies (Created with Flourish^[11]^)


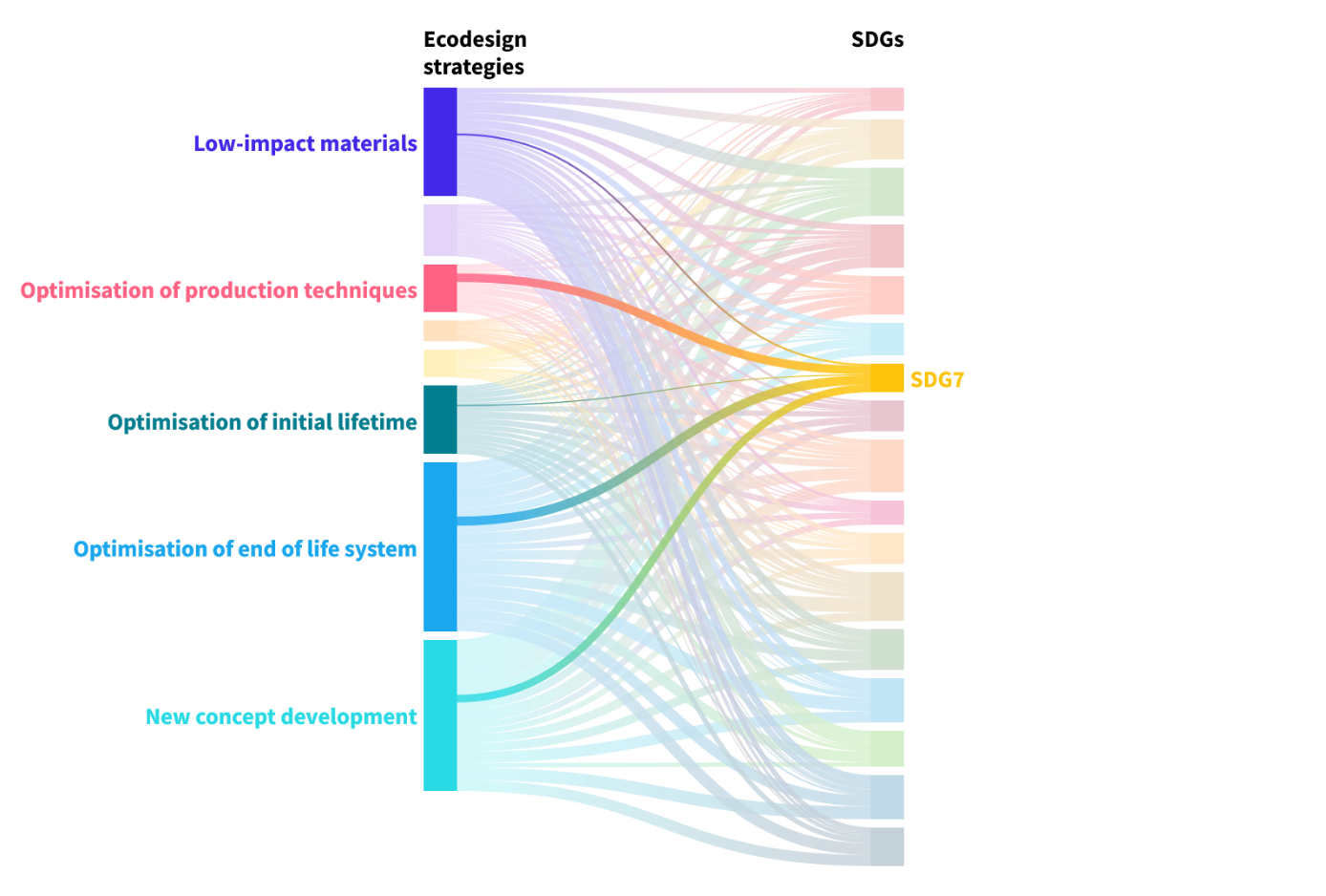


**Figure S 44** Relationship of the chosen studies in SDG 7 with the ecodesign strategies (Created with Flourish^[11]^)


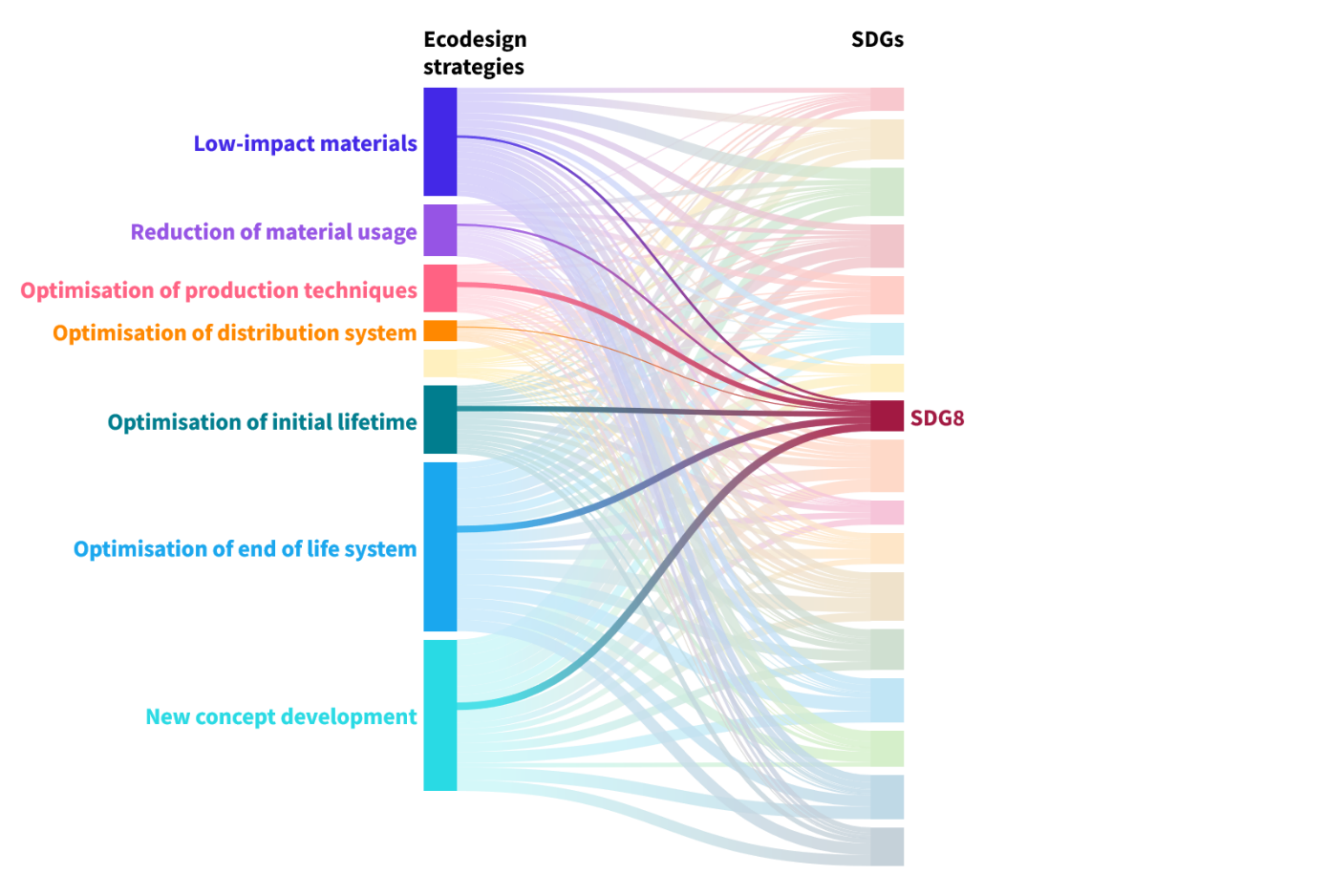


**Figure S 45** Relationship of the chosen studies in SDG 8 with the ecodesign strategies (Created with Flourish^[11]^)


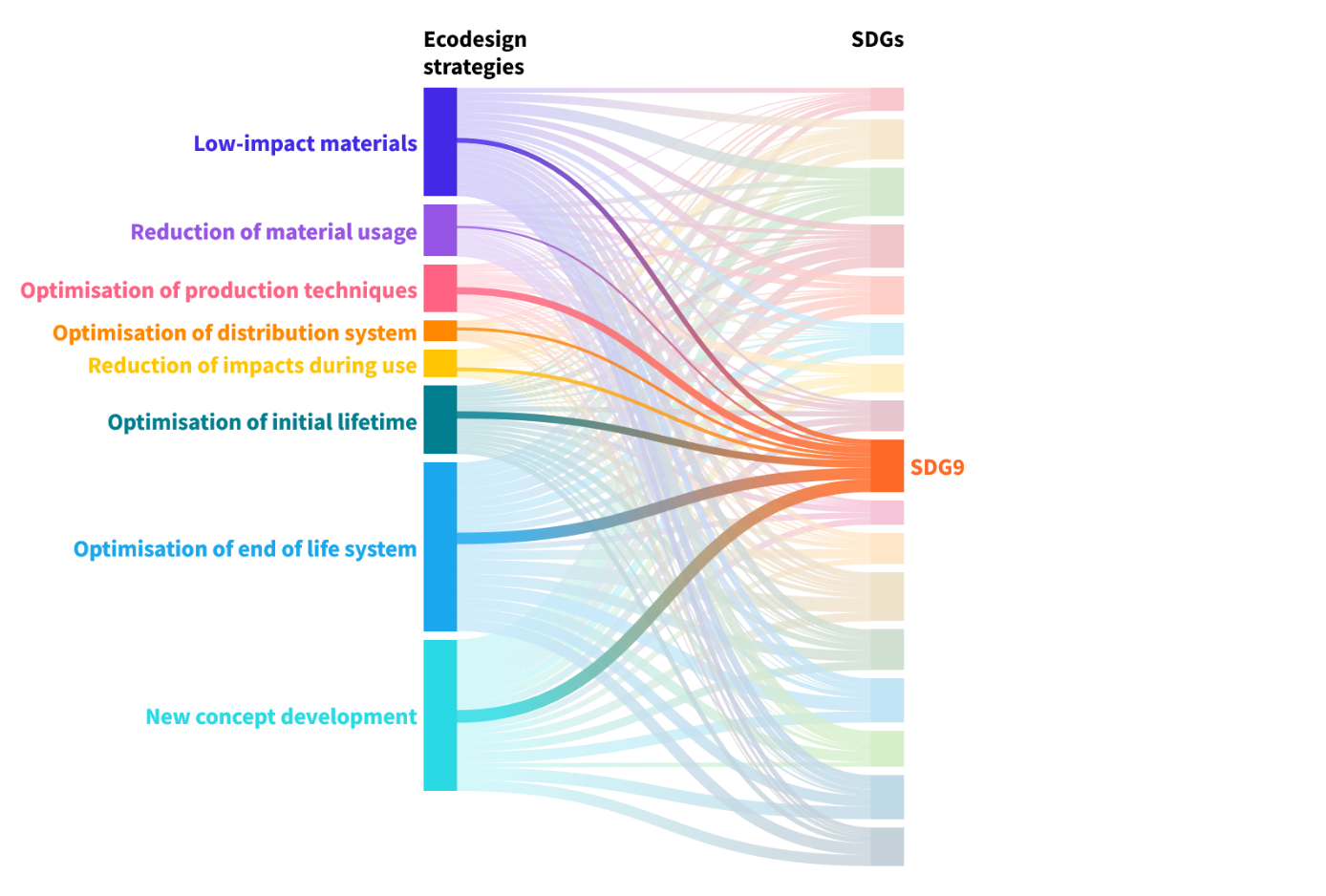


**Figure S 46** Relationship of the chosen studies in SDG 9 with the ecodesign strategies (Created with Flourish^[11]^)


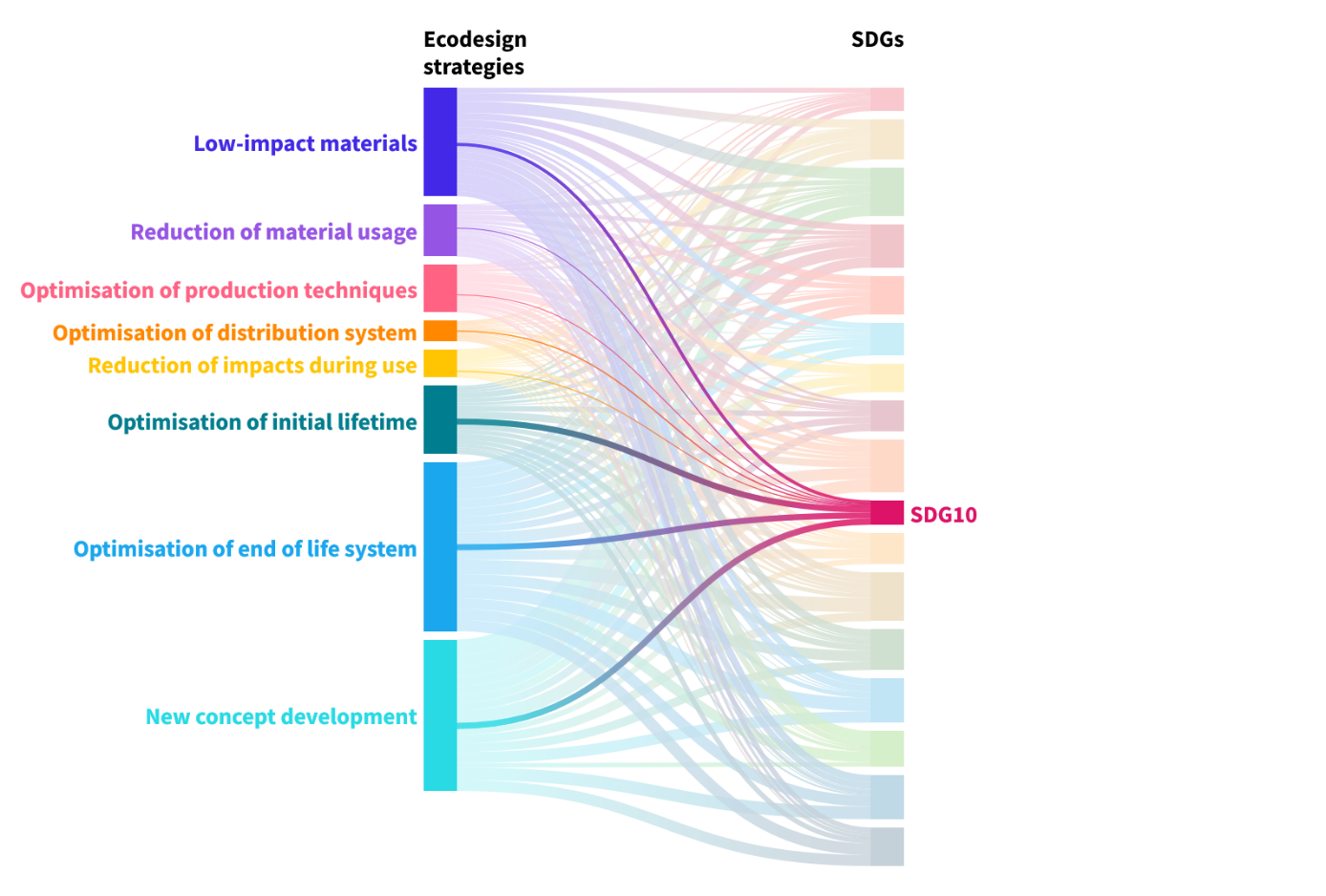


**Figure S 47** Relationship of the chosen studies in SDG 10 with the ecodesign strategies (Created with Flourish^[11]^)


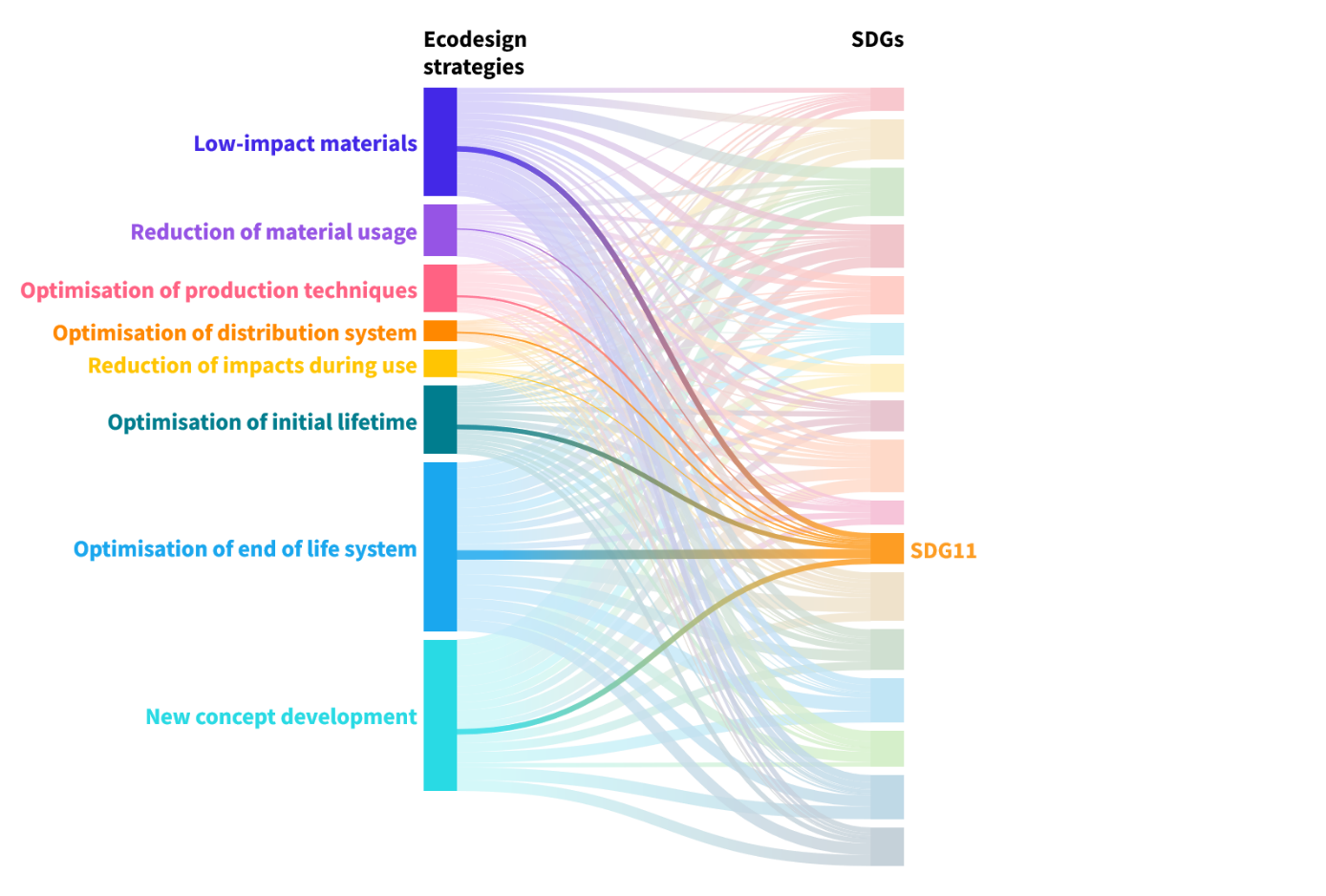


**Figure S 48** Relationship of the chosen studies in SDG 11 with the ecodesign strategies (Created with Flourish^[11]^)


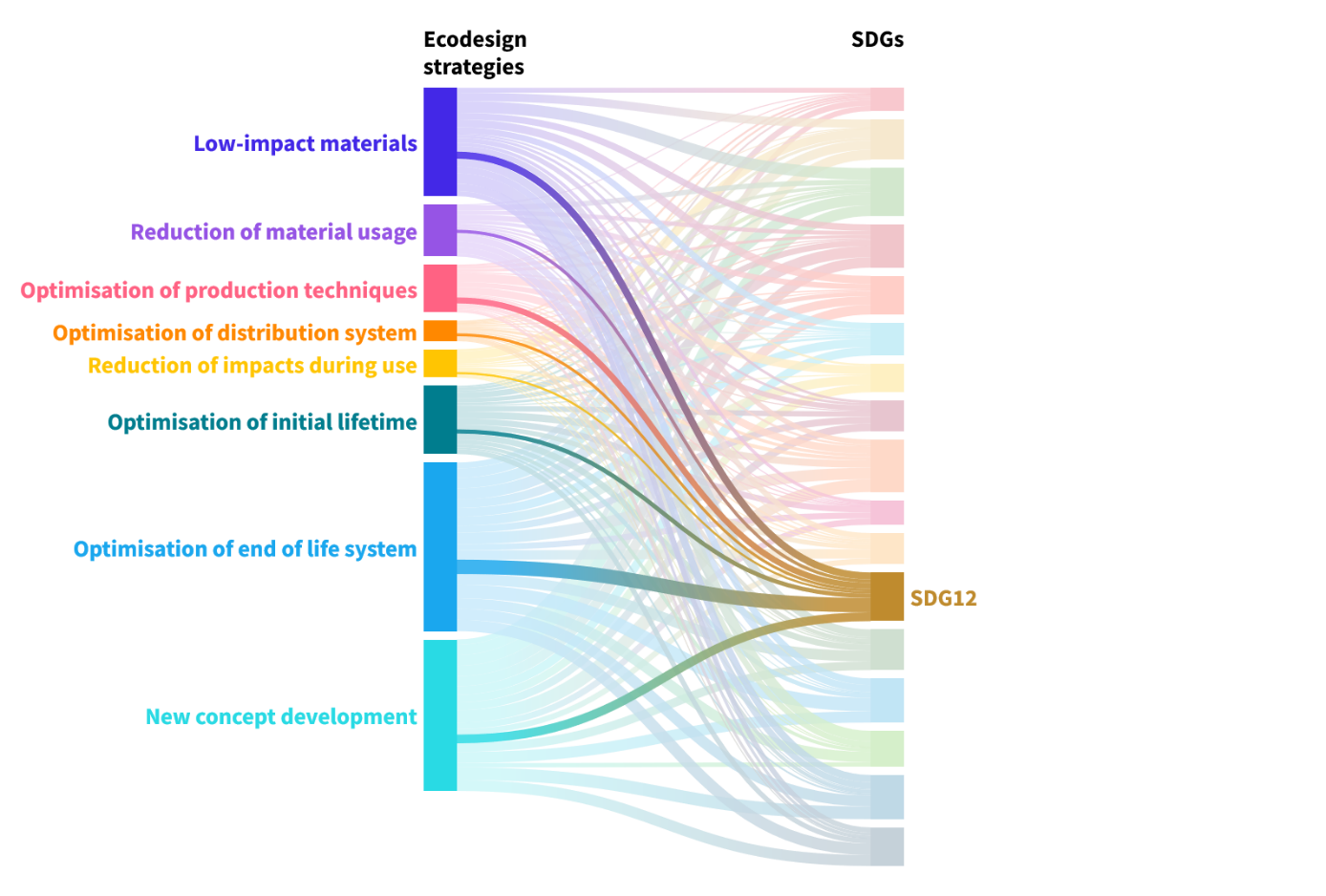


**Figure S 49** Relationship of the chosen studies in SDG 12 with the ecodesign strategies (Created with Flourish^[11]^)


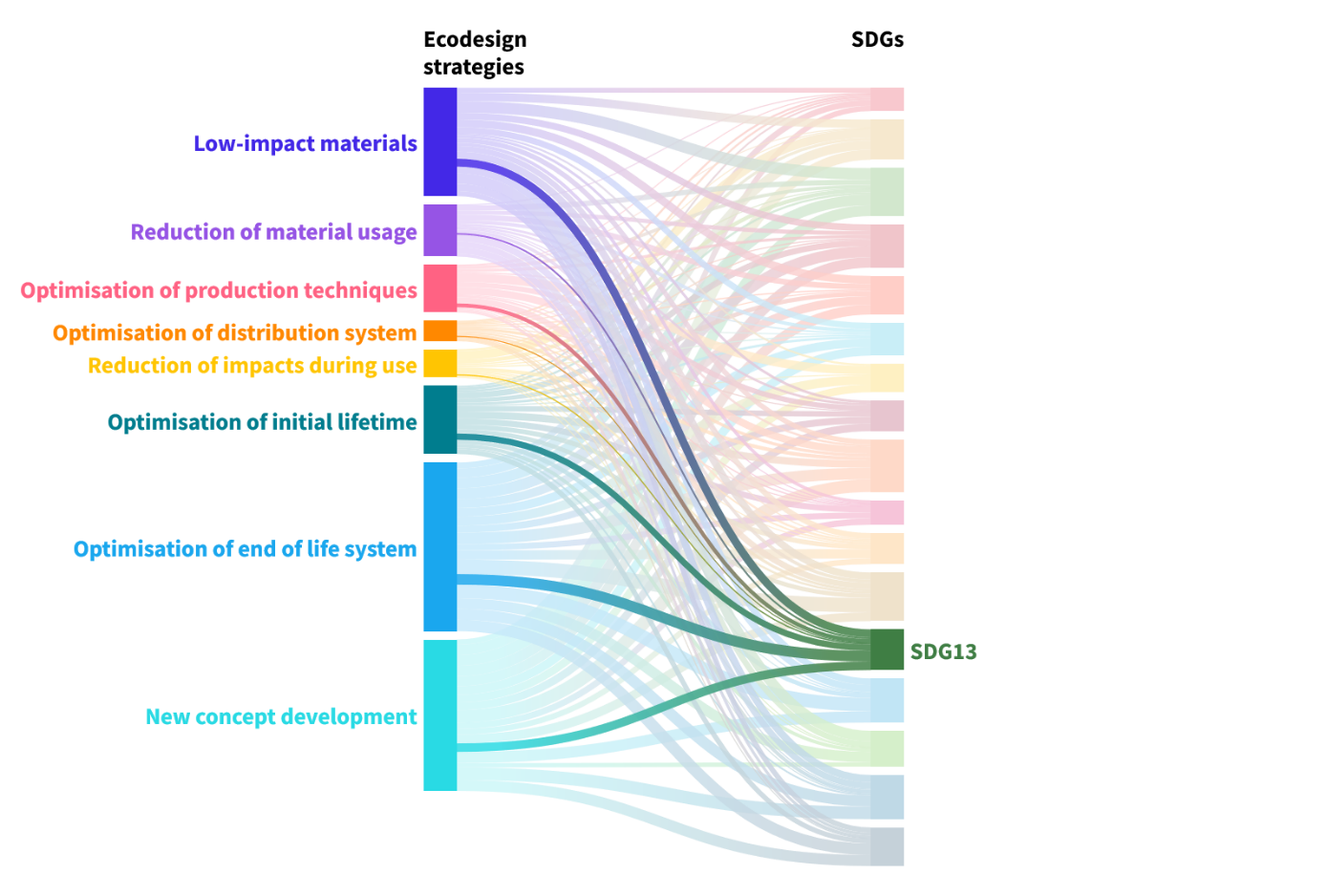


**Figure S 50** Relationship of the chosen studies in SDG 13 with the ecodesign strategies (Created with Flourish^[11]^)


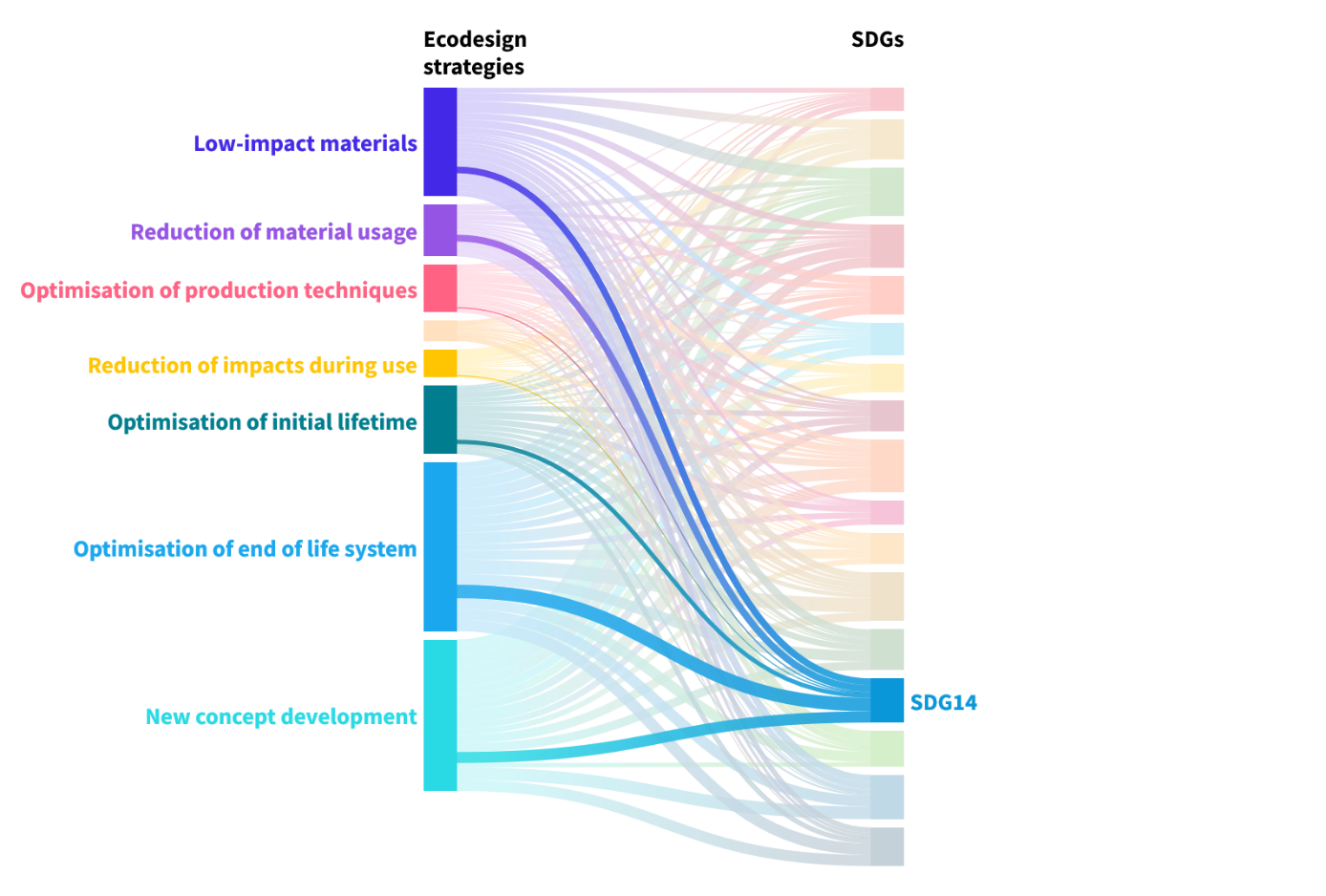


**Figure S 51** Relationship of the chosen studies in SDG 14 with the ecodesign strategies (Created with Flourish^[11]^)


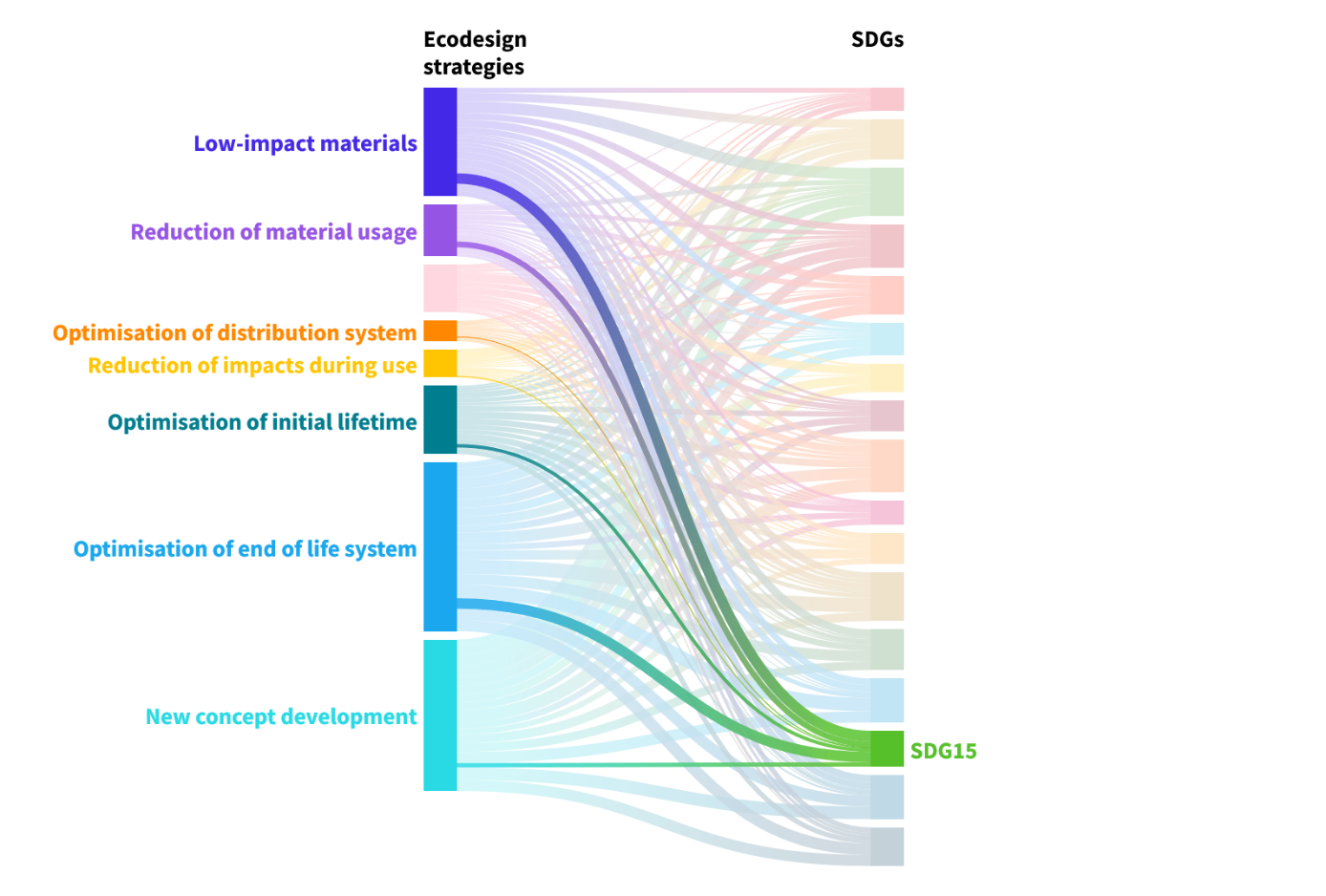


**Figure S 52** Relationship of the chosen studies in SDG 15 with the ecodesign strategies (Created with Flourish^[11]^)


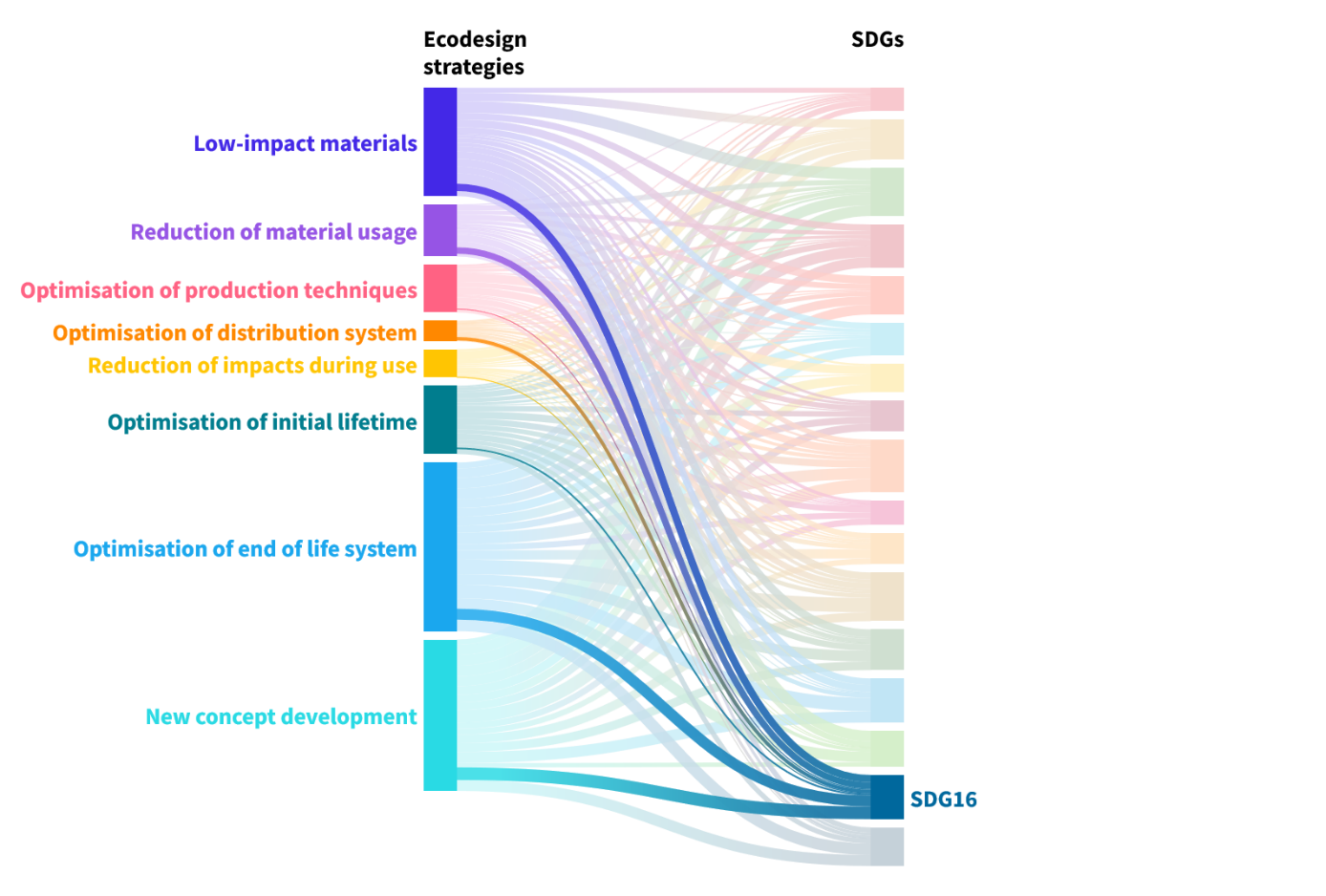


**Figure S 53** Relationship of the chosen studies in SDG 16 with the ecodesign strategies (Created with Flourish^[11]^)


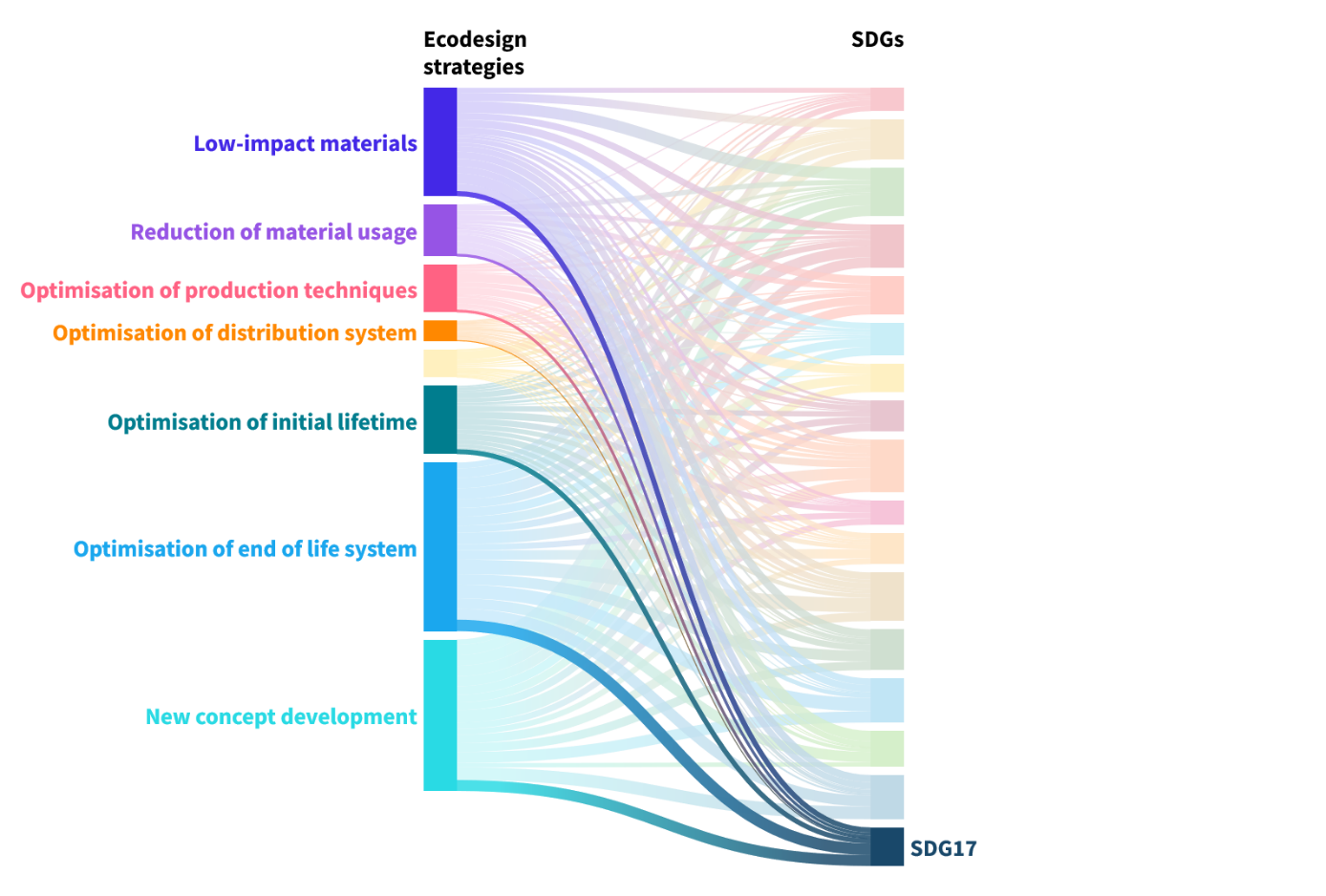


**Figure S 54** Relationship of the chosen studies in SDG 17 with the ecodesign strategies (Created with Flourish^[11]^)

**Table S 3** Indicators identified for the parameters within the Plastic Ecodesign Index Score methodology

| Nr. | Parameter | Indicator | Description | Unit |
| --- | --- | --- | --- | --- |
| 1 | Less use of additives | ed1_cleanmat | Amount of harmful additives in the material/product composition that are not recoverable after use (%) | % |
| 2 | Use of renewable materials | ed1_renewmat | Amount of bio-based and other renewable alternatives in the material composition (%) | % |
| 3 | Use of recyclates in production | ed1_recymat | Amount of recyclates in the material composition to fulfill same function (%) | % |
| 4 | Recyclable materials after production | ed1_recyblmat | Total substitution potential of the recyclates of the material/product after use (Dimensionless) | No unit |
| 5 | Reduction in weight of products | ed2_redwt | Percentage reduction in the weight through different measures to fulfill same function (%) | % |
| 6 | Reduction in transport volume | ed2_redtransvol | Percentage reduction in volume while transporting (%) | % |
| 7 | Renewable energy in production | ed3_renewener | Amount of renewable energy used in the production of materials/products (%) | % |
| 8 | Recovery of wastes during production | ed3_cleanprod | Percentage of wastes generated that are not recovered (%) | % |
| 9 | Reduced energy consumption | ed3_enercons | Reduction in the energy consumption of the machines and infrastructure (%) | % |
| 10 | Recovery of auxiliary materials (waters, chemicals) | ed3_auxmat | Recovery and reuse of auxiliary materials during the production (%) | % |
| 11 | Electric vehicle fleet | ed4_cleantrans | Percentage of EV fleet for the distribution of the finished products (%) | % |
| 12 | Reduced air transport | ed4_airtrans | Percentage of materials transported via air transport (%) | % |
| 13 | Recyclable packaging material | ed4_packmat | Recyclability of product packaging after use (based on the polymer specific recycling rate) (%) | % |
| 14 | Reuse of packaging material | ed4_packre | Reuse of packaging material (%) | % |
| 15 | Labelling of products for recyclability | ed5_label | Amount of labelling done for the different polymers used in the product | % |
| 16 | Increase in lifetime of products with multiple use | ed5_life | Increase in the lifetime of the products/components/materials during use (based on the industrial average) | Years |
| 17 | Reduction in resource consumption during use | ed5_rescons | Percentage reduction in the resource consumption for the product during use phase | % |
| 18 | Availability of instructions to repair | ed6_repair | Availability of instructions to repair each and every component during use phase | % |
| 19 | Availability of spare parts | ed6_spare | Availability of spare parts to repair each and every component during use phase | % |
| 20 | Ease of disassembly for repair | ed6_design | Percentage of components that can be easily disassembled | % |
| 21 | Products recovered for reuse, refurbish and recycle | ed7_recsys | Percentage of products/components that can be recovered for reuse and recycling | % |
| 22 | Monomaterials used in the product | ed7_mono | Amount of mono-materials used in the product that can produce homogenous recyclates | % |
| 23 | Price of recyclates from the product | ed7_recprice | Price of recyclates from the products competitive to that of the virgin polymers | $/kg |
| 24 | Products that are incinerated after use | ed7_inc | Percentage of products/components that are incinerated (region-specific, polymer-specific) | % |
| 25 | Products that are landfilled and disposed after use | ed7_dis | Percentage of products/components that are disposed (region-specific, polymer-specific) | % |
| 26 | Product as service (leased, shared) | ed8_sharedcomp | Amount of products/components out of plastic that can be leased/shared | % |
| 27 | Data management for Sustainability Assessment | ed8_LCSA | Number of Life Cycle Sustainability Assessment studies conducted for products and materials in the portfolio | No unit |
| 28 | Take-back facilities for products | ed8_takeback | Amount of materials and components recovered from the take-back facilities | % |
| 29 | Regional recycling infrastructure | ed8_recinfra | Amount of products produced/distributed/used in a region where there is a lack of recycling infrastructure | % |
| 30 | Reduction of product use in regions  with uncontrolled disposal | ed8_disposal | Amount of products used in regions where uncontrolled disposal takes place | MT or % |

**References**

[1] Kuori Website, *Our Materials | Kuori Website.* **2025.** https://www.kuori.ch/our-materials.

[2] Engel, *Plastic recycling process - ENGEL.* **2025.** https://www.engelglobal.com/en/products/injection-moulding-processes/plastic-recycling-process#two-stage-process.

[3] Holypoly, *Recycling mit Designanspruch: LAMY×HolyPoly.* **2024.** https://www.holypoly.co/de/news/PM-Lamy-aquaplus-re.

[4] Cirplus GmbH, *Cirplus launches new services for consistent quality in the procurement of recycled plastics.* **2023.** https://drive.google.com/file/d/1GA1VwXVzr3cOkDxyEbljOKNTxTlOvbDi/view.

[5] Pact Group, *Barrier packaging for chemicals | Pact Group.* **2025.** https://pactgroup.com/products-services/packaging/household-industrial/barrier_packaging_for_chemicals/.

[6] N. J. van Eck, L. Waltman, *Scientometrics.* **2010**, *84*, 523.

[7] Chris James, *Elsevier 2023 Sustainable Development Goals (SDGs) Mapping*, Elsevier BV. **2023**.

[8] R. Kumar, A. Verma, A. Shome, R. Sinha, S. Sinha, P. K. Jha, R. Kumar, P. Kumar, Shubham, S. Das, P. Sharma, P. V. Vara Prasad, *Sustainability.* **2021**, *13*, 9963.

[9] United Nations Statistics, *SDG Indicators.* **2024.** https://unstats.un.org/sdgs/indicators/indicators-list/.

[10] N. Navarre, J. M. Mogollón, A. Tukker, V. Barbarossa, *Resources, Conservation and Recycling.* **2022**, *185*, 106508.

[11] Flourish, *https://flourish.studio.* **2024.** https://flourish.studio/.
