# Supplementary material for: Plastics and Sustainable Development – Identifying and Quantifying Ecodesign Strategies for Plastics
Source: Glob Chall. 2026 Mar 23;10(3):e00033. doi: 10.1002/gch2.202500033 (PMC13093836; doi:10.1002/gch2.202500033)

WILEY-VCH

Supporting Information 2 (SI2)

Plastics and Sustainable Development - A Framework to Identify and Quantify Ecodesign Strategies for Plastics - A Literature-based Analysis

Venkateshwaran Venkatachalam [*], *Sebastian Spierling, Mikolaj Owsianiak, Frederik R. Wurm, Leonie Barner and Hans-Josef Endres*

[*] Correspondence: venkatachalam@ikk.uni-hannover.de, Tel.: +49-511-762-13328

List of tables and figures

Document	Page
Screening analysis of literature for qualitative assessment	2
Qualitative assessment of the literature allocated to 17 SDGs	3 - 9
Scoring of the impacts of plastics based on qualitative assessment	10 - 19
Relationship between the literature and ecodesign strategies	20 - 25
Interaction between literature allocated to an SDG towards other SDGs	26
Keyword occurrence for the literature allocated to 17 SDGs	27

Screening analysis of literature for qualitative assessment					
SDG	Keywords (Apart from 'Plastic', 'Polymer')	Total results	Studies selected for title and abstract scan	Studies selected for full-paper and eligibility scan	Studies selected for the review
SDG 1 - No Poverty	Poverty, People, Population	49	25	15	10
SDG 2 - Zero Hunger	Agriculture, Hunger, Food, Food security	119	70	45	20
SDG 3 - Good Health and Well-being	Health, Death, Illness, Disease, Mortality	109	75	38	20
SDG 4 - Quality Education	Education, Skills, Qualification	153	50	28	20
SDG 5 - Gender Equality	Gender, Women, Equality, Policy	65	40	26	20
SDG 6 - Clean Water and Sanitation	Water, Sanitation, Drinking, Scarcity	133	67	32	20
SDG 7 - Affordable and Clean Energy	Energy, Renewable, Sustainable, Clean	70	42	28	20
SDG 8 - Decent Work and Economic Growth	Economy, Job, Growth, Employment	61	39	25	20
SDG 9 - Industry, Innovation and Infrastructure	Innovation, Infrastructure, Research, Industry	76	59	42	30
SDG 10 - Reduced Inequalities	Income, Injustice, Inequality, Social	70	53	38	20
SDG 11 - Sustainable Cities and Communities	Cities, Municipal, Waste, Communities, Settlements	82	60	43	20
SDG 12 - Responsible Consumption and Production	Sustainable, Production, Consumption, Footprint, Waste, Efficiency	197	83	57	30
SDG 13 - Climate Action	Climate Change, Global Warming, Mitigation	82	62	48	30
SDG 14 - Life Below Water	Marine, Pollution, Coast, Fishing	230	108	56	30
SDG 15 - Life on Land	Ecosystem, Soil, Land, Terrestrial, Wildlife, Wetlands	195	87	51	30
SDG 16 - Peace, Justice and Strong Institutions	Justice, Peace, Regulation, Legislation, Governance, Policy	94	62	38	20
SDG 17 - Partnership for the Goals	Partnership, Cooperation, Global, Trade, Market	209	72	42	20
	**Total**	**1994**	**1054**	**652**	**380**

Qualitative assessment of the literature allocated to 17 SDGs

Studies considered for the qualitative assessment							Impacts of plastics on the achievement of SDGs (Highly beneficial, Beneficial, Neutral, Critical, Highly critical)					Interactions with other SDGs		Ecodesign strategies and their contribution to achieve the targets/indicators (Strong relationship, Moderate relationship, Unclear relationship, No relationship)							
S.No.	Author	Title of the study	Year	Region	Link	Summary/Important observations	Raw material	Production	Use	End of Life	Transportation/Logistics	Relationship with SDGs (Scopus)	Possible relationship with other SDGs	Low-impact materials	Reduction of materials usage	Optimisation of production techniques	Optimisation of distribution system	Reduction of impacts during use	Optimisation/Extension of lifetime	Optimisation of end-of-life system	New concept development
Goal 1. End poverty in all its forms everywhere	Keywords: Poverty, People, Population																				
1	Mpanangombe et al.	Poverty, politics and plastic - Organic waste sorting in Blantyre's public markets	2021	Africa	https://www.sciencedirect.com/science/article/pii/S2226585621000352?pes=vor	• Plastic contamination with the organic wastes and mindless disposal in Malawi • Policies to incentivise better waste separation	Critical	Critical	Critical	Highly critical	Critical	11,12,16,17	4, 8, 9, 13	Moderate	No	No	No	Strong	No	Strong	Strong
2	David Katz	Plastic Bank: launching Social Plastic® revolution	2019	Asia, Latin America	https://journals.openedition.org/factsreports/5478#text	• Stop ocean plastic while reducing poverty • Exchanging plastic wastes for cash • Recycled plastic wastes are sold to the companies	Critical	N/A	N/A	Neutral	Neutral	8, 9, 10, 12, 14, 17	1, 4, 11	Strong	No	No	Moderate	No	No	Strong	Moderate
3	Mukama et al.	Practices, Concerns, and Willingness to Participate in Solid Waste Management in Two Urban Slums in Central Uganda	2016	Africa	https://www.hindawi.com/journals/jeph/2016/6830163/	• Quantitative data collected from 435 residents on waste disposal • Plastics were the most reused wastes • Plastic collection and dsipopsal practices create difficulty for recollection and recycling	Critical	N/A	Critical	Highly critical	Highly critical	11,12,17	1, 3, 6, 8, 13	Moderate	No	No	No	No	No	Strong	Moderate
4	Gall et al.	Building a circular plastics economy with informal waste pickers: Recyclate quality, business model, and societal impacts	2020	Africa	https://www.sciencedirect.com/science/article/pii/S0921344920300070	• Interviews done with waste pickers, management team of a plastic recycling company in Nairobi, Kenya • Analysis of the recyclates were conducted • A reliable supplier network needed to contact pickers with formal recycling chain	Critical	N/A	N/A	Critical	Critical	8,9,12,17	1, 10	Strong	Unclear	No	Moderate	Unclear	No	Strong	Moderate
5	El Mekaoui et al.	Plastic bags ban and social marginalization: Evidence from Morocco	2021	Africa	http://www.pjoes.com/Plastic-Bags-Ban-and-Social-Marginalization-nEvidence-from-Morocco,132981,0,2.html	• Implications of plastic ban in Morocco on the informal market vendors, poverty and social inequality • Rise of plastic bags illegally entering the markets as the customers got used to carry all their groceries	Beneficial	Beneficial	Beneficial	Critical	N/A	1, 8, 10, 17	9, 11	Strong	No	No	Unclear	Unclear	Moderate	Moderate	Moderate
6	Jenks et al.	The poverty of plastics bans: Environmentalism's win is a loss for disabled people	2020	Global	https://journals.sagepub.com/doi/10.1177/0261018319868362	• How straw bans can be disadvantageous to disabled people. • Banning single use plastic straws wont solve the plastic problem but by banning them is a loss for disabled people who cant opt other alternatives	Beneficial	Beneficial	Beneficial	Critical	N/A		8, 10	Strong	No	No	No	Moderate	Moderate	Moderate	Strong
7	Stoler et al.	Piped water flows but sachet consumption grows: The paradoxical drinking water landscape of an urban slum in Ashaiman, Ghana	2021	Africa	https://www.sciencedirect.com/science/article/pii/S0197397515000107?pes=vor	• Household survey of 95 residents on complexity of drinking water decision making in low-income societies and consumption of sachet water • Different social and economic factors play roles when people make decisions to buy water in sachets	N/A	N/A	Beneficial	Critical	Beneficial	1, 6.11, 17	10, 12, 14	Unclear	No	No	Moderate	No	No	Strong	Moderate
8	Velis et al.	Enabling the informal recycling sector to prevent plastic pollution and deliver an inclusive circular economy	2022	Africa	https://www.sciencedirect.com/science/article/pii/S1462901122002866?pes=vor	• Policy interventions to support the informal recycling sector and to improve the plastic recycling and reduce environmental risks • Improve informal recycling sector, revenue from recycled materials and material quality can reduce the impacts of plastic pollution	Critical	Critical	Critical	Critical	Neutral	11, 14, 17	1, 8, 9, 10, 12	No	No	No	No	No	No	Strong	Strong
9	Obiadi Bons N. Onochie A.O	The negative impacts of poverty in urban and rural architecture in Nigeria	2019	Africa	https://www.ijstr.org/final-print/mar2019/The-Negative-Impacts-Of-Poverty-In-Urban-And-Rural-Architecture-In-Nigeria.pdf	• With the abundance of plastic bottles and soil,poor communities in Nigeria (as they dont have access to better building materials and due to bad governance) are now building with abandoned plasti bottles filled with sand	Beneficial	N/A	Beneficial	Beneficial	N/A	1, 17	9, 10, 11, 12	Strong	No	No	No	Moderate	Moderate	Strong	Strong
10	Klocker et al.	From troublesome materials to fluid technologies: Making and playing with plastic-bag footballs	2018	Africa	https://journals.sagepub.com/doi/10.1177/1474474017732979	• Footballs made of plastic bags appear as symbols of poverty, material deprivation and ultimately inferior • Through recycling plastic bags and sourcing them from landfill sites, the youngsters are recovering the plastic wastes to make footballs out of them	Highly critical	Critical	Critical	Beneficial	N/A	17	1, 10, 12	Strong	Strong	Strong	Moderate	No	Moderate	Strong	Strong
Goal 2. End hunger, achieve food security and improved nutrition and promote sustainable agriculture	Keywords: Agriculture, Hunger, Food, Food security																				
1	He et al.	Plastic mulch: Tradeoffs between productivity and greenhouse gas emissions	2018	Asia	https://www.sciencedirect.com/science/article/abs/pii/S0959652617325738?via%3Dihub	• Plastic mulch increase the yield and productivity of crops but at the same time cause increased GHG emissions • Calculate the GHG emissions from LCA and yield of using Plastic mulch • Analyse the tradeoffs between them	Highly critical	Highly critical	Neutral	Highly critical	Highly critical	9,12,13	2, 15	Strong	No	No	Unclear	Moderate	Moderate	Strong	Strong
2	Saeed et al.	Competency of groundwater recharge of irrigated cotton field subjacent to sowing methods, plastic mulch, water productivity, and yield under climate change	2022	Asia	https://link.springer.com/article/10.1007/s11356-021-17017-0	• Cotton bed without plastic mulching improved the productivity and water use efficiency more than with mulch • Recommended that current cotton sowing to be replaced with bed planting and plastic mulching to be prohibited	Critical	N/A	Critical	N/A	N/A	2, 6, 8, 13	15	No	No	No	No	Moderate	No	No	Unclear
3	Wang et al.	Plastic mulching reduces nitrogen footprint of food crops in China: A meta-analysis	2020	Asia	https://www.sciencedirect.com/science/article/abs/pii/S0048969720350087?via%3Dihub	• Plastic mulching (PM) enhanced grain yield and reduced N footprint resulted in less reactive nitrogen loss • PM could be regarded as a suitable practice for sustainable intensification of crop production in China when suitable crop species and local environments are considered.	Beneficial	N/A	Beneficial	N/A	N/A	8, 17	2, 6, 15	No	No	No	No	Moderate	No	Moderate	No
4	Castillo-Diaz et al.	The management of agriculture plastic waste in the framework of circular economy. Case of the almeria greenhouse (Spain)	2021	Europe	https://mdpi-res.com/d_attachment/ijerph/ijerph-18-12042/article_deploy/ijerph-18-12042-v2.pdf?version=1637162725	• Estimate the production of agricultural plastic wastes from greenhouse in Almeira • Identify existing alternatives and relation between oil price and recycled plastics	Critical	N/A	Neutral	Highly critical	Critical	2, 12	6, 13, 14, 15, 17	Strong	No	Moderate	Moderate	Strong	Moderate	Strong	Strong
5	Batista et al.	Dealing with Plastic Waste from Agriculture Activity	2022	Europe	https://mdpi-res.com/d_attachment/agronomy/agronomy-12-00134/article_deploy/agronomy-12-00134-v4.pdf?version=1642644087	• Managing and controlling the plastic wastes that arise from the agriultural activities • Disposed plastic wastes can be used as precursor for Activated Carbon manufacture	Critical	N/A	Critical	Critical	N/A	6	2, 12, 13, 14, 15	Strong	No	No	No	Unclear	No	Strong	Strong
6	Pazienza et al.	For a new plastics economy in agriculture: Policy reflections on the EU strategy from a local perspective	2020	Europe	https://www.sciencedirect.com/science/article/abs/pii/S0959652619347146	• Evaluate economic policy and their social implications in effectively managing plastic wastes in agriculture • Farmers are being interviewed on their opinion on the economic tools (EPR/ax subsidy) on the plastic wastes	Critical	Critical	Highly critical	Highly critical	Neutral	2, 3,12,14,17	8, 15	Unclear	No	No	No	Moderate	No	Moderate	Strong
7	Chen et al.	Response of carbon footprint to plastic film mulch application in spring maize production and mitigation strategy	2021	Asia	https://www.sciencedirect.com/science/article/pii/S2095311920632786?pes=vor	• Highlights the necessity of reducing Plastic mulching in agriculture • Quantifies the carbon footprint of PM	Highly critical	Highly critical	Neutral	Highly critical	N/A	2, 8, 12, 13, 17	14, 15	Unclear	No	No	No	Strong	No	Moderate	Strong
8	Xie et al.	Yield, economic benefit, soil water balance, and water use efficiency of intercropped maize/potato in responses to mulching practices on the semiarid loess plateau	2021	Asia	https://mdpi-res.com/d_attachment/agriculture/agriculture-11-01100/article_deploy/agriculture-11-01100-v3.pdf?version=1636613098	• Intercropping of potatoes with maize (grown with plastic mulching) and their effects on yield, water consumption were analysed	Beneficial	N/A	Beneficial	Critical	N/A	2,6,8		No	No	No	No	Moderate	No	No	Moderate
9	Zhang et al.	Plastic pollution in croplands threatens long-term food security	2020	Asia	https://onlinelibrary.wiley.com/doi/epdf/10.1111/gcb.15043?src=getftr	• Meta-analysis on agricultural benefits and impacts of plastic mulching • Nation wide soil sampling for plastic film residues	Critical	Critical	Beneficial	Highly critical	N/A	2,3,14,17	12, 13, 15	Strong	No	No	No	Strong	No	Strong	Moderate
10	Lwanga et al.	Field evidence for transfer of plastic debris along a terrestrial food chain	2017	Latin America	https://www.nature.com/articles/s41598-017-14588-2	• First time evidence for micro and macroplastic transfer from soil to chickens	Highly critical	Highly critical	Highly critical	Highly critical	Highly critical	17	2, 3, 10, 14, 15	Strong	No	No	No	Strong	No	Strong	Strong
11	Ruimin et al.	Behavior of microplastics and plastic film residues in the soil environment: A critical review	2020	Global	https://www.sciencedirect.com/science/article/abs/pii/S0048969719347138?via%3Dihub	• Summarizing the distribution, indentifcation, effects and mitigation of microplastic in soils	Critical	Critical	Neutral	Highly critical	Critical	2, 3, 6, 14	11, 15	Strong	No	No	No	Moderate	No	Strong	Strong
12	Chen et al.	Effects of plastic film combined with straw mulch on grain yield and water use efficiency of winter wheat in Loess Plateau	2015	Asia	https://www.sciencedirect.com/science/article/abs/pii/S0378429014003323?via%3Dihub	• Investigate the effects of plastic film combined straw mulch on the grain yield of winter wheat	Beneficial	Beneficial	Highly beneficial	N/A	N/A	2, 6, 15, 17	11	No	No	No	No	No	No	No	No
13	Hu et al.	Comparative analysis of carbon footprint between conventional smallholder operation and innovative largescale farming of urban agriculture in Beijing, China	2021	Asia	https://peerj.com/articles/11632/	• Comparison of Carbon Footprint between largescale agriculture and conventional smallholder operation • Plastic contributes to the lion share of CF in both the cases which brings the need of designing materials that can have a longer use phase and sustainable recycling/degradation	Critical	Critical	Critical	Highly critical	Neutral	2, 9, 11, 12, 13, 17	8, 10, 14	Moderate	No	No	No	Strong	Strong	Strong	Strong
14	Gao et al.	Effects of plastic mulching and plastic residue on agricultural production: A meta-analysis	2019	Asia	https://www.sciencedirect.com/science/article/abs/pii/S0048969718335472?via%3Dihub	• Meta analysis on effects of plastic film and residual plastic on yield and waste use • The crop yield reduced with the increase in residual amount of plastic film in the soil	Beneficial	Beneficial	Neutral	Highly critical	Neutral	2, 17	10, 11, 12, 14, 15	Moderate	No	No	No	Strong	Moderate	Strong	Strong
15	Brodhagen et al.	Policy considerations for limiting unintended residual plastic in agricultural soils	2017	Global	https://www.sciencedirect.com/science/article/abs/pii/S1462901116307249?via%3Dihub	• Policies are needed to mitigate uncertaintly of biodegradable plastic in soils • Existing standards for certifying biodegradability are analysed	Beneficial	N/A	Neutral	Highly critical	Neutral	2, 3	6, 12, 13, 14, 15, 17	Moderate	No	No	No	Moderate	No	Moderate	Strong
16	Stubenrauch et al.	Plastic Pollution in Soils: Governance Approaches to Foster Soil Health and Closed Nutrient Cycles	2020	Europe	https://www.mdpi.com/2076-3298/7/5/38	• Qualitative governance analysis assessing effectiveness of policy instruments to avoid plastic pollution • Economic policy instruments aiming at a phasing-out of fossil fuels will also effectively reduce the total plastic	Critical	Critical	Highly critical	Highly critical	Critical	2, 3, 14	6, 10, 11, 12, 13, 15, 16, 17	Moderate	No	No	No	Moderate	Moderate	Strong	Strong
17	Edet et al.	Impact of "sachet water" microplastic on agricultural soil physicochemistry, antibiotics resistance, bacteria diversity and function	2022	Africa	https://link.springer.com/article/10.1007/s42452-022-05206-6	• Evaluation of potential impacts of microplastics on soil in Nigeria • Soil samples incubated with microplastic showed reduction in microbial diversity	N/A	N/A	Critical	Highly critical	N/A	2, 6	10, 11, 15	Strong	No	No	No	Moderate	Moderate	Strong	Strong
18	Sun et al	Contamination of phthalate esters, organochlorine pesticides and polybrominated diphenyl ethers in agricultural soils from the Yangtze River Delta of China	2016	Asia	https://www.sciencedirect.com/science/article/pii/S004896971531694?via%3Dihub	• Phthalate Esters used as plasticizer in polymers enters the soils through use of plastics in agriculture • Agricultural plastic films found as a major source of PAEs	Highly critical	Highly critical	Neutral	Highly critical	Critical	2, 8, 11, 17	14, 15	Strong	No	No	No	Moderate	No	No	No
19	Xucheng et al.	Did plastic mulching constantly increase crop yield but decrease soil water in a semiarid rain-fed area?	2020	Asia	https://www.sciencedirect.com/science/article/abs/pii/S0378377420302845?via%3Dihub	• Evaluation of plastic mulching on soil water balance in maize and wheat fields • Sustainabilit of mulching for crop production	N/A	N/A	Beneficial	Critical	N/A	2, 8	6, 14, 15	Moderate	No	No	No	No	No	Moderate	Strong
20	Cozzolino et al.	Appraisal of biodegradable mulching films and vegetal-derived biostimulant application as eco-sustainable practices for enhancing lettuce crop performance and nutritive value	2020	Europe	https://www.mdpi.com/2073-4395/10/3/427	• Comparison of the perforance of biodegradable plastic mulch with the normal PM from LDPE	Neutral	N/A	N/A	Beneficial	N/A	2, 8, 12	13, 14, 15	Strong	No	No	No	Moderate	No	Strong	Strong
Goal 3. Ensure healthy lives and promote well-being for all at all ages	Keywords: Health, Death, Illness, Disease, Mortality																				
1	Metcalf et al.	Quantifying the importance of plastic pollution for the dissemination of human pathogens: The challenges of choosing an appropriate 'control' material	2021	Global	https://www.sciencedirect.com/science/article/pii/S004896972107368X?via%3Dihub	• Review of all published studies that detected human pathogenic bacteria on surface of plastic pollution • With the increase in plastic pollution, microplastics rise increasing the surface of human pathogens	Highly critical	Highly critical	Critical	Highly critical	Highly critical	3, 12	6, 10, 11, 14, 15, 16	Strong	No	No	No	Strong	No	Strong	Strong
2	Bouwmeester et al.	Potential Health Impact of Environmentally Released Micro- and Nanoplastics in the Human Food Production Chain: Experiences from Nanotoxicology	2015	Global	https://pubs.acs.org/doi/10.1021/acs.est.5b01090	• Review on the occurence of environmentally released micro and nanoplastics in the human food production chain and their potential health impact • Toxicity of the particles, POPs from the plastic particles and the leaching of additives are some of the paths which results in the microplastics enter food chain	Highly critical	Highly critical	Highly critical	Highly critical	Critical	3, 14	6, 10, 11, 12, 15	Strong	No	No	No	Strong	No	Strong	Moderate
3	Pramod Kumar	Role of Plastics on Human Health	2018	Asia	https://link.springer.com/article/10.1007/s12098-017-2595-7	• Reviews literature about health effects of plastics in humans • Effects of different plastic additives lie phthalates, Bisphenol • Recommendations to reduce the exposure and alternatives to plastics	Critical	Highly critical	Highly critical	Critical	N/A	3	5, 10, 12, 14, 15	Strong	Moderate	Moderate	No	No	No	No	Strong
4	Li et al.	Environmental risks of disposable face masks during the pandemic of COVID-19: Challenges and management	2022	Asia	https://www.sciencedirect.com/science/article/pii/S004896972200972X?via%3Dihub	• Environmental risks of disposable face msks during COVID pandemic • Concentration fo the FM leachates and the fate of the FMs should be analysed by the governments in detail	N/A	Highly critical	Beneficial	Highly critical	Highly critical	3, 12	6, 9, 10, 11, 13, 14, 15	Strong	No	No	No	No	No	Strong	Strong
5	Yuan et al.	Human health concerns regarding microplastics in the aquatic environment - From marine to food systems	2022	Global	https://www.sciencedirect.com/science/article/pii/S004896972200822 1?via%3Dihub	• Review on the definition and chatacterisation of microplastics in the marine ecosystem	Highly critical	Highly critical	N/A	Highly critical	Highly critical	3, 14	6, 10, 11, 15, 16, 17	Strong	Moderate	No	No	Moderate	No	Strong	Moderate
6	Landrigan et al.	Human health and ocean pollution	2020	Global	https://annalsofglobalhealth.org/article/10.5334/aogh.2831/	• Examine the known impacts of ocean pollution on human health • Propose interventions and inform policy makers to control/prevent the pollution	Highly critical	Highly critical	N/A	Highly critical	Critical	2,3, 6,7,8,11,12,13,14, 17	10, 15	Strong	Moderate	Moderate	Moderate	No	No	Strong	Strong
7	Groh et al.	Overview of known plastic packaging-associated chemicals and their hazards	2019	Global	https://www.sciencedirect.com/science/article/pii/S004896971833828?pes=vor	• Compilaton of list of chemicals associated with Plastic packaging • Numerous data gaps exist with regards to the use patterns and exposure • Identified the most hazardous substances in packaging and its hazard information	Critical	Highly critical	N/A	Highly critical	N/A	3	5, 6, 10, 11, 12 14, 15, 17	No	No	Moderate	No	No	No	No	Moderate
8	Yee et al.	Impact of microplastics and nanoplastics on human health	2021	Global	https://www.mdpi.com/2079-4991/11/2/496	• Behaviour and breakdown of NP and their possible health impacts	Highly critical	Highly critical	Highly critical	Highly critical	N/A	3, 14	5, 10, 11, 12, 17	Moderate	No	No	No	Moderate	No	Moderate	Unclear
9	Leslie et al.	Discovery and quantification of plastic particle pollution in human blood	2022	Europe	https://www.sciencedirect.com/science/article/pii/S016041202201258	• A method was validated for polymer mass concentrations in human whole blood • Polymers in human blood represent several high production volume plastics	Highly critical	Highly critical	N/A	Highly critical	N/A	3	5, 10, 11, 12, 14, 15, 17	Strong	No	No	No	No	No	Strong	Moderate
10	Jiang et al.	Health impacts of environmental contamination of micro- And nanoplastics: A review	2020	Global	https://link.springer.com/content/pdf/10.1186/s12199-020-00870-9.pdf	• Transport and bioaccumulation of MNPs and exposure in Human and animals are studied • Multiple experimental models are needed to study the effects of MNPs	Highly critical	Highly critical	N/A	Highly critical	N/A	3, 14	5, 10, 11, 12, 14, 15, 17	Strong	No	No	No	No	No	Strong	Moderate
11	Adebiyi-Abiola et al.	Cleaning up plastic pollution in Africa	2019	Africa	https://www.science.org/doi/10.1126/science.aax3539	• Plastic waste management and initiatives to prevent plastic pollution in African countries	Critical	N/A	Neutral	Critical	N/A		3, 4, 8, 9, 10, 11, 12, 13, 14, 15, 17	Moderate	Strong	Moderate	No	No	No	Strong	Strong
12	Yang et al.	Waste management, informal recycling, environmental pollution and public health	2017	Asia	https://jech.bmj.com/content/72/3/237	• Effective integration of informal sector in waste collection is needed • Collaboration between different stakeholders essential	Critical	Critical	Critical	Highly critical	Critical	1, 3, 10, 12, 17	5, 8, 9, 11, 14, 15	Moderate	No	No	No	No	Moderate	Strong	Strong
13	Hahladakis et al.	An overview of chemical additives present in plastics: Migration, release, fate and environmental impact during their use, disposal and recycling	2018	Global	https://www.sciencedirect.com/science/article/pii/S030438941730763X?pes=vor	• Contamination of additives during use, disposal and recycling of plastics	Highly critical	Highly critical	Critical	Highly critical	N/A	3, 9, 12	1, 2, 5, 6, 14, 15, 17	Strong	Strong	Moderate	No	No	Moderate	Strong	Strong
14	Gasperi et al.	Microplastics in air: Are we breathing it in?	2018	Global	https://www.sciencedirect.com/science/article/abs/pii/S246858441730011 9?via%3Dihub	• Review on the occurence of MPs in the atmosphere and the subsequent health risks on humans	Critical	N/A	Highly critical	Highly critical	N/A	3	10, 11, 12, 15, 17	Strong	Strong	No	No	No	No	Strong	Moderate

15	Barboza et al.	Marine microplastic debris: An emerging issue for food security, food safety and human health	2018	Global	https://www.sciencedirect.com/science/article/pii/S0025326X183 0376X?pes=vor	• Reviewed evidence of seafood contamination by MPs<br>• Assessment of risk posed to humans is challenging	Critical	N/A	Critical	Highly critical	N/A	2, 14	1, 3, 6, 9, 11, 12, 15, 17	Strong	No	No	No	Moderate	No	Strong	Moderate
16	Mason et al.	Synthetic Polymer Contamination in Bottled Water	2018	Global	https://www.frontiersin.org/articles/10.3389/fchem.2018.00407/f ull	• Contamination of water with microplastics when bottling it<br>• It was found that 93% of the time the analysed water bottles had an average MP contamination	Highly critical	Highly critical	N/A	Highly critical	N/A	3, 6	2, 9, 10, 11, 12, 14, 15	Strong	Strong	Strong	No	No	Moderate	No	Strong
17	Wright et al.	Plastic and Human Health: A Micro Issue?	2017	Global	https://pubs.acs.org/doi/10.1021/acs.est.7b00423	• Review of the potential helath impacts of plastics on human health<br>• Apart from being present in seafood, traces of MP/NPs found in sugar and honey	Highly critical	Highly critical	N/A	Highly critical	N/A	3	2, 9, 11, 12, 14, 15	Strong	Strong	No	No	Moderate	No	Strong	Strong
18	Fred-Ahmadu et al.	Interaction of chemical contaminants with microplastics: Principles and perspectives	2020	Global	https://www.sciencedirect.com/science/article/abs/pii/S00489697 1935973X?via%3Dihub	• Review of plastic additives and their impacts<br>• Factors that influence the sorption capacity of plastics	Highly critical	Highly critical	N/A	Highly critical	N/A	3, 14	2, 6, 11, 12, 15	Strong	Strong	No	No	Moderate	No	Strong	Strong
19	Velis et al.	Mismanagement of Plastic Waste through Open Burning with Emphasis on the Global South: A Systematic Review of Risks to Occupational and Public Health	2021	Global	https://pubs.acs.org/doi/10.1021/acs.est.0c08536	• Review of studies on the effects of open burning of plastic wastes	Highly critical	Highly critical	N/A	Highly critical	N/A	3, 11, 12, 17	2, 6, 7, 8, 9, 11, 13	Strong	No	No	No	No	Moderate	Strong	Strong
20	Tang et al.	Polybrominated diphenyl ethers (PBDEs) and heavy metals in road dusts from a plastic waste recycling area in north China: implications for human health	2016	Asia	https://link.springer.com/article/10.1007/s11356-015-5296-7?utm_source=getftr&utm_medium=getftr&utm_campaign=getftr_pi lot	• Assessment of road dust from the reycling site for possible diphenyl ether pollution	Highly critical	Highly critical	N/A	Highly critical	Critical	3, 12	2, 8, 9, 11, 13, 14, 15	Strong	No	Moderate	No	Moderate	Moderate	Strong	Strong
**Goal 4. Ensure inclusive and equitable quality education and promote lifelong learning opportunities for all**	**Keywords: Education, Skills, Qualification**																				
1	Chmielewski et al.	Microplastic in the environment. The role of education in raising social awareness of the handling of plastic waste	2021	Global	http://jsite.uwm.edu.pl/articles/view/2215/	• Negative effects of exposure of MPs on human health<br>• Necessity to raise social awarness in handling plastic waste	Highly critical	Highly critical	Neutral	Neutral	Critical	3	4, 9, 11, 12, 13, 14, 15, 17	Moderate	No	Strong	Moderate	Moderate	Moderate	Strong	Strong
2	Mironenko et al.	Education Against Plastic Pollution: Current Approaches and Best Practices	2020	Global	https://link.springer.com/chapter/10.1007/698_2020_486	• Review the educational initiatives to see how informal education reacted to the plastic problem<br>• To check if there is anything mising from the big picture of education as a tool	Critical	Critical	Neutral	Neutral	N/A	3, 4, 12,14	6, 9, 11, 13, 15, 17	Moderate	Unclear	No	No	Moderate	Moderate	Strong	Strong
3	Bennett et al.	Informing the public and educating students on plastic recycling	2021	North America	https://www.mdpi.com/2313-4321/6/4/69	• Identify the resources needed to build university courses and review the education material for recycling	Highly critical	Highly critical	Neutral	Neutral	N/A	4, 12	9, 11, 13, 14, 15, 17	No	Unclear	Strong	No	Moderate	Moderate	Strong	Strong
4	Dalu et al.	Is awareness on plastic pollution being raised in schools? Understanding perceptions of primary and secondary school educators	2020	Africa	https://www.mdpi.com/2071-1050/12/17/6775	• Investigate whether plastic pollution integrated into school curriculums	Critical	N/A	Neutral	Critical	N/A	4, 12, 14, 17	8, 9, 11, 13, 15	No	Moderate	No	No	No	Moderate	Moderate	Moderate
5	Kowasch et al.	Circular economy, cradle to cradle and zero waste frameworks in teacher education for sustainability	2022	Europe	https://www.emerald.com/insight/content/doi/10.1108/IJSHE-10-2021-0428/full/html	• How alternative concepts like Circular Economy can contribute to the sustainability education	Critical	N/A	Neutral	Neutral	N/A	4, 8, 12	10, 11, 13, 14, 15, 17	No	Moderate	No	No	No	Moderate	Strong	Strong
6	Jimenez et al.	'It's my responsibility': perspectives on environmental justice and education for sustainability among international school students in Singapore	2021	Asia	https://www.tandfonline.com/doi/full/10.1080/09620214.2020.18 56000	• Analyzes student's perspective on sustainable development<br>• Students had an understandng of resource consumption and their privilege to access the resources	N/A	N/A	Beneficial	Critical	N/A	4, 8, 9, 12, 13, 17	5, 10, 11, 14, 15	Moderate	Moderate	No	No	No	Moderate	No	Moderate
7	Situmorang et al.	The difference of knowledge and behavior of college students on plastic waste problems	2020	Asia	https://www.mdpi.com/2071-1050/12/19/7851	• Studied the differences in the knowledge on negative effects of plastic among students of different streams	Critical	N/A	Neutral	Highly critical	N/A	4, 12	8, 13, 14, 15, 17	Moderate	No	No	No	No	No	Strong	Moderate
8	Hammami et al.	Survey on awareness and attitudes of secondary school students regarding plastic pollution: implications for environmental education and public health in Sharjah city, UAE	2017	Asia	https://link.springer.com/article/10.1007/s11356-017-9625-x	• Assess knowledge and attitude about plastic pollution among secondary school students	Critical	Critical	Highly critical	Highly critical	N/A	3, 4, 11, 12	6, 8, 9, 13, 14, 15, 17	Moderate	Moderate	No	No	No	No	Strong	Strong
9	Parejo et al.	Plastics as an educational resource for sustainable development: A case study in Ghana	2021	Africa	https://www.mdpi.com/2071-1050/13/12/6727	• Identifying new ways to recycle plastic and imparting education to the children about usage of plastic	Highly critical	Critical	Neutral	Highly critical	Critical	4, 12, 17	1, 6, 8, 9, 10, 11, 13, 14, 15	Strong	Strong	No	No	No	Moderate	Strong	Strong
10	Phan Hoang et al.	Measuring the effect of environmental education for sustainable development at elementary schools: A case study in Da Nang city, Vietnam	2016	Asia	https://www.sciencedirect.com/science/article/pii/S24682039163 01303?via%3Dihub	• Description of the environmental education given to the elementary school in Vietnam thereby increasing the awareness about solid waste management	Critical	N/A	N/A	Critical	N/A	4, 8, 11, 12	13, 14, 15, 17	Strong	Moderate	No	No	No	Moderate	Strong	Strong
11	Khanam et al.	Knowledge, attitude and practice on uses of plastic products, their disposal and environmental pollution: A study among school-going adolescents	2019	Asia	http://www.journaldmims.com/article.asp?issn=0974-3901;year=2019;volume=14;issue=2;spage=57;epage=60;aulast=Kh anam	• Purposive sampling on studying the use and practice of plastic products among school adoloscents	Highly critical	N/A	Highly critical	Highly critical	N/A	3, 4, 11	9, 12, 13, 17	Moderate	No	No	No	No	Moderate	Strong	Moderate
12	Barra et al.	Sustainable chemistry challenges from a developing country perspective: Education, plastic pollution, and beyond	2018	South America	https://www.sciencedirect.com/science/article/abs/pii/S24522236 17300524?via%3Dihub	• Challenges of implementing sustainable chemistry in Latin America to prevent plastic pollution	Highly critical	N/A	N/A	Highly critical	N/A	4, 8, 12, 14, 17	9, 11, 13, 15	Strong	No	Moderate	No	No	Moderate	Strong	Strong
13	So et al.	Environmental education in primary schools: A case study with plastic resources and recycling	2018	Asia	https://www.tandfonline.com/doi/full/10.1080/03004279.2018.15 18336	• Challenges of implementing plastic education in Hongkong based on the survey of case studies with teachers	Critical	N/A	Critical	Critical	Critical	4, 12	8, 9, 11, 13	Moderate	No	No	No	No	Moderate	Strong	Strong
14	Cheung et al.	A train-the-trainer design for green ambassadors in an environmental education programme on plastic waste recycling	2018	Asia	https://www.tandfonline.com/doi/full/10.1080/10382046.2017.12 85138	• Understand the usefulness and feasibility of train the trainer approach in Hongkong where students become green ambassadors	Highly critical	Critical	Neutral	Neutral	N/A	4, 12	6, 8, 9, 11, 14, 15, 17	No	Moderate	No	No	No	No	Strong	Moderate
15	Chow et al.	Plastic waste problem and education for plastic waste management	2017	Asia	https://link.springer.com/chapter/10.1007/978-981-10-3344-5_8	• Effective teaching strategies for children about plastic waste management	Critical	Critical	Neutral	Critical	N/A	8, 12	4, 9, 11, 14, 15, 17	Moderate	No	No	No	No	No	Moderate	Moderate
16	Soares et al.	On the path to minimize plastic pollution: The perceived importance of education and knowledge dissemination strategies	2021	Europe	https://www.sciencedirect.com/science/article/abs/pii/S0025326X 21009243?via%3Dihub	• Describing perceived factors affecting environmental awareness among people on plastic pollution	Highly critical	N/A	Critical	Highly critical	N/A	4, 12	5, 6, 8, 11, 14, 15, 17	No	No	No	No	No	Moderate	Moderate	Strong
17	Mandrikas et al.	In-service Teachers' Needs and Mentor's Practices in Applying a Teaching–Learning Sequence on Nanotechnology and Plastics in Primary Education	2021	Europe	https://link.springer.com/article/10.1007/s10956-021-09908-1?utm_source=getftr&utm_medium=getftr&utm_campaign=getftr_pi lot	• Needs of teachers when implementing Teaching-Learning sequence on cutting edge topics like nanotechnology or plastics in oceans	N/A	N/A	N/A	Critical	N/A	14	4, 12, 17	No	No	No	No	No	No	Moderate	No
18	Lopez-Fernandez et al.	How Can Socio-scientific Issues Help Develop Critical Thinking in Chemistry Education? A Reflection on the Problem of Plastics	2022	Europe	https://pubs.acs.org/doi/10.1021/acs.jchemed.2c00223	• How social scientific issues like plastic pollution can help critical thinking among students in chemistry?<br>• Evaluation of 8-grade students on thir critical thinking	Critical	N/A	Neutral	Critical	N/A		4, 12, 17	Moderate	No	No	No	No	No	Moderate	Moderate
19	du Bois et al.	Design Against the Plastic Soup - The Effect of Small Product Designs in Sustainable Design Education	2019	Europe	https://www.cambridge.org/core/journals/proceedings-of-the-international-conference-on-engineering-design/article/design-against-the-plastic-soup-the-effect-of-small-product-designs-in-sustainable-design-education/13E4F135C5F60186F4EB5C8C957897A4	• Design students explore the opportunities to deal with the plastic problem	Critical	Critical	Critical	Highly critical	Critical	4, 7, 9, 12, 14	11, 13, 17	Moderate	Moderate	Strong	Moderate	No	Moderate	Strong	Strong
20	Schiffer et al.	Microplastics Outreach Program: A Systems-Thinking Approach to Teach High School Students about the Chemistry and Impacts of Plastics	2020	North America	https://pubs.acs.org/doi/10.1021/acs.jchemed.9b00249	• Discuss the curriculum of an outreach program that discuss about the plastic pollution	Critical	Critical	N/A	Critical	N/A	4, 14	12, 13, 15	Moderate	No	Moderate	No	No	Moderate	Strong	Moderate
**Goal 5. Achieve gender equality and empower all women and girls**	**Keywords: Gender, Women, Equality, Policy**																				
1	De La Pena et al.	Empowering Women Thru Plastics Recycling: A Livelihood Project for Women in Marginalized Communities	2018	Asia	https://ieeexplore.ieee.org/abstract/document/8601555	• Discussion of the challenges and methods of the recycling program in Manila that empowers women and provide them with income	Critical	N/A	N/A	Beneficial	N/A	4, 5, 9, 10, 11, 16, 17	1, 8, 12	No	No	No	Moderate	No	No	Moderate	Moderate
2	Braun et al.	Plastic Bags, Pollution, and Identity: Women and the Gendering of Globalization and Environmental Responsibility in Mali	2015	Africa	https://journals.sagepub.com/doi/10.1177/0891243215602101	• Interview with women in the markets of Mali about plastic bag pollution	Critical	N/A	Neutral	Highly critical	Critical	8, 17	1, 2, 4, 5, 6, 10, 11, 12	Moderate	No	No	No	No	No	Strong	Strong
3	Chatterjee et al.	Green chemistry – Remedy to societal hygiene: A graphical review	2020	Asia	https://www.sciencedirect.com/science/article/pii/S26660865203 0028X?via%3Dihub	• Graphical review on how green chemistry can bring a healthy and green society<br>• Synopsis of existing menstrual hygiene needs and challenges	Critical	N/A	Critical	Critical	N/A	11, 12, 17	1, 4, 5, 7, 8	Moderate	No	No	No	No	No	Strong	Moderate
4	Anne Marie Hanson	Women's environmental health activism around waste and plastic pollution in the coastal wetlands of Yucatán	2017	South America	https://www.tandfonline.com/doi/full/10.1080/13552074.2017.13 35450	• Women's grassroots organisations role in confronting waste pollution	Highly critical	N/A	Critical	Highly critical	N/A	3, 4, 11, 12, 14, 15	1, 5, 10, 17	Strong	Moderate	No	No	No	No	Strong	Moderate
5	Li et al.	Is Female a More Pro-Environmental Gender? Evidence from China	2022	Asia	https://doi.org/10.3390/ijerph19138002	• Gender does affect green psychology and behaviors, with females reporting a higher level of environmentalism in China	Critical	N/A	Critical	Critical	N/A	5	4, 8, 11, 12	Moderate	No	No	No	No	Strong	Strong	Strong
6	Lange et al.	Understanding the Socio-Demographic Profile of Waste Re-Users in a Suburban Setting in South Africa	2022	Africa	https://www.mdpi.com/2079-9276/11/5/45	• Understand the socio-demographic behaviour of waste reusers and the influencing factors	Critical	N/A	Critical	Critical	N/A	4, 11, 12, 17	5, 10, 13	No	Moderate	No	No	No	Strong	Strong	Moderate
7	Chukwuone et al.	Determinants of household's waste disposal practices and willingness to participate in reducing the flow of plastics into the ocean: Evidence from coastal city of Lagos Nigeria	2022	Africa	https://journals.plos.org/plosone/article?id=10.1371/journal.pone .0267739	• Provides waste management information to reduce illegal waste disposal<br>• Survey to understand the socio-demographic information on waste disposal in Lagos	Critical	Critical	Highly critical	Highly critical	Critical	11, 12, 14, 17	5, 6, 8, 9, 13, 15	Strong	Moderate	No	No	No	Moderate	Strong	Strong
8	Robert Grace	Overcoming Gender Bias in Design	2021	Global	https://onlinelibrary.wiley.com/doi/epdf/10.1002/peng.20521?src =getftr	• How Gender bias influence the design thinking in industrial design	N/A	N/A	Critical	Highly critical	N/A	5	6, 8, 9, 10, 12	No	No	No	No	Moderate	Moderate	No	Moderate
9	Muralidharan et al.	The Role of Guilt in Influencing Sustainable Pro-Environmental Behaviors among Shoppers	2018	Europe	https://www.journalofadvertisingresearch.com/content/58/3/349	• Discuss about how guilt is perceived by male and femal to unfluence sutainable behaviour	Critical	Critical	Highly critical	Critical	N/A	5	4, 6, 9, 11, 14, 15	Moderate	Moderate	No	Moderate	No	Strong	No	Strong
10	Mikołajewska et al.	Bisphenol A – Application, sources of exposure and potential risks in infants, children and pregnant women	2015	Global	http://ijomeh.eu/Bisphenol-a-application-sources-of-exposure-and-potential-risks-in-infants-children-and-pregnant-women,1991,0,2.html	• Potential sources of Bisphenol A and its adverse health effects	Critical	Highly critical	Critical	Highly critical	Critical	9	2, 5, 6, 8, 12, 14, 15, 17	Strong	Strong	No	No	No	No	Strong	Strong
11	Gao et al.	Feminine Hygiene Products - A Neglected Source of Phthalate Exposure in Women	2020	Asia	https://pubs.acs.org/doi/10.1021/acs.est.9b03927	• Different types of Phthalates found in women sanitary products and characterised	Critical	Highly critical	Critical	N/A	N/A	9, 17	5, 8, 11, 12	Strong	Strong	No	No	No	No	Moderate	Strong
12	Valvi et al.	Variability and predictors of urinary phthalate metabolites in Spanish pregnant women	2015	Europe	https://www.sciencedirect.com/science/article/pii/S14384639140 01163?via%3Dihub	• Influencing factors that determine the concentration of phthalates in the urine	Critical	Highly critical	Highly critical	N/A	N/A	6	5, 10, 11, 12	Strong	Strong	No	No	No	No	No	Moderate
13	Anne Marie Hanson	Women's ecological oral histories of recycling and development in coastal Yucatán	2016	South America	https://www.tandfonline.com/doi/full/10.1080/0966369X.2015.10 13445	• Strategic importance of oral histories from women on the prevention of pollution. Focus on the work of removing unwanted garbage and protect coastal landscape	Highly critical	N/A	Critical	Highly critical	N/A	8, 11, 12, 14, 17	1, 5, 10	Strong	Moderate	No	No	No	No	Strong	Moderate
14	Norfaryanti et al.	Environmental-friendly food products' packaging: Women's purchasing preferences	2019	Asia	https://www.ijrte.org/wp-content/uploads/papers/v8i2S4/B11010782S419.pdf	• Purchase preferences of women regarding ecofriendly packaging	Highly critical	N/A	Highly critical	Highly critical	Critical	14, 17	5, 6, 8, 11, 12, 15	Strong	Moderate	No	Moderate	No	Moderate	Strong	Moderate
15	Bula Sirika Wayessa	Prepared in pots, served in plastics: Rural Ethiopian women's responses to the global economy	2020	Africa	https://journals.sagepub.com/doi/10.1177/1466138120946774	• Decline of the traditional pottery due to the imported plastic products	Critical	Critical	Highly critical	N/A	Critical	17	1, 5, 8, 9, 12	No	No	No	No	No	No	No	Strong
16	Muposhi et al.	Is the use of green shopping bags gendered? Evidence from a gender equality conscious emerging market	2022	Africa	http://www.inderscience.com/storage/f211012785693114.pdf	• Gender differences in the use of green shopping bags and attitude towards pro-environmental attitudes	Highly critical	Critical	Critical	Critical	N/A	5, 10, 12, 17	8, 9, 14, 15	Moderate	Strong	No	No	No	Moderate	Strong	Strong
17	Alharbi et al.	Use of Plastics with Hot Food among Saudi Pregnant Women Is Associated with Increased Concentrations of A1C, Thyroid-Stimulating Hormone, and Homocysteine and Decreased Concentrations of Vitamins and Minerals	2020	Asia	https://www.mdpi.com/2072-6643/12/9/2609	• Analyisis of data regarding Saudi pregnant women and their frequency of plastic use to look for health complications	Highly critical	Highly critical	Highly critical	N/A	N/A		5, 10, 12	Strong	No	No	No	Strong	No	No	Moderate
18	Chowdhury et al.	WASH and MHM experiences of disabled females living in Dhaka slums of Bangladesh	2022	Asia	https://iwaponline.com/washdev/article/12/10/683/91438/WASH-and-MHM-experiences-of-disabled-females	• Interviews with disabled females living in Slums on the access to WASH facilities	Critical	N/A	Neutral	Critical	N/A	5, 6, 10, 17	8, 9, 11, 12, 14	No	No	No	No	No	No	Strong	Moderate
19	Anusha et al.	Ready-to-eat food consumption practices, food safety knowledge and relation to gender and education level of consumers in Kuala Lumpur, Malaysia	2020	Asia	https://www.myfoodresearch.com/uploads/8/4/8/5/84855864/_19__fr-2020-037_anusha.pdf	• Consumer's consumption patterns on ready to eat foods	N/A	N/A	Beneficial	N/A	N/A		3, 6, 10, 11, 12	No	No	No	No	No	Strong	No	No
20	Trowbridge et al.	Fossil fuel is the common denominator between climate change and petrochemical exposures, and effects on women and children's health	2022	Global	https://obgyn.onlinelibrary.wiley.com/doi/10.1002/ijgo.14408	• Link between climate change and industrial production of products from fossil based resources	Highly critical	Highly critical	Critical	Highly critical	Critical	13	5, 6, 12, 14, 15, 17	Strong	Moderate	No	No	No	No	Strong	Moderate
**Goal 6. Ensure availability and sustainable management of water and sanitation for all**	**Keywords: Water, Sanitation, Drinking, Scarcity**																				
1	Mihai et al.	Plastic Pollution, Waste Management Issues, and Circular Economy Opportunities in Rural Communities	2022	Global	https://www.mdpi.com/2071-1050/14/1/20	• Effects of plastic pollution on rural communities	Highly critical	Highly critical	Critical	Highly critical	Critical	3, 8, 9, 12, 15, 17	1, 5, 10, 11, 14	Strong	Strong	Moderate	Moderate	Moderate	Moderate	Strong	Strong
2	Yeasmin et al.	Piloting a low-cost hardware intervention to reduce improper disposal of solid waste in communal toilets in low-income settlements in Dhaka, Bangladesh	2017	Asia	https://bmcpublichealth.biomedcentral.com/articles/10.1186/s12 889-017-4693-x	• Factors contributing to the improper disposal of solid wastes in the low income settlements	N/A	N/A	Beneficial	Neutral	N/A	1, 6, 11, 12	3, 4, 5, 9, 10, 14, 17	No	No	No	No	No	Moderate	Strong	Strong
3	Geetha T	Endocrine disruptors in boiled drinking water carried in plastic containers: a pilot study in Thrissur, Kerala, India	2021	Asia	https://link.springer.com/article/10.1007/s13201-021-01524-z	• Survey of plastic bottles carrying boiled water for phthalates and other chemicals	Critical	Critical	Highly critical	N/A	N/A	3, 4, 6, 17	10, 11, 12	Strong	No	No	No	No	No	No	Moderate
4	Liu et al.	Do estrogenic compounds in drinking water migrating from plastic pipe distribution system pose adverse effects to human? An analysis of scientific literature	2017	Asia	https://link.springer.com/article/10.1007/s11356-016-8032-z	• Evaluation of health risks associated with the water transported in pipes	Critical	Highly critical	Highly critical	N/A	Highly critical	6	10, 11, 12	Strong	No	No	No	No	No	No	Moderate
5	Manjaya et al.	Informally vended sachet water: Handling practices and microbial water quality	2019	Africa	https://www.mdpi.com/2073-4441/11/4/800	• Analysis of microbial concentration of the drinking water sold in packets in Malawi	Highly critical	Critical	Highly critical	N/A	N/A	6, 17	1, 3, 4, 10, 14	Strong	No	No	No	Moderate	No	No	Moderate
6	Barletta et al.	Distribution, sources and consequences of nutrients, persistent organic pollutants, metals and microplastics in South American estuaries	2019	South America	https://www.sciencedirect.com/science/article/abs/pii/S00489697 18337264?via%3Dihub	• Conservation of south american estuaries against plastic pollution	Highly critical	Highly critical	Critical	Highly critical	Critical	6, 11, 14, 15, 17	1, 4, 5, 10, 12, 13	Strong	Strong	No	No	No	No	Strong	Strong
7	Haris et al.	Installing public handwashing facilities and integrating them with water fountains to reduce plastic pollution and prevent spread of infections	2021	Europe	https://journals.sagepub.com/doi/10.1177/17579139211002221	• Explore the extent of how handwashing can prevent plastic pollution so that santiser dispenser dont end up in landfills	Critical	N/A	Critical	Highly critical	N/A	6	4, 5, 9, 11, 12	No	Moderate	No	No	No	No	Strong	Strong
8	Dorji et al.	On-site domestic wastewater treatment system using shredded waste plastic bottles as biofilter media: Pilot-scale study on effluent standards in Bhutan	2022	Asia	https://www.sciencedirect.com/science/article/abs/pii/S00456535 21022013?via%3Dihub	• Analysis of using shredded plastics as a biofilter for effluent treatment plant	Highly critical	Highly critical	N/A	Neutral	N/A	6, 11, 17	8, 9, 12	No	No	No	No	No	Moderate	Moderate	Moderate
9	Szczotko et al.	Evaluation of susceptibility of polymer and rubber materials intended into contact with drinking water on biofilm formation	2016	Europe	https://pubmed.ncbi.nlm.nih.gov/27925711/	• Evaluate the susceptibility of biofilm formation when water comes in contact with polymer and rubber materials	N/A	Critical	N/A	N/A	N/A	3, 6	5, 9	No	Moderate	No	No	No	No	No	No
10	Alda-Vidal et al.	"Unflushables": Establishing a global agenda for action on everyday practices associated with sewer blockages, water quality, and plastic pollution	2020	Global	https://wires.onlinelibrary.wiley.com/doi/10.1002/wat2.1452	• Review on different factors of household practices to understand challenges of unflushables in toilets	Critical	Critical	Critical	Highly critical	N/A	6, 9, 17	4, 5, 11, 12, 14, 15	Moderate	No	No	No	No	No	Strong	Moderate
11	Egun et al.	Beat the plastic: an approach to polyethylene terephthalate (PET) bottle waste management in Nigeria	2020	Africa	https://link.springer.com/article/10.1007/s42768-020-00052-x	• Analyse the PET bottle system in Nigeria and emphasise the need for a policy in waste management	Highly critical	Critical	Highly critical	Highly critical	Critical	4, 6, 8, 11, 12, 15, 16, 17	1, 3, 9	Strong	Moderate	Moderate	Moderate	Moderate	Moderate	Strong	Strong

12	Winter et al.	Women's sanitation practices in informal settlements: A multi-level analysis of factors influencing utilisation in Nairobi, Kenya	2018	Africa	https://www.tandfonline.com/doi/full/10.1080/17441692.2018.1534256	• Factors that influence women's sanitation in an informal settlement in Nairobi, Kenya	N/A	N/A	Beneficial	N/A	N/A	6, 11, 17	1, 3, 9, 10, 12	No	No	No	No	No	No	Strong	No
13	O Briain et al.	The role of wet wipes and sanitary towels as a source of white microplastic fibres in the marine environment	2020	Europe	https://www.sciencedirect.com/science/article/pii/S0043135420305583?pes=vor	• Role of sewage related debris (wetwipes, towels) as a source of microplastic fibres in the enviroment	Highly critical	Critical	Critical	Highly critical	N/A	6, 9, 12, 14	3, 11, 15	Strong	Moderate	No	No	No	No	Strong	Moderate
14	Borde et al.	Community challenges when using large plastic bottles for Solar Energy Disinfection of Water (SODIS)	2016	Africa	https://bmcpublichealth.biomedcentral.com/articles/10.1186/s12889-016-3535-6	• Factors influencing the use and acceptance of solar disinfected water in large plastic containers against small plastic bottles	Neutral	Critical	Beneficial	N/A	N/A	1, 3, 6, 17	9, 11, 12	No	No	Strong	No	No	No	No	No
15	Diehl et al.	Do-it-Yourself (DIY) Workspaces Run by Local Entrepreneurs that Transform Plastic Waste into Valuable Water and Sanitation Products	2019	Asia	https://ieeexplore.ieee.org/document/8601880	• Transforming plastic waste into valuable water and sanitation products with IY approach	Critical	Critical	Critical	Beneficial	N/A	3, 6, 11, 12	1, 14, 15, 17	No	No	No	No	No	Strong	Strong	Strong
16	Necibi et al.	Contaminants of emerging concern in african wastewater effluents: Occurrence, impact and removal technologies	2021	Africa	https://www.mdpi.com/2071-1050/13/3/1125	• Review on the research efforts on the contaminants in wastewater effluents	Critical	N/A	N/A	Highly critical	N/A	3, 6, 11, 17	1, 12, 14, 15	Strong	No	No	No	No	No	Moderate	Strong
17	Taduswan et al.	Microplastic contamination in a conventional wastewater treatment plant in Thailand	2021	Asia	https://journals.sagepub.com/doi/10.1177/0734242X20982055	• Analysis of microplastics in WWTP in Thailand	Highly critical	Highly critical	N/A	Highly critical	N/A	6, 11, 17	3, 12, 14, 15	Strong	No	No	No	No	No	Strong	Strong
18	Muanda et al.	Factors and impacts of informal settlements residents' sanitation practices on access and sustainability of sanitation services in the policy context of free basic sanitation	2020	Africa	https://iwaponline.com/washdev/article/10/2/238/73957/Factors-and-impacts-of-informal-settlements	• Factors associated with the resident's sanitation practices in the context free basic sanitation practice in South Africa	N/A	N/A	Beneficial	N/A	N/A	6, 11	1, 3, 9, 10	No	No	No	No	No	No	Moderate	Moderate
19	Semey et al.	Characteristics of packaged water production facilities in greater Accra, Ghana: Implications for water safety and associated environmental impacts	2020	Africa	https://iwaponline.com/washdev/article/10/1/146/72131/Characteristics-of-packaged-water-production	• Assessment of water packed in sachets in Ghana	N/A	Critical	N/A	Critical	N/A	6, 11	1, 3, 9	No	No	No	No	No	No	Moderate	Moderate
20	Tang et al.	Different senescent HDPE pipe-risk: brief field investigation from source water to tap water in China (Changsha City)	2015	Asia	https://link.springer.com/article/10.1007/s11356-015-5275-z	• Investigation of pipe water from PE pipes	Critical	Highly critical	Neutral	Critical	N/A	6, 17	3, 12	No	Moderate	No	No	No	No	Moderate	No
**Goal 7. Ensure access to affordable, reliable, sustainable and modern energy for all**	**Keywords: Energy, Renewable, Sustainable, Clean**																				
1	Dong et al.	Design of special plastic bearings and their application in renewable energy conversion system	2015	Asia	https://openmaterialssciencejournal.com/VOLUME/9/PAGE/203/	• Use of plastic bearings in energy storage and conversion technologies	Beneficial	Beneficial	Highly beneficial	N/A	N/A	7	8, 9, 11, 12, 13	No	No	No	No	No	Moderate	Moderate	No
2	Stephen et al.	Completing the value chain for plastic recyclers in Nigeria: An integration of renewable solar and conventional gas energy sources for fuel production	2018	Africa	https://onepetro.org/SPENAIC/proceedings-abstract/18NAIC/All-18NAIC/SPE-193436-MS/215775	• Use of solar energy for the pyrolysis of plastic wastes as a fuel source	Critical	Critical	N/A	Beneficial	N/A	7, 12, 17	8, 9, 11, 13	No	No	Strong	No	No	No	Strong	Moderate
3	Sagariga et al.	Plastic to Fuel Conversion System Using Renewable Energy Assisted Pyrolysis	2020	Asia	https://ieeexplore.ieee.org/document/9074266	• Renewable technologies as power source for the pyrolysis of plastic wastes	Critical	Critical	N/A	Beneficial	N/A	7, 12	8, 9, 13	No	No	Strong	No	No	No	Strong	Moderate
4	Posen et al.	Greenhouse gas mitigation for U.S. plastics production: Energy first, feedstocks later	2017	North America	https://iopscience.iop.org/article/10.1088/1748-9326/aa60a7	• Strategies to reduce greenhouse gas emissions from US plasics production	Neutral	Neutral	N/A	Neutral	N/A	7, 9, 12, 13	8, 11	Strong	No	Strong	No	No	No	No	Moderate
5	Gebre et al.	Recent Trends in the Pyrolysis of Non-Degradable Waste Plastics	2021	Global	https://chemistry-europe.onlinelibrary.wiley.com/doi/10.1002/open.202100184	• Review of pyrolysis of plastic wastes and the factors affecting them	Critical	Critical	N/A	Beneficial	N/A		7, 8, 9, 11, 12, 13	No	No	Strong	No	No	No	Strong	Moderate
6	Gug et al.	Processing and properties of a solid energy fuel from municipal solid waste (MSW) and recycled plastics	2015	North America	https://www.sciencedirect.com/science/article/pii/S0956053X14004565?via%3Dihub	• Effects of processing conditions on the properties of MSW solid fuels	Critical	Critical	N/A	Beneficial	N/A	7, 11, 12	8, 9, 13	No	No	Strong	No	No	No	Strong	Moderate
7	Sharuddin et al.	Pyrolysis of plastic waste for liquid fuel production as prospective energy resource	2018	Global	https://iopscience.iop.org/article/10.1088/1757-899X/334/1/012001	• Review of the thermal and catalytic degradation of plastic through pyrolysis	Critical	Critical	N/A	Beneficial	N/A	6, 12	7, 8, 9, 11, 13	No	No	Strong	No	No	No	Strong	Moderate
8	Lombardi et al.	A review of technologies and performances of thermal treatment systems for energy recovery from waste	2015	Global	https://www.sciencedirect.com/science/article/pii/S0956053X14005273?via%3Dihub	• Review of different thermal treatments of different wastes	N/A	N/A	N/A	Beneficial	N/A	7, 9, 11, 12	8, 13	No	No	Moderate	No	No	No	Moderate	Moderate
9	Wong et al.	Current state and future prospects of plastic waste as source of fuel: A review	2015	Global	https://www.sciencedirect.com/science/article/pii/S1364032115003307?via%3Dihub	• In depth analysis with respect to the pyrolysis of plastic wastes	Critical	Critical	N/A	Beneficial	Beneficial	7, 8, 12	9, 11, 13	No	No	Moderate	No	No	No	Moderate	Moderate
10	Sharuddin et al.	A review on pyrolysis of plastic wastes	2016	Global	https://www.sciencedirect.com/science/article/pii/S0196890416300619?pes=vor	• Review of pyrolysis for each type of plastic and their main process parameters	N/A	N/A	N/A	Beneficial	N/A	6, 9	7, 8, 11, 12, 13	No	No	Moderate	No	No	No	Moderate	Moderate
11	Lam et al.	Microwave vacuum pyrolysis of waste plastic and used cooking oil for simultaneous waste reduction and sustainable energy conversion: Recovery of cleaner liquid fuel and techno-economic analysis	2019	Asia	https://www.sciencedirect.com/science/article/abs/pii/S1364032119305672?via%3Dihub	• Economic feasibility of microwave vacuum pyrolysis	N/A	N/A	N/A	Beneficial	N/A	7	8, 9, 11, 12, 13	No	No	Moderate	No	No	No	Moderate	Moderate
12	Shen et al.	Waste-to-energy: Dehalogenation of plastic-containing wastes	2016	Global	https://www.sciencedirect.com/science/article/pii/S0956053X15302579?pes=vor	• Review on the process parameters for plastic dehalogenation	Critical	Critical	N/A	Beneficial	N/A	7, 12	8, 9, 11, 13	No	No	Moderate	No	No	No	Moderate	Moderate
13	Chandrasekeran et al.	Materials and Energy Recovery from E-Waste Plastics	2018	Global	https://pubs.acs.org/doi/10.1021/acssuschemeng.7b03282	• Energy efficient processes to extract value added materials from ewastes	Critical	Critical	N/A	Beneficial	Critical	7, 12	8, 9, 11, 13	No	No	Moderate	No	No	No	Moderate	Moderate
14	Bukkarapu et al.	Management, conversion, and utilization of waste plastic as a source of sustainable energy to run automotive: a review	2018	Global	https://www.tandfonline.com/doi/full/10.1080/15567036.2018.1486898	• Review on the pyrolysis of plastic wastes	Critical	Critical	N/A	Beneficial	N/A	7, 13	8, 9, 11, 12	No	No	No	No	No	No	Moderate	Moderate
15	Devasahayam et al.	Review: Opportunities for simultaneous energy/materials conversion of carbon dioxide and plastics in metallurgical processes	2019	Global	https://www.sciencedirect.com/science/article/abs/pii/S221499371930096X?via%3Dihub	• Review of simultaneous conversion and utilization of carbon dioxide and plastics into useful fuels	Critical	Critical	N/A	Beneficial	N/A	7, 9, 12, 13	8, 11	No	No	No	No	No	No	Moderate	Moderate
16	Surenderan et al.	Characterization studies on waste plastics as a feedstock for energy recovery in Malaysia	2018	Asia	https://researchportal.hw.ac.uk/en/publications/characterization-studies-on-waste-plastics-as-a-feedstock-for-ene	• Characterizing waste plastics as a feedstock for using them as fuel. Proximate and ultimate analysis was carried out	Critical	Critical	N/A	Neutral	N/A	12, 17	7, 8, 9, 11	No	No	Moderate	No	No	No	Moderate	No
17	Lim et al.	Optimal sorting and recycling of plastic waste as a renewable energy resource considering economic feasibility and environmental pollution	2023	Global	https://www.sciencedirect.com/science/article/pii/S095758202200979X?pes=vor	• Optimization model to calculate the optimal sorting and recycling of plastic wastes. State of the art systems are reviewed and discussed	Critical	Critical	N/A	Neutral	N/A	7, 12	8, 9, 10, 11, 17	No	No	Moderate	No	No	Strong	Strong	Moderate
18	Willenbacher et al.	Machine learning for optimization of energy and plastic consumption in the production of thermoplastic parts in SME	2021	Europe	https://www.mdpi.com/2071-1050/13/12/6800	• Discuss on whether machine learning can improve energy consumption and reduce incorrectly produced parts in SME	N/A	Beneficial	N/A	N/A	N/A	7, 9, 12	4, 8, 11	Strong	No	Moderate	No	No	No	No	Moderate
19	Olazabal et al.	From plastic waste to new materials for energy storage	2022	Global	https://pubs.rsc.org/en/content/articlelanding/2022/PY/D2PY00592A	• Description of strategies to use plastic wastes as a cheap feedstock in the production of energy storage devices like supercapacitors	Critical	N/A	N/A	Beneficial	N/A		7, 8, 9, 11, 12	Strong	No	Moderate	No	No	No	Strong	Moderate
20	Nugraho et al.	Plastic waste as an alternative energy	2017	Global	https://aip.scitation.org/doi/abs/10.1063/1.5043022	• Use of pyrolysisi oil as a fuel source	Critical	Critical	N/A	Beneficial	N/A	7, 12	8, 9, 11	No	No	Moderate	No	No	No	Strong	Moderate
**Goal 8. Promote sustained, inclusive and sustainable economic growth, full and productive employment and decent work for all**	**Keywords: Economy, Job, Growth, Employment**																				
1	Umeda et al.	Potential impacts of the European Union's circular economy policy on Japanese manufacturers	2020	Asia	https://www.fujipress.jp/ijat/au/ijate001400060857/	• Implications of European CE policy on Japanese Manufacturers • Challenges on how the European CE Policy is different from that of the Japaneses ones and their impacts	N/A	N/A	N/A	Critical	N/A	7, 8, 9, 12	10, 11, 17	Moderate	No	Moderate	No	No	Moderate	Strong	No
2	Andreoni et al.	Polyethylene recycling: Waste policy scenario analysis for the EU-27	2015	Europe	https://www.sciencedirect.com/science/article/pii/S0301479715300414?via%3Dihub	• Adoption of the best recycling practices and reduction in the consumption of plastic bags in EU	N/A	N/A	N/A	Critical	N/A	8, 12	7, 9, 10, 11, 17	No	No	No	No	No	Moderate	No	Moderate
3	Nara et al.	Expected impact of industry 4.0 technologies on sustainable development: A study in the context of Brazil's plastic industry	2021	South America	https://www.sciencedirect.com/science/article/abs/pii/S2352550920305212?via%3Dihub	• Decision making process in implementing Industry 4.0 in Plastic industries in Brazil • The technologies are associated with susbstantial positive impacts on several metrics	N/A	Neutral	N/A	Neutral	N/A	8, 9, 12, 17	10, 11, 13	No	No	Moderate	No	No	No	No	Moderate
4	Vimal et al.	Analysis of barriers that impede the elimination of single-use plastic in developing economy context	2019	Asia	https://www.sciencedirect.com/science/article/abs/pii/S0959652620326767?via%3Dihub	• Based on a Decision Making Trial and Evaluation Laboratory, a framework has been developed to understand the barriers of eliminating single use plastic bags	Critical	Neutral	N/A	Highly critical	N/A	17	4, 8, 9, 10, 11, 12	No	Moderate	Moderate	No	No	Strong	Moderate	Strong
5	Rossi et al.	Circular economy indicators for organizations considering sustainability and business models: Plastic, textile and electro-electronic cases	2019	South America	https://www.sciencedirect.com/science/article/abs/pii/S0959652619340077?via%3Dihub	• Developing a set of indicators linking CE principles and business models along with the pillars of sustainability	N/A	N/A	N/A	N/A	N/A	8, 9	4, 7, 11, 12, 17	No	No	Moderate	No	No	Moderate	Moderate	Moderate
6	Wu et al.	Supporting a circular economy: Insights from Taiwan's plastic waste sector and lessons for developing countries	2020	Asia	https://www.sciencedirect.com/science/article/abs/pii/S235255092031349X?via%3Dihub	• Analysing and understanding of developing the Taiwan plastic industry into a circular economy which will result in different impacts on the job creation and innovation	Highly critical	Critical	Critical	Highly critical	N/A	8, 9, 12, 17	11, 13, 14, 15	No	Moderate	Moderate	No	No	Moderate	Strong	Strong
7	Carola Guyot Phung	Implications of the circular economy and digital transition on skills and green jobs in the plastics industry	2019	Europe	https://journals.openedition.org/factsreports/5498	• How Europe's plastic industry generate income and job growth during their transition towards circular economy	N/A	N/A	N/A	Beneficial	N/A		4, 8, 9, 11, 12, 17	No	Moderate	Strong	No	No	Moderate	Strong	Strong
8	Hofmann et al.	The value chain and activities of polyethylene terephthalate plastics in the South African waste economy	2020	Africa	https://journals.sagepub.com/doi/10.1177/0269094220931697	• A qualitative research on the complete value chain of PET is done to identify the possibilities of job creation in South Africa	Critical	N/A	N/A	Neutral	N/A	8, 9, 17	4, 10, 11, 12, 13	No	No	No	No	No	Moderate	Strong	Moderate
9	Bala et al.	Identifying the prospects of decent job creation along the value chain of plastic recycling	2021	Africa	https://www.tandfonline.com/doi/abs/10.1080/0376835X.2021.2018290?journalCode=cdsa20	• Survey of the recycling of plastics in Capetown Southafrica to understand the barriers and impacts on job creation	N/A	N/A	N/A	Neutral	N/A	8, 12, 17	1, 4, 9, 10, 11	Moderate	No	No	No	No	No	Moderate	Moderate
10	Bening et al.	The true cost of solving the plastic waste challenge in developing countries: The case of Ghana	2022	Africa	https://www.sciencedirect.com/science/article/pii/S0959652621038270?via%3Dihub	• Novel model based method to assess the cost structure of the recycling sector in developing countries, haveing Accra Ghana as a case study	N/A	N/A	N/A	Neutral	N/A	1, 8, 10 11, 12, 17	9	No	No	No	No	No	Moderate	Moderate	Moderate
11	Bai et al.	Ecologically unequal exchange of plastic waste? A longitudinal analysis of international trade in plastic waste	2021	Global	http://jwsr.pitt.edu/ojs/jwsr/article/view/1026	• Examine the extent of the Global plastic trade from Global north to South • Higher waste import associate with the greater economic develoment in non high income countries	Critical	Critical	N/A	Beneficial	Critical	8	9, 10, 11, 12, 16, 17	Moderate	Moderate	No	No	No	Moderate	Moderate	Strong
12	Torres et al.	The need for technical improvement in the plastics recycling industry in middle-income countries: The Peruvian case	2016	South America	https://journals.sagepub.com/doi/10.1177/1477760616031200402	• How variations in technical and work regulations cna influence the cost strucutre of plastic recycling operations in Peru	N/A	N/A	N/A	Critical	Critical	8, 12	4, 9, 10, 11, 13, 17	No	No	No	No	No	No	Moderate	No
13	Cordier et al.	Plastic pollution and economic growth: The influence of corruption and lack of education	2021	Global	https://www.sciencedirect.com/science/article/pii/S0921800920322217?via%3Dihub	• Based on models and scenario analysis, the factors that affect the inadequately managed plastic waste across the world	Highly critical	Critical	N/A	Highly critical	N/A	8, 16	4, 9, 10, 11, 12, 17	No	No	No	No	No	No	Moderate	No
14	Stuart J. Barnes	Understanding plastics pollution - The role of economic development and technological research	2019	Global	https://www.sciencedirect.com/science/article/abs/pii/S0269749119306505?via%3Dihub	• Relationship between mismanaged plastic waste and income per capita for different countries and findings from the study on reducing plastic pollution	Highly critical	Highly critical	N/A	Beneficial	Highly critical	8, 14	9, 10, 15, 16	No	No	No	No	No	Unclear	Moderate	Moderate
15	Wu et al.	Impact of strategic control and supply chain management on recycled plastic additive manufacturing	2022	Global	https://www.sciencedirect.com/science/article/pii/S0959652622021126?via%3Dihub	• How localization of plastics production through additive manufacturing can decentralise the supply chain thereby reducing the pollution	N/A	Beneficial	N/A	N/A	Beneficial	9, 12, 13, 17	8, 11	No	No	Strong	Strong	No	No	Moderate	Strong
16	Silva et al.	Simulation of elements of industry 4.0 applied to the production processes of a company dedicated to the manufacture of plastic products	2020	South America	https://www.cal-tek.eu/proceedings/i3m/2020/emss/012/	• Simulation model to help companies consider factors that will cut down the costs and wastes	N/A	Beneficial	N/A	N/A	N/A	8, 9, 17	11, 12	Strong	Strong	Strong	No	No	No	No	No
17	Babarinsa et al.	Potential Socio-economic impact of replacing traditional woven baskets with reusable plastic crates on livelihoods of basker makers in southeast Nigeria	2022	Africa	https://ajfand.net/Volume22/No6/Babarinsa22035.pdf	• Assessment of the potential socio-economic impacts when finding new product alternatives using a questionnaire	Beneficial	Beneficial	Neutral	Beneficial	Beneficial	17	1, 4, 8, 9, 12	Strong	No	No	No	No	No	No	Strong
18	Alvarado-Diaz et al	Design of a Plastic Shredding Machine to Obtain Small Plastic Waste	2021	South America	https://thesai.org/Downloads/Volume12No6/Paper_54-Design_of_a_Plastic_Shredding_Machine.pdf	• To design a plastic shredding machine that will recycle automatically the plastic wastes which will then be used for 3D filament printing	Highly critical	Highly critical	N/A	Neutral	N/A	17	8, 9, 11, 12	No	No	Strong	No	No	Strong	No	No
19	Arabi et al.	Impacts of marine plastic on ecosystem services and economy: State of South African research	2020	Africa	https://sajs.co.za/article/view/7695	• Review highlighting the knowledge gaps on the marine plastic pollution and the economic impacts of pollution	Highly critical	Highly critical	N/A	Highly critical	N/A	8, 12, 14	9, 10, 11, 13, 15, 17	No	No	No	No	No	No	No	Moderate
20	Adedeji Adelodun	Plastic Recovery and Utilization: From Ocean Pollution to Green Economy	2021	Global	https://www.frontiersin.org/articles/10.3389/fenvs.2021.683403/full	• Review on the fate and control measures for plastics and the techniques for resource control ae addressed	Highly critical	Highly critical	N/A	Highly critical	N/A	8, 14	7, 9, 10, 11, 12, 13, 17	No	No	Moderate	No	No	Moderate	Moderate	Moderate
**Goal 9. Build resilient infrastructure, promote inclusive and sustainable industrialization and foster innovation**	**Keywords: Innovation, Infrastructure, Research, Industry**																				
1	Moshood et al.	Biodegradable plastic applications towards sustainability: A recent innovations in the green product	2022	Global	https://www.sciencedirect.com/science/article/pii/S266679082200009X?via%3Dihub	• Various motives that drive companies to produce biodegradable plastics	Neutral	N/A	N/A	Neutral	N/A	9, 12	8, 13, 14, 15	Moderate	No	No	No	No	No	No	Strong
2	Raddadi et al.	Biodegradation of oil-based plastics in the environment: Existing knowledge and needs of research and innovation	2019	Global	https://www.sciencedirect.com/science/article/abs/pii/S0048969719319709?via%3Dihub	• Summary of the approaches to monitor the biodradability of oil-based conventional plastics in terrestrial and acquatic environments	Highly critical	Highly critical	N/A	Critical	N/A	14	9, 12, 13, 15	Moderate	No	No	No	No	Moderate	No	No
3	Getor et al.	The role of technological innovation in plastic production within a circular economy framework	2020	Europe	https://www.sciencedirect.com/science/article/abs/pii/S0921344920304110?via%3Dihub	• Framework for decision making in identifying the right virgin-recycled resin mix ratio when using different batches	Highly critical	Critical	N/A	Critical	N/A	14	8, 9, 12, 13, 15	Strong	No	Moderate	No	No	Moderate	Moderate	Strong
4	Junior et al.	Knowledge-based dynamic capabilities for sustainable innovation: The case of the green plastic project	2019	South America	https://www.mdpi.com/2071-1050/11/8/2392	• Identifying the set of practices based on interviews that are essential to the development of sustainable innovation with green plastic project as a case study	N/A	N/A	N/A	N/A	N/A	9	8, 12, 13, 17	Strong	No	Strong	No	No	No	No	Strong
5	Nel et al.	Collaboration and infrastructure is needed to develop an African perspective on micro(nano)plastic pollution	2021	Africa	https://iopscience.iop.org/article/10.1088/1748-9326/abdaeb	• A development of a pan-African research network that is needed to support research exchange is being identified	Highly critical	Highly critical	N/A	Highly critical	N/A	17	1, 9, 10, 11, 12, 16	No	No	No	No	No	No	No	Strong
6	Friedrich et al.	Supporting the development process for building products by the use of research portfolio analysis: A case study for wood plastics composite materials	2016	Europe	https://www.sciencedirect.com/science/article/pii/S2214509515300188?via%3Dihub	• Evaluation of the European scientific projects in composites to understand the strategic innovation planning	N/A	N/A	Beneficial	N/A	N/A	9	12, 13, 14, 15, 17	Moderate	No	No	No	No	No	No	Moderate
7	Sommer et al.	Recycling and recovery infrastructures for glass and carbon fiber reinforced plastic waste from wind energy industry: A European case study	2021	Europe	https://www.sciencedirect.com/science/article/abs/pii/S0956053X20307108?via%3Dihub	• An ex-ante analysis on the cost of recycling infrastructure for wind energy blades made of composites	N/A	N/A	N/A	Critical	N/A	7, 12	8, 9, 11, 13, 17	No	Moderate	No	No	No	Strong	Strong	Strong

8	Gong et al.	Investigation into circular economy of plastics: The case of the UK fast moving consumer goods industry	2020	Europe	https://www.sciencedirect.com/science/article/abs/pii/S0959652619338119?via%3Dihub	• Review on the initiatives in the FMCG industry on implementing CE of plastics	Critical	N/A	N/A	Critical	N/A	9, 12	8, 11, 13, 14, 15, 17	Strong	Strong	Moderate	Moderate	No	Strong	Moderate	Strong
9	Dijkstra et al.	In the business of dirty oceans: Overview of startups and entrepreneurs managing marine plastic	2021	Europe	https://www.sciencedirect.com/science/article/abs/pii/S0025326X2030998X?via%3Dihub	• Overview on the entreprenuerial and SMEs working on solutions to reduce marine plastics	Critical	Critical	N/A	Neutral	N/A	9, 14	8, 11, 12, 13, 15, 17	Moderate	No	No	No	No	Moderate	Strong	Moderate
10	Siltaloppi et al.	Toward a sustainable plastics value chain: Core conundrums and emerging solution mechanisms for a systemic transition	2021	Europe	https://www.sciencedirect.com/science/article/pii/S0959652621023313?via%3Dihub	• Qualitative study on the complex interdependencies across the value chain of plastics	Critical	Critical	N/A	Highly critical	N/A	9	8, 11, 12, 13, 14, 15, 17	Moderate	No	Moderate	No	Moderate	Strong	Strong	Moderate
11	Cheng et al.	Investigating the Economic Feasibility of Community-Scale Plastic Recycling Facilities	2021	Asia	https://ieeexplore.ieee.org/document/9612502/	• Examines the economic feasibility of community scale recycling of plastic wastes	Critical	Critical	N/A	Neutral	N/A	9, 12, 17	8, 11, 13, 14, 15	No	No	Moderate	No	No	Moderate	Moderate	Strong
12	Bing et al.	Global reverse supply chain redesign for household plastic waste under the emission trading scheme	2015	Global	https://www.sciencedirect.com/science/article/pii/S0959652615001328?via%3Dihub	• Redesigning a reverse supply chain to understand emission trading restrictions in other countries	Critical	Critical	N/A	Critical	Critical	8, 9, 12, 13, 17	10, 11, 14, 16	No	No	No	Strong	No	No	Strong	Strong
13	Muposhi et al.	Considerations, benefits and unintended consequences of banning plastic shopping bags for environmental sustainability: A systematic literature review	2022	Global	https://journals.sagepub.com/doi/10.1177/0734242X211003965	• Literature review on the policy options and effectiveness of banning plastic bags for environmental sustainability	Highly critical	Highly critical	Neutral	Highly critical	Critical	9, 17	3, 4, 8, 10, 11, 12, 13, 14, 15, 16	No	No	No	No	Moderate	No	Strong	Moderate
14	Knoblauch et al.	Government policies combatting plastic pollution	2021	Global	https://www.sciencedirect.com/science/article/pii/S2468202021000619?pes=vor	• Literature review of government actions to regulate plastic pollution	Critical	Critical	Highly critical	Highly critical	Critical		9, 12, 13, 14, 15, 16, 17	No	No	No	Moderate	Moderate	Moderate	Strong	Moderate
15	Rivera-Huerta et al.	Innovation in the informal sector: The case of plastic recycling firms in Mexico	2022	North America	https://www.tandfonline.com/doi/full/10.1080/20421338.2020.1864881	• Highlighting the different types of innovations in the Mexican informal plastic recycling industry	N/A	N/A	N/A	Neutral	N/A	8, 9, 12, 17	1, 3, 4, 6, 10, 11	No	No	Moderate	No	No	Moderate	No	No
16	Oyake-Ombis et al.	Managing plastic waste in East Africa: Niche innovations in plastic production and solid waste	2015	Africa	https://www.sciencedirect.com/science/article/pii/S0197397515000673?pes=vor	• 7 innovation pathways through which plastic pollution can be reduced in Kenya	Highly critical	Critical	Critical	Critical	Critical	9, 11, 12, 17	1, 8, 13, 14, 15	No	No	No	Moderate	Moderate	Moderate	Strong	Moderate
17	Cordier et al.	How much innovation is needed to protect the ocean from plastic contamination?	2019	Global	https://www.sciencedirect.com/science/article/abs/pii/S0048969719312501?via%3Dihub	• Based on a system dynamics model, scenarios are developed in controlling plastic debris entering ocean	Highly critical	Highly critical	Critical	Highly critical	Critical	12, 14	1, 6, 8, 9, 10, 11, 13, 15, 16, 17	No	Strong	Moderate	Strong	Strong	Strong	Strong	Moderate
18	Huang et al.	Evolution of network relations, enterprise learning, and cluster innovation networks: the case of the Yuyao plastics industry cluster	2018	Asia	https://www.tandfonline.com/doi/full/10.1080/09537325.2017.1297786	• How industrial cluster networks can promote economic growth and innoation with an example of yuyao plastics cluster	N/A	Neutral	N/A	N/A	N/A	9	4, 8, 11, 12, 17	Moderate	No	Moderate	No	No	No	Moderate	Strong
19	Oyinlola et al.	Digital innovations for transitioning to circular plastic value chains in Africa	2022	Africa	https://www.tandfonline.com/doi/full/10.1080/23322373.2021.1999750	• Overview of plastic trade and assessment of existing digital solutions for a transition to a circular economy	Highly critical	Highly critical	N/A	Highly critical	N/A	9, 12, 17	8, 11, 13, 14, 15	No	No	No	No	Moderate	No	Moderate	Strong
20	Ishartomo et al.	Learning from plastic waste village in Boyolali Indonesia: SME-based plastic recycling industries	2019	Asia	https://aip.scitation.org/doi/abs/10.1063/5.0000700	• Study on how recylcing of plastic wastes through SMEs provide income to an Indonesian village	Critical	Critical	Critical	Highly critical	Critical	9, 12, 16	1, 6, 8, 10, 11, 17	No	No	No	No	No	No	Moderate	No
21	Conchubhair et al.	Joint effort among research infrastructures to quantify the impact of plastic debris in the ocean	2019	Global	https://iopscience.iop.org/article/10.1088/1748-9326/ab17ed	• Study on how analysing the research infrastructures can help in identifiying the impacts of plastic debris in the oceans	Highly critical	Highly critical	N/A	Highly critical	N/A	14	4, 8, 9, 11, 12, 15, 17	No	No	No	No	No	No	No	Moderate
22	Ma et al.	Economic evaluation of infrastructures for thermochemical upcycling of post-consumer plastic waste	2022	North America	https://pubs.rsc.org/en/content/articlelanding/2023/GC/D2GC04005K	• Holistic framework to analyse the economic feasibility of thermochemical recycling of plastic wastes	Critical	Critical	N/A	Neutral	N/A	7, 12, 13	8, 9, 11, 17	No	No	Moderate	Moderate	No	No	Moderate	No
23	Hinton et al.	Innovations Toward the Valorization of Plastics Waste	2022	Global	https://www.annualreviews.org/doi/10.1146/annurev-matsci-081320-032344	• State of the art valorization of plastic wastes	Highly critical	Highly critical	N/A	Highly critical	N/A	9, 12	7, 8, 11, 13	No	No	Strong	No	No	No	Strong	No
24	Nancy Lamontagne	Recycling the Unrecyclable Industry innovations aim to keep traditionally difficult-to-recycle plastics out of the landfill	2016	North America	https://onlinelibrary.wiley.com/doi/10.1002/j.1941-9635.2016.tb01526.x	• Innovations in the industry that recycles the unrecyclable plastic wastes	Critical	Critical	N/A	Neutral	Neutral	9	8, 12, 13, 17	No	No	Strong	No	No	No	Strong	Strong
25	Vollmer et al.	Beyond Mechanical Recycling: Giving New Life to Plastic Waste	2020	Global	https://onlinelibrary.wiley.com/doi/10.1002/anie.201915651	• Review of various chemical recycling routes of plastic wastes	Critical	Critical	N/A	Neutral	N/A	12	7, 8, 9, 11, 13, 14, 15	No	No	Strong	No	No	No	Strong	No
26	Muposhi et al.	Embedding Ecopreneurial Behaviour: Proposed Social Marketing Interventions From Value-In-Behaviour Perceptions of Plastic Waste Ecopreneurs	2023	Africa	https://journals.sagepub.com/doi/10.1177/15245004221150222	• To develop an understanding of the relationship between ecopreneur and social marketing in the context of developing economy	Highly critical	N/A	N/A	Highly critical	N/A	9, 11, 12, 17	1, 3, 4, 6, 8	No	No	No	No	No	No	Strong	Strong
27	da Silva et al.	Evolution toward environment sustainable behavior: search for survival in the plastic industry in Brazil	2019	South America	https://link.springer.com/article/10.1007/s10668-018-0085-3	• Qualitative assessment of information obtained from plastic industries on the evolution of sustainability behaviour in Brazil	Critical	Critical	N/A	Highly critical	N/A	9, 17	8, 11, 12, 13, 14, 15	No	No	Moderate	No	No	Moderate	No	No
28	Ana Espada	Design as a source of innovation to establish circular models: An opportunity to prevent the single use of plastic	2020	Europe	https://www.igi-global.com/gateway/chapter/full-text-pdf/258713	• Design solution to support the business transition to a circular economy	Critical	Critical	Critical	Critical	Critical	9, 12	4, 11, 13, 14, 15, 17	No	Moderate	Moderate	No	Moderate	Strong	Moderate	Moderate
29	Chow et al.	Research and development of a new waste collection bin to facilitate education in plastic recycling	2016	Asia	https://www.tandfonline.com/doi/abs/10.1080/1533015X.2016.1141723?journalCode=ueec20	• Deisign of plastic recycling bin to collect plastic wastes of different polymer types according to the production and waste generation rate	N/A	N/A	Critical	Critical	Critical	9, 11, 12	4, 8	No	No	No	No	Moderate	Strong	Moderate	Strong
30	Reddy et al.	Review of the Utilization of Plastic Wastes as a Resource Material in Civil Engineering Infrastructure Applications	2022	Global	https://ascelibrary.org/doi/epdf/10.1061/(ASCE)HZ.2153-5515.0000717?src=getftr	• Literature review of utilizing plastic wastes in applications like bricks, embankments in accordance to the international standards	Critical	Critical	N/A	Neutral	N/A	14	8, 9, 12, 13	Moderate	No	Moderate	No	No	No	No	Moderate
**Goal 10. Reduce inequality within and among countries**	Keywords: Income, Injustice, Inequality, Social																				
1	Velis et al.	Plastic pollution global treaty to cover waste pickers and open burning?	2022	Global	https://journals.sagepub.com/doi/10.1177/0734242X211069583	• Discussion on a framework for the international plastic treaty to include open burning of plastic wastes	Highly critical	Highly critical	Critical	Highly critical	Critical		1, 3, 10, 11, 12, 13, 14, 15, 16, 17	No	No	No	No	No	No	Strong	Strong
2	Benedetta Cotta	What goes around, comes around? Access and allocation problems in Global North–South waste trade	2020	Global	https://link.springer.com/article/10.1007/s10784-020-09479-3	• Lessons learnt on allocating and access to burdens on processing and disposal of recyclable wastes	Highly critical	Highly critical	N/A	Highly critical	Highly critical	3, 10	1, 8, 9, 11, 12, 13, 14, 15, 16, 17	No	No	No	Strong	No	Moderate	Moderate	Strong
3	Samantha Chisholm Hatfield	Plastic suffocation: Climate change threatens indigenous populations and traditional ecological knowledge	2019	Global	https://jmic.online/issues/v8n2/1/	• Discuss about how plastic use and wastes produced threatens the lives of indigeneous population	Highly critical	Highly critical	Highly critical	Highly critical	Critical	14	3, 10, 11, 13, 15, 16, 17	Strong	No	No	No	No	Strong	No	No
4	Horvath et al.	Designing Business Solutions for plastic waste management to enhance circular transitions in Kenya	2018	Africa	https://www.mdpi.com/2071-1050/10/5/1664	• Discuss how priorities of circular economy is not applicable equally across the world and with a help of Buscinesss Model Canvas approach, a holistic solution is offered	Highly critical	Critical	Critical	Critical	Highly critical	12, 17	4, 8, 9, 10, 11, 14, 15, 16	No	Moderate	Moderate	Strong	Moderate	Moderate	Moderate	Strong
5	Horne et al.	High-rise plastic: Socio-material entanglements in apartments	2022	Global	https://rgs-ibg.onlinelibrary.wiley.com/doi/10.1111/geoj.12457	• How the use and understanding of plastics in the apartments differ across the different classes of people in the apartments across the world	N/A	N/A	Highly beneficial	Critical	N/A	11, 12	1, 8, 9, 10	No	No	No	No	No	Strong	No	No
6	Jamie Furniss	Alternative framings of transnational waste flows reflections based on the Egypt China PET plastic	2015	Africa	https://rgs-ibg.onlinelibrary.wiley.com/doi/full/10.1111/area.12160	• Discuss about the neocolonial geographies of inequality paradigm when it comes to the trade of plastic and recyclin gin Egypt	N/A	N/A	N/A	N/A	Critical	12, 17	8, 10	No	No	No	No	No	No	No	No
7	Borman et al.	Impact of plastic packaging design on the sustainability of plastic recyclers	2019	Asia	http://ir.lib.cyut.edu.tw:8080/bitstream/310901800/36775/1/ACISE-IJASE+2019-003-edited-final.pdf	• Impact of material value conservation on the plastic packaging sustainability thereby increasing the recycling share	Critical	Critical	Neutral	Highly critical	N/A	4, 12, 14, 15	9, 11, 13	Moderate	No	Moderate	No	No	Moderate	Moderate	Moderate
8	Saskia Abrahms-Kavunenko	Toward an anthropology of plastics	2021	Global	https://journals.sagepub.com/doi/10.1177/13591835211066808	• An anthropological view on Plastics across their lifecycle comparing their benefits with the disadvantages and harm caused by them	Highly critical	Highly critical	Neutral	Highly critical	N/A		3, 10, 11, 13, 14, 15, 16	No	No	No	No	No	No	No	No
9	Nicolas Schlitz	Environmental change and the informal plastic recycling networks of Kolkata	2020	Asia	https://onlinelibrary.wiley.com/doi/10.1111/sjtg.12324	• Understanding the contradictory entanglement of economic and environmental change in plastic recycling with a case study on the workers of informal plastic recycling in India	Critical	Critical	N/A	Critical	Neutral	8, 11, 12, 17	1, 3, 9, 10, 13	No	No	No	No	No	Strong	Strong	Moderate
10	Hossain et al.	Socioeconomic Relation with Plastic Consumption on 61 Countries Classified by Continent, Income Status and Coastal Regions	2021	Global	https://link.springer.com/article/10.1007/s00128-021-03231-6	• Potential relationship between the plastic consumptions and socio-economic status of different countries	Critical	Critical	N/A	Beneficial	N/A		1, 3, 4, 8, 9, 10, 11, 14, 15	No	No	No	No	No	No	No	No
11	Kumar et al.	Estimation of the generation rate of different types of plastic wastes and possible revenue recovery from informal recycling	2018	Asia	https://www.sciencedirect.com/science/article/abs/pii/S0956053X18305348?via%3Dihub	• Establishing a relationship between plastic waste generation and the socio-economic groups	Critical	N/A	N/A	Critical	Neutral	11, 12, 17	1, 4, 8, 9, 10	No	No	No	No	No	No	Strong	No
12	Gareiou et al.	Awareness of Citizens for the Single-Use Plastics: Comparison between a High-Income and an Upper-Middle-Income Economy of the Easter Mediterranean Region, Greece and Lebanon	2022	Europe/Asia	https://www.mdpi.com/2071-1050/14/3/1912	• Study on the current use of plastics and potential for replacing them with sustainable alternatives	Critical	N/A	N/A	Critical	Critical	17	4, 9, 10, 11, 12, 14, 15	Moderate	No	No	No	No	Moderate	Moderate	No
13	Knoblauch et al.	Developing countries in the lead-what drives the diffusion of plastic bag policies?	2018	Global	https://www.mdpi.com/2071-1050/10/6/1994	• Diffusion policies, bans and taxes on Plastic bags between Global North and Global South	Critical	N/A	N/A	Critical	Critical	4, 11, 12, 14, 17	9, 10, 13, 15	Moderate	No	No	No	No	Moderate	Moderate	Moderate
14	Muisa Zikali et al.	Household solid waste handling practices and recycling value for integrated solid waste management in a developing city in Zimbabwe	2022	Africa	https://reader.elsevier.com/reader/sd/pii/S246822762200059X?token=6C68F1F74CD44A4D91CEC1FBC97F734702B373C7CB07B636F6BAC960F1B3048427FC6EE2247757057F51A162DED80050&originRegion=eu-west-1&originCreation=20230126134126	• Characterise, quantify and analyse the different solid waste management practices and recyclin gof them in Zimbabwe	N/A	N/A	N/A	Critical	Critical	1, 10, 11, 12, 17	1, 3, 4, 8, 9, 15	Moderate	No	No	No	No	Moderate	Moderate	No
15	Karasik et al.	Inequitable distribution of plastic benefits and burdens on economies and public health	2023	Global	https://www.frontiersin.org/articles/10.3389/fmars.2022.1017247/full	• Assessment of benefits and burdens of plastics across different stakeholders on economic and public health	Neutral	Neutral	Neutral	Neutral	Neutral	3, 10, 12	1, 4, 8, 9, 11, 14, 15 16, 17	No	No	No	No	No	No	No	Moderate
16	Navarre et al.	Recycled plastic packaging from the Dutch food sector pollutes Asian oceans	2022	Europe	https://www.sciencedirect.com/science/article/pii/S0921344922003512?pes=vor	• Estimating the plastic packaging in the Dutch diet	Neutral	Critical	Beneficial	Highly critical	Beneficial	12, 14	2, 9, 10, 11, 13, 15, 16	No	No	No	No	No	Strong	Strong	Moderate
17	Abalansa et al.	The marine plastic litter issue: A social-economic analysis	2020	Global	https://www.mdpi.com/2071-1050/12/20/8677	• Issue of Marine Plastic Pollution addressed based on Driver-Pressure-State-Impact-Response framework	Critical	Critical	Critical	Highly critical	Critical	2, 14, 15	6, 10, 11, 12	Moderate	No	No	No	No	Moderate	Moderate	Moderate
18	Cook et al.	Scaling up resource recovery of plastics in the emergent circular economy to prevent plastic pollution: Assessment of risks to health and safety in the Global South	2022	Global	https://journals.sagepub.com/doi/10.1177/0734242X221105415	• Assessment of potential risks in the recovery of post consumer plastic wastes in the Global South	Critical	Critical	Critical	Critical	N/A	3, 11, 12, 17	1, 4, 8, 9, 10, 13, 14, 15	Moderate	No	No	No	No	Moderate	Moderate	Moderate
19	Chowdhury et al.	The ecological impact of plastic pollution in a changing climate	2022	Asia	https://portlandpress.com/emergtoplifesci/article/6/4/389/232120/The-ecological-impact-of-plastic-pollution-in-a	• Connectivity and state of knowledge between plastic pollution, climate change and biodiversity loss in Global South	Highly critical	Highly critical	N/A	Highly critical	N/A	1, 2, 7, 10, 13, 14, 17	3, 4, 9, 11, 12, 15	No	No	No	No	No	No	No	Strong
20	Stuart J. Barnes	Out of sight, out of mind: Plastic waste exports, psychological distance and consumer plastic purchasing	2019	Global	https://www.sciencedirect.com/science/article/abs/pii/S0959378018312081?via%3Dihub	• How the countries with high consumption of plastics by transporting the wastes to other countries shift the burdens and change their perceptions on pollution	Critical	Critical	Critical	Highly critical	Critical		4, 10, 11, 12, 13, 14, 15, 17	No	No	No	No	No	No	No	Strong
**Goal 11. Make cities and human settlements inclusive, safe, resilient, and sustainable**	Keywords: Cities, Municipal, Waste, Communities, Settlements																				
1	Venkatesh et al.	Microbial degradation of plastics: Sustainable approach to tackling environmental threats facing big cities of the future	2021	Global	https://www.sciencedirect.com/science/article/pii/S1018364721000239	• Review of studies that analysed the degradation of plastics using microbes	Neutral	Critical	N/A	Critical	N/A	11	3, 9, 10, 12, 14, 15	No	No	No	No	No	No	Strong	No
2	Adam et al.	Attitudinal and behavioural segments on single-use plastics in Ghana: Implications for reducing marine plastic pollution	2021	Africa	https://www.sciencedirect.com/science/article/pii/S2667010021001645	• Examines behaviour of three different segments of society (avoiders, potential avoiders and patrons) towards SUP bags	Critical	Critical	Neutral	Critical	N/A	11, 14, 17	1, 3, 4, 6, 8, 9, 10, 12	No	No	No	No	Strong	Moderate	Strong	No
3	Varkey et al.	Identifying barriers to reducing single-use plastic use in a coastal metropolitan city in Canada	2021	North America	https://www.sciencedirect.com/science/article/abs/pii/S0964569121001472	• Quantitative and Qualitative methods to identify the barriers to reduce SUP bags in Halifax, Canada	Critical	Critical	Neutral	Critical	Critical	9, 14	4, 10, 11, 12, 15, 17	No	No	No	Moderate	No	No	Moderate	Strong
4	Hossain et al.	Strategies for mitigating plastic wastes management problem: A lifecycle assessment study in Hong Kong	2021	Asia	https://www.sciencedirect.com/science/article/abs/pii/S0956053X21003652	• Evaluate the environmental impacts of different EoL options of plastic wastes in Hongkong using LCA	N/A	N/A	N/A	Critical	N/A	9, 11, 12, 17	7, 13	No	No	Moderate	No	No	No	Moderate	No
5	Dong et al.	Uncovering opportunity of low-carbon city promotion with industrial system innovation: Case study on industrial symbiosis projects in China	2014	Asia	https://www.sciencedirect.com/science/article/pii/S030142151310410	• Analyses how industrial symbiosis projects can minimise CO2 emissions	N/A	N/A	N/A	Neutral	N/A	8, 9, 11, 12, 13, 17	7	No	No	No	No	No	No	Moderate	No
6	Wardrop et al.	Estimation of packaged water consumption and associated plastic waste production from household budget surveys	2017	Africa	https://iopscience.iop.org/article/10.1088/1748-9326/aa751f	• Quantify the use of packaged water relative to the household water requirements in west african countries	Critical	Critical	N/A	Beneficial	N/A	6, 11, 12	1, 3, 4, 9, 14, 15, 17	Moderate	No	No	No	No	Moderate	No	No
7	Abrokwah et al.	Drivers of single-use plastic waste generation: lessons from packaged water consumers in Ghana	2022	Africa	https://link.springer.com/article/10.1007/s10708-021-10390-w	• Determine the contextual factors and socio-demographic characterisitcs in the use of bottled water and opportunities for better waste management in Ghana	N/A	N/A	Beneficial	Critical	N/A	6, 11, 12, 17	1, 9, 10	No	No	No	No	No	Moderate	Moderate	No
8	Asari et al.	Analysis of mismanaged plastic waste in Samoa to suggest proper waste management in Pacific island countries	2019	Oceania	https://journals.sagepub.com/doi/10.1177/0734242X19867391	• Quantify the consumption of plastic wastes based on questionnaire conducted among housholds in Samoa	Critical	N/A	N/A	Highly critical	Critical	11, 12, 14, 17	4, 6, 9, 10, 15	Moderate	Moderate	No	No	No	No	Moderate	Moderate
9	Salhofer et al.	Plastic recycling practices in vietnam and related hazards for health and the environment	2021	Asia	https://www.mdpi.com/1660-4601/18/8/4203	• How the plastic wastes are handled in Vietnam with the help of case studies	Critical	Critical	N/A	Highly critical	Critical	3, 9, 11, 12, 17	1, 6, 8, 13, 14, 15	Moderate	No	Moderate	No	No	Moderate	Moderate	Moderate
10	Agusningtyas et al.	Processing of plastic waste from Klotok Landfill Kediri City with thermal cracking method	2019	Asia	https://iopscience.iop.org/article/10.1088/1755-1315/245/1/012041	• Characterisation of plastic wastes for the thermal cracking of them into liquid fuels in Indonesia	Critical	N/A	N/A	Neutral	N/A	11, 17	7, 9, 12, 13, 14, 15	No	No	Moderate	No	No	No	Strong	No
11	Kabir et al.	Plastic recycling from municipal solid waste in Jashore city of Bangladesh	2021	Asia	http://www.ieomsociety.org/singapore2021/papers/929.pdf	• Investigation of total solid wastes collection and overall recycling process at Jashore, Bangladesh	N/A	N/A	N/A	Neutral	Critical	9, 11, 12	3, 13	No	No	No	No	No	No	Moderate	No
12	Muslihun et al.	An Environmental Study on the Paid Plastic Bag Use Policy in the city of Semarang	2020	Asia	https://www.e3s-conferences.org/articles/e3sconf/abs/2020/62/e3sconf_icenis2020_06001/e3sconf_icenis2020_06001.html	• Analyse the implementation of policies, consumer behaviour and factors in the paid plastic bag policy in Semarang, Indonesia	N/A	N/A	Highly critical	Highly critical	N/A	11, 14	12, 13, 15, 17	No	No	No	No	No	No	No	Moderate
13	Cohen et al.	Parks and Recreational Areas as Sinks of Plastic Debris in Urban Sites: The Case of Light-Density Microplastics in the City of Amsterdam, The Netherlands	2022	Europe	https://www.mdpi.com/2076-3298/9/1/5	• Quantify the abundance and types of light density microplastics in public spaces in Amsterdam, The Netherlands	N/A	N/A	Critical	Highly critical	Critical	11	4, 6, 12, 13, 14, 15, 17	Strong	No	No	No	No	Moderate	Strong	Strong
14	Schuyler et al.	Environmental context and socio-economic status drive plastic pollution in Australian cities	2022	Australia	https://iopscience.iop.org/article/10.1088/1748-9326/ac5690	• Factors affecting the plastic litter distribution across different population groups based on generalised additive modelling of different sites across Australia	N/A	N/A	Critical	Highly critical	Critical	11, 12, 14	4, 13, 15, 17	Strong	No	No	No	No	Moderate	Strong	Strong
15	Correa et al.	Challenges to reducing post-consumer plastic rejects from the MSW selective collection at two MRFs in São Paulo city, Brazil	2022	South America	https://link.springer.com/article/10.1007/s10163-022-01387-9	• Characterizing the composition of screened mass flows of mixed wastesat two MRFs in Sao Paulo	Critical	Critical	N/A	Highly critical	Critical	11, 12	4, 8, 9, 10, 13, 14, 15, 17	Strong	No	No	No	No	Strong	Strong	Strong

16	Pertiwi et al.	Preliminary Study on Plastic Waste Handling in Semarang City - Indonesia: Estimated Generation and Existing Management	2018	Asia	https://www.e3s-conferences.org/articles/e3sconf/abs/2018/48/e3sconf_icenis18_07008/e3sconf_icenis18_07008.html	• Assessment of plastic waste management based on the material flow analysis of PET bottles in Semarang, Indonesia	Critical	Critical	N/A	Highly critical	Critical	6, **11**, 12, 14, 17	4, 9, 13, 15	Strong	No	No	No	No	Strong	Strong	Strong
17	Taha Ahmed Al-Tayyar	Characteristics of Plastic Solid Wastes in Mosul City and Their Reuse	2021	Asia	https://iopscience.iop.org/article/10.1088/1755-1315/779/1/012071	• Study the types and characteristics of wastes of different plastic types from Mosul city, Iraq	Critical	Critical	N/A	Critical	N/A	**11**, 12, 14, 15	4, 8, 9, 13, 17	Moderate	No	Moderate	No	No	Moderate	Moderate	Moderate
18	Brooks et al.	The Chinese import ban and its impact on global plastic waste trade	2018	Asia	https://www.science.org/doi/10.1126/sciadv.aat0131	• Using commodity trade data to characterise the impacts of chinese import ban on plastic wastes	Critical	Critical	N/A	Highly critical	Critical	10, **11**, 12, 14, 17	9, 13, 15, 16	Moderate	Moderate	No	Moderate	No	Moderate	Strong	Strong
19	Fernández-Braña et al.	Looking beyond the banning of lightweight bags: analysing the role of plastic (and fuel) impacts in waste collection at a Portuguese city	2019	Europe	https://link.springer.com/article/10.1007/s11356-019-05938-w	• Environmental impacts of collection of MSW in the city of Aveiro, Portugal using LCA	N/A	N/A	Neutral	Critical	Neutral	6, 9, **11**, 12	13, 17	Moderate	No	No	Moderate	No	No	Moderate	No
20	Roche Cerasi et al.	Household plastic waste habits and attitudes: A pilot study in the city of Valencia	2021	Europe	https://journals.sagepub.com/doi/10.1177/0734242X21996415	• Understand the people's opinion and attitudes on plastic recycling in the city of Valencia, Spain	N/A	N/A	N/A	Neutral	Neutral	**11**, 12, 17	4, 8, 9, 13, 14, 15	Moderate	Moderate	Moderate	Moderate	No	No	Moderate	No
**Goal 12. Ensure sustainable consumption and production patterns**	**Keywords: Sustainable, Production, Consumption, Footprint, Waste, Efficiency**																			
1	Tang et al.	Toward Infinitely Recyclable Plastics Derived from Renewable Cyclic Esters	2019	Global	https://www.sciencedirect.com/science/article/pii/S2451929418304911	• Review highlighting the plastics derived from cyclic esters along with their properties and recyclability	Neutral	Neutral	N/A	Beneficial	N/A		7, 9, 11, 12, 13, 14, 15	Moderate	No	Moderate	No	No	No	Strong	Moderate
2	Nguyen Thi Khanh Chi	Ethical consumption behavior towards eco-friendly plastic products: Implication for cleaner production	2022	Asia	https://www.sciencedirect.com/science/article/pii/S2666784322000092?via%3Dihub	• Impacts of different concerns and highlighting the role of different social networks towards the ethical consumption of ecofriendly plastic products	Critical	N/A	N/A	Critical	N/A	8, 9, **12**	10, 11, 17	Moderate	Moderate	Moderate	Moderate	No	No	No	No
3	Zhao et al.	Upcycling to Sustainably Reuse Plastics	2021	Global	https://onlinelibrary.wiley.com/doi/10.1002/adma.202100843	• Presenting the latest research on upcycling of plastics including photocatalysis and electrocatalysis	Critical	N/A	N/A	Neutral	N/A	7, 13	9, 12, 14, 15	Moderate	No	Strong	No	No	Strong	Strong	Moderate
4	Shangwa et al.	Conceptualization and design of a small pyrolysis plant for the sustainable production of paraffin from plastic waste	2020	Africa	https://www.sciencedirect.com/science/article/pii/S2212827120308349?via%3Dihub	• Design of a pyrolysis plasnt to recycle the PET plastic wastes into fuel	Critical	Critical	N/A	Neutral	N/A	**12**	3, 7, 9, 13	No	No	No	No	No	No	Strong	No
5	Gong et al.	Energy- and Labor-aware Production Scheduling for Sustainable Manufacturing: A Case Study on Plastic Bottle Manufacturing	2017	Europe	https://www.sciencedirect.com/science/article/pii/S2212827116312963?via%3Dihub	• Empirical energy model for the production of plastic bottle by blow molding	N/A	N/A	N/A	N/A	N/A	9, **12**	7, 13	No	No	Strong	No	No	No	No	No
6	Katie Conolon	Plastic roads: not all they're paved up to be	2022	Global	https://www.tandfonline.com/doi/full/10.1080/13504509.2021.1915406	• Analysis of how global south perceives using plastic to pave roads as a solution but not consider the impacts caused by the use of them	Highly critical	Highly critical	Critical	Highly critical	Highly critical	**12**	3, 9, 10, 11, 13, 14, 15, 16, 17	No	No	No	No	No	No	Strong	No
7	Milios et al.	Sustainability impact assessment of increased plastic recycling and future pathways of plastic waste management in Sweden	2018	Europe	https://www.mdpi.com/2313-4321/3/3/33	• Assessment of sustainability impacts of plastic waste with three future scenarios included	Critical	Critical	N/A	Neutral	Neutral	8, 9, **12**, 13	7, 11, 14, 15, 17	No	Moderate	Moderate	Moderate	No	Moderate	Strong	No
8	Deshpande et al.	Multi-criteria decision analysis (MCDA) method for assessing the sustainability of end-of-life alternatives for waste plastics: A case study of Norway	2020	Europe	https://www.sciencedirect.com/science/article/pii/S0048969720308639?pes=vor	• Assessment of environmental, economic and social impacts of treating waste fishing gears in Norway and a multi criteria decision analysis to rank the EoL options	Critical	N/A	N/A	Highly critical	Critical	8, 9, **12**, 14, 15, 17	7, 13	No	No	No	No	No	Strong	Strong	No
9	Evode et al.	Plastic waste and its management strategies for environmental sustainability	2021	Global	https://www.sciencedirect.com/science/article/pii/S2666016421000645?via%3Dihub	• Discuss the lifecycle of different plastic types across the whole lifecycle	Critical	Critical	N/A	Highly critical	N/A	**12**, 13, 15	3, 11, 14, 17	Moderate	No	No	No	No	No	Moderate	Moderate
10	Lee et al.	Environmental Sustainability Framework for Plastic Waste Management—a Case Study of Bubble Tea Industry in Malaysia	2022	Asia	https://link.springer.com/article/10.1007/s41660-022-00230-w	• Environmental performance of landfill and WTE incineration of plastic wastes using LCA	Critical	Critical	Critical	Neutral	N/A	8, 9, 11, **12**, 13, 17	6, 7, 14, 15	No	No	Moderate	No	Moderate	No	Strong	Moderate
11	Yuan et al.	Sustainability-inspired upcycling of waste polyethylene terephthalate plastic into porous carbon for CO2capture	2022	Asia	https://pubs.rsc.org/en/content/articlelanding/2022/GC/D1GC03600A	• Synthesis of porous carbon from physical and chemical activation from three PET plastic waste as a type of CCS technology and their environmental and economic assessment	N/A	N/A	N/A	Highly beneficial	N/A	8, 9, **12**, 13	11, 14, 15	No	No	Moderate	No	No	No	Strong	Strong
12	Slusarczyk et al.	Solution for sustainable development: Provisions limiting the consumption of disposable plastic carrier bags in Poland	2018	Europe	https://jssidoi.org/jssi/uploads/papers/27/Slusarczyk_Solution_for_sustainable_development_provisions_limiting_the_consumption_of_disposable_plastic_carrier_bags_in_Poland.pdf	• Analysis of different ways to redue the pollution caused by using plastic carry bags and discuss about policies in Europe, especially in Poland	Critical	Critical	Critical	Highly critical	N/A	8, 17	8, 9, 11, 12, 13, 14, 15	No	No	No	Moderate	Moderate	No	Moderate	Moderate
13	Chidambarampadmavathy et al.	Sustainable bio-plastic production through landfill methane recycling	2017	Australia	https://www.sciencedirect.com/science/article/abs/pii/S1364032116311376?via%3Dihub	• Reviews the different plastic disposal approaches, methane capture in landfills and production of biodegradable PHB using methanotrophs from landfills	Critical	Beneficial	N/A	Critical	N/A	7, 9, 11, **12**, 13, 15	6, 8, 14	Strong	No	Moderate	No	No	No	Strong	Moderate
14	Govil et al.	Lignocellulosic feedstock: A review of a sustainable platform for cleaner production of nature's plastics	2020	Global	https://www.sciencedirect.com/science/article/abs/pii/S0959652620325683?via%3Dihub	• Review the valuable R&D activities to biosynthesize PHA using lignocellulosic biomass as potential raw material	Critical	Critical	N/A	Critical	N/A	9, **12**	2, 8, 13, 14, 15	Moderate	No	No	No	No	No	Moderate	No
15	Babayemi et al.	Ensuring sustainability in plastics use in Africa: consumption, waste generation, and projections	2019	Africa	https://enveurope.springeropen.com/articles/10.1186/s12302-019-0254-5	• Historical analysis of polymer consumption and production with the associated pollution potential	Critical	Highly critical	N/A	Highly critical	N/A	10, **12**, 14, 17	3, 7, 8, 9, 11, 13, 15, 16	Moderate	Moderate	Moderate	No	Moderate	Moderate	Moderate	Strong
16	de Vargas Mores et al.	Sustainability and innovation in the Brazilian supply chain of green plastic	2018	South America	https://www.sciencedirect.com/science/article/abs/pii/S0959652617330974?via%3Dihub	• Analyse the innovation process of green plastic (bio-based) through the lens of sustainable supply chain management	Beneficial	N/A	N/A	N/A	Beneficial	9, **12**, 15	2, 11, 13, 14	Moderate	No	Moderate	Moderate	No	No	No	Moderate
17	Burnley et al.	The environmental and financial benefits of recovering plastics from residual municipal waste before energy recovery	2018	Europe	https://www.sciencedirect.com/science/article/abs/pii/S0956053X18304574?via%3Dihub	• LCA and cost effectiveness of different kerbside collections of municipal wastes before energy recovery	N/A	N/A	N/A	Neutral	N/A	9, 11, **12**, 13	7, 8, 10, 14, 15	No	No	No	Moderate	No	No	Strong	No
18	Gerassimidou et al.	Development of an integrated sustainability matrix to depict challenges and trade-offs of introducing bio-based plastics in the food packaging value chain	2021	Global	https://www.sciencedirect.com/science/article/pii/S095965262035424X?via%3Dihub	• A sustainbility decision matrix was created to understand the impacts of changing to bio-based from fossil based plastics	N/A	N/A	N/A	Neutral	N/A	8, **12**, 14, 17	1, 2, 3, 9, 10, 11, 13, 15	Moderate	No	Moderate	No	No	Moderate	Strong	Strong
19	Lombardi et al.	Material flow analysis and sustainability of the Italian plastic packaging management	2021	Europe	https://www.sciencedirect.com/science/article/abs/pii/S0959652620356195?via%3Dihub	• Assessment of post consumer waste recycling rate to measure the level of circular economy in Italy	N/A	N/A	N/A	Neutral	N/A	11, **12**, 17	9, 13, 14, 15	No	No	No	No	No	No	Moderate	Moderate
20	Herberz et al.	Sustainability assessment of a single-use plastics ban	2020	Global	https://www.mdpi.com/2071-1050/12/9/3746	• LCA for single use plastic and non-plastic alternatives were conducted and effects of a plastic ban on marine litter was discussed	Highly critical	Highly critical	N/A	Highly critical	N/A	9, **12**, 14	11, 13, 15, 17	Moderate	Strong	No	No	No	No	Strong	Unclear
21	Briassoulis et al.	Recirculation potential of post-consumer /industrial bio-based plastics through mechanical recycling - Techno-economic sustainability criteria and indicators	2021	Global	https://www.sciencedirect.com/science/article/pii/S014139102030149X?via%3Dihub	• Defines the environmental, TESA (Techno-Economic Sustainability Assessment), and social sustainability criteria for resources, processing and alternative EoL routes for post consumer bio-based plastics	Neutral	N/A	N/A	Neutral	Neutral	9, **12**	2, 3, 8, 13, 14, 15	Moderate	No	No	No	No	No	Moderate	Moderate
22	Borland et al.	Sustainability and sustainable development strategies in the U.K. plastic electronics industry	2019	Europe	https://onlinelibrary.wiley.com/doi/10.1002/csr.1722	• Theoretical insights on the ecocentric ecoinnovations in the electronics industry in UK that contributes to the sustainable development	N/A	N/A	Neutral	Highly critical	N/A	8, 9, **12**, 15, 17	7, 11, 13, 14	No	No	No	No	Moderate	Moderate	Moderate	Unclear
23	Cardamone et al.	About the environmental sustainability of the European management of WEEE plastics	2021	Europe	https://www.sciencedirect.com/science/article/pii/S0956053X21001094?via%3Dihub	• Quantifies the environmental impacts of recovering and disposing WEEE plastic in the environment and argues for the strong reduction fo waste exports	Critical	Critical	N/A	Highly critical	Highly critical	9, **12**	1, 3, 8, 10, 11, 13, 14, 15	Moderate	No	Moderate	Strong	No	No	Strong	No
24	Blanc et al.	Use of bio-based plastics in the fruit supply chain: An integrated approach to assess environmental, economic, and social sustainability	2019	Europe	https://www.mdpi.com/2071-1050/11/9/2475	• Analysis of the environmental, social and economic impacts of replacing the conventional plastics with the bio-based plastics in a fruit supply chain in Italy	N/A	N/A	N/A	Critical	N/A	7, 8, 9, **12**	2, 10, 13, 14	Moderate	No	No	No	No	No	No	No
25	Sundqvist-Andberg et al.	Sustainability governance and contested plastic food packaging – An integrative review	2021	Europe	https://www.sciencedirect.com/science/article/pii/S0959652621013305?via%3Dihub	• Discusses the contestations between different sustainability goals and interest that relate to food packaging and emphasises that the different functions need to be acknolwedged in policy design	Critical	Critical	Critical	Critical	N/A	17	2, 3, 6, 7, 9, 11, 12, 13, 14, 15	Moderate	Moderate	No	No	Moderate	Moderate	Moderate	Moderate
26	Jefferson	WHITHER PLASTICS?—Petrochemicals, plastics and sustainability in a garbage-riddled world	2019	Global	https://www.sciencedirect.com/science/article/abs/pii/S2214629619303172?via%3Dihub	• Significance of plastic wastes, recycling and production of plastics in the environment globally	Critical	Critical	Neutral	Highly critical	N/A	14	3, 8, 9, 10, 11, 12, 13, 15	Moderate	Moderate	Moderate	No	No	Moderate	Strong	Moderate
27	Faisal Kabir et al.	End of life plastics to enhance sustainability of pavement construction utilizing a hybrid treatment of bio-oil and carbon coating	2021	North America	https://www.sciencedirect.com/science/article/abs/pii/S095006182100204X?via%3Dihub	• Construction of pavements using plastic wastes with bio-oil and carbon coating and analysis of their mechanical properties	Critical	Critical	N/A	Neutral	N/A		9, 11, 12, 13, 14, 15	No	Moderate	No	No	No	Moderate	Moderate	Strong
28	Moshood et al.	Sustainability of biodegradable plastics: New problem or solution to solve the global plastic pollution?	2022	Global	https://www.sciencedirect.com/science/article/pii/S2666086522000157?via%3Dihub	• Analysis of the sustainability of bio-degradeable plastics in the environment and framework for their longterm viability	Critical	Critical	N/A	Beneficial	N/A	3, 9, **12**	2, 8, 11, 13, 14, 15	Moderate	No	No	No	No	No	Strong	Moderate
29	Ardolino et al.	How to enhance the environmental sustainability of WEEE plastics management: An LCA study	2021	Europe	https://www.sciencedirect.com/science/article/pii/S0956053X21005109?pes=vor	• Management of plastic wastes from WEEE and the environmental performance of it was caluclated with the help of LCA	Critical	Highly critical	N/A	Highly critical	Critical	9, **12**, 17	1, 3, 6, 7, 8, 11, 13, 14, 15	No	No	No	No	No	No	Moderate	No
30	Mwanza et al.	Major Obstacles to Sustainability in the Plastic Industry	2017	Africa	https://www.sciencedirect.com/science/article/pii/S2351978917300276?via%3Dihub	• An assessment on the obstacles of Plastic solid waste recycling based on the survey conducted in Zambia	N/A	N/A	N/A	Critical	Critical	9, 11	7, 8, 10, 12, 17	No	No	No	No	No	No	Moderate	Strong
**Goal 13. Take urgent action to combat climate change and its impacts**	**Keywords: Climate Change, Global Warming, Mitigation**																			
1	Adyel et al.	Plastics in blue carbon ecosystems: a call for global cooperation on climate change goals	2022	Global	https://www.thelancet.com/journals/lanplh/article/PIIS2542-5196(21)00327-2/fulltext	• Assess how the sequestration capacity of Blue Carbon Ecosystems (Marshlands, Mangroves) affected due to the increase in plastic pollution	Critical	Critical	N/A	Highly critical	Critical		8, 9, 10, 11, 12, 13, 14, 15, 17	No	No	No	No	No	No	Strong	Moderate
2	Ford et al.	The fundamental links between climate change and marine plastic pollution	2022	Global	https://www.sciencedirect.com/science/article/pii/S0048969721054693	• With different overarching ways, the link between the climate change and plastic pollution are concretised	Highly critical	Critical	N/A	Highly critical	Critical	3, 12, **13**, 14	2, 6, 8, 9, 10, 11, 15, 17	Moderate	Moderate	Moderate	No	No	Moderate	Strong	Strong
3	Kane et al.	Reducing the environmental impacts of plastics while increasing strength: Biochar fillers in biodegradable, recycled, and fossil-fuel derived plastics	2022	North America	https://www.sciencedirect.com/science/article/pii/S266668202200226	• Use of bio-char as a filler in the recycled plastics and assessment of their environmnetal and material properties	N/A	N/A	N/A	Neutral	N/A	9, 12, **13**	7, 8, 14, 15	No	Moderate	No	No	No	No	No	Moderate
4	Tiso et al.	The metabolic potential of plastics as biotechnological carbon sources – Review and targets for the future	2022	Global	https://www.sciencedirect.com/science/article/pii/S1096717621001920	• Assessment of biotechnological routes that converts plastic wastes into valuable bio-plasics and biosurfactants	Critical	N/A	N/A	Neutral	N/A	**13**	7, 8, 9, 12, 14, 15	No	No	No	No	No	No	Moderate	Moderate
5	Stegmann et al.	The plastics integrated assessment model (PLAIA): Assessing emission mitigation pathways and circular economy strategies for the plastics sector	2022	Global	https://www.sciencedirect.com/science/article/pii/S221501612200504	• Plastics Integrated Assessment model was introduced, which assess the global interactions of the plastics sector (resource use, circular economy, tradeoffs) and insights on their interactions with other sectors	N/A	N/A	N/A	N/A	N/A	12, **13**	7, 8, 9, 11, 14, 15, 17	No	No	No	No	No	Moderate	Unclear	No
6	Shen et al.	(Micro)plastic crisis: Un-ignorable contribution to global greenhouse gas emissions and climate change	2020	Global	https://www.sciencedirect.com/science/article/abs/pii/S0959652620301852?via%3Dihub	• Proposes measures to solve and alleviate the microplastic crisis by production control on global plastics and improve treatment of plastic waste	Highly critical	Highly critical	N/A	Highly critical	Highly critical	8, 9, 12, **13**, 14	2, 3, 11, 15, 17	Moderate	No	No	No	No	Moderate	Moderate	Strong
7	Cuello et al.	Impact of plastic film mulching on increasing greenhouse gas emissions in temperate upland soil during maize cultivation	2015	Asia	https://www.sciencedirect.com/science/article/pii/S092913931500505?pes=vor	• Plastic film mulching, although increase grain productivity, decreased soil organic matter content and increased the two major GHG emissions when an experimental setup was conducted	N/A	N/A	N/A	Neutral	N/A	2, 8, **13**	11, 12, 14, 15	Moderate	No	No	No	No	No	No	No
8	Sharma et al.	Contribution of plastic and microplastic to global climate change and their conjoining impacts on the environment - A review	2023	Global	https://www.sciencedirect.com/science/article/pii/S004896972312433?via%3Dihub	• Review the impact of plastic pollution on climate change and thereby highlighting the vulnerable effects, measures to tackle them and critically analyze the inter connected problems and mitigation strategies	Highly critical	Highly critical	Neutral	Beneficial	N/A	3, **13**, 14	2, 6, 8, 9, 11, 12, 15, 16, 17	Moderate	No	No	No	No	Moderate	No	Moderate
9	Bora et al.	Waste polypropylene plastic recycling toward climate change mitigation and circular economy: Energy, environmental, and technoeconomic perspectives	2020	North America	https://pubs.acs.org/doi/pdf/10.1021/acssuschemeng.0c06311?src=getftr	• Environmental and economic analysis of two chemical recycling technologies (Pyrolysis and Gasification) and both of them found to have better performance than conventional treatment systems	N/A	N/A	N/A	Neutral	N/A	7, 8, 9, 12, **13**	14, 15	No	No	No	No	No	No	Moderate	No
10	Liu et al.	How does circular economy respond to greenhouse gas emissions reduction: An analysis of Chinese plastic recycling industries	2018	Asia	https://www.sciencedirect.com/science/article/abs/pii/S1364032118302673?via%3Dihub	• Analysis of the circular economy strategies and their effect on chinese plastic recycling industries along with the projection (using IPAT formula) and policy measure to reduce the plastic consumption and pollution	N/A	N/A	N/A	Critical	Neutral	8, 9, 12, **13**, 17	11, 14, 15	No	No	No	No	No	No	Strong	Moderate
11	Zapata	The relationship between climate conditions and consumption of bottled water: A potential link between climate change and plastic pollution	2021	South America	https://www.sciencedirect.com/science/article/abs/pii/S0921800921001488?via%3Dihub	• Discusses on how high temperatures can result in the increase in consumption of bottled water which further exacerbate the climate change	N/A	N/A	Critical	Critical	N/A	6, **13**, 17	1, 3, 8, 9, 10, 11, 12, 16	Moderate	No	No	No	Moderate	No	No	No
12	Nicholson et al.	Manufacturing energy and greenhouse gas emissions associated with plastics consumption	2021	North America	https://www.sciencedirect.com/science/article/pii/S2542435120306267?pes=vor	• Supply chain energy and GHG emissions of the major commodity polymers are estimated and helps in the development of circular economy for synthetic polymers	Critical	N/A	N/A	Critical	N/A	7, 9, **13**	8, 11, 12, 14, 15	Moderate	No	Moderate	No	No	No	Moderate	Moderate
13	Meys et al.	Achieving net-zero greenhouse gas emission plastics by a circular carbon economy	2021	Global	https://www.science.org/doi/10.1126/science.abg9853	• Future projection of net zero emissions in production of plastics with biomass and CO2	Neutral	Neutral	N/A	Neutral	N/A	12, **13**	7, 8, 9, 11, 14, 15, 17	Strong	No	Strong	No	No	No	Strong	Strong
14	Kouloumpis et al.	Potential trade-offs between eliminating plastics and mitigating climate change: An LCA perspective on Polyethylene Terephthalate (PET) bottles in Cornwall	2020	Europe	https://www.sciencedirect.com/science/article/abs/pii/S0048969720321987?via%3Dihub	• Assess the climate chnage impact of potential subsitution of PET by glass using data on consumer behaviour, recycling infrastructure and waste management practices in UK	N/A	N/A	N/A	Critical	Beneficial	9, 12, **13**	8, 11, 14, 15, 16	No	No	No	No	No	Strong	No	Moderate
15	Sevigné-Itoiz et al.	Contribution of plastic waste recovery to greenhouse gas (GHG) savings in Spain	2015	Europe	https://www.sciencedirect.com/science/article/pii/S0956053X1500751?pes=vor	• Quantifying the GHG emissions (with the help of CLCA) and savings due to the recycling and disposal of post consumer plastics in Spain along with the analysis of the recycling of plastics locally and through exports	N/A	N/A	N/A	Critical	Critical	9, 12, **13**, 17	7, 8, 11, 14, 15	No	No	No	Moderate	No	Moderate	Moderate	No
16	Chu et al.	Life-cycle GHG emissions and the associated carbon-peak strategies for PS, PVC, ABS	2022	Asia	https://www.sciencedirect.com/science/article/abs/pii/S0921344922001434?pes=vor	• GHG emissions in the lifecycle of different polymers along with the mitigation strategies and scenario analysis in 2030	Highly critical	Highly critical	N/A	Critical	N/A	9, 12, **13**	7, 8, 11, 14, 15, 16	Strong	No	Moderate	No	No	No	Moderate	Moderate
17	Lee et al.	Impact of plastic film mulching on global warming in entire chemical and organic cropping systems: Life cycle assessment	2021	Asia	https://www.sciencedirect.com/science/article/abs/pii/S095965262101475X?via%3Dihub	• Net GWP for mulching and no mulching in maize cultivation was carried out	Critical	Critical	Beneficial	Neutral	N/A	2, 9, 12, **13**	14, 15	No	No	No	No	Moderate	No	No	No
18	Borui Gu	Research on the Impact of Plastic Recycling Industry on Greenhouse Gas Emissions	2021	Asia	https://iopscience.iop.org/article/10.1088/1755-1315/781/3/032030/pdf	• Input output model was used to quantify the relationship between China's recycling industry and reduction of GHG emissions (Uses IPAT formula to quantify)	N/A	N/A	N/A	Critical	N/A	9, 12, **13**, 17	8, 11, 14, 15, 16	Moderate	No	No	No	No	Unclear	Moderate	Unclear
19	Bauer et al.	Plastics and climate change breaking carbon lock-ins through three mitigation pathways	2022	Global	https://www.sciencedirect.com/science/article/pii/S2590332222001403?pes=vor	• Policy actions to achieve net zero targets for plastic industry by identifying the carbon lock-ins in the lifecycle and other mititgation strategies	N/A	N/A	N/A	Critical	N/A	9, 12, **13**	7, 8, 11, 14, 15, 16, 17	Moderate	No	Strong	No	No	Moderate	Strong	Moderate
20	Liu et al.	How does the global plastic waste trade contribute to environmental benefits: Implication for reductions of greenhouse gas emissions?	2021	Asia	https://www.sciencedirect.com/science/article/pii/S0301479721003455?via%3Dihub	• Addresses the question of whether there has been a reduction in the GHG emissions due to the recycling policies for treating plastic wastes in China	N/A	N/A	N/A	Neutral	Critical	**13**, 14, 17	7, 8, 9, 10, 11, 12, 15, 16	No	No	No	No	No	Strong	Strong	No
21	Tyler Eddy	Climate change drowned out by plastic	2019	Global	https://onlinelibrary.wiley.com/doi/10.1002/aqc.3084	• Argues plastic pollution to be a greater threat to oeans and ecosystems	Critical	Critical	N/A	Highly critical	N/A		11, 12, 13, 14, 15, 16	No	No	No	No	No	Strong	Strong	No
22	Vea et al.	Inclusion of multiple climate tipping as a new impact category in life cycle assessment of polyhydroxyalkanoate (PHA)-based plastics	2021	Europe	https://www.sciencedirect.com/science/article/pii/S0048969721026152?via%3Dihub	• Potential of PHA toward climate change and how it affects climate tipping point, which was introduced as a separte category	N/A	N/A	N/A	Neutral	N/A	9, 12, **13**	7, 11, 14, 15	Strong	No	No	No	No	No	Moderate	Strong
23	Sandberg et al.	Accounting for carbon flows into and from bio plastic in a national climate inventory	2022	Europe	https://onlinelibrary.wiley.com/doi/10.1111/gcbb.13017	• Different approaches to account for stored carbon within plastics using TIMES-Sweden Model based on the data on material stock changes, trade patterns of Sweden, target recycling rates	N/A	N/A	N/A	Neutral	N/A	7, 12, **13**	8, 9, 11, 14, 15, 17	No	No	No	No	No	No	Strong	No

24	Barbara Demeneix	How fossil fuel-derived pesticides and plastics harm health, biodiversity, and the climate	2020	Global	https://www.sciencedirect.com/science/article/pii/S2213858720301169?pes=vor	• How the rise of chemicals and plastics from petrochemical sources threaten biodiverstiy, climate change and health	Highly critical	Highly critical	N/A	Highly critical	N/A	3	2, 7, 8, 9, 10, 11, 12, 13, 14, 15, 16	Strong	Moderate	Moderate	No	No	Moderate	No	No
25	Tickner et al.	Transitioning the Chemical Industry: The Case for Addressing the Climate, Toxics, and Plastics Crises	2021	Global	https://www.tandfonline.com/doi/full/10.1080/00139157.2021.1979857	• Addresses the current state of the chemical industry and the rise of plastic pollution along with the strategies to take corrective actions	Critical	Critical	Neutral	Highly critical	Critical		3, 7, 8, 9, 11, 12, 13, 14, 15, 16, 17	Strong	No	Moderate	No	Moderate	No	Moderate	Strong
26	Karin Kvale	Implications of plastic pollution on global marine carbon cycling and climate	2022	Global	https://portlandpress.com/emergtoplifesci/article/6/4/359/231923/Implications-of-plastic-pollution-on-global-marine	• Review on the global climate impacts, ocean acidifcation and other impacts based on the carbon cycle and plastic pollution	Critical	Critical	N/A	Critical	N/A	13, 14	15, 16, 17	No	Moderate	No	No	No	Moderate	No	No
27	Jankowska et al.	Transforming the Plastic Production System Presents Opportunities to Tackle the Climate Crisis	2022	Global	https://www.mdpi.com/2071-1050/14/11/6539	• Interventions to improve the sustainability and circularity of plastic system	Critical	N/A	N/A	Critical	N/A	12, 13, 14	2, 3, 9, 11, 15, 16, 17	Strong	No	No	No	No	No	Moderate	Moderate
28	Vishnuradhan et al.	Can plastics affect near surface layer ocean processes and climate?	2019	Global	https://www.sciencedirect.com/science/article/abs/pii/S0025326X19300724?via%3Dihub	• Changes in the solar radiation in water column due to the plastics have the potential to affect physical processes and can induce climate feedback cycles	Critical	Critical	N/A	Highly critical	N/A	13, 14	4, 9, 11, 12, 16, 17	No	No	No	No	No	Strong	Strong	Moderate
29	Oktavilia et al.	Plastic Industry and World Environmental Problems	2020	Global	https://www.e3s-conferences.org/articles/e3sconf/abs/2020/62/e3sconf_icenis2020_05020/e3sconf_icenis2020_05020.html	• Regression model that shows the relationship between plastic production and pollution/emissions	Critical	N/A	N/A	Critical	N/A	13	8, 9, 11, 12, 14, 15, 16, 17	No	No	No	No	No	No	Moderate	No
30	An et al.	Estimated material metabolism and life cycle GHG emission of major plastics in China - A commercial sector scale perspective	2022	Asia	https://www.sciencedirect.com/science/article/abs/pii/S092134492200009X?via%3Dihub	• Investigate the environmental impacts caused by three synthetic resins PVC, PP and PE across al stages of lifecycle using an integrated LCA-MFA framework	Critical	Critical	N/A	Highly critical	N/A	9, 12, 13, 17	11, 14, 15, 16	Strong	No	Moderate	No	No	No	Moderate	Moderate
**Goal 14. Conserve and sustainably use the oceans, seas and marine resources for sustainable development**	**Keywords: Marine, Pollution, Coast, Fishing**																				
1	Xanthos et al.	International policies to reduce plastic marine pollution from single-use plastics (plastic bags and microbeads): A review	2017	Global	https://www.sciencedirect.com/science/article/pii/S0025326X17301650?pes=vor	• Reviews international market based strategies and policies to reduce plastic bags and microbeads	Critical	Critical	Highly critical	Highly critical	N/A	14	3, 4, 6, 11, 12, 13, 15, 16, 17	No	Moderate	No	No	No	No	Moderate	Moderate
2	Schnurr et al.	Reducing marine pollution from single-use plastics (SUPs): A review	2018	Global	https://www.sciencedirect.com/science/article/abs/pii/S0025326X18307033?via%3Dihub	• Review highlighting new legislative and non-legislative interventions for the ban of Single Use plastics since 2017 and effectivenss of frameworks to help reduce pollution	Critical	Critical	Highly critical	Highly critical	N/A	14	3, 6, 11, 12, 13, 15, 16, 17	No	Moderate	No	No	No	No	Moderate	Moderate
3	Villarrubia-Gómez et al.	Marine plastic pollution as a planetary boundary threat – The drifting piece in the sustainability puzzle	2018	Global	https://www.sciencedirect.com/science/article/pii/S0308597X17305456?pes=vor	• Reviewing different literature to understand if the marine plastic pollution as a planetary boundary threat	Critical	Critical	Critical	Highly critical	N/A	13, 14	3, 9, 11, 12, 15, 16, 17	No	Moderate	No	No	No	No	Moderate	Moderate
4	Vince et al.	Plastic pollution challenges in marine and coastal environments: from local to global governance	2017	Global	https://onlinelibrary.wiley.com/doi/10.1111/rec.12388	• Governance and policies that are in place to curb plastic pollution in oceans	N/A	N/A	Critical	Highly critical	N/A	14, 16	3, 9, 10, 11, 12, 15, 17	No	Moderate	No	No	No	No	Moderate	Moderate
5	Wilcox et al.	Using expert elicitation to estimate the impacts of plastic pollution on marine wildlife	2016	Global	https://www.sciencedirect.com/science/article/pii/S0308597X15002985?via%3Dihub	• Analysis of the global threats of plastic pollution with the elicitated responses from experts in the field	Critical	Critical	Highly critical	Highly critical	N/A	14, 15	4, 8, 12, 16, 17	No	No	No	No	No	No	Moderate	Moderate
6	Thiel et al.	Impacts of marine plastic pollution from continental coasts to subtropical gyres-fish, seabirds, and other vertebrates in the SE Pacific	2018	Oceania	https://www.frontiersin.org/articles/10.3389/fmars.2018.00238/full	• Distribution of plastic pollution and ingestion/entanglement of plastics by the fauna across the islands in the South East Asia	N/A	N/A	Critical	Highly critical	N/A	14, 15	2, 3, 4, 8, 9, 10, 11, 12, 13, 16, 17	No	No	No	No	No	No	Moderate	Moderate
7	Schmaltz et al.	Plastic pollution solutions: emerging technologies to prevent and collect marine plastic pollution	2020	Global	https://www.sciencedirect.com/science/article/pii/S0160412020320225?pes=vor	• Review to determine the emerging technologies and policies to prevent plastic pollution	N/A	N/A	Critical	Highly critical	N/A	3, 14, 17	6, 8, 9, 11, 12, 15, 16	No	Moderate	Moderate	No	No	No	Moderate	Moderate
8	Andrew Turner	Black plastics: Linear and circular economies, hazardous additives and marine pollution	2018	Global	https://www.sciencedirect.com/science/article/pii/S0160412018302125?via%3Dihub	• Reviews the contemporary literature related to the characterstics, environmental impacts and lifecycle of black plastics, examining regulations related to the disposal of them containing additives	N/A	Critical	Critical	Highly critical	N/A	12, 14	9, 11, 13, 15, 16, 17	Moderate	Moderate	No	No	No	No	Moderate	Moderate
9	Adam et al.	Policies to reduce single-use plastic marine pollution in West Africa	2020	Africa	https://www.sciencedirect.com/science/article/abs/pii/S0308597X19304865?via%3Dihub	• Reviews the Single Use Plastic Policies in the 16 countries in West Africa	Critical	Critical	Neutral	Highly critical	Critical	14, 17	2, 3, 6, 8, 10, 11, 12, 13, 15, 16	No	Moderate	No	No	No	No	Strong	Moderate
10	Lestari et al.	The impact of improper solid waste management to plastic pollution in Indonesian coast and marine environment	2019	Asia	https://www.sciencedirect.com/science/article/abs/pii/S0025326X19306435?via%3Dihub	• Literature review to study the effect of improper solid waste management and plastic pollution in different regions of Indonesia	Critical	Critical	Critical	Highly critical	Critical	11, 12, 14	3, 5, 6, 8, 9, 13, 15, 16, 17	No	Moderate	Moderate	No	No	No	Strong	Moderate
11	Compa et al.	Risk assessment of plastic pollution on marine diversity in the Mediterranean Sea	2019	Europe/Asia	https://www.sciencedirect.com/science/article/abs/pii/S0048969719318984?via%3Dihub	• Identify and estimate the exposure to the plastic ingestion in the Mediterranean sea	N/A	N/A	N/A	Highly critical	Critical	14	3, 11, 12, 15	No	No	No	No	No	No	Moderate	Moderate
12	Oliveira et al.	Marine Environmental Plastic Pollution: Mitigation by Microorganism Degradation and Recycling Valorization	2020	Global	https://www.frontiersin.org/articles/10.3389/fmars.2020.567126/full	• Reviews the important studies on marine plastic pollution along with the mitigation strategies	Neutral	N/A	N/A	Critical	Critical	3, 12, 14	6, 7, 8, 9, 11, 13, 15, 17	Moderate	No	No	No	No	No	Moderate	Moderate
13	Borrelle et al.	Why we need an international agreement on marine plastic pollution	2017	Global	https://www.pnas.org/doi/full/10.1073/pnas.1714450114	• Discusses on the necessity of an international colaboration and agreement for curbing the marine plastic pollution	N/A	N/A	N/A	Highly critical	N/A	14	9, 10, 11, 12, 13, 15, 17	No	No	No	No	No	Moderate	Strong	Moderate
14	Clayton et al.	Policy responses to reduce single-use plastic marine pollution in the Caribbean	2021	Caribbean	https://www.sciencedirect.com/science/article/pii/S0025326X20309516?pes=vor	•Review and analysis of different policyc responses in carribean countries towards reducing plastic pollution	N/A	Critical	Critical	Highly critical	Critical	12, 14, 16	6, 8, 9, 10, 11, 13, 15, 17	Strong	Moderate	No	No	No	Moderate	Moderate	Moderate
15	Akinwumi et al.	Marine plastic pollution and affordable housing challenge: Shredded waste plastic stabilized soil for producing compressed earth bricks	2019	Africa	https://www.sciencedirect.com/science/article/pii/S2214509518303899?pes=vor	• Feasibility study of using waste plastic stabilized soil to produce compressed earth bricks (CEB)	N/A	N/A	N/A	Neutral	N/A	10, 11, 12, 14	9, 13, 15, 17	No	Moderate	No	No	No	No	Moderate	Moderate
16	Manfra et al.	Biodegradable polymers: A real opportunity to solve marine plastic pollution?	2021	Global	https://www.sciencedirect.com/science/article/abs/pii/S0304389421007275?via%3Dihub	• Review on the use of biodegradable polymers as a solution to counter the plastic pollution	Critical	N/A	N/A	Critical	N/A	9, 12, 14	11, 13, 15	Strong	No	No	No	No	No	No	Moderate
17	Ghayebzadeh et al.	Estimation of plastic waste inputs from land into the Caspian Sea: A significant unseen marine pollution	2020	Asia	https://www.sciencedirect.com/science/article/abs/pii/S0025326X19310276?via%3Dihub	• Quantifying the amount of plastics mismanaged in the Caspian Sea countries	N/A	N/A	N/A	Highly critical	N/A	11, 12, 14	13, 15, 17	No	No	No	No	No	No	Moderate	No
18	Marks et al.	The geopolitical economy of Thailand's marine plastic pollution crisis	2020	Asia	https://onlinelibrary.wiley.com/doi/10.1111/apv.12255	• Obstacles and barriers on mitigating marine plastic pollution in Thailand	Critical	Critical	Critical	Highly critical	N/A	3, 12, 14, 17	8, 9, 11, 13, 15, 16	Moderate	Moderate	No	No	No	Moderate	Strong	Strong
19	Chowdhury et al.	Estimating marine plastic pollution from COVID-19 face masks in coastal regions	2021	Global	https://www.sciencedirect.com/science/article/pii/S0025326X21004537?pes=vor	• Estimate annula face mask utilization and plastic pollution from mismanaged masks in the coastal region of 46 countries	N/A	N/A	Beneficial	Highly critical	N/A	3, 11, 12, 14	6, 8, 9, 13, 15, 17	No	No	No	No	No	No	Strong	No
20	Walther et al.	Plastic pollution of four understudied marine ecosystems: a review of mangroves, seagrass meadows, the Arctic Ocean and the deep seafloor	2022	Global	https://portlandpress.com/emergtoplifesci/article/6/4/371/231922/Plastic-pollution-of-four-understudied-marine	• Review focussing on the effect of plastic pollution on understudied marine ecosystems like mangroves, arctic and seagrass	N/A	N/A	Critical	Highly critical	N/A	14	2, 3, 6, 9, 11, 12, 13, 15, 17	No	No	No	No	No	No	Moderate	Moderate
21	Green et al.	Impacts of discarded plastic bags on marine assemblages and ecosystem functioning	2015	Europe	https://pubs.acs.org/doi/10.1021/acs.est.5b00277	• Experiment conducted to assess the impact of conventional and biodegradable plastic carrier bags near shores in Dublin, Ireland	N/A	N/A	N/A	Critical	N/A	12, 14	9, 15	Moderate	No	No	No	No	Moderate	No	No
22	Willis et al.	Cleaner seas - reducing marine pollution	2022	Global	https://link.springer.com/article/10.1007/s11160-021-09674-8	• Identifying current and future practices and policies based on the observations of growing marine pollution with the help of expert review	N/A	Critical	Critical	Highly critical	Critical	3, 9, 14	4, 6, 8, 9, 10, 11, 12, 15, 16, 17	Moderate	Moderate	No	No	Moderate	Moderate	Moderate	Strong
23	Kyriakopoulos et al.	Investigating the Human Impacts and the Environmental consequences of microplastics disposal	2022	Global	https://www.mdpi.com/2071-1050/14/2/828	• Theoretical, technical and planning analysis was developed for investigating the impacts of microplastic disposal on humans and ecosystems	Critical	Critical	N/A	Highly critical	N/A	14	6, 11, 12, 15, 16, 17	Strong	No	No	No	No	Moderate	Moderate	Moderate
24	Wang et al.	Seawater-Degradable Polymers—Fighting the Marine Plastic Pollution	2020	Global	https://onlinelibrary.wiley.com/doi/10.1002/advs.202001121	• Review of the factors that affect the biodegradability of plastics in the marine ecosystems	N/A	N/A	N/A	Neutral	N/A	12, 14	8, 9, 11, 13, 15, 16, 17	Strong	No	No	No	No	No	Moderate	Moderate
25	Issifu et al.	A review of the production, recycling and management of marine plastic pollution	2020	Global	https://www.mdpi.com/2077-1312/8/11/945	• Theoretical and empirical overview of marine plastic pollution and ist effect on marine ecosystem. Also discusses SDGs that are relevant to marine plastic pollution	Critical	Critical	Critical	Highly critical	Critical	8, 14, 17	3, 6, 9, 11, 12, 15	No	Moderate	Moderate	No	No	Moderate	Moderate	No
26	Kwon et al.	Global styrene oligomers monitoring as new chemical contamination from polystyrene plastic marine pollution	2015	Global	https://www.sciencedirect.com/science/article/pii/S0304389415005762?via%3Dihub	• Sampling styrene oligomers from sandy beaches and flow diagram to propose leaching of oligomers and a global contaminant	Highly critical	N/A	N/A	Highly critical	N/A	14	3, 9, 11, 12, 13, 15	Moderate	No	No	No	No	No	Moderate	No
27	Peter Dauvergne	The power of environmental norms: marine plastic pollution and the politics of microbeads	2018	Global	https://www.tandfonline.com/doi/full/10.1080/09644016.2018.1449090	• Discusses the uneven phaseout of microbeads in cosmetic and hygienic products along with the policies and advocacy organisations to phase out microbeads	Critical	Highly critical	Critical	Highly critical	N/A	14	3, 4, 6, 9, 11, 12, 13, 15, 16, 17	Strong	Moderate	No	No	No	No	Moderate	Strong
28	Peter Dauvergne	Why is the global governance of plastic failing the oceans?	2021	Global	https://www.sciencedirect.com/science/article/abs/pii/S0959378017314140?via%3Dihub	• Role of governance and strong institutions to curb the plastic pollution in oceans and other ecosystems	Highly critical	Critical	Critical	Highly critical	Critical	11, 12, 14	8, 9, 10, 13, 15, 16, 17	Moderate	Moderate	No	No	No	Moderate	Strong	Strong
29	Abbott et al.	Reducing marine plastic pollution: Policy insights from economics	2019	Global	https://www.journals.uchicago.edu/doi/10.1093/reep/rez007	• Role of economics and economic policies in mitigating Marine Plastic Pollution	Critical	Critical	Critical	Highly critical	Critical	12, 14, 17	3, 6, 8, 9, 10, 11, 13, 15, 16	Moderate	Moderate	No	No	No	Moderate	Moderate	Moderate
30	Harris et al.	Using citizen science to evaluate extended producer responsibility policy to reduce marine plastic debris shows no reduction in pollution levels	2021	North America	https://www.sciencedirect.com/science/article/pii/S0308597X20309660?via%3Dihub	• Understand the effectiveness of Extended Producer Responsibility for packaging in reducing shoreline Pollution in Canada using citizen science	N/A	N/A	N/A	Critical	Critical	12, 14	9, 11, 13, 15, 17	No	No	No	No	No	Moderate	Strong	No
**Goal 15. Protect, restore and promote sustainable use of terrestrial ecosystems, sustainably manage forests, combat desertification, and halt and reverse land degradation and halt biodiversity loss**	**Keywords: Ecosystem, Soil, Land, Terrestrial, Wildlife, Wetlands**																				
1	Dris et al.	Beyond the ocean: Contamination of freshwater ecosystems with (micro-)plastic particles	2015	Global	https://www.publish.csiro.au/en/EN14172	• Focusses on the methodology of quantifying the contaminants of plastic debris in the freshwater ecosystems	Critical	Critical	N/A	Highly critical	Critical	11, 14	2, 3, 6, 12, 15, 16, 17	No	No	No	No	No	Moderate	Moderate	Moderate
2	Chae et al.	Current research trends on plastic pollution and ecological impacts on the soil ecosystem: A review	2018	Global	https://www.sciencedirect.com/science/article/abs/pii/S0269749117348637?via%3Dihub	• Studied the effect of plastic pollution on the environment especiall on the soil ecosystem	Critical	Critical	N/A	Highly critical	Highly critical	6, 11, 14	2, 3, 9, 12, 13, 15, 17	Strong	No	No	No	No	Moderate	Moderate	Moderate
3	Kumar et al.	Impacts of plastic pollution on ecosystem services, sustainable development goals, and need to focus on circular economy and policy interventions	2021	Global	https://www.mdpi.com/2071-1050/13/17/9963	• Provide background assessment of the adverse effects of plastic pollution on natural ecosystems and the link between SDGs and plastics	Critical	Critical	Critical	Critical	Critical	4, 8, 9, 12, 13, 14, 16, 17	1, 2, 3, 5, 6, 7, 10, 11, 15	Moderate	Moderate	No	Moderate	Moderate	Moderate	Moderate	No
4	Strungaru et al.	Micro- (nano) plastics in freshwater ecosystems: Abundance, toxicological impact and quantification methodology	2019	Global	https://www.sciencedirect.com/science/article/pii/S0165993618304254?via%3Dihub	• Examination of the potential toxicological effects of MP and NP on freshwater biota	Critical	Critical	N/A	Highly critical	Critical		3, 6, 9, 11, 12, 13, 14, 15, 17	No	No	No	No	No	Moderate	Moderate	No
5	Qadeer et al.	Agricultural plastic mulching as a potential key source of microplastic pollution in the terrestrial ecosystem and consequences	2021	Global	https://www.sciencedirect.com/science/article/abs/pii/S092134492100464X?via%3Dihub	• Problems of plastic mulching on the terrestrial ecsystems - A perspective	Critical	Critical	Neutral	Critical	Critical	2	3, 6, 9, 11, 12, 13, 14, 15, 17	Moderate	Moderate	No	No	No	No	Moderate	No
6	Bandopadhyay et al.	Biodegradable plastic mulch films: Impacts on soil microbial communities and ecosystem functions	2018	Global	https://www.frontiersin.org/articles/10.3389/fmicb.2018.00819/full	• Review on the research and impacts of plastic mulch films on soil biological and biogeochemical processes, with special emphasi on biodegradable plastic mulch films	N/A	N/A	N/A	Neutral	N/A	2	14, 15	No	No	No	No	No	No	Moderate	No
7	Koskei et al.	Effects of increased plastic film residues on soil properties and crop productivity in agro-ecosystem	2021	Asia	https://www.sciencedirect.com/science/article/abs/pii/S0304389421004842?via%3Dihub	• Landfill experiment to evaluate and understand the effect of plastic residues from mulching on soil and soil properties	N/A	N/A	N/A	Critical	N/A	8	2, 3, 14, 15	Moderate	No	No	No	No	No	Moderate	No
8	Yu et al.	Micro plastics in soil ecosystem – A review of sources, fate, and ecological impact	2022	Global	http://pse.agriculturejournals.cz/artkey/pse-202201-0001_micro-plastics-in-soil-ecosystem-a-review-of-sources-fate-and-ecological-impact.php	• Comprehensive review of soli pollution due to microplastic	Critical	Critical	N/A	Highly critical	Critical	3, 8	9, 11, 12, 13, 14, 15	Moderate	Moderate	No	No	No	No	Moderate	No
9	Hurley et al.	Plastic waste in the terrestrial environment	2020	Global	https://www.sciencedirect.com/science/article/pii/B9780128178805000074?via%3Dihub	• Review chapter that discusses the different sources and impacts of plastic pollution in the terrestrial environment	Critical	Critical	Critical	Highly critical	Critical	14	2, 3, 6, 8, 9, 10, 11, 12, 13, 15, 16, 17	Moderate	Moderate	No	No	No	Moderate	Moderate	Moderate
10	Astner et al.	Mechanical formation of micro- and nano-plastic materials for environmental studies in agricultural ecosystems	2019	North America	https://www.sciencedirect.com/science/article/abs/pii/S0048969719328141?via%3Dihub	• Development of techniques to study the formation of MP and NP when using PBAT and LDPE plastic mulch film residues	N/A	N/A	N/A	Critical	N/A		3, 11, 12, 14, 15, 16, 17	Moderate	Moderate	No	No	Moderate	No	Moderate	Moderate
11	Azevedo-Santos et al.	Plastic pollution: A focus on freshwater biodiversity	2021	Global	https://link.springer.com/article/10.1007/s13280-020-01496-5	• Evidence of plastic ingestion in different species in the freshwater ecosystem	Critical	Critical	N/A	Beneficial	N/A	6, 14, 15	2, 3, 4, 9, 10, 11, 12, 16, 17	Strong	Moderate	No	No	No	No	Strong	Moderate
12	Schell et al.	Occurrence, Fate and Fluxes of Plastics and Microplastics in Terrestrial and Freshwater Ecosystems	2020	Global	https://link.springer.com/chapter/10.1007/398_2019_40	• Different aspects of plastic pollution, their sources and their impacts on ecosystems	Highly critical	Critical	Critical	Highly critical	Critical	3, 6, 14	2, 4, 8, 9, 10, 11, 12, 13, 15, 16, 17	Strong	Strong	No	No	No	No	Moderate	Moderate
13	Zhao et al.	Fate of plastic film residues in agro-ecosystem and its effects on aggregate-associated soil carbon and nitrogen stocks	2021	Asia	https://www.sciencedirect.com/science/article/abs/pii/S0304389421009183?via%3Dihub	• Landfill investigation on the effect of Bio and LDPE films on soil systems	N/A	N/A	N/A	Highly critical	N/A		2, 12, 14, 15	Strong	No	No	No	No	No	No	No
14	Blettler et al.	Plastic pollution in freshwater ecosystems: macro-, meso-, and microplastic debris in a floodplain lake	2017	South America	https://link.springer.com/article/10.1007/s10661-017-6305-8	• Effects of macro, meso and microplastic pollution on freshwater lakes in parana, Brazil	N/A	N/A	N/A	Critical	N/A	3, 13, 14	12, 15	No	No	No	No	No	No	Moderate	No
15	Malizia et al.	Terrestrial ecologists should stop ignoring plastic pollution in the Anthropocene time	2019	Global	https://www.sciencedirect.com/science/article/abs/pii/S0048969719310228?via%3Dihub	• Argues for considering plastic pollution when talking about the global research and climate change	Critical	N/A	N/A	Highly critical	N/A		3, 6, 11, 12, 14, 15	No	No	No	No	No	No	Moderate	No
16	Fan et al.	A review on the occurrence and influence of biodegradable microplastics in soil ecosystems: Are biodegradable plastics substitute or threat?	2022	Global	https://www.sciencedirect.com/science/article/pii/S0160412022001702?pes=vor	• Summarises the eco-toxicological effects of Biodegradable Microplastics in soil ecosystems	Critical	Critical	N/A	Critical	N/A		2, 3, 8, 9, 12, 14, 15	Strong	Moderate	No	No	No	No	Moderate	Moderate
17	Susanti et al.	Microplastics and the Impact of Plastic on Wildlife: A Literature Review	2020	Global	https://iopscience.iop.org/article/10.1088/1755-1315/528/1/012013	• Impact of microplastics on birds and animals in the environment	Critical	Critical	N/A	Highly critical	N/A	2, 14, 17	3, 6, 11, 12, 13, 15	Strong	No	No	No	No	No	No	No
18	Wang et al.	Uptake, translocation, and biological impacts of micro(nano)plastics in terrestrial plants: Progress and prospects	2022	Global	https://www.sciencedirect.com/science/article/abs/pii/S0013935121011610?via%3Dihub	• Review based on the literature analysis identifying the current knowledge gaps and suggest prospective lines for research	Critical	Critical	N/A	Critical	Critical		2, 3, 12, 14, 15	Strong	No	No	No	No	No	No	No
19	Zhao et al.	Microscopic anthropogenic litter in terrestrial birds from Shanghai, China: Not only plastics but also natural fibers	2016	Asia	https://www.sciencedirect.com/science/article/pii/S0048969716301127?pes=vor	• Microscopic fragements from natural fibres could be more contaminating than microplastics on the birdlife based on a sampling study	Critical	Critical	N/A	Critical	N/A		2, 3, 12, 14, 15	Strong	Moderate	No	No	No	No	Moderate	No
20	Maclean et al.	The terrestrial plastisphere: Diversity and polymer-colonizing potential of plastic-associated microbial communities in soil	2021	Europe	https://www.mdpi.com/2076-2607/9/9/1876	• Discusses about the plastisphere that colonizes the micobial organisms in sil which could aid in the degradation of plastics	N/A	N/A	N/A	N/A	N/A	14	2, 3, 12, 15	No	No	No	No	No	No	No	No

21	Mo et al.	Environmental fate and impacts of biodegradable plastics in agricultural soil ecosystems	2023	Global	https://www.sciencedirect.com/science/article/pii/S0929139322002839?via%3Dihub	• Systematically analyze the environmental fate and impacts of biodregradable plastics in agriculture and propose crucial challenges and prioritzed research in the future.	Critical	Critical	Critical	Highly critical	Critical		2, 3, 9, 12, 14, 15	Strong	Moderate	No	No	No	No	Moderate	No
22	Amaneesh et al.	Gross Negligence: Impacts of Microplastics and Plastic Leachates on Phytoplankton Community and Ecosystem Dynamics	2023	Global	https://pubs.acs.org/doi/full/10.1021/acs.est.2c05817	• Citation Network Analysis to show the impacts and research of Microplastic and Plastic Leachates on the phytoplankton community	Critical	Critical	N/A	Highly critical	Critical	3, 14	2, 8, 9, 11, 12, 15, 16, 17	Moderate	Moderate	No	No	No	No	Moderate	Moderate
23	Guimarães et al.	Toxic effects of naturally-aged microplastics on zebrafish juveniles: A more realistic approach to plastic pollution in freshwater ecosystems	2021	South America	https://www.sciencedirect.com/science/article/abs/pii/S0304389420328247?via%3Dihub	• Toxicity analysis of naturally aged polystyrene microplastics on freshwater ecosystems	N/A	N/A	N/A	Critical	N/A		3, 6, 14, 15	Moderate	No	No	No	No	No	No	No
24	Kasavan et al.	Plastic pollution in water ecosystems: A bibliometric analysis from 2000 to 2020	2021	Global	https://www.sciencedirect.com/science/article/abs/pii/S0959652621021648?via%3Dihub	• Bibliometric analysis on the trends, hotspots and research gaps on plastic pollution in freshwater ecosystems	Critical	Critical	N/A	Highly critical	N/A	14	3, 6, 8, 9, 10, 11, 12, 13, 15	No	No	No	No	No	No	Moderate	No
25	Tudor et al.	The issue of plastic and microplastic pollution in soil	2019	Global	https://revmaterialeplastice.ro/Articles.asp?ID=5214	• Factors that affect the plastic pollution in soil	N/A	Critical	N/A	Critical	N/A	6, 12, 14	2, 3, 15	No	No	No	No	No	No	Moderate	No
26	Ullah et al.	Micro(nano)plastic pollution in terrestrial ecosystem: emphasis on impacts of polystyrene on soil biota, plants, animals, and humans	2023	Global	https://link.springer.com/article/10.1007/s10661-022-10769-3?utm_source=getftr&utm_medium=getftr&utm_campaign=getftr_pilot	• Systematic review on the impact of Polystyrene microplastic pollution in terrestrial environment	N/A	N/A	N/A	Highly critical	N/A	3, 14	2, 4, 6, 9, 11, 12, 15	Moderate	Moderate	No	No	No	Moderate	Moderate	No
27	Zhang et al.	Microplastics pollution from different plastic mulching years accentuate soil microbial nutrient limitations	2022	Asia	https://www.sciencedirect.com/science/article/abs/pii/S1342937X21002434?via%3Dihub	• Analysis of the soil properties for microplastic pollution in a tobacco field in China	N/A	N/A	N/A	Critical	Critical	17	2, 3, 11, 12, 14, 15	Moderate	No	No	No	No	No	Moderate	No
28	Joos et al.	Soil under stress: The importance of soil life and how it is influenced by (micro)plastic pollution	2022	Global	https://www.sciencedirect.com/science/article/pii/S2001037022001167?pes=vor	• Observed effects of plastisphere on the plant-soil-microbiome system	N/A	N/A	N/A	Critical	N/A	2	14, 15	No	No	No	No	No	No	No	No
29	Andrea et al.	Unraveling the role of plastic waste pollution in the Amvrakikos Wetlands National Park, Greece: The stakeholders' views	2020	Europe	https://www.mdpi.com/2077-1312/8/8/549	• Plastic waste management in a national park in Greece and the Stakeholder views on mitigating them	Critical	Critical	Critical	Highly critical	Critical	11, 12, 14, 16	2, 3, 6, 8, 9, 13, 15	Moderate	Moderate	No	No	No	Moderate	Moderate	Moderate
30	Chen et al.	Feasibility of using plastic wastes as constructed wetland substrates and potential for pharmaceuticals and personal care products removal	2020	Asia	https://www.mdpi.com/2077-1312/8/8/549	• Use of plastic wastes as wetland substrates for removal of personal care and pharmaceutcial products and analysed by an experiment uusing different polymers	N/A	Critical	N/A	Neutral	N/A	6, 12	2, 3, 14, 15	No	No	No	No	No	Moderate	Moderate	Moderate
**Goal 16. Promote peaceful and inclusive societies for sustainable development, provide access to justice for all and build effective, accountable and inclusive institutions at all levels**	**Keywords: Justice, Peace, Regulation, Legislation, Governance, Policy**																				
1	Katie Conolon	Adaptive injustice: Responsibility to act in the plastics economy	2020	Global	https://www.sciencedirect.com/science/article/abs/pii/S0921344919304690?via%3Dihub	• Social awareness and th concept of adaptive injustice is discussed wherein the orgnaisations take no responsibility for the plastic wastes they produce	Highly critical	Highly critical	Critical	Highly critical	Critical		10, 11, 12, 16, 17	Strong	Moderate	No	No	No	Moderate	Strong	Strong
2	Kim et al.	Designing for Green and Grey: Insights from Single-Use Plastic Water Bottles	2022	Asia	https://www.mdpi.com/1660-4601/19/3/1423	• Evaluating the openability of bottles with inclusive designs for the elderly in South Korea	Critical	Highly critical	Critical	Critical	N/A	9, 10, 17	6, 11, 12, 16	No	Moderate	Strong	No	No	No	No	Strong
3	Bahl et al.	Don't Transfer Waste, Transform Waste: A Sustainable Approach towards Zero Waste Events Initiative during Hockey World Cup in Bhubaneshwar, India	2021	Asia	https://iopscience.iop.org/article/10.1088/1755-1315/795/1/012018	• Efficient collection and sorting of plastic wastes in a hockey world cup in Bhubaneshwar, India	Critical	N/A	Neutral	Beneficial	Beneficial	10, 11, 12, 17	8, 9, 16	No	No	No	No	No	No	Strong	Strong
4	Garcia et al.	Marine Plastic Pollution in Asia: All Hands on Deck!	2019	Asia	https://brill.com/view/journals/cjel/3/1/article-p11_2.xml	• Government efforts to minimise the marine plastic pollution are studied for the countries of China and Indonesia	Critical	N/A	Critical	Highly critical	Critical	14	8, 9, 10, 11, 12, 15, 16, 17	Moderate	Moderate	No	No	Strong	No	Strong	Strong
5	Liboiron et al.	Abundance and types of plastic pollution in surface waters in the Eastern Arctic (Inuit Nunangat) and the case for reconciliation science	2021	North America	https://www.sciencedirect.com/science/article/abs/pii/S0048969721018775?via%3Dihub	• Insights and governance on how to measure and categorize plastic particles in arctic	Highly critical	N/A	Highly critical	Highly critical	Highly critical	14	10, 11, 12, 15, 16, 17	Strong	Strong	No	Moderate	No	No	Strong	Strong
6	Paul Jobin	Our 'good neighbor' Formosa Plastics: petrochemical damage(s) and the meanings of money	2020	Asia	https://www.tandfonline.com/doi/full/10.1080/23251042.2020.1803541	• Analyses the practices of the petrochemical company Formosa through interviews when challenged by a lawsuit	Highly critical	Highly critical	Critical	Highly critical	N/A	3, **16**	5, 6, 11, 13, 14, 15	Moderate	Moderate	Moderate	No	No	No	Moderate	Strong
7	Hsu et al.	Closing the loop on plastics in Europe - The role of data, information and knowledge	2022	Europe	https://www.sciencedirect.com/science/article/pii/S2352550922002251?via%3Dihub	• Through interviews with stakeholders, understanding how information and knowledge of makrets for secondary plastics can be improved in Europe	Critical	Critical	Critical	Highly critical	Critical		8, 9, 11, 12, 16, 17	Moderate	Strong	No	No	No	Moderate	Moderate	Strong
8	Poto et al.	Suggestions for a systematic regulatory approach to ocean plastics	2021	Global	https://www.mdpi.com/2304-8158/10/9/2197	• Investigate the problems to better improve regulatory process of ocean plastics	Critical	N/A	N/A	Highly critical	N/A	2, 14	3, 12, 16, 17	Moderate	Moderate	No	No	No	No	Moderate	Strong
9	Owens et al.	Mopping Up or Turning Off the Tap? Environmental Injustice and the Ethics of Plastic Pollution	2021	Global	https://www.frontiersin.org/articles/10.3389/fmars.2021.713385/full	• Discusses the disproportionate responsibility falling on the global south when it comes to the plastic pollution	Critical	Critical	Highly critical	Highly critical	Highly critical	14	1, 2, 3, 6, 10, 11, 12, 16, 17	Moderate	Moderate	No	No	No	No	Moderate	Strong
10	Wang et al.	Circular Economy and the Changing Geography of International Trade in Plastic Waste	2022	Global	https://www.mdpi.com/1660-4601/19/22/15020	• Discuss about the economic policies in plastics trade and the regulations to keep it in a transparent and sustainable manner	Highly critical	Highly critical	Highly critical	Highly critical	Highly critical	8, 10, 12, 14, 17	9, 13, 15, 16	Moderate	Moderate	No	Strong	No	No	Strong	Strong
11	Margaret Handley	Confronting the plasticine: promise in a world wrapped in plastic	2022	South America	https://www.sciencedirect.com/science/article/pii/S2542519622001942?via%3Dihub	• Effects of plastic pollution in the rivers of Guatemela	Highly critical	N/A	N/A	Highly critical	N/A		6, 9, 11, 12, 14, 15, 16, 17	No	No	No	Strong	No	No	Strong	Strong
12	Shirley et al.	Unwrapping Victoria's general environmental duty to plastics communities: Synthetic statutes	2022	Australia	https://journals.sagepub.com/doi/10.1177/1037969X221111596	• Plastic pollution in Victoria from the lens of environmental justice	Critical	Critical	N/A	Highly critical	N/A	13	10, 11, 12, 16, 17	No	No	No	Strong	No	No	Strong	Strong
13	Alice Mah	Future-proofing capitalism: The paradox of the circular economy for plastics	2021	Global	https://direct.mit.edu/glep/article/21/2/121/97367/Future-Proofing-Capitalism-The-Paradox-of-the	• Role of corporations in global governance of plastic pollution	Critical	Critical	N/A	Highly critical	Critical	9, 12, 14, 17	10, 11, 13, 16	Strong	No	No	Strong	No	Moderate	Strong	Strong
14	Ortiz et al.	A Regional Response to a Global Problem: Single Use Plastics Regulation in the Countries of the Pacific Alliance	2020	South America	https://www.mdpi.com/2071-1050/12/19/8093	• An illustrative case study on the national and subnational regulation on the consumption and managment of plastic bags for Mexico, Colombia, Chile, Peru and Ecuador	Critical	Critical	N/A	Highly critical	N/A		12, 14, 15, 16, 17	Moderate	Moderate	No	No	No	No	Strong	Strong
15	Carlini et al.	Advancing the international regulation of plastic pollution beyond the united nations environment assembly resolution on marine litter and microplastics	2018	Global	https://onlinelibrary.wiley.com/doi/10.1111/reel.12258	• Discusses the role of United Nations in combating marine plastic pollution	Critical	Critical	N/A	Highly critical	Critical	12, 14	11, 15, 16, 17	No	No	No	No	No	No	Strong	Moderate
16	Steensgaard et al.	From macro- to microplastics - Analysis of EU regulation along the life cycle of plastic bags	2017	Europe	https://www.sciencedirect.com/science/article/abs/pii/S026974911631898X?via%3Dihub	• Identification of different regulatory frameworks with respect to the life cycle of plastic carrier bags	Highly critical	Highly critical	Critical	Highly critical	Critical	6, 12, 14	8, 9, 10, 11, 13, 15, 16, 17	Moderate	Moderate	No	Moderate	No	Moderate	Strong	Strong
17	Tan et al.	Moving policy and regulation forward for single-use plastic alternatives	2021	Global	https://link.springer.com/article/10.1007/s11783-021-1423-5	• Review of the regulative frameworks for the single use plastic products alternatives	Critical	Critical	N/A	Critical	N/A	4, 8, 9, **16**, 17	12, 14, 15	Moderate	No	No	No	No	No	Moderate	Strong
18	Tham Hoang	Plastic pollution: Where are we regarding research and risk assessment in support of management and regulation?	2022	Global	https://setac.onlinelibrary.wiley.com/doi/10.1002/ieam.4627	• Analysis of the information adequacy for the research in plastic pollution	Critical	N/A	N/A	Highly critical	N/A		12, 13, 14, 15, 16, 17	Moderate	Moderate	No	No	No	No	Strong	Strong
19	Saputra et al.	Combining the concept of green accounting with the regulation of prohibition of disposable plastic use	2021	Asia	https://econjournals.com/index.php/ijeep/article/view/10087	• Qualitative research that emphasizes an indepth understanding of green accounting after prohibition of plastics	Critical	Critical	Highly critical	Highly critical	Critical	12	6, 8, 9, 10, 11, 14, 15, 16, 17	Moderate	Moderate	No	No	No	No	Strong	Strong
20	Tessnow-von Wysocki et al.	Plastics at sea: Treaty design for a global solution to marine plastic pollution	2019	Global	https://www.sciencedirect.com/science/article/abs/pii/S1462901119301364?via%3Dihub	• Identification of treat design to curb plastics at seaand improve the success of future mechanisms	Critical	Critical	N/A	Highly critical	N/A	13, 14	12, 15, 16, 17	Strong	Moderate	No	No	No	No	Moderate	Strong
**Goal 17. Strengthen the means of implementation and revitalize the global partnership for sustainable development**	**Keywords: Partnership, Cooperation, Global, Trade, Market**																				
1	Bank et al.	Global plastic pollution observation system to aid policy	2021	Global	https://pubs.acs.org/doi/pdf/10.1021/acs.est.1c00818?src=getftr	• Argument for a global monitoring system for plastic pollution needed for sustainability assessment. A need for evidence based policy is required.	Highly critical	Highly critical	N/A	Highly critical	N/A	8, 11, 14, 15, **17**	9, 10, 12, 13, 16	No	No	No	No	No	No	Strong	Strong
2	Marcus Haward	Plastic pollution of the world's seas and oceans as a contemporary challenge in ocean governance	2018	Global	https://www.nature.com/articles/s41467-018-03104-3.pdf	• Review of the united nations meeting on plastic pollution and policy governance to prevent marine plastic pollution	Highly critical	Highly critical	N/A	Highly critical	Critical	14	12, 15, 16, 17	No	No	No	No	No	No	Strong	Strong
3	Freeman et al.	Between source and sea: The role of wastewater treatment in reducing marine microplastics	2020	Global	https://www.sciencedirect.com/science/article/pii/S030147972030574O?pes=vor	• Current state of knowledge on the role WWTP can play in reducing marine plastic pollution	Highly critical	Highly critical	N/A	Highly critical	N/A	6, 9, 14	8, 10, 11, 12, 16, 17	No	No	No	No	No	No	Strong	Moderate
4	Duong et al.	A Model Template Green Environment Initiative for Recycling plastic bottles with progressive entrepreneurship partnership	2018	Asia	https://ieeexplore.ieee.org/document/8481786	• Propse solutions for environmental friendly solutions recovering waste lastic and recycling bottles	Critical	N/A	N/A	Beneficial	N/A	14	8, 9, 11, 12, 17	No	No	Strong	No	No	Moderate	Strong	No
5	Kandziora et al.	The important role of marine debris networks to prevent and reduce ocean plastic pollution	2019	Global	https://www.sciencedirect.com/science/article/abs/pii/S0025326X19300499?via%3Dihub	• Achievments, challenges and role of national and regional marine plastic debris networks all over the world to curb plastic pollution	Highly critical	Highly critical	N/A	Highly critical	N/A	11, 12, 14, **17**	13, 15	No	No	No	No	No	No	Strong	Strong
6	Li et al.	The collapse of global plastic waste trade: Structural change, cascading failure process and potential solutions	2021	Global	https://www.sciencedirect.com/science/article/abs/pii/S0959652621021533?via%3Dihub	• Cascading failure model to understand the overloading capacity of plastic waste across different countries	Highly critical	Highly critical	Critical	Highly critical	Highly critical	3, 10, 12, **17**	1, 8, 9, 11, 14, 15, 16	No	No	No	No	No	No	Strong	Strong
7	Galaiduk et al.	Transnational Plastics: An Australian Case for Global Action	2020	Australia	https://com-mendeley-prod-publicsharing-pdfstore.s3.eu-west-1.amazonaws.com/eef8-CC-BY-2/10.3389/fenvs.2020.00115.pdf?X-Amz-Security-Token=IQoJb3JpZ2luX2VjEBAaCWV1LXdlc3QtMSJHMEUCIEYC9WYI6Rjd9W%2FCLrlYu1sZCkKcmcEa81OHs0gBigcUAiEAlyLyY3PdTNydsr%[illegible]	• Sources of drifting ocean plastics that enters and leaves Australia	Highly critical	Highly critical	N/A	Highly critical	Highly critical	9, 12, 14, **17**	3, 6, 11, 15	No	No	No	No	No	No	Strong	Strong
8	Barrowclough et al.	Transforming the Global Plastics Economy: The Role of Economic Policies in the Global Governance of Plastic Pollution	2022	Global	https://www.mdpi.com/2076-0760/11/1/26	• Literature review and findings on the global governance process on plastic pollution	Highly critical	Highly critical	N/A	Highly critical	Highly critical	3, 9, 12, 13, 14	6, 8, 10, 11, 15, 16, 17	Strong	Moderate	Moderate	Moderate	No	Strong	Strong	Strong
9	Kuan et al.	Towards regional cooperation on sustainable plastic recycling: comparative analysis of plastic waste recycling policies and legislations in Japan and Malaysia	2022	Asia	https://link.springer.com/article/10.1007/s10098-021-02263-0	• Comparative analysis of different plastic recycling policies and legislations between Malysia and Indonesia	Highly critical	N/A	N/A	Highly critical	Critical	12, **17**	7, 8, 9, 10, 11, 13, 14, 15	Moderate	No	No	No	No	No	Strong	Strong
10	Finska et al.	Troubled waters - Where is the bridge? Confronting marine plastic pollution from international watercourses	2018	Global	https://onlinelibrary.wiley.com/doi/epdf/10.1111/reel.12257?src=getftr	• Possibilites for more integrated measures to prevent polltion	Highly critical	N/A	N/A	Highly critical	Highly critical	6, 14	8, 9, 10, 11, 12, 15, 17	Strong	No	No	No	No	Moderate	No	Strong
11	Sun et al.	Regional cooperation in marine plastic waste cleanup in the south china sea region	2021	Asia	https://www.mdpi.com/2071-1050/13/16/9221	• Simulation of a cooperation model to investigate the cleanup system to reduce plastic pollution	Highly critical	N/A	N/A	Highly critical	N/A	10, 14, **17**	11, 12, 13, 15	Strong	No	No	No	No	No	Strong	Strong
12	Nancy Lamontagne	Academic-Industry Partnerships: Preparing Tomorrow's Plastics Professionals	2015	North America	https://onlinelibrary.wiley.com/doi/10.1002/j.1941-9635.2015.tb01281.x	• Analysis of how collaborations between plastics industry and engineering programs	N/A	Beneficial	N/A	N/A	N/A		4, 7, 8, 9, 12, 17	Moderate	No	Moderate	No	No	No	No	No
13	Stuchtey et al.	Project stop: City partnerships to prevent ocean plastics in Indonesia	2019	Asia	https://journals.openedition.org/factsreports/5409	• How Project STOP helps in reducing plastic pollution through several measures	Highly critical	N/A	N/A	Critical	Neutral	8, 11, 12, 14, **17**	9, 10, 15	No	No	No	No	No	Strong	Strong	Strong
14	Raubenheimer et al.	Rethinking global governance of plastics – The role of industry	2020	Global	https://www.sciencedirect.com/science/article/abs/pii/S0308597X19309698?via%3Dihub	• Conceptualising a global EPR scheme to control plastic pollution	Critical	N/A	N/A	Highly critical	N/A	9, 12, 14	8, 10, 11, 15, 16, 17	No	No	No	No	No	Strong	Strong	Moderate
15	Fadeeva et al.	'Unlocking circular economy for prevention of marine plastic pollution: An exploration of G20 policy and initiatives'	2021	Global	https://www.sciencedirect.com/science/article/pii/S030147972013827?via%3Dihub	• Highlights existing policies and identifies further policy measures for MPP merging Circular Economy and Life Cycle Perspectives	Highly critical	Critical	N/A	Highly critical	N/A	8, 12, 14	9, 13, 15, 16, 17	Strong	Moderate	Moderate	No	No	Strong	Strong	Strong
16	Nielsen et al.	Politics and the plastic crisis: A review throughout the plastic life cycle	2020	Global	https://wires.onlinelibrary.wiley.com/doi/10.1002/wene.360	• Literature review on life cycle of plastics and plastic objects normally addressed in the current literature	Highly critical	Highly critical	Critical	Highly critical	N/A	8, 14	9, 10, 11, 12, 13, 15, 16, 17	Strong	Strong	No	No	No	Moderate	Strong	Strong
17	Shruti et al.	Strengthening citizen science partnerships with frontline sanitation personnel to study and tackle plastic pollution	2022	Global	https://www.sciencedirect.com/science/article/pii/S1462901122002519?pes=vor	• Exploring the need and advantages of having sanitary personnel into citizen science to monitor the plastic pollution	Critical	Critical	N/A	Highly critical	Critical		3, 8, 9, 10, 11, 12, 14, 15, 17	No	No	No	No	No	No	Strong	Strong
18	Micah Landon-Lane	Corporate social responsibility in marine plastic debris governance	2018	Global	https://www.sciencedirect.com/science/article/abs/pii/S0025326X17310111?via%3Dihub	• Discuss how implementing CSR in combination with governmental frameworks would work effective against plastic pollution	Critical	N/A	Critical	Highly critical	N/A	14	9, 12, 13, 15, 16, 17	Moderate	Strong	Moderate	No	No	Strong	Strong	Strong
19	Shah et al.	Plastics waste metabolism in a Petro-Island state: Towards solving a "wicked problem" in trinidad and tobago	2019	North America	https://www.mdpi.com/2071-1050/11/23/6580	• Institutional factors that shape the solutions for the plastic pollution problem	Critical	N/A	N/A	Highly critical	N/A	12, **17**	8, 9, 11, 13, 14, 15	No	No	Moderate	No	No	Moderate	Strong	Strong
20	Bagai et al.	Beating plastic pollution: UNEP's priorities and partnership in India	2021	Asia	https://content.iospress.com/articles/environmental-policy-and-law/epl201069	• Highlights the magnitude of plastic pollution and role of UNEP in India	Critical	N/A	N/A	Critical	N/A	12, 14, **17**	9, 11, 15	Strong	Strong	No	No	No	No	Strong	Moderate

Scoring of the impacts of plastics based on qualitative assessment							
Studies considered for the qualitative assessment			Impacts of plastics on the achievement of SDGs (+3 - Highly beneficial, +2 - Beneficial, +1 - Neutral, 0 - N/A, -2 - Critical, -3 - Highly critical)<br>For SDG with 10 Publications: Total Above -20 - Highly critical; -1 to - 20 - Critical; -1 to 1 - Neutral; 1 to 20 - Beneficial; Above 20 - Highly beneficial<br>For SDG with 20 Publications: Above -40 - Highly critical; -1 to - 40 - Critical; -1 to 1 - Neutral; 1 to 40 - Beneficial; Above 40 - Highly beneficial<br>For SDG with 30 Publications: Above -60 - Highly critical; -1 to - 60 - Critical; -1 to 1 - Neutral; 1 to 60 - Beneficial; Above 60 - Highly beneficial				
S.No.	Author	Title of the study	Raw material	Production	Use	End of Life	Transportation/Logistics
Goal 1. End poverty in all its forms everywhere	Keywords: Poverty, People, Population						
1	Mpanangombe et al.	Poverty, politics and plastic - Organic waste sorting in Blantyre's public markets	-2	-2	-2	-3	-2
2	David Katz	Plastic Bank: launching Social Plastic® revolution	-2	0	0	1	1
3	Mukama et al.	Practices, Concerns, and Willingness to Participate in Solid Waste Management in Two Urban Slums in Central Uganda	-2	0	-2	-3	-3
4	Gall et al.	Building a circular plastics economy with informal waste pickers: Recyclate quality, business model, and societal impacts	-2	0	0	-2	-2
5	El Mekaoui et al.	Plastic bags ban and social marginalization: Evidence from Morocco	2	2	2	-2	0
6	Jenks et al.	The poverty of plastics bans: Environmentalism's win is a loss for disabled people	2	2	2	-2	0
7	Stoler et al.	Piped water flows but sachet consumption grows: The paradoxical drinking water landscape of an urban slum in Ashaiman, [illegible]	0	0	2	-2	2
8	Velis et al.	Enabling the informal recycling sector to prevent plastic pollution and deliver an inclusive circular economy	-2	-2	-2	-2	1
9	Obiadi Bons N. Onochie A.C	The -2 impacts of poverty in urban and rural architecture in Nigeria	2	0	2	2	0
10	Klocker et al.	From troublesome materials to fluid technologies: Making and playing with plastic-bag footballs	-3	-2	-2	2	0
Total			-7	-2	0	-11	-3
Goal 2. End hunger, achieve food security and improved nutrition and promote sustainable agriculture	Keywords: Agriculture, Hunger, Food, Food security						
1	He et al.	Plastic mulch: Tradeoffs between productivity and greenhouse gas emissions	-3	-3	1	-3	-3
2	Saeed et al.	Competency of groundwater recharge of irrigated cotton field subjacent to sowing methods, plastic mulch, water productivity, and yield under climate change	-2	0	-2	0	0
3	Wang et al.	Plastic mulching reduces nitrogen footprint of food crops in China: A meta-analysis	2	0	2	0	0
4	Castillo-Diaz et al.	The management of agriculture plastic waste in the framework of circular economy. Case of the almeria greenhouse (Spain)	-2	0	1	-3	-2
5	Batista et al.	Dealing with Plastic Waste from Agriculture Activity	-2	0	-2	-2	0
6	Pazienza et al.	For a new plastics economy in agriculture: Policy reflections on the EU strategy from a local perspective	-2	-2	-3	-3	1
7	Chen et al.	Response of carbon footprint to plastic film mulch application in spring maize production and mitigation strategy	-3	-3	1	-3	0
8	Xie et al.	Yield, economic benefit, soil water balance, and water use efficiency of intercropped maize/potato in responses to mulching practices on the semiarid loess plateau	2	0	2	-2	0
9	Zhang et al.	Plastic pollution in croplands threatens long-term food security	-2	-2	2	-3	0
10	Lwanga et al.	Field evidence for transfer of plastic debris along a terrestrial food chain	-3	-3	-3	-3	-3
11	Ruimin et al.	Behavior of microplastics and plastic film residues in the soil environment: A critical review	-2	-2	1	-3	-2
12	Chen et al.	Effects of plastic film combined with straw mulch on grain yield and water use efficiency of winter wheat in Loess Plateau	2	2	3	0	0
13	Hu et al.	Comparative analysis of carbon footprint between conventional smallholder operation and innovative largescale farming of urban agriculture in Beijing, China	-2	-2	-2	-3	1
14	Gao et al.	Effects of plastic mulching and plastic residue on agricultural production: A meta-analysis	2	2	1	-3	1
15	Brodhagen et al.	Policy considerations for limiting unintended residual plastic in agricultural soils	2	0	1	-3	1
16	Stubenrauch et al.	Plastic Pollution in Soils: Governance Approaches to Foster Soil Health and Closed Nutrient Cycles	-2	-2	-3	-3	-2
17	Edet et al.	Impact of "sachet water" microplastic on agricultural soil physicochemistry, antibiotics resistance, bacteria diversity and function	0	0	-2	-3	0
18	Sun et al	Contamination of phthalate esters, organochlorine pesticides and polybrominated diphenyl ethers in agricultural soils from the Yangtze River Delta of China	-3	-3	1	-3	-2
19	Xucheng et al.	Did plastic mulching constantly increase crop yield but decrease soil water in a semiarid rain-fed area?	0	0	2	-2	0

20	Cozzolino et al.	Appraisal of biodegradable mulching films and vegetal-derived biostimulant application as eco-sustainable practices for enhancing lettuce crop performance and nutritive value	1	0	0	2	0
**Total**			**-17**	**-18**	**1**	**-43**	**-10**
**Goal 3. Ensure healthy lives and promote well-being for all at all ages**	**Keywords: Health, Death, Illness, Disease, Mortality**						
1	Metcalf et al.	Quantifying the importance of plastic pollution for the dissemination of human pathogens: The challenges of choosing an appropriate 'control' material	-3	-3	-2	-3	-3
2	Bouwmeester et al.	Potential Health Impact of Environmentally Released Micro- and Nanoplastics in the Human Food Production Chain: Experiences from Nanotoxicology	-3	-3	-3	-3	-2
3	Pramod Kumar	Role of Plastics on Human Health	-2	-3	-3	-2	0
4	Li et al.	Environmental risks of disposable face masks during the pandemic of COVID-19: Challenges and management	0	-3	2	-3	-3
5	Yuan et al.	Human health concerns regarding microplastics in the aquatic environment - From marine to food systems	-3	-3	0	-3	-3
6	Landrigan et al.	Human health and ocean pollution	-3	-3	0	-3	-2
7	Groh et al.	Overview of known plastic packaging-associated chemicals and their hazards	-2	-3	0	-3	0
8	Yee et al.	Impact of microplastics and nanoplastics on human health	-3	-3	-3	-3	0
9	Leslie et al.	Discovery and quantification of plastic particle pollution in human blood	-3	-3	0	-3	0
10	Jiang et al.	Health impacts of environmental contamination of micro- And nanoplastics: A review	-3	-3	0	-3	0
11	Adebiyi-Abiola et al.	Cleaning up plastic pollution in Africa	-2	0	1	-2	0
12	Yang et al.	Waste management, informal recycling, environmental pollution and public health	-2	-2	-2	-3	-2
13	Hahladakis et al.	An overview of chemical additives present in plastics: Migration, release, fate and environmental impact during their use, disposal and recycling	-3	-3	-2	-3	0
14	Gasperi et al.	Microplastics in air: Are we breathing it in?	-2	0	-3	-3	0
15	Barboza et al.	Marine microplastic debris: An emerging issue for food security, food safety and human health	-2	0	-2	-3	0
16	Mason et al.	Synthetic Polymer Contamination in Bottled Water	-3	-3	0	-3	0
17	Wright et al.	Plastic and Human Health: A Micro Issue?	-3	-3	0	-3	0
18	Fred-Ahmadu et al.	Interaction of chemical contaminants with microplastics: Principles and perspectives	-3	-3	0	-3	0
19	Velis et al.	Mismanagement of Plastic Waste through Open Burning with Emphasis on the Global South: A Systematic Review of Risks to Occupational and Public Health	-3	-3	0	-3	0
20	Tang et al.	Polybrominated diphenyl ethers (PBDEs) and heavy metals in road dusts from a plastic waste recycling area in north China: implications for human health	-3	-3	0	-3	-2
**Total**			**-51**	**-50**	**-17**	**-58**	**-17**
**Goal 4. Ensure inclusive and equitable quality education and promote lifelong learning opportunities for all**	**Keywords: Education, Skills, Qualification**						
1	Chmielewski et al.	Microplastic in the environment. The role of education in raising social awareness of the handling of plastic waste	-3	-3	1	1	-2
2	Mironenko et al.	Education Against Plastic Pollution: Current Approaches and Best Practices	-2	-2	1	1	0
3	Bennett et al.	Informing the public and educating students on plastic recycling	-3	-3	1	1	0
4	Dalu et al.	Is awareness on plastic pollution being raised in schools? Understanding perceptions of primary and secondary school educators	-2	0	1	-2	0
5	Kowasch et al.	Circular economy, cradle to cradle and zero waste frameworks in teacher education for sustainability	-2	0	1	1	0
6	Jimenez et al.	'It's my responsibility': perspectives on environmental justice and education for sustainability among international school students in Singapore	0	0	2	-2	0
7	Situmorang et al.	The difference of knowledge and behavior of college students on plastic waste problems	-2	0	1	-3	0
8	Hammami et al.	Survey on awareness and attitudes of secondary school students regarding plastic pollution: implications for environmental education and public health in Sharjah city, UAE	-2	-2	-3	-3	0
9	Parejo et al.	Plastics as an educational resource for sustainable development: A case study in Ghana	-3	-2	1	-3	-2
10	Phan Hoang et al.	Measuring the effect of environmental education for sustainable development at elementary schools: A case study in Da Nang city, Vietnam	-2	0	0	-2	0

11	Khanam et al.	Knowledge, attitude and practice on uses of plastic products, their disposal and environmental pollution: A study among school-going adolescents	-3	0	-3	-3	0
12	Barra et al.	Sustainable chemistry challenges from a developing country perspective: Education, plastic pollution, and beyond	-3	0	0	-3	0
13	So et al.	Environmental education in primary schools: A case study with plastic resources and recycling	-2	0	-2	-2	-2
14	Cheung et al.	A train-the-trainer design for green ambassadors in an environmental education programme on plastic waste recycling	-3	-2	1	1	0
15	Chow et al.	Plastic waste problem and education for plastic waste management	-2	-2	1	-2	0
16	Soares et al.	On the path to minimize plastic pollution: The perceived importance of education and knowledge dissemination strategies	-3	0	-2	-3	0
17	Mandrikas et al.	In-service Teachers' Needs and Mentor's Practices in Applying a Teaching–Learning Sequence on Nanotechnology and Plastics in Primary Education	0	0	0	-2	0
18	Lopez-Fernandez et al.	How Can Socio-scientific Issues Help Develop Critical Thinking in Chemistry Education? A Reflection on the Problem of Plastics	-2	0	1	-2	0
19	du Bois et al.	Design Against the Plastic Soup - The Effect of Small Product Designs in Sustainable Design Education	-2	-2	-2	-3	-2
20	Schiffer et al.	Microplastics Outreach Program: A Systems-Thinking Approach to Teach High School Students about the Chemistry and Impacts of Plastics	-2	-2	0	-2	0
**Total**			**-43**	**-20**	**0**	**-32**	**-8**
**Goal 5. Achieve gender equality and empower all women and girls**	**Keywords: Gender, Women, Equality, Policy**						
1	De La Pena et al.	Empowering Women Thru Plastics Recycling: A Livelihood Project for Women in Marginalized Communities	-2	0	0	2	0
2	Braun et al.	Plastic Bags, Pollution, and Identity: Women and the Gendering of Globalization and Environmental Responsibility in Mali	-2	0	1	-3	-2
3	Chatterjee et al.	Green chemistry – Remedy to societal hygiene: A graphical review	-2	0	-2	-2	0
4	Anne Marie Hanson	Women's environmental health activism around waste and plastic pollution in the coastal wetlands of Yucatán	-3	0	-2	-3	0
5	Li et al.	Is Female a More Pro-Environmental Gender? Evidence from China	-2	0	-2	-2	0
6	Lange et al.	Understanding the Socio-Demographic Profile of Waste Re-Users in a Suburban Setting in South Africa	-2	0	-2	-2	0
7	Chukwuone et al.	Determinants of household's waste disposal practices and willingness to participate in reducing the flow of plastics into the ocean: [illegible]	-2	-2	-3	-3	-2
8	Robert Grace	Overcoming Gender Bias in Design	0	0	-2	-3	0
9	Muralidharan et al.	The Role of Guilt in Influencing Sustainable Pro-Environmental Behaviors among Shoppers	-2	-2	-3	-2	0
10	Mikołajewska et al.	Bisphenol A – Application, sources of exposure and potential risks in infants, children and pregnant women	-2	-3	-2	-3	-2
11	Gao et al.	Feminine Hygiene Products - A Neglected Source of Phthalate Exposure in Women	-2	-3	-2	0	0
12	Valvi et al.	Variability and predictors of urinary phthalate metabolites in Spanish pregnant women	-2	-3	-3	0	0
13	Anne Marie Hanson	Women's ecological oral histories of recycling and development in coastal Yucatán	-3	0	-2	-3	0
14	Norfaryanti et al.	Environmental-friendly food products' packaging: Women's purchasing preferences	-3	0	-3	-3	-2
15	Bula Sirika Wayessa	Prepared in pots, served in plastics: Rural Ethiopian women's responses to the global economy	-2	-2	-3	0	-2
16	Muposhi et al.	Is the use of green shopping bags gendered? Evidence from a gender equality conscious emerging market	-3	-2	-2	-2	0
17	Alharbi et al.	Use of Plastics with Hot Food among Saudi Pregnant Women Is Associated with Increased Concentrations of A1C, Thyroid-Stimulating [illegible]	-3	-3	-3	0	0
18	Chowdhury et al.	WASH and MHM experiences of disabled females living in Dhaka slums of Bangladesh	-2	0	1	-2	0
19	Anusha et al.	Ready-to-eat food consumption practices, food safety knowledge and relation to gender and education level of consumers in Kuala Lumpur, [illegible]	0	0	2	0	0
20	Trowbridge et al.	Fossil fuel is the common denominator between climate change and petrochemical exposures, and effects on women and [illegible]	-3	-3	-2	-3	-2
**Total**			**-42**	**-23**	**-34**	**-34**	**-12**
**Goal 6. Ensure availability and sustainable management of water and sanitation for all**	**Keywords: Water, Sanitation, Drinking, Scarcity**						
1	Mihai et al.	Plastic Pollution, Waste Management Issues, and Circular Economy Opportunities in Rural Communities	-3	-3	-2	-3	-2
2	Yeasmin et al.	Piloting a low-cost hardware intervention to reduce improper disposal of solid waste in communal toilets in low-income settlements in Dhaka, Bangladesh	0	0	2	1	0
3	Geetha T	Endocrine disruptors in boiled drinking water carried in plastic containers: a pilot study in Thrissur, Kerala, India	-2	-2	-3	0	0
4	Liu et al.	Do estrogenic compounds in drinking water migrating from plastic pipe distribution system pose adverse effects to human? An analysis of scientific literature	-2	-3	-3	0	-3
5	Manjaya et al.	Informally vended sachet water: Handling practices and microbialwater quality	-3	-2	-3	0	0

6	Barletta et al.	Distribution, sources and consequences of nutrients, persistent organic pollutants, metals and microplastics in South American estuaries	-3	-3	-2	-3	-2
7	Haris et al.	Installing public handwashing facilities and integrating them with water fountains to reduce plastic pollution and prevent spread of infections	-2	0	-2	-3	0
8	Dorji et al.	On-site domestic wastewater treatment system using shredded waste plastic bottles as biofilter media: Pilot-scale study on effluent standards in Bhutan	-3	-3	0	1	0
9	Szczotko et al.	Evaluation of susceptibility of polymer and rubber materials intended into contact with drinking water on biofilm formation	0	-2	0	0	0
10	Alda-Vidal et al.	"Unflushables": Establishing a global agenda for action on everyday practices associated with sewer blockages, water quality, and plastic pollution	-2	-2	-2	-3	0
11	Egun et al.	Beat the plastic: an approach to polyethylene terephthalate (PET) bottle waste management in Nigeria	-3	-2	-3	-3	-2
12	Winter et al.	Women's sanitation practices in informal settlements: A multi-level analysis of factors influencing utilisation in Nairobi, Kenya	0	0	2	0	0
13	O Briain et al.	The role of wet wipes and sanitary towels as a source of white microplastic fibres in the marine environment	-3	-2	-2	-3	0
14	Borde et al.	Community challenges when using large plastic bottles for Solar Energy Disinfection of Water (SODIS)	1	-2	2	0	0
15	Diehl et al.	Do-it-Yourself (DIY) Workspaces Run by Local Entrepreneurs that Transform Plastic Waste into Valuable Water and Sanitation Products	-2	-2	-2	2	0
16	Necibi et al.	Contaminants of emerging concern in african wastewater effluents: Occurrence, impact and removal technologies	-2	0	0	-3	0
17	Taduswan et al.	Microplastic contamination in a conventional wastewater treatment plant in Thailand	-3	-3	0	-3	0
18	Muanda et al.	Factors and impacts of informal settlements residents' sanitation practices on access and sustainability of sanitation services in the policy context of free basic sanitation	0	0	2	0	0
19	Semey et al.	Characteristics of packaged water production facilities in greater Accra, Ghana: Implications for water safety and associated environmental impacts	0	-2	0	-2	0
20	Tang et al.	Different senescent HDPE pipe-risk: brief field investigation from source water to tap water in China (Changsha City)	-2	-3	1	-2	0
**Total**			**-34**	**-36**	**-15**	**-24**	**-9**
**Goal 7. Ensure access to affordable, reliable, sustainable and modern energy for all**	**Keywords: Energy, Renewable, Sustainable, Clean**						
1	Dong et al.	Design of special plastic bearings and their application in renewable energy conversion system	2	2	3	0	0
2	Stephen et al.	Completing the value chain for plastic recyclers in Nigeria: An integration of renewable solar and conventional gas energy sources for fuel production	-2	-2	0	2	0
3	Sagariga et al.	Plastic to Fuel Conversion System Using Renewable Energy Assisted Pyrolysis	-2	-2	0	2	0
4	Daniel Posen et al.	Greenhouse gas mitigation for U.S. plastics production: Energy first, feedstocks later	1	1	0	1	0
5	Gebre et al.	Recent Trends in the Pyrolysis of Non-Degradable Waste Plastics	-2	-2	0	2	0
6	Gug et al.	Processing and properties of a solid energy fuel from municipal solid waste (MSW) and recycled plastics	-2	-2	0	2	0
7	Sharuddin et al.	Pyrolysis of plastic waste for liquid fuel production as prospective energy resource	-2	-2	0	2	0
8	Lombardi et al.	A review of technologies and performances of thermal treatment systems for energy recovery from waste	0	0	0	2	0
9	Wong et al.	Current state and future prospects of plastic waste as source of fuel: A review	-2	-2	0	2	2
10	Sharuddin et al.	A review on pyrolysis of plastic wastes	0	0	0	2	0
11	Lam et al.	Microwave vacuum pyrolysis of waste plastic and used cooking oil for simultaneous waste reduction and sustainable energy conversion: Recovery of cleaner liquid fuel and techno	0	0	0	2	0
12	Shen et al.	Waste-to-energy: Dehalogenation of plastic-containing wastes	-2	-2	0	2	0
13	Chandrasekeran et al.	Materials and Energy Recovery from E-Waste Plastics	-2	-2	0	2	-2
14	Bukkarapu et al.	Management, conversion, and utilization of waste plastic as a source of sustainable energy to run automotive: a review	-2	-2	0	2	0
15	Devasahayam et al.	Review: Opportunities for simultaneous energy/materials conversion of carbon dioxide and plastics in metallurgical processes	-2	-2	0	2	0
16	Surenderan et al.	Characterization studies on waste plastics as a feedstock for energy recovery in Malaysia	-2	-2	0	1	0
17	Lim et al.	Optimal sorting and recycling of plastic waste as a renewable energy resource considering economic feasibility and environmental pollution	-2	-2	0	1	0
18	Willenbacher et al.	Machine learning for optimization of energy and plastic consumption in the production of thermoplastic parts in SME	0	2	0	0	0
19	Olazabal et al.	From plastic waste to new materials for energy storage	-2	0	0	2	0
20	Nugraho et al.	Plastic waste as an alternative energy	-2	-2	0	2	0
**Total**			**-25**	**-21**	**3**	**33**	**0**

Goal 8. Promote sustained, inclusive and sustainable economic growth, full and productive employment and decent work for all	Keywords: Economy, Job, Growth, Employment						
1	Umeda et al.	Potential impacts of the European Union's circular economy policy on Japanese manufacturers	0	0	0	-2	0
2	Andreoni et al.	Polyethylene recycling: Waste policy scenario analysis for the EU-27	0	0	0	-2	0
3	Nara et al.	Expected impact of industry 4.0 technologies on sustainable development: A study in the context of Brazil's plastic industry	0	1	0	1	0
4	Vimal et al.	Analysis of barriers that impede the elimination of single-use plastic in developing economy context	-2	1	0	-3	0
5	Rossi et al.	Circular economy indicators for organizations considering sustainability and business models: Plastic, textile and electro-electronic cases	0	0	0	0	0
6	Wu et al.	Supporting a circular economy: Insights from Taiwan's plastic waste sector and lessons for developing countries	-3	-2	-2	-3	0
7	Carola Guyot Phung	Implications of the circular economy and digital transition on skills and green jobs in the plastics industry	0	0	0	2	0
8	Hofmann et al.	The value chain and activities of polyethylene terephthalate plastics in the South African waste economy	-2	0	0	1	0
9	Bala et al.	Identifying the prospects of decent job creation along the value chain of plastic recycling	0	0	0	1	0
10	Bening et al.	The true cost of solving the plastic waste challenge in developing countries: The case of Ghana	0	0	0	1	0
11	Bai et al.	Ecologically unequal exchange of plastic waste? A longitudinal analysis of international trade in plastic waste	-2	-2	0	2	-2
12	Torres et al.	The need for technical improvement in the plastics recycling industry in middle-income countries: The Peruvian case	0	0	0	-2	-2
13	Cordier et al.	Plastic pollution and economic growth: The influence of corruption and lack of education	-3	-2	0	-3	0
14	Stuart J. Barnes	Understanding plastics pollution - The role of economic development and technological research	-3	-3	0	2	-3
15	Wu et al.	Impact of strategic control and supply chain management on recycled plastic additive manufacturing	0	2	0	0	2
16	Silva et al.	Simulation of elements of industry 4.0 applied to the production processes of a company dedicated to the manufacture of plastic products	0	2	0	0	0
17	Babarinsa et al.	Potential Socio-economic impact of replacing traditional woven baskets with reusable plastic crates on livelihoods of basker makers in southeast Nigeria	2	2	1	2	2
18	Alvarado-Diaz et al	Design of a Plastic Shredding Machine to Obtain Small Plastic Waste	-3	-3	0	1	0
19	Arabi et al.	Impacts of marine plastic on ecosystem services and economy: State of South African research	-3	-3	0	-3	0
20	Adedeji Adelodun	Plastic Recovery and Utilization: From Ocean Pollution to Green Economy	-3	-3	0	-3	0
**Total**			-22	-10	-1	-8	-3

Goal 9. Build resilient infrastructure, promote inclusive and sustainable industrialization and foster innovation	Keywords: Innovation, Infrastructure, Research, Industry						
1	Moshood et al.	Biodegradable plastic applications towards sustainability: A recent innovations in the green product	1	0	0	1	0
2	Raddadi et al.	Biodegradation of oil-based plastics in the environment: Existing knowledge and needs of research and innovation	-3	-3	0	-2	0
3	Getor et al.	The role of technological innovation in plastic production within a circular economy framework	-3	-2	0	-2	0
4	Junior et al.	Knowledge-based dynamic capabilities for sustainable innovation: The case of the green plastic project	0	0	0	0	0
5	Nel et al.	Collaboration and infrastructure is needed to develop an African perspective on micro(nano)plastic pollution	-3	-3	0	-3	0
6	Friedrich et al.	Supporting the development process for building products by the use of research portfolio analysis: A case study for wood plastics composite materials	0	0	2	0	0
7	Sommer et al.	Recycling and recovery infrastructures for glass and carbon fiber reinforced plastic waste from wind energy industry: A European case study	0	0	0	-2	0
8	Gong et al.	Investigation into circular economy of plastics: The case of the UK fast moving consumer goods industry	-2	0	0	-2	0
9	Dijkstra et al.	In the business of dirty oceans: Overview of startups and entrepreneurs managing marine plastic	-2	-2	0	1	0
10	Siltaloppi et al.	Toward a sustainable plastics value chain: Core conundrums and emerging solution mechanisms for a systemic transition	-2	-2	0	-3	0
11	Cheng et al.	Investigating the Economic Feasibility of Community-Scale Plastic Recycling Facilities	-2	-2	0	1	0
12	Bing et al.	Global reverse supply chain redesign for household plastic waste under the emission trading scheme	-2	-2	0	-2	-2
13	Muposhi et al.	Considerations, benefits and unintended consequences of banning plastic shopping bags for environmental sustainability: A systematic literature review	-3	-3	1	-3	-2
14	Knoblauch et al.	Government policies combatting plastic pollution	-2	-2	-3	-3	-2
15	Rivera-Huerta et al.	Innovation in the informal sector: The case of plastic recycling firms in Mexico	0	0	0	1	0
16	Oyake-Ombis et al.	Managing plastic waste in East Africa: Niche innovations in plastic production and solid waste	-3	-2	-2	-2	-2
17	Cordier et al.	How much innovation is needed to protect the ocean from plastic contamination?	-3	-3	-2	-3	-2
18	Huang et al.	Evolution of network relations, enterprise learning, and cluster innovation networks: the case of the Yuyao plastics industry cluster	0	1	0	0	0
19	Oyinlola et al.	Digital innovations for transitioning to circular plastic value chains in Africa	-3	-3	0	-3	0
20	Ishartomo et al.	Learning from plastic waste village in Boyolali Indonesia: SME-based plastic recycling industries	-2	-2	-2	-3	-2
21	Conchubhair et al.	Joint effort among research infrastructures to quantify the impact of plastic debris in the ocean	-3	-3	0	-3	0
22	Ma et al.	Economic evaluation of infrastructures for thermochemical upcycling of post-consumer plastic waste	-2	-2	0	1	0

23	Hinton et al.	Innovations Toward the Valorization of Plastics Waste	-3	-3	0	-3	0
24	Nancy Lamontagne	Recycling the Unrecyclable Industry innovations aim to keep traditionally difficult-to-recycle plastics out of the landfill	-2	-2	0	1	1
25	Vollmer et al.	Beyond Mechanical Recycling: Giving New Life to Plastic Waste	-2	-2	0	1	0
26	Muposhi et al.	Embedding Ecopreneurial Behaviour: Proposed Social Marketing Interventions From Value-In-Behaviour Perceptions of Plastic Waste Ecopreneurs	-3	0	0	-3	0
27	da Silva et al.	Evolution toward environment sustainable behavior: search for survival in the plastic industry in Brazil	-2	-2	0	-3	0
28	Ana Espada	Design as a source of innovation to establish circular models: An opportunity to prevent the single use of plastic	-2	-2	-2	-2	-2
29	Chow et al.	Research and development of a new waste collection bin to facilitate education in plastic recycling	0	0	-2	-2	-2
30	Reddy et al.	Review of the Utilization of Plastic Wastes as a Resource Material in Civil Engineering Infrastructure Applications	-2	-2	0	1	0
**Total**			**-55**	**-48**	**-10**	**-41**	**-15**
**Goal 10. Reduce inequality within and among countries**	**Keywords: Income, Injustice, Inequality, Social**						
1	Velis et al.	Plastic pollution global treaty to cover waste pickers and open burning?	-3	-3	-2	-3	-2
2	Benedetta Cotta	What goes around, comes around? Access and allocation problems in Global North–South waste trade	-3	-3	0	-3	-3
3	Samantha Chisholm Hatfiel	Plastic suffocation: Climate change threatens indigenous populations and traditional ecological knowledge	-3	-3	-3	-3	-2
4	Horvath et al.	Designing Business Solutions for plastic waste management to enhance circular transitions in Kenya	-3	-2	-2	-2	-3
5	Horne et al.	High-rise plastic: Socio-material entanglements in apartments	0	0	3	-2	0
6	Jamie Furniss	Alternative framings of transnational waste flows reflections based on the Egypt China PET plastic	0	0	0	0	-2
7	Borman et al.	Impact of plastic packaging design on the sustainability of plastic recyclers	-2	-2	1	-3	0
8	Saskia Abrahms-Kavunenk	Toward an anthropology of plastics	-3	-3	1	-3	0
9	Nicolas Schlitz	Environmental change and the informal plastic recycling networks of Kolkata	-2	-2	0	-2	1
10	Hossain et al.	Socioeconomic Relation with Plastic Consumption on 61 Countries Classified by Continent, Income Status and Coastal Regions	-2	-2	0	2	0
11	Kumar et al.	Estimation of the generation rate of different types of plastic wastes and possible revenue recovery from informal recycling	-2	0	0	-2	1
12	Gareiou et al.	Awareness of Citizens for the Single-Use Plastics: Comparison between a High-Income and an Upper-Middle-Income Economy of the Easter Mediterranean Region, Greece and Lebanon	-2	0	0	-2	-2
13	Knoblauch et al.	Developing countries in the lead-what drives the diffusion of plastic bag policies?	-2	0	0	-2	-2
14	Muisa Zikali et al.	Household solid waste handling practices and recycling value for integrated solid waste management in a developing city in Zimbabwe	0	0	0	-2	-2
15	Karasik et al.	Inequitable distribution of plastic benefits and burdens on economies and public health	1	1	1	1	1
16	Navarre et al.	Recycled plastic packaging from the Dutch food sector pollutes Asian oceans	1	-2	2	-3	2
17	Abalansa et al.	The marine plastic litter issue: A social-economic analysis	-2	-2	-2	-3	-2
18	Cook et al.	Scaling up resource recovery of plastics in the emergent circular economy to prevent plastic pollution: Assessment of risks to health and safety in the Global South	-2	-2	-2	-2	0
19	Chowdhury et al.	The ecological impact of plastic pollution in a changing climate	-3	-3	0	-3	0
20	Stuart J. Barnes	Out of sight, out of mind: Plastic waste exports, psychological distance and consumer plastic purchasing	-2	-2	-2	-3	-2
**Total**			**-34**	**-30**	**-5**	**-40**	**-17**
**Goal 11. Make cities and human settlements inclusive, safe, resilient, and sustainable**	**Keywords: Cities, Municipal, Waste, Communities, Settlements**						
1	Venkatesh et al.	Microbial degradation of plastics: Sustainable approach to tackling environmental threats facing big cities of the future	1	-2	0	-2	0
2	Adam et al.	Attitudinal and behavioural segments on single-use plastics in Ghana: Implications for reducing marine plastic pollution	-2	-2	1	-2	0
3	Varkey et al.	Identifying barriers to reducing single-use plastic use in a coastal metropolitan city in Canada	-2	-2	1	-2	-2
4	Hossain et al.	Strategies for mitigating plastic wastes management problem: A lifecycle assessment study in Hong Kong	0	0	0	-2	0
5	Dong et al.	Uncovering opportunity of low-carbon city promotion with industrial system innovation: Case study on industrial symbiosis projects in China	0	0	0	1	0
6	Wardrop et al.	Estimation of packaged water consumption and associated plastic waste production from household budget surveys	-2	-2	0	2	0
7	Abrokwah et al.	Drivers of single-use plastic waste generation: lessons from packaged water consumers in Ghana	0	0	2	-2	0
8	Asari et al.	Analysis of mismanaged plastic waste in Samoa to suggest proper waste management in Pacific island countries	-2	0	0	-3	-2
9	Salhofer et al.	Plastic recycling practices in vietnam and related hazards for health and the environment	-2	-2	0	-3	-2
10	Agusningtyas et al.	Processing of plastic waste from Klotok Landfill Kediri City with thermal cracking method	-2	0	0	1	0
11	Kabir et al.	Plastic recycling from municipal solid waste in Jashore city of Bangladesh	0	0	0	1	-2
12	Muslihun et al.	An Environmental Study on the Paid Plastic Bag Use Policy in the city of Semarang	0	0	-3	-3	0
13	Cohen et al.	Parks and Recreational Areas as Sinks of Plastic Debris in Urban Sites: The Case of Light-Density Microplastics in the City of Amsterdam, The Netherlands	0	0	-2	-3	-2
14	Schuyler et al.	Environmental context and socio-economic status drive plastic pollution in Australian cities	0	0	-2	-3	-2
15	Correa et al.	Challenges to reducing post-consumer plastic rejects from the MSW selective collection at two MRFs in São Paulo city, Brazil	-2	-2	0	-3	-2

16	Pertiwi et al.	Preliminary Study on Plastic Waste Handling in Semarang City - Indonesia: Estimated Generation and Existing Management	-2	-2	0	-3	-2
17	Taha Ahmed Al-Tayyar	Characteristics of Plastic Solid Wastes in Mosul City and Their Reuse	-2	-2	0	-2	0
18	Brooks et al.	The Chinese import ban and its impact on global plastic waste trade	-2	-2	0	-3	-2
19	Fernández-Braña et al.	Looking beyond the banning of lightweight bags: analysing the role of plastic (and fuel) impacts in waste collection at a Portuguese city	0	0	1	-2	1
20	Roche Cerasi et al.	Household plastic waste habits and attitudes: A pilot study in the city of Valencia	0	0	0	1	1
**Total**			**-19**	**-18**	**-2**	**-32**	**-16**
**Goal 12. Ensure sustainable consumption and production patterns**	**Keywords: Sustainable, Production, Consumption, Footprint, Waste, Efficiency**						
1	Tang et al.	Toward Infinitely Recyclable Plastics Derived from Renewable Cyclic Esters	1	1	0	2	0
2	Nguyen Thi Khanh Chi	Ethical consumption behavior towards eco-friendly plastic products: Implication for cleaner production	-2	0	0	-2	0
3	Zhao et al.	Upcycling to Sustainably Reuse Plastics	-2	0	0	1	0
4	Shangwa et al.	Conceptualization and design of a small pyrolysis plant for the sustainable production of paraffin from plastic waste	-2	-2	0	1	0
5	Gong et al.	Energy- and Labor-aware Production Scheduling for Sustainable Manufacturing: A Case Study on Plastic Bottle Manufacturing	0	0	0	0	0
6	Katie Conolon	Plastic roads: not all they're paved up to be	-3	-3	-2	-3	-3
7	Milios et al.	Sustainability impact assessment of increased plastic recycling and future pathways of plastic waste management in Sweden	-2	-2	0	1	1
8	Deshpande et al.	Multi-criteria decision analysis (MCDA) method for assessing the sustainability of end-of-life alternatives for waste plastics: A case study of Norway	-2	0	0	-3	-2
9	Evode et al.	Plastic waste and its management strategies for environmental sustainability	-2	-2	0	-3	0
10	Lee et al.	Environmental Sustainability Framework for Plastic Waste Management—a Case Study of Bubble Tea Industry in Malaysia	-2	-2	-2	1	0
11	Yuan et al.	Sustainability-inspired upcycling of waste polyethylene terephthalate plastic into porous carbon for CO2capture	0	0	0	3	0
12	Slusarczyk et al.	Solution for sustainable development: Provisions limiting the consumption of disposable plastic carrier bags in Poland	-2	-2	-2	-3	0
13	idambarampadmavathy et	Sustainable bio-plastic production through landfill methane recycling	-2	2	0	-2	0
14	Govil et al.	Lignocellulosic feedstock: A review of a sustainable platform for cleaner production of nature's plastics	-2	-2	0	-2	0
15	Babayemi et al.	Ensuring sustainability in plastics use in Africa: consumption, waste generation, and projections	-2	-3	0	-3	0
16	de Vargas Mores et al.	Sustainability and innovation in the Brazilian supply chain of green plastic	2	0	0	0	2
17	Burnley et al.	The environmental and financial benefits of recovering plastics from residual municipal waste before energy recovery	0	0	0	1	0
18	Gerassimidou et al.	Development of an integrated sustainability matrix to depict challenges and trade-offs of introducing bio-based plastics in the food packaging value chain	0	0	0	1	0
19	Lombardi et al.	Material flow analysis and sustainability of the Italian plastic packaging management	0	0	0	1	0
20	Herberz et al.	Sustainability assessment of a single-use plastics ban	-3	-3	0	-3	0
21	Briassoulis et al.	Recirculation potential of post-consumer /industrial bio-based plastics through mechanical recycling - Techno-economic sustainability criteria and indicators	1	0	0	1	1
22	Borland et al.	Sustainability and sustainable development strategies in the U.K. plastic electronics industry	0	0	1	-3	0
23	Cardamone et al.	About the environmental sustainability of the European management of WEEE plastics	-2	-2	0	-3	-3
24	Blanc et al.	Use of bio-based plastics in the fruit supply chain: An integrated approach to assess environmental, economic, and social sustainability	0	0	0	-2	0
25	Sundqvist-Andberg et al.	Sustainability governance and contested plastic food packaging – An integrative review	-2	-2	-2	-2	0
26	Jefferson	WHITHER PLASTICS?—Petrochemicals, plastics and sustainability in a garbage-riddled world	-2	-2	1	-3	0
27	Faisal Kabir et al.	End of life plastics to enhance sustainability of pavement construction utilizing a hybrid treatment of bio-oil and carbon coating	-2	-2	0	1	0
28	Moshood et al.	Sustainability of biodegradable plastics: New problem or solution to solve the global plastic pollution?	-2	-2	0	2	0
29	Ardolino et al.	How to enhance the environmental sustainability of WEEE plastics management: An LCA study	-2	-3	0	-3	-2
30	Mwanza et al.	Major Obstacles to Sustainability in the Plastic Industry	0	0	0	-2	-2
**Total**			**-36**	**-31**	**-6**	**-26**	**-8**
**Goal 13. Take urgent action to combat climate change and its impacts**	**Keywords: Climate Change, Global Warming, Mitigation**						
1	Adyel et al.	Plastics in blue carbon ecosystems: a call for global cooperation on climate change goals	-2	-2	0	-3	-2
2	Ford et al.	The fundamental links between climate change and marine plastic pollution	-3	-2	0	-3	-2
3	Kane et al.	Reducing the environmental impacts of plastics while increasing strength: Biochar fillers in biodegradable, recycled, and fossil-fuel derived plastics	0	0	0	1	0
4	Tiso et al.	The metabolic potential of plastics as biotechnological carbon sources – Review and targets for the future	-2	0	0	1	0
5	Stegmann et al.	The plastics integrated assessment model (PLAIA): Assessing emission mitigation pathways and circular economy strategies for the plastics sector	0	0	0	0	0
6	Shen et al.	(Micro)plastic crisis: Un-ignorable contribution to global greenhouse gas emissions and climate change	-3	-3	0	-3	-3
7	Cuello et al.	Impact of plastic film mulching on increasing greenhouse gas emissions in temperate upland soil during maize cultivation	0	0	0	1	0
8	Sharma et al.	Contribution of plastic and microplastic to global climate change and their conjoining impacts on the environment - A review	-3	-3	1	2	0
9	Bora et al.	Waste polypropylene plastic recycling toward climate change mitigation and circular economy: Energy, environmental, and technoeconomic perspectives	0	0	0	1	0
10	Liu et al.	How does circular economy respond to greenhouse gas emissions reduction: An analysis of Chinese plastic recycling industries	0	0	0	-2	1

11	Zapata	The relationship between climate conditions and consumption of bottled water: A potential link between climate change and plastic pollution	0	0	-2	-2	0
12	Nicholson et al.	Manufacturing energy and greenhouse gas emissions associated with plastics consumption	-2	0	0	-2	0
13	Meys et al.	Achieving net-zero greenhouse gas emission plastics by a circular carbon economy	1	1	0	1	0
14	Kouloumpis et al.	Potential trade-offs between eliminating plastics and mitigating climate change: An LCA perspective on Polyethylene Terephthalate (PET) bottles in Cornwall	0	0	0	-2	2
15	Sevigné-Itoiz et al.	Contribution of plastic waste recovery to greenhouse gas (GHG) savings in Spain	0	0	0	-2	-2
16	Chu et al.	Life-cycle GHG emissions and the associated carbon-peak strategies for PS, PVC, ABS	-3	-3	0	-2	0
17	Lee et al.	Impact of plastic film mulching on global warming in entire chemical and organic cropping systems: Life cycle assessment	-2	-2	2	1	0
18	Borui Gu	Research on the Impact of Plastic Recycling Industry on Greenhouse Gas Emissions	0	0	0	-2	0
19	Bauer et al.	Plastics and climate change breaking carbon lock-ins through three mitigation pathways	0	0	0	-2	0
20	Liu et al.	How does the global plastic waste trade contribute to environmental benefits: Implication for reductions of greenhouse gas emissions?	0	0	0	1	-2
21	Tyler Eddy	Climate change drowned out by plastic	-2	-2	0	-3	0
22	Vea et al.	Inclusion of multiple climate tipping as a new impact category in life cycle assessment of polyhydroxyalkanoate (PHA)-based plastics	0	0	0	1	0
23	Sandberg et al.	Accounting for carbon flows into and from bio plastic in a national climate inventory	0	0	0	1	0
24	Barbara Demeneix	How fossil fuel-derived pesticides and plastics harm health, biodiversity, and the climate	-3	-3	0	-3	0
25	Tickner et al.	Transitioning the Chemical Industry: The Case for Addressing the Climate, Toxics, and Plastics Crises	-2	-2	1	-3	-2
26	Karin Kvale	Implications of plastic pollution on global marine carbon cycling and climate	-2	-2	0	-2	0
27	Jankowska et al.	Transforming the Plastic Production System Presents Opportunities to Tackle the Climate Crisis	-2	0	0	-2	0
28	Vishnuradhan et al.	Can plastics affect near surface layer ocean processes and climate?	-2	-2	0	-3	0
29	Oktavilia et al.	Plastic Industry and World Environmental Problems	-2	0	0	-2	0
30	An et al.	Estimated material metabolism and life cycle GHG emission of major plastics in China - A commercial sector scale perspective	-2	-2	0	-3	0
**Total**			**-36**	**-27**	**2**	**-35**	**-10**
**Goal 14. Conserve and sustainably use the oceans, seas and marine resources for sustainable development**	**Keywords: Marine, Pollution, Coast, Fishing**						
1	Xanthos et al.	International policies to reduce plastic marine pollution from single-use plastics (plastic bags and microbeads): A review	-2	-2	-3	-3	0
2	Schnurr et al.	Reducing marine pollution from single-use plastics (SUPs): A review	-2	-2	-3	-3	0
3	Villarrubia-Gómez et al.	Marine plastic pollution as a planetary boundary threat – The drifting piece in the sustainability puzzle	-2	-2	-2	-3	0
4	Vince et al.	Plastic pollution challenges in marine and coastal environments: from local to global governance	0	0	-2	-3	0
5	Wilcox et al.	Using expert elicitation to estimate the impacts of plastic pollution on marine wildlife	-2	-2	-3	-3	0
6	Thiel et al.	Impacts of marine plastic pollution from continental coasts to subtropical gyres-fish, seabirds, and other vertebrates in the SE Pacific	0	0	-2	-3	0
7	Schmaltz et al.	Plastic pollution solutions: emerging technologies to prevent and collect marine plastic pollution	0	0	-2	-3	0
8	Andrew Turner	Black plastics: Linear and circular economies, hazardous additives and marine pollution	0	-2	-2	-3	0
9	Adam et al.	Policies to reduce single-use plastic marine pollution in West Africa	-2	-2	1	-3	-2
10	Lestari et al.	The impact of improper solid waste management to plastic pollution in Indonesian coast and marine environment	-2	-2	-2	-3	-2
11	Compa et al.	Risk assessment of plastic pollution on marine diversity in the Mediterranean Sea	0	0	0	-3	-2
12	Oliveira et al.	Marine Environmental Plastic Pollution: Mitigation by Microorganism Degradation and Recycling Valorization	1	0	0	-2	-2
13	Borrelle et al.	Why we need an international agreement on marine plastic pollution	0	0	0	-3	0
14	Clayton et al.	Policy responses to reduce single-use plastic marine pollution in the Caribbean	0	-2	-2	-3	-2
15	Akinwumi et al.	Marine plastic pollution and affordable housing challenge: Shredded waste plastic stabilized soil for producing compressed earth bricks	0	0	0	1	0
16	Manfra et al.	Biodegradable polymers: A real opportunity to solve marine plastic pollution?	-2	0	0	-2	0
17	Ghayebzadeh et al.	Estimation of plastic waste inputs from land into the Caspian Sea: A significant unseen marine pollution	0	0	0	-3	0
18	Marks et al.	The geopolitical economy of Thailand's marine plastic pollution crisis	-2	-2	-2	-3	0
19	Chowdhury et al.	Estimating marine plastic pollution from COVID-19 face masks in coastal regions	0	0	2	-3	0
20	Walther et al.	Plastic pollution of four understudied marine ecosystems: a review of mangroves, seagrass meadows, the Arctic Ocean and the deep seafloor	0	0	-2	-3	0
21	Green et al.	Impacts of discarded plastic bags on marine assemblages and ecosystem functioning	0	0	0	-2	0
22	Willis et al.	Cleaner seas - reducing marine pollution	0	-2	-2	-3	-2
23	Kyriakopoulos et al.	Investigating the Human Impacts and the Environmental consequences of microplastics disposal	-2	-2	0	-3	0
24	Wang et al.	Seawater-Degradable Polymers—Fighting the Marine Plastic Pollution	0	0	0	1	0
25	Issifu et al.	A review of the production, recycling and management of marine plastic pollution	-2	-2	-2	-3	-2
26	Kwon et al.	Global styrene oligomers monitoring as new chemical contamination from polystyrene plastic marine pollution	-3	0	0	-3	0
27	Peter Dauvergne	The power of environmental norms: marine plastic pollution and the politics of microbeads	-2	-3	-2	-3	0
28	Peter Dauvergne	Why is the global governance of plastic failing the oceans?	-3	-2	-2	-3	-2
29	Abbott et al.	Reducing marine plastic pollution: Policy insights from economics	-2	-2	-2	-3	-2
30	Harris et al.	Using citizen science to evaluate extended producer responsibility policy to reduce marine plastic debris shows no reduction in pollution levels	0	0	0	-2	-2

**Total**			-29	-31	-34	-78	-20
**Goal 15. Protect, restore and promote sustainable use of terrestrial ecosystems, sustainably manage forests, combat desertification, and halt and reverse land degradation and halt biodiversity loss**	**Keywords: plastics and ecosystems and sustainable**						
1	Dris et al.	Beyond the ocean: Contamination of freshwater ecosystems with (micro-)plastic particles	-2	-2	0	-3	-2
2	Chae et al.	Current research trends on plastic pollution and ecological impacts on the soil ecosystem: A review	-2	-2	0	-3	-3
3	Kumar et al.	Impacts of plastic pollution on ecosystem services, sustainable development goals, and need to focus on circular economy and policy interventions	-2	-2	-2	-2	-2
4	Strungaru et al.	Micro- (nano) plastics in freshwater ecosystems: Abundance, toxicological impact and quantification methodology	-2	-2	0	-3	-2
5	Qadeer et al.	Agricultural plastic mulching as a potential key source of microplastic pollution in the terrestrial ecosystem and consequences	-2	-2	1	-2	-2
6	Bandopadhyay et al.	Biodegradable plastic mulch films: Impacts on soil microbial communities and ecosystem functions	0	0	0	1	0
7	Koskei et al.	Effects of increased plastic film residues on soil properties and crop productivity in agro-ecosystem	0	0	0	-2	0
8	Yu et al.	Micro plastics in soil ecosystem – A review of sources, fate, and ecological impact	-2	-2	0	-3	-2
9	Hurley et al.	Plastic waste in the terrestrial environment	-2	-2	-2	-3	-2
10	Astner et al.	Mechanical formation of micro- and nano-plastic materials for environmental studies in agricultural ecosystems	0	0	0	-2	0
11	Azevedo-Santos et al.	Plastic pollution: A focus on freshwater biodiversity	-2	-2	0	2	0
12	Schell et al.	Occurrence, Fate and Fluxes of Plastics and Microplastics in Terrestrial and Freshwater Ecosystems	-3	-2	-2	-3	-2
13	Zhao et al.	Fate of plastic film residues in agro-ecosystem and its effects on aggregate-associated soil carbon and nitrogen stocks	0	0	0	-3	0
14	Blettler et al.	Plastic pollution in freshwater ecosystems: macro-, meso-, and microplastic debris in a floodplain lake	0	0	0	-2	0
15	Malizia et al.	Terrestrial ecologists should stop ignoring plastic pollution in the Anthropocene time	-2	0	0	-3	0
16	Fan et al.	A review on the occurrence and influence of biodegradable microplastics in soil ecosystems: Are biodegradable plastics substitute or threat?	-2	-2	0	-2	0
17	Susanti et al.	Microplastics and the Impact of Plastic on Wildlife: A Literature Review	-2	-2	0	-3	0
18	Wang et al.	Uptake, translocation, and biological impacts of micro(nano)plastics in terrestrial plants: Progress and prospects	-2	-2	0	-2	-2
19	Zhao et al.	Microscopic anthropogenic litter in terrestrial birds from Shanghai, China: Not only plastics but also natural fibers	-2	-2	0	-2	0
20	Maclean et al.	The terrestrial plastisphere: Diversity and polymer-colonizing potential of plastic-associated microbial communities in soil	0	0	0	0	0
21	Mo et al.	Environmental fate and impacts of biodegradable plastics in agricultural soil ecosystems	-2	-2	-2	-3	-2
22	Amaneesh et al.	Gross Negligence: Impacts of Microplastics and Plastic Leachates on Phytoplankton Community and Ecosystem Dynamics	-2	-2	0	-3	-2
23	Guimarães et al.	Toxic effects of naturally-aged microplastics on zebrafish juveniles: A more realistic approach to plastic pollution in freshwater ecosystems	0	0	0	-2	0
24	Kasavan et al.	Plastic pollution in water ecosystems: A bibliometric analysis from 2000 to 2020	-2	-2	0	-3	0
25	Tudor et al.	The issue of plastic and microplastic pollution in soil	0	-2	0	-2	0
26	Ullah et al.	Micro(nano)plastic pollution in terrestrial ecosystem: emphasis on impacts of polystyrene on soil biota, plants, animals, and humans	0	0	0	-3	0
27	Zhang et al.	Microplastics pollution from different plastic mulching years accentuate soil microbial nutrient limitations	0	0	0	-2	-2
28	Joos et al.	Soil under stress: The importance of soil life and how it is influenced by (micro)plastic pollution	0	0	0	-2	0
29	Andrea et al.	Unraveling the role of plastic waste pollution in the Amvrakikos Wetlands National Park, Greece: The stakeholders' views	-2	-2	-2	-3	-2
30	Chen et al.	Feasibility of using plastic wastes as constructed wetland substrates and potential for pharmaceuticals and personal care products removal	0	-2	0	1	0
**Total**			-37	-38	-9	-62	-27
**Goal 16. Promote peaceful and inclusive societies for sustainable development, provide access to justice for all and build effective, accountable and inclusive institutions at all levels**	**Keywords: Justice, Peace, Regulation, Legislation, Governance, Policy**						
1	Katie Conolon	Adaptive injustice: Responsibility to act in the plastics economy	-3	-3	-2	-3	-2
2	Kim et al.	Designing for Green and Grey: Insights from Single-Use Plastic Water Bottles	-2	-3	-2	-2	0
3	Bahl et al.	Don't Transfer Waste, Transform Waste: A Sustainable Approach towards Zero Waste Events Initiative during Hockey World Cup in Bhubaneshwar, India	-2	0	1	2	2
4	Garcia et al.	Marine Plastic Pollution in Asia: All Hands on Deck!	-2	0	-2	-3	-2
5	Liboiron et al.	Abundance and types of plastic pollution in surface waters in the Eastern Arctic (Inuit Nunangat) and the case for reconciliation science	-3	0	-3	-3	-3
6	Paul Jobin	Our 'good neighbor' Formosa Plastics: petrochemical damage(s) and the meanings of money	-3	-3	-2	-3	0
7	Hsu et al.	Closing the loop on plastics in Europe - The role of data, information and knowledge	-2	-2	-2	-3	-2
8	Poto et al.	Suggestions for a systematic regulatory approach to ocean plastics	-2	0	0	-3	0
9	Owens et al.	Mopping Up or Turning Off the Tap? Environmental Injustice and the Ethics of Plastic Pollution	-2	-2	-3	-3	-3
10	Wang et al.	Circular Economy and the Changing Geography of International Trade in Plastic Waste	-3	-3	-3	-3	-3
11	Margaret Handley	Confronting the plasticine: promise in a world wrapped in plastic	-3	0	0	-3	0

12	Shirley et al.	Unwrapping Victoria's general environmental duty to plastics communities: Synthetic statutes	-2	-2	0	-3	0
13	Alice Mah	Future-proofing capitalism: The paradox of the circular economy for plastics	-2	-2	0	-3	-2
14	Ortiz et al.	A Regional Response to a Global Problem: Single Use Plastics Regulation in the Countries of the Pacific Alliance	-2	-2	0	-3	0
15	Carlini et al.	Advancing the international regulation of plastic pollution beyond the united nations environment assembly resolution on marine litter and microplastics	-2	-2	0	-3	-2
16	Steensgaard et al.	From macro- to microplastics - Analysis of EU regulation along the life cycle of plastic bags	-3	-3	-2	-3	-2
17	Tan et al.	Moving policy and regulation forward for single-use plastic alternatives	-2	-2	0	-2	0
18	Tham Hoang	Plastic pollution: Where are we regarding research and risk assessment in support of management and regulation?	-2	0	0	-3	0
19	Saputra et al.	Combining the concept of green accounting with the regulation of prohibition of disposable plastic use	-2	-2	-3	-3	-2
20	Tessnow-von Wysocki et al	Plastics at sea: Treaty design for a global solution to marine plastic pollution	-2	-2	0	-3	0
**Total**			-46	-33	-23	-53	-21
**Goal 17. Strengthen the means of implementation and revitalize the global partnership for sustainable development**	**Keywords: Partnership, Cooperation, Global, Trade, Market**						
1	Bank et al.	Global plastic pollution observation system to aid policy	-3	-3	0	-3	0
2	Marcus Haward	Plastic pollution of the world's seas and oceans as a contemporary challenge in ocean governance	-3	-3	0	-3	-2
3	Freeman et al.	Between source and sea: The role of wastewater treatment in reducing marine microplastics	-3	-3	0	-3	0
4	Duong et al.	A Model Template Green Environment Initiative for Recycling plastic bottles with progressive entrepreneurship partnership	-2	0	0	2	0
5	Kandziora et al.	The important role of marine debris networks to prevent and reduce ocean plastic pollution	-3	-3	0	-3	0
6	Li et al.	The collapse of global plastic waste trade: Structural change, cascading failure process and potential solutions	-3	-3	-2	-3	-3
7	Galaiduk et al.	Transnational Plastics: An Australian Case for Global Action	-3	-3	0	-3	-3
8	Barrowclough et al.	Transforming the Global Plastics Economy: The Role of Economic Policies in the Global Governance of Plastic Pollution	-3	-3	0	-3	-3
9	Kuan et al.	Towards regional cooperation on sustainable plastic recycling: comparative analysis of plastic waste recycling policies and legislations in Japan and Malaysia	-3	0	0	-3	-2
10	Finska et al.	Troubled waters - Where is the bridge? Confronting marine plastic pollution from international watercourses	-3	0	0	-3	-3
11	Sun et al.	Regional cooperation in marine plastic waste cleanup in the south china sea region	-3	0	0	-3	0
12	Nancy Lamontagne	Academic-Industry Partnerships: Preparing Tomorrow's Plastics Professionals	0	2	0	0	0
13	Stuchtey et al.	Project stop: City partnerships to prevent ocean plastics in Indonesia	-3	0	0	-2	1
14	Raubenheimer et al.	Rethinking global governance of plastics – The role of industry	-2	0	0	-3	0
15	Fadeeva et al.	'Unlocking circular economy for prevention of marine plastic pollution: An exploration of G20 policy and initiatives'	-3	-2	0	-3	0
16	Nielsen et al.	Politics and the plastic crisis: A review throughout the plastic life cycle	-3	-3	-2	-3	0
17	Shruti et al.	Strengthening citizen science partnerships with frontline sanitation personnel to study and tackle plastic pollution	-2	-2	0	-3	-2
18	Micah Landon-Lane	Corporate social responsibility in marine plastic debris governance	-2	0	-2	-3	0
19	Shah et al.	Plastics waste metabolism in a Petro-Island state: Towards solving a "wicked problem" in trinidad and tobago	-2	0	0	-3	0
20	Bagai et al.	Beating plastic pollution: UNEP's priorities and partnership in India	-2	0	0	-2	0
**Total**			-51	-26	-6	-50	-17

Relationship between the literature and ecodesign strategies (3 - Strong, 2 - Moderate, 1 - Unclear , 0 - No)

S.Nr.	Author	Title of the study	Low-impact materials	Reduction of materials usage	Optimisation of production techniques	Optimisation of distribution system	Reduction of impacts during use	Optimisation/Extension of lifetime	Optimisation of end-of-life system	New concept development
			a) Cleaner materials<br>b) Renewable materials<br>c) Lower energy content materials<br>d) Recycled/Recyclable materials	a) Reduction in weight<br>b) Reduction in (transport) volume	a) Alternative production techniques<br>b) Fewer production steps<br>c) Lower/cleaner energy consumption<br>d) Fewer/cleaener production and consumables	a) Less/Cleaner/Reusable Packaging<br>c) Energy-efficient logistics	a) Lower energy consumption<br>b) Cleaner energy source<br>c) Fewer consumables needed<br>d) Cleaner consumable<br>e) No waste of energy/consumables	a) Reliability and durability<br>b) Modular product structure<br>c) Classic Design<br>d) Human centered design	a) Reuse of product<br>b) Remanufacturing/Refurbishing<br>c) Recycling of materials	a) Dematerialization<br>b) Shared use of product<br>c) Integration of functions<br>d) Functional optimization<br>e) Efficient function fulfillment<br>f) Policy framework changes
**Goal 1. End poverty in all its forms everywhere**	**Keywords: Poverty, People, Population**									
1	Mpanangombe et al.	Poverty, politics and plastic - Organic waste sorting in Blantyre's public markets	2	0	0	0	3	0	3	3
2	David Katz	Plastic Bank: launching Social Plastic® revolution	3	0	0	2	0	0	3	2
3	Mukama et al.	Practices, Concerns, and Willingness to Participate in Solid Waste Management in Two Urban Slums in Central Uganda	2	0	0	0	0	0	3	2
4	Gall et al.	Building a circular plastics eco0my with informal waste pickers: Recyclate quality, business model, and societal impacts	3	1	0	2	1	0	3	2
5	El Mekaoui et al.	Plastic bags ban and social marginalization: Evidence from Morocco	3	0	0	1	1	2	2	2
6	Jenks et al.	The poverty of plastics bans: Environmentalism's win is a loss for disabled people	3	0	0	0	2	2	2	3
7	Stoler et al.	Piped water flows but sachet consumption grows: The paradoxical drinking water landscape of an urban slum in Ashaiman, Ghana	1	0	0	2	0	0	3	2
8	Velis et al.	Enabling the informal recycling sector to prevent plastic pollution and deliver an inclusive circular eco0my	0	0	0	0	0	0	3	3
9	Obiadi Bons N. O0chie A.O	The negative impacts of poverty in urban and rural architecture in Nigeria	3	0	0	0	2	2	3	3
10	Klocker et al.	From troublesome materials to fluid tech0logies: Making and playing with plastic-bag footballs	3	3	3	2	0	2	3	3
**Total**			**23**	**4**	**3**	**9**	**9**	**8**	**28**	**25**
**Goal 2. End hunger, achieve food security and improved nutrition and promote sustainable agriculture**	**Keywords: Agriculture, Hunger, Food, Food security**									
1	He et al.	Plastic mulch: Tradeoffs between productivity and greenhouse gas emissions	3	0	0	1	2	2	3	3
2	Saeed et al.	Competency of groundwater recharge of irrigated cotton field subjacent to sowing methods, plastic mulch, water productivity, and yield under climate change	0	0	0	0	2	0	0	1
3	Wang et al.	Plastic mulching reduces nitrogen footprint of food crops in China: A meta-analysis	0	0	0	0	2	0	2	0
4	Castillo-Diaz et al.	The management of agriculture plastic waste in the framework of circular eco0my. Case of the almeria greenhouse (Spain)	3	0	2	2	3	2	3	3
5	Batista et al.	Dealing with Plastic Waste from Agriculture Activity	3	0	0	0	1	0	3	3
6	Pazienza et al.	For a new plastics eco0my in agriculture: Policy reflections on the EU strategy from a local perspective	1	0	0	0	2	0	2	3
7	Chen et al.	Response of carbon footprint to plastic film mulch application in spring maize production and mitigation strategy	1	0	0	0	3	0	2	3
8	Xie et al.	Yield, eco0mic benefit, soil water balance, and water use efficiency of intercropped maize/potato in responses to mulching practices on the semiarid loess plateau	0	0	0	0	2	0	0	2
9	Zhang et al.	Plastic pollution in croplands threatens long-term food security	3	0	0	0	3	0	3	2
10	Lwanga et al.	Field evidence for transfer of plastic debris along a terrestrial food chain	3	0	0	0	3	0	3	3
11	Ruimin et al.	Behavior of microplastics and plastic film residues in the soil environment: A critical review	3	0	0	0	2	0	3	3
12	Chen et al.	Effects of plastic film combined with straw mulch on grain yield and water use efficiency of winter wheat in Loess Plateau	0	0	0	0	0	0	0	0
13	Hu et al.	Comparative analysis of carbon footprint between conventional smallholder operation and in0vative largescale farming of urban agriculture in Beijing, China	2	0	0	0	3	3	3	3
14	Gao et al.	Effects of plastic mulching and plastic residue on agricultural production: A meta-analysis	2	0	0	0	3	2	3	3
15	Brodhagen et al.	Policy considerations for limiting unintended residual plastic in agricultural soils	2	0	0	0	2	0	2	3
16	Stubenrauch et al.	Plastic Pollution in Soils: Governance Approaches to Foster Soil Health and Closed Nutrient Cycles	2	0	0	0	2	2	3	3
17	Edet et al.	Impact of "sachet water" microplastic on agricultural soil physicochemistry, antibiotics resistance, bacteria diversity and function	3	0	0	0	2	2	3	3
18	Sun et al	Contamination of phthalate esters, orga0chlorine pesticides and polybrominated diphenyl ethers in agricultural soils from the Yangtze River Delta of China	3	0	0	0	2	0	0	0
19	Xucheng et al.	Did plastic mulching constantly increase crop yield but decrease soil water in a semiarid rain-fed area?	2	0	0	0	0	0	2	3
20	Cozzoli0 et al.	Appraisal of biodegradable mulching films and vegetal-derived biostimulant application as eco-sustainable practices for enhancing lettuce crop performance and nutritive value	3	0	0	0	2	0	3	3
**Total**			**39**	**0**	**2**	**3**	**41**	**13**	**43**	**47**
**Goal 3. Ensure healthy lives and promote well-being for all at all ages**	**Keywords: Health, Death, Illness, Disease, Mortality**									
1	Metcalf et al.	Quantifying the importance of plastic pollution for the dissemination of human pathogens: The challenges of choosing an appropriate 'control' material	3	0	0	0	3	0	3	3
2	Bouwmeester et al.	Potential Health Impact of Environmentally Released Micro- and Nanoplastics in the Human Food Production Chain: Experiences from Nanotoxicology	3	0	0	0	3	0	3	2
3	Pramod Kumar	Role of Plastics on Human Health	3	2	2	0	0	0	0	3
4	Li et al.	Environmental risks of disposable face masks during the pandemic of COVID-19: Challenges and management	3	0	0	0	0	0	3	3
5	Yuan et al.	Human health concerns regarding microplastics in the aquatic environment - From marine to food systems	3	2	0	0	2	0	3	2
6	Landrigan et al.	Human health and ocean pollution	3	2	2	2	0	0	3	3
7	Groh et al.	Overview of known plastic packaging-associated chemicals and their hazards	0	0	2	0	0	0	0	2
8	Yee et al.	Impact of microplastics and na0plastics on human health	2	0	0	0	2	0	2	1
9	Leslie et al.	Discovery and quantification of plastic particle pollution in human blood	3	0	0	0	0	0	3	2
10	Jiang et al.	Health impacts of environmental contamination of micro- And naoplastics: A review	3	0	0	0	0	0	3	2
11	Adebiyi-Abiola et al.	Cleaning up plastic pollution in Africa	2	3	2	0	0	0	3	3
12	Yang et al.	Waste management, informal recycling, environmental pollution and public health	2	0	0	0	0	2	3	3
13	Hahladakis et al.	An overview of chemical additives present in plastics: Migration, release, fate and environmental impact during their use, disposal and recycling	3	3	2	0	0	2	3	3
14	Gasperi et al.	Microplastics in air: Are we breathing it in?	3	3	0	0	0	0	3	2
15	Barboza et al.	Marine microplastic debris: An emerging issue for food security, food safety and human health	3	0	0	0	2	0	3	2
16	Mason et al.	Synthetic Polymer Contamination in Bottled Water	3	3	3	0	0	2	0	3
17	Wright et al.	Plastic and Human Health: A Micro Issue?	3	3	0	0	2	0	3	3
18	Fred-Ahmadu et al.	Interaction of chemical contaminants with microplastics: Principles and perspectives	3	3	0	0	2	0	3	3
19	Velis et al.	Mismanagement of Plastic Waste through Open Burning with Emphasis on the Global South: A Systematic Review of Risks to Occupational and Public Health	3	0	0	0	0	2	3	3
20	Tang et al.	Polybrominated diphenyl ethers (PBDEs) and heavy metals in road dusts from a plastic waste recycling area in 0rth China: implications for human health	3	0	2	0	2	2	3	3
**Total**			**54**	**24**	**15**	**2**	**18**	**10**	**50**	**51**

**Goal 4. Ensure inclusive and equitable quality education and promote lifelong learning opportunities for all**	**Keywords: Education, Skills, Qualification**									
1	Chmielewski et al.	Microplastic in the environment. The role of education in raising social awareness of the handling of plastic waste	2	0	3	2	2	2	3	3
2	Mironenko et al.	Education Against Plastic Pollution: Current Approaches and Best Practices	2	1	0	0	2	2	3	3
3	Bennett et al.	Informing the public and educating students on plastic recycling	0	1	3	0	2	2	3	3
4	Dalu et al.	Is awareness on plastic pollution being raised in schools? Understanding perceptions of primary and secondary school educators	0	2	0	0	0	2	2	2
5	Kowasch et al.	Circular eco0my, cradle to cradle and zero waste frameworks in teacher education for sustainability	0	2	0	0	0	2	3	3
6	Jimenez et al.	'It's my responsibility': perspectives on environmental justice and education for sustainability among international school students in Singapore	2	2	0	0	0	2	0	2
7	Situmorang et al.	The difference of k0wledge and behavior of college students on plastic waste problems	2	0	0	0	0	0	3	2
8	Hammami et al.	Survey on awareness and attitudes of secondary school students regarding plastic pollution: implications for environmental education and public health in Sharjah city, UAE	2	2	0	0	0	0	3	3
9	Parejo et al.	Plastics as an educational resource for sustainable development: A case study in Ghana	3	3	0	0	0	2	3	3
10	Phan Hoang et al.	Measuring the effect of environmental education for sustainable development at elementary schools: A case study in Da Nang city, Vietnam	3	2	0	0	0	2	3	3
11	Khanam et al.	Knowledge, attitude and practice on uses of plastic products, their disposal and environmental pollution: A study among school-going adolescents	2	0	0	0	0	2	3	2
12	Barra et al.	Sustainable chemistry challenges from a developing country perspective: Education, plastic pollution, and beyond	3	0	2	0	0	2	3	3
13	So et al.	Environmental education in primary schools: A case study with plastic resources and recycling	2	0	0	0	0	2	3	3
14	Cheung et al.	A train-the-trainer design for green ambassadors in an environmental education programme on plastic waste recycling	0	2	0	0	0	0	3	2
15	Chow et al.	Plastic waste problem and education for plastic waste management	2	0	0	0	0	0	2	2
16	Soares et al.	On the path to minimize plastic pollution: The perceived importance of education and k0wledge dissemination strategies	0	0	0	0	0	2	2	3
17	Mandrikas et al.	In-service Teachers' Needs and Mentor's Practices in Applying a Teaching–Learning Sequence on Na0tech0logy and Plastics in Primary Education	0	0	0	0	0	0	2	0
18	Lopez-Fernandez et al.	How Can Socio-scientific Issues Help Develop Critical Thinking in Chemistry Education? A Reflection on the Problem of Plastics	2	0	0	0	0	0	2	2
19	du Bois et al.	Design Against the Plastic Soup - The Effect of Small Product Designs in Sustainable Design Education	2	2	3	2	0	2	3	3
20	Schiffer et al.	Microplastics Outreach Program: A Systems-Thinking Approach to Teach High School Students about the Chemistry and Impacts of Plastics	2	0	2	0	0	2	3	2
**Total**			**31**	**19**	**13**	**4**	**6**	**28**	**52**	**49**
**Goal 5. Achieve gender equality and empower all women and girls**	**Keywords: Gender, Women, Equality, Policy**									
1	De La Pena et al.	Empowering Women Thru Plastics Recycling: A Livelihood Project for Women in Marginalized Communities	0	0	0	2	0	0	2	2
2	Braun et al.	Plastic Bags, Pollution, and Identity: Women and the Gendering of Globalization and Environmental Responsibility in Mali	2	0	0	0	0	0	3	3
3	Chatterjee et al.	Green chemistry – Remedy to societal hygiene: A graphical review	2	0	0	0	0	0	3	2
4	Anne Marie Hanson	Women's environmental health activism around waste and plastic pollution in the coastal wetlands of Yucatán	3	2	0	0	0	0	3	2
5	B.Lynne Milgram	From Trash to Totes: Recycled Production and Cooperative Economy Practice in the Philippines	2	0	0	0	0	3	3	3
6	Lange et al.	Understanding the Socio-Demographic Profile of Waste Re-Users in a Suburban Setting in South Africa	0	2	0	0	0	3	3	2
7	Chukwuone et al.	Determinants of household's waste disposal practices and willingness to participate in reducing the flow of plastics into the ocean: Evidence from coastal city of Lagos Nigeria	3	2	0	0	0	2	3	3
8	Robert Grace	Overcoming Gender Bias in Design	0	0	0	0	2	2	0	2
9	Muralidharan et al.	The Role of Guilt in Influencing Sustainable Pro-Environmental Behaviors among Shoppers	2	2	0	2	0	3	0	3
10	Mikołajewska et al.	Bisphenol A – Application, sources of exposure and potential risks in infants, children and pregnant women	3	3	0	0	0	0	3	3
11	Gao et al.	Feminine Hygiene Products - A Neglected Source of Phthalate Exposure in Women	3	3	0	0	0	0	2	3
12	Valvi et al.	Variability and predictors of urinary phthalate metabolites in Spanish pregnant women	3	3	0	0	0	0	0	2
13	Anne Marie Hanson	Women's ecological oral histories of recycling and development in coastal Yucatán	3	2	0	0	0	0	3	2
14	0rfaryanti et al.	Environmental-friendly food products' packaging: Women's purchasing preferences	3	2	0	2	0	2	3	2
15	Bula Sirika Wayessa	Prepared in pots, served in plastics: Rural Ethiopian women's responses to the global eco0my	0	0	0	0	0	0	0	3
16	Muposhi et al.	Is the use of green shopping bags gendered? Evidence from a gender equality conscious emerging market	2	3	0	0	0	2	3	3
17	Alharbi et al.	Use of Plastics with Hot Food among Saudi Pregnant Women Is Associated with Increased Concentrations of A1C, Thyroid-Stimulating Hormone, and Homocysteine and Decreased Concentrations of Vitamins and Minerals	3	0	0	0	3	0	0	2
18	Chowdhury et al.	WASH and MHM experiences of disabled females living in Dhaka slums of Bangladesh	0	0	0	0	0	0	3	2
19	Anusha et al.	Ready-to-eat food consumption practices, food safety knowledge and relation to gender and education level of consumers in Kuala Lumpur, Malaysia	0	0	0	0	0	3	0	0
20	Trowbridge et al.	Fossil fuel is the common de0minator between climate change and petrochemical exposures, and effects on women and children's health	3	2	0	0	0	0	3	2
**Total**			**37**	**26**	**0**	**6**	**5**	**20**	**40**	**46**
**Goal 6. Ensure availability and sustainable management of water and sanitation for all**	**Keywords: Water, Sanitation, Drinking, Scarcity**									
1	Mihai et al.	Plastic Pollution, Waste Management Issues, and Circular Eco0my Opportunities in Rural Communities	3	3	2	2	2	2	3	3
2	Yeasmin et al.	Piloting a low-cost hardware intervention to reduce improper disposal of solid waste in communal toilets in low-income settlements in Dhaka, Bangladesh	0	0	0	0	0	2	3	3
3	Geetha T	Endocrine disruptors in boiled drinking water carried in plastic containers: a pilot study in Thrissur, Kerala, India	3	0	0	0	0	0	0	2
4	Liu et al.	Do estrogenic compounds in drinking water migrating from plastic pipe distribution system pose adverse effects to human? An analysis of scientific literature	3	0	0	0	0	0	0	2
5	Manjaya et al.	Informally vended sachet water: Handling practices and microbialwater quality	3	0	0	0	2	0	0	2
6	Barletta et al.	Distribution, sources and consequences of nutrients, persistent organic pollutants, metals and microplastics in South American estuaries	3	3	0	0	0	0	3	3
7	Haris et al.	Installing public handwashing facilities and integrating them with water fountains to reduce plastic pollution and prevent spread of infections	0	2	0	0	0	0	3	3
8	Dorji et al.	On-site domestic wastewater treatment system using shredded waste plastic bottles as biofilter media: Pilot-scale study on effluent standards in Bhutan	0	0	0	0	0	2	2	2
9	Szczotko et al.	Evaluation of susceptibility of polymer and rubber materials intended into contact with drinking water on biofilm formation	0	2	0	0	0	0	0	0
10	Alda-Vidal et al.	"Unflushables": Establishing a global agenda for action on everyday practices associated with sewer blockages, water quality, and plastic pollution	2	0	0	0	0	0	3	2
11	Egun et al.	Beat the plastic: an approach to polyethylene terephthalate (PET) bottle waste management in Nigeria	3	2	2	2	2	2	3	3

12	Winter et al.	Women’s sanitation practices in informal settlements: A multi-level analysis of factors influencing utilisation in Nairobi, Kenya	0	0	0	0	0	0	3	0
13	O Briain et al.	The role of wet wipes and sanitary towels as a source of white microplastic fibres in the marine environment	3	2	0	0	0	0	3	2
14	Borde et al.	Community challenges when using large plastic bottles for Solar Energy Disinfection of Water (SODIS)	0	0	3	0	0	0	0	0
15	Diehl et al.	Do-it-Yourself (DIY) Workspaces Run by Local Entrepreneurs that Transform Plastic Waste into Valuable Water and Sanitation Products	0	0	0	0	0	3	3	3
16	Necibi et al.	Contaminants of emerging concern in african wastewater effluents: Occurrence, impact and removal tech0logies	3	0	0	0	0	0	2	3
17	Taduswan et al.	Microplastic contamination in a conventional wastewater treatment plant in Thailand	3	0	0	0	0	0	3	3
18	Muanda et al.	Factors and impacts of informal settlements residents’ sanitation practices on access and sustainability of sanitation services in the policy context of free basic sanitation	0	0	0	0	0	0	2	2
19	Semey et al.	Characteristics of packaged water production facilities in greater Accra, Ghana: Implications for water safety and associated environmental impacts	0	0	0	0	0	0	2	2
20	Tang et al.	Different senescent HDPE pipe-risk: brief field investigation from source water to tap water in China (Changsha City)	0	2	0	0	0	0	2	0
**Total**			**29**	**16**	**7**	**4**	**6**	**11**	**40**	**40**
**Goal 7. Ensure access to affordable, reliable, sustainable and modern energy for all**	**Keywords: Energy, Renewable, Sustainable, Clean**									
1	Dong et al.	Design of special plastic bearings and their application in renewable energy conversion system	0	0	0	0	0	2	2	0
2	Stephen et al.	Completing the value chain for plastic recyclers in Nigeria: An integration of renewable solar and conventional gas energy sources for fuel production	0	0	3	0	0	0	3	2
3	Sagariga et al.	Plastic to Fuel Conversion System Using Renewable Energy Assisted Pyrolysis	0	0	3	0	0	0	3	2
4	Daniel Posen et al.	Greenhouse gas mitigation for U.S. plastics production: Energy first, feedstocks later	3	0	3	0	0	0	0	2
5	Gebre et al.	Recent Trends in the Pyrolysis of 0n-Degradable Waste Plastics	0	0	3	0	0	0	3	2
6	Gug et al.	Processing and properties of a solid energy fuel from municipal solid waste (MSW) and recycled plastics	0	0	3	0	0	0	3	2
7	Sharuddin et al.	Pyrolysis of plastic waste for liquid fuel production as prospective energy resource	0	0	3	0	0	0	3	2
8	Lombardi et al.	A review of tech0logies and performances of thermal treatment systems for energy recovery from waste	0	0	2	0	0	0	2	2
9	Wong et al.	Current state and future prospects of plastic waste as source of fuel: A review	0	0	2	0	0	0	2	2
10	Sharuddin et al.	A review on pyrolysis of plastic wastes	0	0	2	0	0	0	2	2
11	Lam et al.	Microwave vacuum pyrolysis of waste plastic and used cooking oil for simultaneous waste reduction and sustainable energy conversion: Recovery of cleaner liquid fuel and tech0-eco0mic analysis	0	0	2	0	0	0	2	2
12	Shen et al.	Waste-to-energy: Dehalogenation of plastic-containing wastes	0	0	2	0	0	0	2	2
13	Chandrasekeran et al.	Materials and Energy Recovery from E-Waste Plastics	0	0	2	0	0	0	2	2
14	Bukkarapu et al.	Management, conversion, and utilization of waste plastic as a source of sustainable energy to run automotive: a review	0	0	0	0	0	0	2	2
15	Devasahayam et al.	Review: Opportunities for simultaneous energy/materials conversion of carbon dioxide and plastics in metallurgical processes	0	0	0	0	0	0	2	2
16	Surenderan et al.	Characterization studies on waste plastics as a feedstock for energy recovery in Malaysia	0	0	2	0	0	0	2	0
17	Lim et al.	Optimal sorting and recycling of plastic waste as a renewable energy resource considering eco0mic feasibility and environmental pollution	0	0	2	0	0	2	2	2
18	Willenbacher et al.	Machine learning for optimization of energy and plastic consumption in the production of thermoplastic parts in SME	3	0	2	0	0	0	0	2
19	Olazabal et al.	From plastic waste to new materials for energy storage	3	0	2	0	0	0	3	2
20	Nugraho et al.	Plastic waste as an alternative energy	0	0	2	0	0	0	3	2
**Total**			**9**	**0**	**40**	**0**	**0**	**4**	**43**	**36**
**Goal 8. Promote sustained, inclusive and sustainable economic growth, full and productive employment and decent work for all**	**Keywords: Economy, Job, Growth, Employment**									
1	Umeda et al.	Potential impacts of the European Union’s circular economy policy on Japanese manufacturers	2	0	2	0	0	2	3	0
2	Andreoni et al.	Polyethylene recycling: Waste policy scenario analysis for the EU-27	0	0	0	0	0	2	0	2
3	Nara et al.	Expected impact of industry 4.0 technologies on sustainable development: A study in the context of Brazil's plastic industry	0	0	2	0	0	0	0	2
4	Vimal et al.	Analysis of barriers that impede the elimination of single-use plastic in developing economy context	0	2	2	0	0	3	2	3
5	Rossi et al.	Circular economy indicators for organizations considering sustainability and business models: Plastic, textile and electro-electronic cases	0	0	2	0	0	2	2	2
6	Wu et al.	Supporting a circular economy: Insights from Taiwan's plastic waste sector and lessons for developing countries	0	2	2	0	0	2	3	3
7	Carola Guyot Phung	Implications of the circular economy and digital transition on skills and green jobs in the plastics	0	2	3	0	0	2	3	3
8	Hofmann et al.	The value chain and activities of polyethylene terephthalate plastics in the South African waste economy	0	0	0	0	0	2	3	2
9	Bala et al.	Identifying the prospects of decent job creation along the value chain of plastic recycling	2	0	0	0	0	0	2	2
10	Bening et al.	The true cost of solving the plastic waste challenge in developing countries: The case of Ghana	0	0	0	0	0	2	2	2
11	Bai et al.	Ecologically unequal exchange of plastic waste? A longitudinal analysis of international trade in plastic waste	2	2	0	0	0	2	2	3
12	Torres et al.	The need for technical improvement in the plastics recycling industry in middle-income countries: The Peruvian case	0	0	0	0	0	0	2	0
13	Cordier et al.	Plastic pollution and economic growth: The influence of corruption and lack of education	0	0	0	0	0	0	2	0
14	Stuart J. Barnes	Understanding plastics pollution - The role of economic development and technological research	0	0	0	0	0	1	2	2
15	Wu et al.	Impact of strategic control and supply chain management on recycled plastic additive manufacturing	0	0	3	3	0	0	2	3
16	Silva et al.	Simulation of elements of industry 4.0 applied to the production processes of a company dedicated to the manufacture of plastic products	3	3	3	0	0	0	0	0
17	Babarinsa et al.	Potential Socio-economic impact of replacing traditional woven baskets with reusable plastic crates on livelihoods of basker makers in southeast Nigeria	3	0	0	0	0	0	0	3
18	Alvarado-Diaz et al	Design of a Plastic Shredding Machine to Obtain Small Plastic Waste	0	0	3	0	0	3	0	0
19	Arabi et al.	Impacts of marine plastic on ecosystem services and economy: State of South African research	0	0	0	0	0	0	0	2
20	Adedeji Adelodun	Plastic Recovery and Utilization: From Ocean Pollution to Green Economy	0	0	2	0	0	2	2	2
**Total**			**12**	**11**	**24**	**3**	**0**	**25**	**32**	**36**
**Goal 9. Build resilient infrastructure, promote inclusive and sustainable industrialization and foster innovation**	**Keywords: Innovation, Infrastructure, Research, Industry**									
1	Moshood et al.	Biodegradable plastic applications towards sustainability: A recent innovations in the green product	2	0	0	0	0	0	0	3
2	Raddadi et al.	Biodegradation of oil-based plastics in the environment: Existing knowledge and needs of research and innovation	2	0	0	0	0	2	0	0
3	Getor et al.	The role of technological innovation in plastic production within a circular economy framework	3	0	2	0	0	2	2	3
4	Junior et al.	Knowledge-based dynamic capabilities for sustainable innovation: The case of the green plastic project	3	0	3	0	0	0	0	3
5	Nel et al.	Collaboration and infrastructure is needed to develop an African perspective on micro(nano)plastic pollution	0	0	0	0	0	0	0	3
6	Friedrich et al.	Supporting the development process for building products by the use of research portfolio analysis: A case study for wood plastics composite materials	2	0	0	0	0	0	0	2

7	Sommer et al.	Recycling and recovery infrastructures for glass and carbon fiber reinforced plastic waste from wind energy industry: A European case study	0	2	0	0	0	3	3	3
8	Gong et al.	Investigation into circular economy of plastics: The case of the UK fast moving consumer goods industry	3	3	2	2	0	3	2	3
9	Dijkstra et al.	In the business of dirty oceans: Overview of startups and entrepreneurs managing marine plastic	2	0	0	0	0	2	3	2
10	Siltaloppi et al.	Toward a sustainable plastics value chain: Core conundrums and emerging solution mechanisms for a systemic transition	2	0	2	0	2	3	3	2
11	Cheng et al.	Investigating the Economic Feasibility of Community-Scale Plastic Recycling Facilities	0	0	2	0	0	2	2	3
12	Bing et al.	Global reverse supply chain redesign for household plastic waste under the emission trading scheme	0	0	0	3	0	0	3	3
13	Muposhi et al.	Considerations, benefits and unintended consequences of banning plastic shopping bags for environmental sustainability: A systematic literature review	0	0	0	0	2	0	3	2
14	Knoblauch et al.	Government policies combatting plastic pollution	0	0	0	2	2	2	3	2
15	Rivera-Huerta et al.	Innovation in the informal sector: The case of plastic recycling firms in Mexico	0	0	2	0	0	2	0	0
16	Oyake-Ombis et al.	Managing plastic waste in East Africa: Niche innovations in plastic production and solid waste	0	0	0	2	2	2	3	2
17	Cordier et al.	How much innovation is needed to protect the ocean from plastic contamination?	0	3	2	3	3	3	3	2
18	Huang et al.	Evolution of network relations, enterprise learning, and cluster innovation networks: the case of the Yuyao plastics industry cluster	2	0	2	0	0	0	2	3
19	Oyinlola et al.	Digital innovations for transitioning to circular plastic value chains in Africa	0	0	0	0	2	0	2	3
20	Ishartomo et al.	Learning from plastic waste village in Boyolali Indonesia: SME-based plastic recycling industries	0	0	0	0	0	0	2	0
21	Conchubhair et al.	Joint effort among research infrastructures to quantify the impact of plastic debris in the ocean	0	0	0	0	0	0	0	2
22	Ma et al.	Economic evaluation of infrastructures for thermochemical upcycling of post-consumer plastic waste	0	0	2	2	0	0	2	0
23	Hinton et al.	Innovations Toward the Valorization of Plastics Waste	0	0	3	0	0	0	3	0
24	Nancy Lamontagne	Recycling the Unrecyclable Industry innovations aim to keep traditionally difficult-to-recycle plastics out of the landfill	0	0	3	0	0	0	3	3
25	Vollmer et al.	Beyond Mechanical Recycling: Giving New Life to Plastic Waste	0	0	3	0	0	0	3	0
26	Muposhi et al.	Embedding Ecopreneurial Behaviour: Proposed Social Marketing Interventions From Value-In-Behaviour Perceptions of Plastic Waste Ecopreneurs	0	0	0	0	0	0	3	3
27	da Silva et al.	Evolution toward environment sustainable behavior: search for survival in the plastic industry in Brazil	0	0	2	0	0	2	0	0
28	Ana Espada	Design as a source of innovation to establish circular models: An opportunity to prevent the single use of plastic	0	2	2	0	2	3	2	2
29	Chow et al.	Research and development of a new waste collection bin to facilitate education in plastic recycling	0	0	0	0	2	3	2	3
30	Reddy et al.	Review of the Utilization of Plastic Wastes as a Resource Material in Civil Engineering Infrastructure Applications	2	0	2	0	0	0	0	2
**Total**			**23**	**10**	**34**	**14**	**17**	**34**	**54**	**59**
**Goal 10. Reduce inequality within and among countries**	**Keywords: Income, Injustice, Inequality, Social**									
1	Velis et al.	Plastic pollution global treaty to cover waste pickers and open burning?	0	0	0	0	0	0	3	3
2	Benedetta Cotta	What goes around, comes around? Access and allocation problems in Global North–South waste trade	0	0	0	3	0	2	2	3
3	Samantha Chisholm Hatfield	Plastic suffocation: Climate change threatens indigenous populations and traditional ecological knowledge	3	0	0	0	0	3	0	0
4	Horvath et al.	Designing Business Solutions for plastic waste management to enhance circular transitions in Kenya	0	2	2	3	2	2	2	3
5	Horne et al.	High-rise plastic: Socio-material entanglements in apartments	0	0	0	0	0	3	0	0
6	Jamie Furniss	Alternative framings of transnational waste flows reflections based on the Egypt China PET plastic	0	0	0	0	0	0	0	0
7	Borman et al.	Impact of plastic packaging design on the sustainability of plastic recyclers	2	0	2	0	0	2	2	2
8	Saskia Abrahms-Kavunenko	Toward an anthropology of plastics	0	0	0	0	0	0	0	0
9	Nicolas Schlitz	Environmental change and the informal plastic recycling networks of Kolkata	0	0	0	0	0	3	3	2
10	Hossain et al.	Socioeconomic Relation with Plastic Consumption on 61 Countries Classified by Continent, Income Status and Coastal Regions	0	0	0	0	0	0	0	0
11	Kumar et al.	Estimation of the generation rate of different types of plastic wastes and possible revenue recovery from informal recycling	0	0	0	0	0	0	3	0
12	Gareiou et al.	Awareness of Citizens for the Single-Use Plastics: Comparison between a High-Income and an Upper-Middle-Income Economy of the Easter Mediterranean Region, Greece and Lebanon	2	0	0	0	0	2	2	0
13	Knoblauch et al.	Developing countries in the lead-what drives the diffusion of plastic bag policies?	2	0	0	0	0	2	2	2
14	Muisa Zikali et al.	Household solid waste handling practices and recycling value for integrated solid waste management in a developing city in Zimbabwe	2	0	0	0	0	2	2	0
15	Karasik et al.	Inequitable distribution of plastic benefits and burdens on economies and public health	0	0	0	0	0	0	0	2
16	Navarre et al.	Recycled plastic packaging from the Dutch food sector pollutes Asian oceans	0	0	0	0	0	3	3	2
17	Abalansa et al.	The marine plastic litter issue: A social-economic analysis	2	0	0	0	0	2	2	2
18	Cook et al.	Scaling up resource recovery of plastics in the emergent circular economy to prevent plastic pollution: Assessment of risks to health and safety in the Global South	2	0	0	0	0	2	2	2
19	Chowdhury et al.	The ecological impact of plastic pollution in a changing climate	0	0	0	0	0	0	0	3
20	Stuart J. Barnes	Out of sight, out of mind: Plastic waste exports, psychological distance and consumer plastic purchasing	0	0	0	0	0	0	0	3
**Total**			15	2	4	6	2	28	28	29
**Goal 11. Make cities and human settlements inclusive, safe, resilient, and sustainable**	**Keywords: Cities, Municipal, Waste, Communities, Settlements**									
1	Venkatesh et al.	Microbial degradation of plastics: Sustainable approach to tackling environmental threats facing big cities of the future	0	0	0	0	0	0	3	0
2	Adam et al.	Attitudinal and behavioural segments on single-use plastics in Ghana: Implications for reducing marine plastic pollution	0	0	0	0	3	2	3	0
3	Varkey et al.	Identifying barriers to reducing single-use plastic use in a coastal metropolitan city in Canada	0	0	0	2	0	0	2	3
4	Hossain et al.	Strategies for mitigating plastic wastes management problem: A lifecycle assessment study in Hong Kong	0	0	2	0	0	0	2	0
5	Dong et al.	Uncovering opportunity of low-carbon city promotion with industrial system innovation: Case study on industrial symbiosis projects in China	0	0	0	0	0	0	2	0
6	Wardrop et al.	Estimation of packaged water consumption and associated plastic waste production from household budget surveys	2	0	0	0	0	2	0	0
7	Abrokwah et al.	Drivers of single-use plastic waste generation: lessons from packaged water consumers in Ghana	0	0	0	0	0	2	2	0
8	Asari et al.	Analysis of mismanaged plastic waste in Samoa to suggest proper waste management in Pacific island countries	2	2	0	0	0	0	2	2
9	Salhofer et al.	Plastic recycling practices in vietnam and related hazards for health and the environment	2	0	2	0	0	2	2	2
10	Agusningtyas et al.	Processing of plastic waste from Klotok Landfill Kediri City with thermal cracking method	0	0	2	0	0	0	3	0
11	Kabir et al.	Plastic recycling from municipal solid waste in Jashore city of Bangladesh	0	0	0	0	0	0	2	0
12	Muslihun et al.	An Environmental Study on the Paid Plastic Bag Use Policy in the city of Semarang	0	0	0	0	0	0	0	2
13	Cohen et al.	Parks and Recreational Areas as Sinks of Plastic Debris in Urban Sites: The Case of Light-Density Microplastics in the City of Amsterdam, The Netherlands	3	0	0	0	0	2	3	3
14	Schuyler et al.	Environmental context and socio-economic status drive plastic pollution in Australian cities	3	0	0	0	0	2	3	3
15	Correa et al.	Challenges to reducing post-consumer plastic rejects from the MSW selective collection at two MRFs in São Paulo city, Brazil	3	0	0	0	0	3	3	3
16	Pertiwi et al.	Preliminary Study on Plastic Waste Handling in Semarang City - Indonesia: Estimated Generation and Existing Management	3	0	0	0	0	3	3	3
17	Taha Ahmed Al-Tayyar	Characteristics of Plastic Solid Wastes in Mosul City and Their Reuse	2	0	2	0	0	2	2	2
18	Brooks et al.	The Chinese import ban and its impact on global plastic waste trade	2	2	0	2	0	2	3	3
19	Fernández-Braña et al.	Looking beyond the banning of lightweight bags: analysing the role of plastic (and fuel) impacts in waste collection at a Portuguese city	2	0	0	2	0	0	2	0
20	Roche Cerasi et al.	Household plastic waste habits and attitudes: A pilot study in the city of Valencia	2	2	2	2	0	0	2	0
**Total**			**26**	**6**	**10**	**8**	**3**	**22**	**44**	**26**
**Goal 12. Ensure sustainable consumption and production patterns**	**Keywords: Sustainable, Production, Consumption, Footprint, Waste, Efficiency**									
1	Tang et al.	Toward Infinitely Recyclable Plastics Derived from Renewable Cyclic Esters	2	0	2	0	0	0	3	2
2	Nguyen Thi Khanh Chi	Ethical consumption behavior towards eco-friendly plastic products: Implication for cleaner production	2	2	2	2	0	0	0	0
3	Zhao et al.	Upcycling to Sustainably Reuse Plastics	2	0	3	0	0	3	3	2
4	Shangwa et al.	Conceptualization and design of a small pyrolysis plant for the sustainable production of paraffin from plastic waste	0	0	0	0	0	0	3	0
5	Gong et al.	Energy- and Labor-aware Production Scheduling for Sustainable Manufacturing: A Case Study on Plastic Bottle Manufacturing	0	0	3	0	0	0	0	0
6	Katie Conolon	Plastic roads: not all they're paved up to be	0	0	0	0	0	0	3	0
7	Milios et al.	Sustainability impact assessment of increased plastic recycling and future pathways of plastic waste management in Sweden	0	2	2	2	0	2	3	0
8	Deshpande et al.	Multi-criteria decision analysis (MCDA) method for assessing the sustainability of end-of-life alternatives for waste plastics: A case study of Norway	0	0	0	0	0	3	3	0
9	Evode et al.	Plastic waste and its management strategies for environmental sustainability	2	0	0	0	0	0	2	2
10	Lee et al.	Environmental Sustainability Framework for Plastic Waste Management—a Case Study of Bubble Tea Industry in Malaysia	0	0	2	0	2	0	3	2
11	Yuan et al.	Sustainability-inspired upcycling of waste polyethylene terephthalate plastic into porous carbon for CO2capture	0	0	2	0	0	0	3	3
12	Slusarczyk et al.	Solution for sustainable development: Provisions limiting the consumption of disposable plastic carrier bags in Poland	0	0	0	2	2	0	2	2
13	idambarampadmavathy et	Sustainable bio-plastic production through landfill methane recycling	3	0	2	0	0	0	3	2
14	Govil et al.	Lignocellulosic feedstock: A review of a sustainable platform for cleaner production of nature's plastics	2	0	0	0	0	0	2	0
15	Babayemi et al.	Ensuring sustainability in plastics use in Africa: consumption, waste generation, and projections	2	2	2	0	2	2	2	3
16	de Vargas Mores et al.	Sustainability and innovation in the Brazilian supply chain of green plastic	2	0	2	2	0	0	0	2
17	Burnley et al.	The environmental and financial benefits of recovering plastics from residual municipal waste before energy recovery	0	0	0	2	0	0	3	0
18	Gerassimidou et al.	Development of an integrated sustainability matrix to depict challenges and trade-offs of introducing bio-based plastics in the food packaging value chain	2	0	2	0	0	2	3	3
19	Lombardi et al.	Material flow analysis and sustainability of the Italian plastic packaging management	0	0	0	0	0	0	2	2
20	Herberz et al.	Sustainability assessment of a single-use plastics ban	2	3	0	0	0	0	3	1

21	Briassoulis et al.	Recirculation potential of post-consumer /industrial bio-based plastics through mechanical recycling - Techno-economic sustainability criteria and indicators	2	0	0	0	0	0	2	2
22	Borland et al.	Sustainability and sustainable development strategies in the U.K. plastic electronics industry	0	0	0	0	2	2	2	1
23	Cardamone et al.	About the environmental sustainability of the European management of WEEE plastics	2	0	2	3	0	0	3	0
24	Blanc et al.	Use of bio-based plastics in the fruit supply chain: An integrated approach to assess environmental, economic, and social sustainability	2	0	0	0	0	0	0	0
25	Sundqvist-Andberg et al.	Sustainability governance and contested plastic food packaging – An integrative review	2	2	0	0	2	2	2	2
26	Jefferson	WHITHER PLASTICS?—Petrochemicals, plastics and sustainability in a garbage-riddled world	2	2	2	0	0	2	3	2
27	Faisal Kabir et al.	End of life plastics to enhance sustainability of pavement construction utilizing a hybrid treatment of bio-oil and carbon coating	0	2	0	0	0	2	2	3
28	Moshood et al.	Sustainability of biodegradable plastics: New problem or solution to solve the global plastic pollution?	2	0	0	0	0	0	3	2
29	Ardolino et al.	How to enhance the environmental sustainability of WEEE plastics management: An LCA study	0	0	0	0	0	0	2	0
30	Mwanza et al.	Major Obstacles to Sustainability in the Plastic Industry	0	0	0	0	0	0	2	3
**Total**			**33**	**15**	**28**	**13**	**10**	**20**	**67**	**41**
**Goal 13. Take urgent action to combat climate change and its impacts**	**Keywords: Climate Change, Global Warming, Mitigation**									
1	Adyel et al.	Plastics in blue carbon ecosystems: a call for global cooperation on climate change goals	0	0	0	0	0	0	3	2
2	Ford et al.	The fundamental links between climate change and marine plastic pollution	2	2	2	0	0	2	3	3
3	Kane et al.	Reducing the environmental impacts of plastics while increasing strength: Biochar fillers in biodegradable, recycled, and fossil-fuel derived plastics	0	2	0	0	0	0	0	2
4	Tiso et al.	The metabolic potential of plastics as biotechnological carbon sources – Review and targets for the future	0	0	0	0	0	0	2	2
5	Stegmann et al.	The plastics integrated assessment model (PLAIA): Assessing emission mitigation pathways and circular economy strategies for the plastics sector	0	0	0	0	0	2	1	0
6	Shen et al.	(Micro)plastic crisis: Un-ignorable contribution to global greenhouse gas emissions and climate change	2	0	0	0	0	2	2	3
7	Cuello et al.	Impact of plastic film mulching on increasing greenhouse gas emissions in temperate upland soil during maize cultivation	2	0	0	0	0	0	0	0
8	Sharma et al.	Contribution of plastic and microplastic to global climate change and their conjoining impacts on the environment - A review	2	0	0	0	0	2	0	2
9	Bora et al.	Waste polypropylene plastic recycling toward climate change mitigation and circular economy: Energy, environmental, and technoeconomic perspectives	No	No	No	No	No	No	3	No
10	Liu et al.	How does circular economy respond to greenhouse gas emissions reduction: An analysis of Chinese plastic recycling industries	0	0	0	0	0	0	3	2
11	Zapata	The relationship between climate conditions and consumption of bottled water: A potential link between climate change and plastic pollution	2	0	0	0	2	0	0	0
12	Nicholson et al.	Manufacturing energy and greenhouse gas emissions associated with plastics consumption	2	0	2	0	0	0	2	2
13	Meys et al.	Achieving net-zero greenhouse gas emission plastics by a circular carbon economy	3	0	3	0	0	0	3	3
14	Kouloumpis et al.	Potential trade-offs between eliminating plastics and mitigating climate change: An LCA perspective on Polyethylene Terephthalate (PET) bottles in Cornwall	0	0	0	0	0	3	0	2
15	Sevigné-Itoiz et al.	Contribution of plastic waste recovery to greenhouse gas (GHG) savings in Spain	0	0	0	2	0	2	2	0
16	Chu et al.	Life-cycle GHG emissions and the associated carbon-peak strategies for PS, PVC, ABS	3	0	2	0	0	0	2	2
17	Lee et al.	Impact of plastic film mulching on global warming in entire chemical and organic cropping systems: Life cycle assessment	0	0	0	0	2	0	0	0
18	Borui Gu	Research on the Impact of Plastic Recycling Industry on Greenhouse Gas Emissions	2	0	0	0	0	1	2	1
19	Bauer et al.	Plastics and climate change breaking carbon lock-ins through three mitigation pathways	2	0	3	0	0	2	3	2
20	Liu et al.	How does the global plastic waste trade contribute to environmental benefits: Implication for reductions of greenhouse gas emissions?	0	0	0	0	0	3	3	0
21	Tyler Eddy	Climate change drowned out by plastic	0	0	0	0	0	3	3	0
22	Vea et al.	Inclusion of multiple climate tipping as a new impact category in life cycle assessment of polyhydroxyalkanoate (PHA)-based plastics	3	0	0	0	0	0	2	3
23	Sandberg et al.	Accounting for carbon flows into and from bio plastic in a national climate inventory	0	0	0	0	0	0	3	0
24	Barbara Demeneix	How fossil fuel-derived pesticides and plastics harm health, biodiversity, and the climate	3	2	2	0	0	2	0	0
25	Tickner et al.	Transitioning the Chemical Industry: The Case for Addressing the Climate, Toxics, and Plastics Crises	3	0	2	0	2	0	2	3
26	Karin Kvale	Implications of plastic pollution on global marine carbon cycling and climate	0	2	0	0	0	2	0	0
27	Jankowska et al.	Transforming the Plastic Production System Presents Opportunities to Tackle the Climate Crisis	3	0	0	0	0	0	2	2
28	Vishnuradhan et al.	Can plastics affect near surface layer ocean processes and climate?	0	0	0	0	0	3	3	2
29	Oktavilia et al.	Plastic Industry and World Environmental Problems	0	0	0	0	0	0	2	0
30	An et al.	Estimated material metabolism and life cycle GHG emission of major plastics in China - A commercial sector scale perspective	3	0	2	0	0	0	2	2
**Total**			**37**	**8**	**18**	**2**	**6**	**29**	**53**	**40**
**Goal 14. Conserve and sustainably use the oceans, seas and marine resources for sustainable development**	**Keywords: Marine, Pollution, Coast, Fishing**									
1	Xanthos et al.	International policies to reduce plastic marine pollution from single-use plastics (plastic bags and microbeads): A review	0	2	0	0	0	0	2	2
2	Schnurr et al.	Reducing marine pollution from single-use plastics (SUPs): A review	0	2	0	0	0	0	2	2
3	Villarrubia-Gómez et al.	Marine plastic pollution as a planetary boundary threat – The drifting piece in the sustainability puzzle	0	2	0	0	0	0	2	2
4	Vince et al.	Plastic pollution challenges in marine and coastal environments: from local to global governance	0	2	0	0	0	0	2	2
5	Wilcox et al.	Using expert elicitation to estimate the impacts of plastic pollution on marine wildlife	0	0	0	0	0	0	2	2
6	Thiel et al.	Impacts of marine plastic pollution from continental coasts to subtropical gyres-fish, seabirds, and other vertebrates in the SE Pacific	0	0	0	0	0	0	2	2
7	Schmaltz et al.	Plastic pollution solutions: emerging technologies to prevent and collect marine plastic pollution	0	2	2	0	0	0	2	2
8	Andrew Turner	Black plastics: Linear and circular economies, hazardous additives and marine pollution	2	2	0	0	0	0	2	2
9	Adam et al.	Policies to reduce single-use plastic marine pollution in West Africa	0	2	0	0	0	0	3	2
10	Lestari et al.	The impact of improper solid waste management to plastic pollution in Indonesian coast and marine environment	0	2	2	0	0	0	3	2
11	Compa et al.	Risk assessment of plastic pollution on marine diversity in the Mediterranean Sea	0	0	0	0	0	0	2	2
12	Oliveira et al.	Marine Environmental Plastic Pollution: Mitigation by Microorganism Degradation and Recycling Valorization	2	0	0	0	0	0	2	2
13	Borrelle et al.	Why we need an international agreement on marine plastic pollution	0	0	0	0	0	2	3	2
14	Clayton et al.	Policy responses to reduce single-use plastic marine pollution in the Caribbean	3	2	0	0	0	2	2	2
15	Akinwumi et al.	Marine plastic pollution and affordable housing challenge: Shredded waste plastic stabilized soil for producing compressed earth bricks	0	2	0	0	0	0	2	2
16	Manfra et al.	Biodegradable polymers: A real opportunity to solve marine plastic pollution?	3	0	0	0	0	0	0	2
17	Ghayebzadeh et al.	Estimation of plastic waste inputs from land into the Caspian Sea: A significant unseen marine pollution	0	0	0	0	0	0	2	0
18	Marks et al.	The geopolitical economy of Thailand's marine plastic pollution crisis	2	2	0	0	0	2	3	3
19	Chowdhury et al.	Estimating marine plastic pollution from COVID-19 face masks in coastal regions	0	0	0	0	0	0	3	0
20	Walther et al.	Plastic pollution of four understudied marine ecosystems: a review of mangroves, seagrass meadows, the Arctic Ocean and the deep seafloor	0	0	0	0	0	0	2	2
21	Green et al.	Impacts of discarded plastic bags on marine assemblages and ecosystem functioning	2	0	0	0	0	2	0	0
22	Willis et al.	Cleaner seas - reducing marine pollution	2	2	0	0	2	2	2	3
23	Kyriakopoulos et al.	Investigating the Human Impacts and the Environmental consequences of microplastics disposal	3	0	0	0	0	2	2	2
24	Wang et al.	Seawater-Degradable Polymers—Fighting the Marine Plastic Pollution	3	0	0	0	0	0	2	2
25	Issifu et al.	A review of the production, recycling and management of marine plastic pollution	0	2	2	0	0	2	2	0
26	Kwon et al.	Global styrene oligomers monitoring as new chemical contamination from polystyrene plastic marine pollution	2	0	0	0	0	0	2	0
27	Peter Dauvergne	The power of environmental norms: marine plastic pollution and the politics of microbeads	3	2	0	0	0	0	2	3
28	Peter Dauvergne	Why is the global governance of plastic failing the oceans?	2	2	0	0	0	2	3	3
29	Abbott et al.	Reducing marine plastic pollution: Policy insights from economics	2	2	0	0	0	2	2	2
30	Harris et al.	Using citizen science to evaluate extended producer responsibility policy to reduce marine plastic debris shows no reduction in pollution levels	0	0	0	0	0	2	3	0
**Total**			**31**	**32**	**6**	**0**	**2**	**20**	**63**	**52**
**Goal 15. Protect, restore and promote sustainable use of terrestrial ecosystems, sustainably manage forests, combat desertification, and halt and reverse land degradation and halt biodiversity loss**	**Keywords: Ecosystem, Soil, Land, Terrestrial, Wildlife, Wetlands**									
1	Dris et al.	Beyond the ocean: Contamination of freshwater ecosystems with (micro-)plastic particles	0	0	0	0	0	2	2	2
2	Chae et al.	Current research trends on plastic pollution and ecological impacts on the soil ecosystem: A review	3	0	0	0	0	2	2	2
3	Kumar et al.	Impacts of plastic pollution on ecosystem services, sustainable development goals, and need to focus on circular economy and policy interventions	2	2	0	2	2	2	2	0
4	Strungaru et al.	Micro- (nano) plastics in freshwater ecosystems: Abundance, toxicological impact and quantification methodology	0	0	0	0	0	2	2	0
5	Qadeer et al.	Agricultural plastic mulching as a potential key source of microplastic pollution in the terrestrial ecosystem and consequences	2	2	0	0	0	0	2	0
6	Bandopadhyay et al.	Biodegradable plastic mulch films: Impacts on soil microbial communities and ecosystem functions	0	0	0	0	0	0	2	0
7	Koskei et al.	Effects of increased plastic film residues on soil properties and crop productivity in agro-ecosystem	2	0	0	0	0	0	2	0
8	Yu et al.	Micro plastics in soil ecosystem – A review of sources, fate, and ecological impact	2	2	0	0	0	0	2	0
9	Hurley et al.	Plastic waste in the terrestrial environment	2	2	0	0	0	2	2	2
10	Astner et al.	Mechanical formation of micro- and nano-plastic materials for environmental studies in agricultural ecosystems	2	2	0	0	2	0	2	2
11	Azevedo-Santos et al.	Plastic pollution: A focus on freshwater biodiversity	3	2	0	0	0	0	3	2
12	Schell et al.	Occurrence, Fate and Fluxes of Plastics and Microplastics in Terrestrial and Freshwater Ecosystems	3	3	0	0	0	0	2	2
13	Zhao et al.	Fate of plastic film residues in agro-ecosystem and its effects on aggregate-associated soil carbon and nitrogen stocks	3	0	0	0	0	0	0	0
14	Blettler et al.	Plastic pollution in freshwater ecosystems: macro-, meso-, and microplastic debris in a floodplain lake	0	0	0	0	0	0	2	0
15	Malizia et al.	Terrestrial ecologists should stop ignoring plastic pollution in the Anthropocene time	0	0	0	0	0	0	2	0
16	Fan et al.	A review on the occurrence and influence of biodegradable microplastics in soil ecosystems: Are biodegradable plastics substitute or threat?	3	2	0	0	0	0	2	2

17	Susanti et al.	Microplastics and the Impact of Plastic on Wildlife: A Literature Review	3	0	0	0	0	0	0	0
18	Wang et al.	Uptake, translocation, and biological impacts of micro(nano)plastics in terrestrial plants: Progress and prospects	3	0	0	0	0	0	0	0
19	Zhao et al.	Microscopic anthropogenic litter in terrestrial birds from Shanghai, China: Not only plastics but also natural fibers	3	2	0	0	0	0	2	0
20	Maclean et al.	The terrestrial plastisphere: Diversity and polymer-colonizing potential of plastic-associated microbial communities in soil	0	0	0	0	0	0	0	0
21	Mo et al.	Environmental fate and impacts of biodegradable plastics in agricultural soil ecosystems	3	2	0	0	0	0	2	0
22	Amaneesh et al.	Gross Negligence: Impacts of Microplastics and Plastic Leachates on Phytoplankton Community and Ecosystem Dynamics	2	2	0	0	0	0	2	2
23	Guimarães et al.	Toxic effects of naturally-aged microplastics on zebrafish juveniles: A more realistic approach to plastic pollution in freshwater ecosystems	2	0	0	0	0	0	0	0
24	Kasavan et al.	Plastic pollution in water ecosystems: A bibliometric analysis from 2000 to 2020	0	0	0	0	0	0	2	0
25	Tudor et al.	The issue of plastic and microplastic pollution in soil	0	0	0	0	0	0	2	0
26	Ullah et al.	Micro(nano)plastic pollution in terrestrial ecosystem: emphasis on impacts of polystyrene on soil biota, plants, animals, and humans	2	2	0	0	0	2	2	0
27	Zhang et al.	Microplastics pollution from different plastic mulching years accentuate soil microbial nutrient limitations	2	0	0	0	0	0	2	0
28	Joos et al.	Soil under stress: The importance of soil life and how it is influenced by (micro)plastic pollution	0	0	0	0	0	0	0	0
29	Andrea et al.	Unraveling the role of plastic waste pollution in the Amvrakikos Wetlands National Park, Greece: The stakeholders' views	2	2	0	0	0	2	2	2
30	Chen et al.	Feasibility of using plastic wastes as constructed wetland substrates and potential for pharmaceuticals and personal care products removal	0	0	0	0	0	2	2	2
**Total**			**49**	**27**	**0**	**2**	**4**	**16**	**49**	**20**
**Goal 16. Promote peaceful and inclusive societies for sustainable development, provide access to justice for all and build effective, accountable and inclusive institutions at all levels**	**Keywords: Justice, Peace, Regulation, Legislation, Governance, Policy**									
1	Katie Conolon	Adaptive injustice: Responsibility to act in the plastics economy	3	2	0	0	0	2	3	3
2	Kim et al.	Designing for Green and Grey: Insights from Single-Use Plastic Water Bottles	0	2	3	0	0	0	0	3
3	Bahl et al.	Don't Transfer Waste, Transform Waste: A Sustainable Approach towards Zero Waste Events Initiative during Hockey World Cup in Bhubaneshwar, India	0	0	0	0	0	0	3	3
4	Garcia et al.	Marine Plastic Pollution in Asia: All Hands on Deck!	2	2	0	0	3	0	3	3
5	Liboiron et al.	Abundance and types of plastic pollution in surface waters in the Eastern Arctic (Inuit Nunangat) and the case for reconciliation science	3	3	0	2	0	0	3	3
6	Paul Jobin	Our 'good neighbor' Formosa Plastics: petrochemical damage(s) and the meanings of money	2	2	2	0	0	0	2	3
7	Hsu et al.	Closing the loop on plastics in Europe - The role of data, information and knowledge	2	3	0	0	0	2	2	3
8	Poto et al.	Suggestions for a systematic regulatory approach to ocean plastics	2	2	0	0	0	0	2	3
9	Owens et al.	Mopping Up or Turning Off the Tap? Environmental Injustice and the Ethics of Plastic Pollution	2	2	0	0	0	0	2	3
10	Wang et al.	Circular Economy and the Changing Geography of International Trade in Plastic Waste	2	2	0	3	0	0	3	3
11	Margaret Handley	Confronting the plasticine: promise in a world wrapped in plastic	0	0	0	3	0	0	3	3
12	Shirley et al.	Unwrapping Victoria's general environmental duty to plastics communities: Synthetic statutes	0	0	0	3	0	0	3	3
13	Alice Mah	Future-proofing capitalism: The paradox of the circular economy for plastics	3	0	0	3	0	2	3	3
14	Ortiz et al.	A Regional Response to a Global Problem: Single Use Plastics Regulation in the Countries of the Pacific Alliance	2	2	0	0	0	0	3	3
15	Carlini et al.	Advancing the international regulation of plastic pollution beyond the united nations environment assembly resolution on marine litter and microplastics	0	0	0	0	0	0	3	2
16	Steensgaard et al.	From macro- to microplastics - Analysis of EU regulation along the life cycle of plastic bags	2	2	0	2	0	2	3	3
17	Tan et al.	Moving policy and regulation forward for single-use plastic alternatives	2	0	0	0	0	0	2	3
18	Tham Hoang	Plastic pollution: Where are we regarding research and risk assessment in support of management and regulation?	2	2	0	0	0	0	3	3
19	Saputra et al.	Combining the concept of green accounting with the regulation of prohibition of disposable plastic use	2	2	0	0	0	0	3	3
20	Tessnow-von Wysocki et al	Plastics at sea: Treaty design for a global solution to marine plastic pollution	3	2	0	0	0	0	2	3
**Total**			**34**	**30**	**5**	**16**	**3**	**8**	**51**	**59**
**Goal 17. Strengthen the means of implementation and revitalize the global partnership for sustainable development**	**Keywords: Partnership, Cooperation, Global, Trade, Market**									
1	Bank et al.	Global plastic pollution observation system to aid policy	0	0	0	0	0	0	3	3
2	Marcus Haward	Plastic pollution of the world's seas and oceans as a contemporary challenge in ocean governance	0	0	0	0	0	0	3	3
3	Freeman et al.	Between source and sea: The role of wastewater treatment in reducing marine microplastics	0	0	0	0	0	0	3	2
4	Duong et al.	A Model Template Green Environment Initiative for Recycling plastic bottles with progressive entrepreneurship partnership	0	No	3	0	0	2	3	0
5	Kandziora et al.	The important role of marine debris networks to prevent and reduce ocean plastic pollution	0	0	0	0	0	0	3	3
6	Li et al.	The collapse of global plastic waste trade: Structural change, cascading failure process and potential solutions	0	0	0	0	0	0	3	3
7	Galaiduk et al.	Transnational Plastics: An Australian Case for Global Action	0	0	0	0	0	0	3	3
8	Barrowclough et al.	Transforming the Global Plastics Economy: The Role of Economic Policies in the Global Governance of Plastic Pollution	3	2	2	2	0	3	3	3
9	Kuan et al.	Towards regional cooperation on sustainable plastic recycling: comparative analysis of plastic waste recycling policies and legislations in Japan and Malaysia	2	0	0	0	0	0	3	3
10	Finska et al.	Troubled waters - Where is the bridge? Confronting marine plastic pollution from international watercourses	3	0	0	0	0	2	0	3
11	Sun et al.	Regional cooperation in marine plastic waste cleanup in the south china sea region	3	0	0	0	0	0	3	3
12	Nancy Lamontagne	Academic-Industry Partnerships: Preparing Tomorrow's Plastics Professionals	2	0	2	0	0	0	0	0
13	Stuchtey et al.	Project stop: City partnerships to prevent ocean plastics in Indonesia	0	0	0	0	0	3	3	3
14	Raubenheimer et al.	Rethinking global governance of plastics – The role of industry	0	0	0	0	0	3	3	2
15	Fadeeva et al.	'Unlocking circular economy for prevention of marine plastic pollution: An exploration of G20 policy and initiatives'	3	2	2	0	0	3	3	3
16	Nielsen et al.	Politics and the plastic crisis: A review throughout the plastic life cycle	3	3	0	0	0	2	3	3
17	Shruti et al.	Strengthening citizen science partnerships with frontline sanitation personnel to study and tackle plastic pollution	0	0	0	0	0	0	3	3
18	Micah Landon-Lane	Corporate social responsibility in marine plastic debris governance	2	3	2	0	0	3	3	3
19	Shah et al.	Plastics waste metabolism in a Petro-Island state: Towards solving a "wicked problem" in trinidad and tobago	0	0	2	0	0	2	3	3
20	Bagai et al.	Beating plastic pollution: UNEP's priorities and partnership in India	3	3	0	0	0	0	3	2
**Total**			**24**	**13**	**13**	**2**	**0**	**23**	**54**	**51**

Interactions between SDGs (According to Scopus mapping system of how the literature is linked to the achievement of SDGs. Note: The literature belonging to a particular SDG is not mapped to the same SDG even if they are mentioned in Scopus)

SDG	1	2	3	4	5	6	7	8	9	10	11	12	13	14	15	16	17
**1**	0	0	0	0	0	1	0	3	2	2	4	4	0	2	0	1	9
**2**	0	0	5	0	0	6	0	7	2	0	2	6	4	4	0	0	9
**3**	1	2	0	0	0	2	1	1	1	1	2	7	1	7	0	0	3
**4**	0	0	4	0	0	0	1	5	2	0	3	15	1	6	0	0	4
**5**	0	0	1	3	0	2	0	2	3	3	6	6	1	4	1	1	11
**6**	2	0	6	2	0	0	0	2	3	0	10	5	0	2	3	1	12
**7**	0	0	0	0	0	2	0	1	5	0	2	14	3	0	0	0	2
**8**	1	0	0	0	0	0	1	0	7	1	1	9	1	3	0	1	10
**9**	0	0	0	0	0	0	2	2	0	0	3	16	2	6	0	1	9
**10**	2	2	3	2	0	0	1	1	0	0	6	11	1	6	2	0	9
**11**	0	0	1	0	0	4	0	1	6	1	0	14	1	8	1	0	10
**12**	0	0	1	0	0	0	3	9	18	1	5	0	7	5	5	0	9
**13**	0	2	3	0	0	1	3	4	13	0	0	17	0	7	0	0	6
**14**	0	0	5	0	0	0	0	1	2	1	5	14	1	0	2	2	5
**15**	0	4	5	1	0	5	0	3	1	0	3	4	2	14	0	2	3
**16**	0	1	1	1	0	1	0	2	3	3	1	6	2	9	0	0	5
**17**	0	0	2	0	0	2	0	4	4	2	3	10	1	15	1	0	0

Interactions between SDGs apart from the ones mentioned in Scopus (Note: The literature belonging to a particular SDG is not mapped to the same SDG even if they are mentioned in Scopus)

SDG	1	2	3	4	5	6	7	8	9	10	11	12	13	14	15	16	17
**1**	0	0	1	2	0	1	0	4	4	6	3	4	2	1	0	0	0
**2**	0	0	1	0	0	5	0	2	0	5	5	5	6	10	17	1	3
**3**	2	6	0	1	7	9	1	4	8	13	17	12	4	12	18	2	9
**4**	1	0	0	0	2	5	0	7	11	3	13	5	14	11	15	0	14
**5**	6	2	1	4	0	8	1	11	6	8	8	13	2	4	6	0	3
**6**	9	0	9	5	6	0	0	1	9	8	7	11	1	7	5	0	2
**7**	0	0	0	1	0	0	0	19	15	1	17	5	12	0	0	0	1
**8**	2	0	0	8	0	0	3	0	13	12	17	10	6	1	3	2	9
**9**	6	0	3	7	0	4	2	23	0	6	19	13	21	13	17	5	14
**10**	9	1	7	9	0	1	0	9	14	0	13	6	10	9	14	7	5
**11**	4	0	4	10	0	4	3	5	11	6	0	6	13	7	13	1	8
**12**	3	7	10	0	0	4	12	11	11	7	18	0	21	21	20	2	6
**13**	1	5	4	1	0	2	11	19	14	5	25	11	0	22	28	15	13
**14**	0	3	13	5	1	14	1	13	22	8	23	16	21	0	28	16	21
**15**	1	19	22	3	1	11	1	6	13	4	14	22	9	16	0	6	9
**16**	1	1	2	0	1	5	0	5	7	8	13	13	5	6	12	0	14
**17**	1	0	2	1	0	2	2	10	13	9	13	10	8	4	16	9	0

Total interactions between SDGs (Note: The literature belonging to a particular SDG is not mapped to the same SDG even if they are mentioned in Scopus)

SDG	1	2	3	4	5	6	7	8	9	10	11	12	13	14	15	16	17
**1**	0	0	1	2	0	2	0	7	6	8	7	8	2	3	0	1	9
**2**	0	0	6	0	0	11	0	9	2	5	7	11	10	14	17	1	12
**3**	3	8	0	1	7	11	2	5	9	14	19	19	5	19	18	2	12
**4**	1	0	4	0	2	5	1	12	13	3	16	20	15	17	15	0	18
**5**	6	2	2	0	0	10	1	13	9	11	14	19	3	8	7	1	14
**6**	11	0	15	0	6	0	0	3	12	8	17	16	1	9	8	1	14
**7**	0	0	0	0	0	2	0	20	20	1	19	19	15	0	0	0	3
**8**	3	0	0	0	0	0	4	0	20	13	18	19	7	4	3	3	19
**9**	6	0	3	0	0	4	4	25	0	6	22	29	23	19	17	6	23
**10**	11	3	10	0	0	1	1	10	14	0	19	17	11	15	16	7	14
**11**	4	0	5	0	0	8	3	6	17	7	0	20	14	15	14	1	18
**12**	3	7	11	0	0	4	15	20	29	8	23	0	28	26	25	2	15
**13**	1	7	7	0	0	3	14	23	27	5	25	28	0	29	28	15	19
**14**	0	3	18	0	1	14	1	14	24	9	28	30	22	0	30	18	26
**15**	1	23	27	0	1	16	1	9	14	4	17	26	11	30	0	8	12
**16**	1	2	3	0	1	6	0	7	10	11	14	19	7	15	12	0	19
**17**	1	0	4	0	0	4	2	14	17	11	16	20	9	19	17	9	0

SDG	Keywords	Number of occurences
SDG1	Human	15
	Poverty	21
	Article	10
	Plastic	10
	Sustainable Development	10
SDG2	Food supply	13
	Agriculture	13
	Food security	10
	Cultivation	8
	China	7
SDG3	Plastic	50
	Human	45
	Public health	28
	Plastic waste	25
	Health risks	25
SDG4	Plastics	11
	Education	9
	Human	7
	Awareness	5
	Environmental education	6
SDG5	Female	99
	Human	98
	Adult	62
	Phthalic acid	27
	Pregnancy	29
SDG6	Sanitation	65
	Human	42
	Drinking water	31
	Non human	26
	Water quality	25
SDG7	Elastomers	20
	Plastic recycling	16
	Pyrolysis	18
	Plastic waste	14
	Energy efficiency	13
SDG8	Recycling	10
	Waste management	7
	Plastic recycling	9
	Circular economy	8
	3D Printers	5
SDG9	Elastomers	12
	Plastic waste	8
	Recycling	8
	Waste management	8
	Innovation	13
SDG10	Plastic	60
	Waste management	44
	Waste disposal	21
	Plastic pollution	16
	Plastic recycling	17
SDG11	Plastic	24
	Plastic waste	18
	Human	12
	Waste disposal	11
	Waste management	12
SDG12	Sustainable Development	67
	Elastomers	36
	Plastics	36
	Recycling	27
	Environmental sustainabilit	24
SDG13	Climate change	42
	Plastic	31
	Ecosystem	10
	Mulching	10
	China	9
SDG14	Plastic	79
	Marine Pollution	85
	Pollution	44
	Plastic pollution	46
	Marine environment	41
SDG15	Plastic	88
	Plastic waste	49
	Microplastic	47
	Ecosystem	42
	Non human	41
SDG16	Human	20
	Elastomers	10
	Justice	7
	Plastic products	8
	Environmental justice	7
SDG17	Waste management	104
	Sustainable Development	130
	Plastic recycling	91
	Circular economy	67
	International cooperation	60

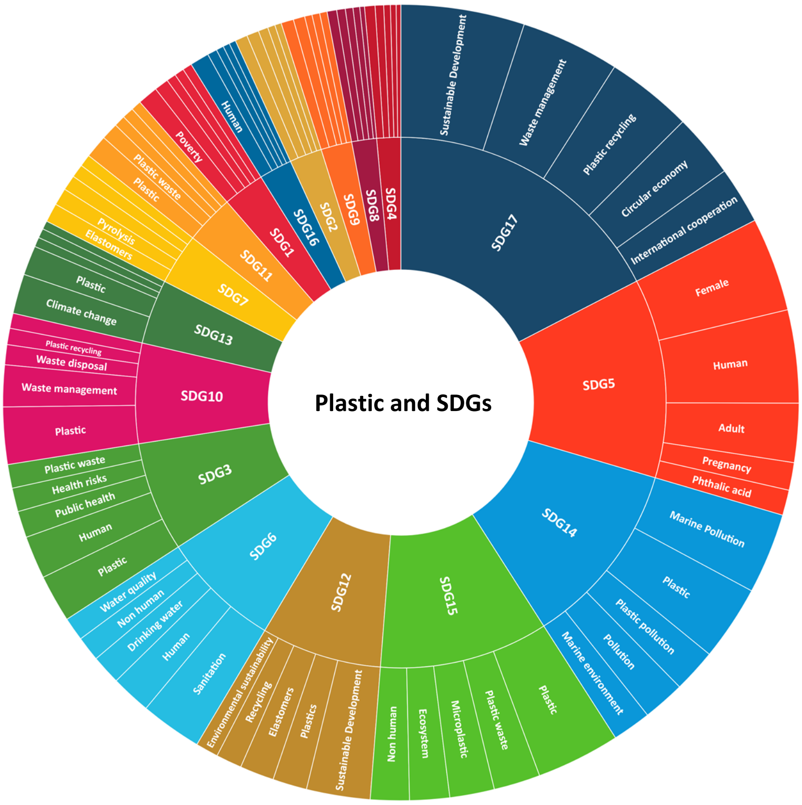

Supplement: Supplementary file 2 — Supporting File 2: gch270099‐sup‐0002‐SuppMat.pdf. [file GCH2-10-e00033-s002.pdf]
